# Genome-Wide Analysis of Differentially Expressed Genes and Splicing Isoforms in Clear Cell Renal Cell Carcinoma

**DOI:** 10.1371/journal.pone.0078452

**Published:** 2013-10-23

**Authors:** Alessio Valletti, Margherita Gigante, Orazio Palumbo, Massimo Carella, Chiara Divella, Elisabetta Sbisà, Apollonia Tullo, Ernesto Picardi, Anna Maria D’Erchia, Michele Battaglia, Loreto Gesualdo, Graziano Pesole, Elena Ranieri

**Affiliations:** 1 Institute of Biomembranes and Bioenergetics, CNR, Bari, Italy; 2 Department of Surgery and Medical Sciences, University of Foggia, Foggia, Italy; 3 Medical Genetics Unit, IRCCS “Casa Sollievo della Sofferenza”, San Giovanni Rotondo, Italy; 4 Department of Emergency and Organ Transplantation, University of Bari “Aldo Moro”, Bari, Italy; 5 Institute of Biomedical Technologies U.O.S. Bari, CNR, Bari, Italy; 6 Department of Biosciences, Biotechnologies and Biopharmaceutics, University of Bari “Aldo Moro”, Bari, Italy; Institute of Clinical Physiology, c/o Toscana Life Sciences Foundation, Italy

## Abstract

Clear cell renal cell carcinoma (ccRCC) is the most common malignant renal epithelial tumor and also the most deadly. To identify molecular changes occurring in ccRCC, in the present study we performed a genome wide analysis of its entire complement of mRNAs. Gene and exon-level analyses were carried out by means of the Affymetrix Exon Array platform. To achieve a reliable detection of differentially expressed cassette exons we implemented a novel methodology that considered contiguous combinations of exon triplets and candidate differentially expressed cassette exons were identified when the expression level was significantly different only in the central exon of the triplet. More detailed analyses were performed for selected genes using quantitative RT-PCR and confocal laser scanning microscopy. Our analysis detected over 2,000 differentially expressed genes, and about 250 genes alternatively spliced and showed differential inclusion of specific cassette exons comparing tumor and non-tumoral tissues. We demonstrated the presence in ccRCC of an altered expression of the PTP4A3, LAMA4, KCNJ1 and TCF21 genes (at both transcript and protein level). Furthermore, we confirmed, at the mRNA level, the involvement of CAV2 and SFRP genes that have previously been identified. At exon level, among potential candidates we validated a differentially included cassette exon in DAB2 gene with a significant increase of DAB2 p96 splice variant as compared to the p67 isoform. Based on the results obtained, and their robustness according to both statistical analysis and literature surveys, we believe that a combination of gene/isoform expression signature may remarkably contribute, after suitable validation, to a more effective and reliable definition of molecular biomarkers for ccRCC early diagnosis, prognosis and prediction of therapeutic response.

## Background

Renal cell carcinoma (RCC) is the most widespread adult renal epithelial cancer, accounting for more than 90% of all renal malignancies; clear cell Renal Cell Carcinoma (ccRCC) is the most common RCC subtype, affecting about 70% of the surgical cases [1,2]. This carcinoma typically contains a regular network of small, thin-walled blood vessels, that are a diagnostic hallmark. In addition, the cytoplasm of the tumor cells is clear as a result of a strong intra-cytoplasmic accumulation of glycogen, cholesterol, neutral lipids, and phospholipids [1]. 

The genetic and molecular alteration pattern of ccRCC has been extensively investigated and has become an important criterion for classification. ccRCC constitutes a typical manifestation of several familial renal cell cancer syndromes (e.g., Birt-Hogg-Dubé and constitutional chromosome 3 translocation syndromes) but occurs mainly in von Hippel-Lindau disease (VHL), a syndrome caused by germline mutations of the VHL tumor suppressor gene, located on chromosome 3p25-26 [3]. The loss or deficiency of VHL (observed in up to 86% of cases) results in an increased accumulation and activity of the hypoxia-inducible transcription factor (HIF), which in turns activates the expression of genes involved in angiogenesis (VEGF, PDGF, SDF, CXCR4, TGFB1, and CTGF), glucose uptake and metabolism (HK2 and PDK4), pH control (CAIX and CAXII), invasion/metastasis (MMP1, SDF, CXCR4, and MST1R), proliferation, and survival (TGFB1) [4]. The products encoded by these genes could constitute new attractive targets for future therapies aimed to inhibit tumor cell growth mainly acting on angiogenesis and mTOR pathways [5]. A very recent study by the Cancer Genome Atlas Research Network identified a recurrent pattern in ccRCC that correlates with tumor stage and severity and offers new views on the opportunities for disease treatment. This remodelling of the cellular metabolism is characterized by the down-regulation of genes involved in the TCA cycle, decreased AMPK and PTEN protein levels, up-regulation of the pentose phosphate pathway and the glutamine transporter genes, increased acetyl-CoA carboxylase protein, and altered promoter methylation of miR-21 (also known as MIR21) and GRB10 [6]. Indeed, in the last 5 years, more targeted therapies have offered a significant increase in progression-free survival of ccRCC patients; but despite their better efficacy and tolerability than chemotherapy, the majority of patients will eventually develop resistant disease and finally succumb [7]. This lack of therapeutic efficacy highlights the need to identify new molecular markers able to discriminate RCC for an early diagnosis, prognosis and for a better prediction of the therapeutic response. 

Gene expression microarrays and RNA-seq represent two distinct and efficient methods of exploring the cancer transcriptome. Recent advances in technology and data analysis allow large-scale genome-wide investigations that go far beyond the single gene-level analysis. Indeed, both techniques can be used to investigate at the same time multiple layers of gene expression regulation, ranging from epigenetic regulation to alternative splicing and non-coding RNAs. Hence, these new technologies open new avenues for analyzing the ccRCC transcriptome in order to identify molecular signatures able to discriminate not only between kidney carcinomas and normal tissue, but also among different types and subtypes of RCC; the ability to recognize specific biological pathways that characterize each tumor consents a better stratification of patients into prognostic risk groups leading to more targeted therapeutic interventions [8]. 

The majority of studies of ccRCC have focused on gene-level coding and non-coding RNAs expression analysis. Several studies identified molecular signatures able to discriminate between metastatic and non-metastatic tumors, thus proving their potential as putative prognostic markers [9-11]. It has also been shown that distinct sets of genes are regulated either as a response to tumor growth or as an early event that promotes tumor growth [12]. A recently published meta-analysis of gene-expression in ccRCC revealed two distinct tumor subsets showing different panels of overexpressed genes and consequently a different prognosis, and a third small tumor subset that might represent a new variant of ccRCC [2,13]. The differential expression of long non-coding RNAs has also been investigated in ccRCC by microarray technology providing new insights into tumor development [14], and by RNA-seq. This methodology identified 5 miRNAs discriminating between non-recurrent versus recurrent and metastatic disease and 12 uniquely distinguished non-recurrent versus metastatic disease, while two different in silico prediction pipelines identified 23 previously unknown miRNAs [15]. Gene expression high-throughput technologies have been extensively applied in ccRCC studies, but to our knowledge, only few investigations have focused on genome-wide alterations of the alternative splicing patterns. A very recent RNA-Seq study detected 113 tumor-specific alternatively spliced genes among which a renal-specific switch between the epithelial FGFR2 IIIb isoform to mesenchymal FGFR2 IIIc isoform [16].

As reported above, some studies have been carried out, but so far, existing data are limited and there is still need for a quantitative genome-wide expression profiling using robust technology to gain a better knowledge in ccRCC gene deregulation. In the present study, we investigated gene expression alterations in ccRCC at both gene and exon level in order to detect additional reliable diagnostic markers and new potential therapeutic targets. Exploiting Affymetrix Exon Array technology, we identified a set of genes differentially expressed in ccRCC tumoral tissue compared to the adjacent non-tumoral (NT) renal tissue of the same patient. Among these genes, we identified four novel ccRCC-associated biomarkers with sensitivity ranging from 77.8% to 100%, suggesting they could be used in some combination with previously identified markers. Obviously further validations are needed in both research and clinical practice. Finally, we developed a novel workflow for exon-level analysis that allowed us to identify a set of exons differentially skipped in ccRCC sample compared to the paired NT renal tissue [17,18]. In this way, we identified four candidate exons that were further validated by qRT-PCR. One of the four candidates resulted significantly tumor-associated after qRT-PCR validation.

## Materials and Methods

### Clinical samples

RCC tissue samples and signed informed consent were obtained from 20 patients with a computed tomography CT confirmed renal mass following a protocol approved by the ethics committee of Bari Province Hospitals. Patients main demographic and clinical features are summarized in Table 1. At the time of surgery, all the patients showed no evidence of other diseases except for one. Tumor samples were staged using the latest TNM-classification and graded according to Fuhrman, this last information was taken into account for expression analysis [19,20]. Immediately after surgery, tumor and paired adjacent non-tumoral renal parenchyma denominated “non-tumoral renal tissue” (NT) were (separately) stored frozen at -80°C according to a standard procedure. Inclusion criteria were histologically confirmed RCC, of the clear cell subtype, and patients not previously submitted to pre-operative therapy.

**Table 1:** Clinical samples characteristics.

**Patient ID**	**Status**	**Date of Collection**	**Gender**	**Age**	**Fuhrman Grade**	**TNM Classification**	**Assay**
**PS_21**	ccRCC	27/04/2004	M	60	G2	T3b N0 M1	qRT-PCR
**PS_22**	ccRCC	28/04/2004	F	69	G2	T3b N0 M0	qRT-PCR
**PS_41**	ccRCC	02/05/2000	M	51	G2	T1b N0 M0	EA
**PS_43**	ccRCC	28/08/2007	F	82	G2	T1a Nx M0	EA/qRT-PCR
**PS_44**	ccRCC	12/07/2007	F	72	G1	pT1 N0 M0	qRT-PCR
**PS_45**	ccRCC	06/05/2007	M	61	G2	pT1a N0 M0	qRT-PCR
**PS_46**	ccRCC	03/11/2007	M	78	G2	pT1 N0 M0	EA/qRT-PCR
**PS_47**	ccRCC	31/08/2007	F	71	G3	pT4 N0 M0	qRT-PCR
**PS_50**	ccRCC	19/01/2007	M	59	G3	pT3b N0 M0	EA/qRT-PCR
**PS_55**	ccRCC	02/07/2007	F	52	G3	T3b N0 M0	EA/qRT-PCR
**PS_56**	ccRCC	22/02/2007	M	62	G3	T2 N0 M0	EA
**PS_58**	ccRCC	10/11/2008	M	46	G1	T1a Nx M0	EA/qRT-PCR
**PS_59**	ccRCC	13/11/2008	F	82	G1	T3a Nx M0	qRT-PCR
**PS_60**	ccRCC	27/03/2009	M	54	G2	T2a Nx M0	EA/qRT-PCR
**PS_61**	ccRCC	27/05/2009	M	48	G1	T2a Nx M0	qRT-PCR
**PS_62**	ccRCC	03/11/2008	M	68	G2	T1b N0 M0	EA/qRT-PCR
**PS_66**	ccRCC	26/01/2009	F	67	G3	T1b Nx M0	qRT-PCR
**PS_67**	ccRCC	20/05/2009	F	59	G1	T1b Nx M0	EA/qRT-PCR
**PS_68**	ccRCC	15/06/2009	M	66	G2	T1b Nx M0	qRT-PCR
**PS_69**	ccRCC	23/01/2009	F	55	G1	T1a Nx M0	qRT-PCR

Fuhrman and tumor/node/metastasis (TNM) classifications are reported for each tumor sample. For each sample, paired adjacent non-tumoral renal parenchyma was also collected and analyzed. EA = Exon Array.

Central review of tissue blocks was performed by an experienced uropathologist and only blocks with >95% viable tumor (ccRCC) or NT tissue were included in this study. 

In total, 10 RCC tumors and their matched non-tumor kidney tissues were used for exon array analysis, whereas qRT-PCR validations were performed on an extended set of samples for a total of 36 tissues consisting of 18 paired ccRCC and NT kidney tissue. Confocal laser scanning microscopy was performed on 6 ccRCC renal biopsies and compared with their respective normal kidney portion.

### RNA extraction and quality assessment

Collected samples were processed for total RNA extraction from 50-100 mg of fresh frozen tissue using the TRIzol reagent (Invitrogen^TM^, Life Technologies^TM^). RNA samples were further purified using the RNeasy Mini kit (Qiagen^®^) according to the manufacturer’s instructions. Purified RNA was then quantified using the NanoDrop^TM^ 1000 Spectrophotometer (NanoDrop Technologies, Berlin, Germany) and RNA quality was determined by running aliquots on the 2100 Bioanalyzer (Agilent Technologies, Waldbronn, Germany). For microarray experiments, samples with a 28S/18S ratio < 1.0, RNA integrity number (RIN) < 6.5 or a concentration < 300 ng/μl were excluded.

### Microarray experiments and data analysis

1μg of total RNA per sample was used as starting material. rRNA was ﬁrst removed using the RiboMinus Human/Mouse Transcriptome Isolation Kit (Invitrogen^TM^, Life Technologies^TM^) and cDNA synthesis was performed using the GeneChip WT cDNA Synthesis Kit (Affymetrix, Santa Clara, CA). The cDNA was fragmented and labeled with biotin using the Affymetrix GeneChip WT Terminal Labeling Kit. Biotinylated targets were hybridized onto a Affymetrix GeneChip Human Exon 1.0 ST Array according to the manufacturer’s instructions. Each array was washed and stained in the Affymetrix Fluidics Station 450 and scanned to generate a CEL ﬁle using the Affymetrix GeneChip Scanner 3000 7G and Command Console^®^ (AGCC) Software (Affymetrix, Santa Clara, CA). The Affymetrix Expression Console Software (version 1.0) was used to perform quality assessment. The exon array data are deposited in GEO with Series accession number GSE47032.

All exon array data were analyzed using Partek Genomic Suite 6.4 software (Partek). The robust multi-array average (RMA) algorithm was used for gene- and exon-level intensity analyses. Data were ﬁltered to consider only those probe sets included in the “Core Meta-Probeset”. The Analysis of Variance (ANOVA) and multi-test correction for p-values in Partek Genomic Suite were used to identify differentially expressed genes and exons. Tissue type (tumor versus NT) was chosen as the candidate variable in the ANOVA model to obtain tumor-related expression changes and splicing events. ANOVA p-values were corrected using Bonferroni method. Lists of genes and exons with significant variation of the expression levels were generated by using a 0.01 FDR criterion as a significant cutoff.

Partek list of significant exon-level probesets (FDR corrected p-value < 0.01) and an exon level ANOVA test list were used to create - by an ad hoc Python script - a database of significant probesets from Exon Array experiments. To use a more stringent analysis criteria we excluded from the final exon lists all those cases in which Exon Array revealed a significant change at a gene-level. The resulting database was employed to explore simple exon skipping events using as reference ASPicDB single exon skipping splicing events after mapping Affymetrix probesets on ASPicDB .gtf files. A single exon from the Partek list was defined significantly skipped only if its adjacent exons were not, by using 0.01 and 0.05 p-value criterions as a significant cutoff for the “skipped” and adjacent exons, respectively. Finally, information about the portion (CDS or UTRs) affected by the splicing event has been added, starting from .gff files generated from ASPicDB.

### Pathway analyses

Ingenuity Pathway Analysis (IPA) software (Ingenuity Systems Inc.) was used to perform a global characterization of the over-represented biological functions within the two sets of up-regulated and down-regulated genes in ccRCC. Each data set was screened for the presence of mapped and function eligible genes. The over-represented biological functions were ranked according to the calculated p-values. The enrichment of specific biological processes and pathways among differentially expressed genes was analyzed also using the Database for Annotation, Visualization and Integrated Discovery (DAVID, http://david.abcc.ncifcrf.gov/) bioinformatics resource [21,22].

### qRT-PCR experiments and data analysis

Reverse transcription of 500 ng of total RNA was performed using QuantiTect^®^ Reverse Transcription kit (Qiagen^®^) and diluted cDNA (1:3) was used in all following qPCR reactions. Control reverse transcription reactions without RT enzyme were also prepared and controlled by PCR with the same primers and amplification conditions applied for qRT-PCR assays (data not shown).

The geNorm VBA applet for Microsoft Excel [23] was used to determine the most stable housekeeping genes for the samples used in this study, selected from a panel of five reference genes (ACTB, B2M, GAPDH, HPRT1 and RPL13) in 8 of the 18 sample pairs used in qRT-PCR experiments (PS_21, PS_43, PS_44, PS_45, PS_46, PS_47, PS_62 and PS_67). For the geNorm analysis, qRT-PCR experiments were performed in duplicate on the ABI PRISM 7900HT platform (Applied Biosystems^®^, Life Technologies^TM^) using 1μl of diluted cDNA as template for each reaction with TaqMan^®^ Universal PCR Master Mix (Applied Biosystems^®^, Life Technologies^TM^). TaqMan assays from Applied Biosystems^®^ were used for the amplification of ACTB (Hs99999903_m1), B2M (Hs99999907_m1), GADPH (Hs99999905_m1), HPRT1 (Hs03929098_m1) and RPL13 (Hs00761672_s1) transcripts. No template controls were included as negative controls for each TaqMan assay. Amplification parameters were as follows: hot start at 95°C for 10 min; 50 amplification cycles (94°C for 15 sec, 60°C for 1 min). Results were first analyzed in SDS 2.4 software and then exported in Microsoft Excel in order to be further analyzed by using geNorm which identified ACTB and RPL13 as the best housekeeping genes for these samples.

qRT-PCR primers for reference genes and differentially expressed genes and exons (including their adjacent exons) were designed using Primer3 software [24], and their specificity was checked with both the UCSC “In-Silico PCR” and the NCBI Primer-BLAST tools. Sequences of primers used for qRT-PCR are reported in Table S1. For gene expression analysis qRT-PCR experiments were carried out in duplicate using the ABI PRISM 7900HT platform (Applied Biosystems^®^, Life Technologies^TM^) using 1μl of diluted cDNA as template for each reaction with QuantiTect^®^ SYBR Green PCR Master Mix (Qiagen^®^). No template controls were included as negative controls for each primer pair. Amplification parameters were as follows: hot start at 95°C for 15 min; 50 amplification cycles (94°C for 15 sec, 62°C for 30 sec, 72°C for 30 sec); dissociation curve step (95° C for 15 sec, 60°C for 15 sec, 95°C for 15 sec). Fluorescence raw data were exported from the SDS 2.4 software (Applied Biosystems^®^, Life Technologies^TM^) and analyzed with the DART-PCR Excel workbook [25]. Actual amplification efficiency values (E) for each amplicon were used to correct Cq values before analyzing these data by the ΔCq method to compare relative expression results. Expression levels were calculated relative to the mean expression levels of ACTB and RPL13 genes, according to the following formula: relative Expression Ratio (rER) = 2^{(Cq_gene/exonX_ – [(Cq_ACTB_ + Cq_RPL13_)/2]}. For exon-level analysis a percentage of inclusion was also calculated as the average of the expression ratio of skipped exon relative to both upstream exon and the downstream one, as well as the expression level fold change for the three exons considering each tissue pair. 

### Confocal laser scanning microscopy

Paraffin-embedded kidney samples were stained for TCF21, LAMA4, KCNJ1 and PTP4A3 (Abcam, Cambridge, UK). Renal biopsies were deparaffinized and underwent epitope unmasking through three microwave cycles at 750W for 5 min in citrate buffer (pH = 6). The slides were incubated with the appropriate blocking solution, primary antibodies (anti-TCF21 1:200, anti-LAMA4 1:500, anti-KCNJ1 1:50, anti-PTP4A3 1:200) and secondary antibodies (Alexa Flour 488 goat anti-rabbit, Molecular Probes, Eugene, OR). All sections were counterstained with TO-PRO-3 (Molecular Probes). Negative controls were prepared with irrelevant antibody. Specific fluorescence was acquired using the confocal microscope Leica TCS SP2 (Leica, Wetzlar, Germany). Fluorescence levels were quantified using Adobe Photoshop software and expressed as area fraction (%).

### Statistical analysis

Two-tailed Student's T tests were performed to assess the statistical significance of gene and protein expression levels differences observed between the tumoral and NT ccRCC samples (and also among the different grades of ccRCC). A p-value < 0.05 was considered statistically significant. Statistical analyses for gene expression data were performed by Analysis ToolPak in Microsoft Excel 2010 software, while Statview software package (version 5.0, SAS Inc. Cary, NC) was used for protein statistical analyses.

## Results

### Differential expression profiling by exon array

Tissue specimens were obtained from 20 patients undergoing radical or partial nephrectomy for unilateral RCC. Table 1 summarizes the main clinical features of the patients included in the study. The morphological features of the tumor tissues corresponded to well-differentiated renal cell carcinomas of the clear cell type, showing prominent cytoplasmic clearing and thin-walled vascular channels. The expression of ~17,800 core transcript clusters were analyzed in the renal tissues of 10 ccRCC tumoral (T) and 10 matched NT samples. A preliminary ANOVA highlighted the “Status” variable (i.e., NT and ccRCC tissues) as the major source of variance, with all other variables considered accounting for the residual variance, with small differences between random variables (i.e., “Patient”) and factors (i.e., “Fuhrman Grade” and “Gender”) (data not shown). “Status” variable was therefore used as a grouping factor in the principal component analysis (PCA) resulting in a clear segregation of NT tissue samples from the neoplastic ones (data not shown).

The gene-level analysis was performed using an ANOVA with all the information about the samples analyzed, with the “Status” variable (discriminating between NT and ccRCC samples), as parameter for comparison. All those transcripts with no significant change in probesets expression (FDR corrected p-value higher or equal to 0.01) or in linear fold change (-1.4 < FC < 1.4 for ccRCC vs NT) were excluded from the final gene list. Only genes passing these filters were retained to improve the statistical relevance of our results.

A list was produced, containing 1,312 up-regulated and 1,150 down-regulated genes in ccRCC, for a total of 2,462 differentially expressed genes (corresponding to 2,468 differentially expressed transcript clusters: 1,316 were up-regulated and 1,152 down-regulated) (Tables S2-S3). Even if the number of genes up-regulated and down-regulated in ccRCC samples was quite similar, down-regulated genes showed a wider range of variation, as their fold changes ranged from -1.4 to -232.9. On the contrary, the fold changes of up-regulated genes ranged from 1.4 to 37.9. Among up-regulated genes, some known transcriptional targets of HIF were found, i.e., CXCR4, TGFB1 and HK2 genes [4].

The exon-level analysis was performed as described above for the gene-level analysis. Probesets with no significant change in expression (FDR corrected p-value higher or equal to 0.01) related to the status parameter were excluded from the final exon list. A more robust analysis was obtained comparing this list with a list of Affymetrix Exon Array probesets corresponding to single exon skipping events collected in ASPicDB [17,18]. In this way, we obtained a list of potentially differentially skipped exons that was further refined using the criteria that upstream and downstream exons should not be significantly differentially expressed (see Materials and Methods section for a more detailed description).

Therefore, a list containing 139 tumor-associated exon inclusion events (where an inclusion event is to be intended as the entire exon triplet involved in the skip event) in 118 genes and 132 normal-associated exon inclusion events in 119 genes (Tables S4-S5) was generated.

### Functional Enrichment Analysis

Ingenuity Pathway Analysis (IPA) software was used to perform a global characterization of over-represented biological functions within the two sets of up-regulated and down-regulated genes in ccRCC. 

Using the Ingenuity Knowledge Database, differentially expressed genes were functionally characterized: 1266/1312 (96%) and 1121/1150 (97%) within the up- and down-regulated gene set, respectively. 

The IPA “Core Analysis” revealed clear differences between biological functions over-represented in the two data sets. Briefly, as shown in Table 2, the set of genes up-regulated in ccRCC is functionally mostly associated with inflammatory response (“Cell-To-Cell Signaling and Interaction”, “Inflammatory Response”, “Infectious Disease”, “Immune Cell Trafficking” and “Lymphoid Tissue Structure and Development”) and cancer (“Cellular Growth and Proliferation”, “Tumor Morphology”, “Cancer”, “Cellular Movement” and “Cell Death”). In particular, as shown in Figure S1, a marked alteration of the immune response seems to be associated with ccRCC. Besides an obvious over-representation of genes associated with cancer (“DNA Replication, Recombination and Repair” and “Cancer”), the down-regulated data set seems to be mostly associated with renal disorders (“Renal and Urological Disease” and “Molecular Transport”) (Table 2 and Figure S2). These data, together with the alteration of several metabolic pathways (“Aminoacid Metabolism”, “Lipid Metabolism”, “Small Molecule Biochemistry” and “Carbohydrate Metabolism”) may suggest a global alteration of the renal function.

**Table 2:** IPA Analysis.

**Top Networks up-regulated genes**				**Top Networks down-regulated genes**			
**Associated Network Functions**			**Score**	**Associated Network Functions**			**Score**
Cell-to-Cell Signaling and Interaction, Inflammatory Response, Infectious Disease			42	Decreased Levels of Albumin, DNA Replication, Recombination, and Repair, Gene Expression			40
Cellular Growth and Proliferation, Tumor Morphology, Developmental Disorder			40	Genetic Disorder, Metabolic Disease, Renal and Urological Disease			38
Antimicrobial Response, Inflammatory Response, Dermatological Diseases and Conditions			38	Molecular Transport, Neurological Disease, Tissue Development			36
Cell Morphology, Cellular Compromise, DNA Replication, Recombination, and Repair			38	Energy Production, Lipid Metabolism, Small Molecule Biochemistry			36
Lipid Metabolism, Small Molecule Biochemistry, Nucleic Acid Metabolism			38	Carbohydrate Metabolism, Energy Production, Small Molecule Biochemistry			34
**Top Bio Functions up-regulated genes**				**Top Bio Functions down-regulated genes**			
**Diseases and Disorders**	**p-value**		**# molecules**	**Diseases and Disorders**	**p-value**		**# molecules**
Inflammatory Response	5.41E-84 - 1.29E-09		328	Developmental Disorder	4.33E-18 - 8.46E-03		101
Cancer	1.57E-61 - 8.10E-10		512	Genetic Disorder	4.33E-18 - 8.46E-03		538
Hematological Disease	3.78E-36 - 1.77E-10		176	Metabolic Disease	4.33E-18 - 8.46E-03		278
Immunological Disease	3.02E-34 - 2.75E-10		387	Cancer	3.62E-14 - 8.46E-03		314
Gastrointestinal Disease	1.33E-30 - 1.22E-09		423	Renal and Urological Disease	3.84E-12 - 7.20E-03		95
**Molecular and Cellular Functions**	**p-value**		**# molecules**	**Molecular and Cellular Functions**	**p-value**		**# molecules**
Cellular Growth and Proliferation	2.40E-66 - 3.77E-10		492	Molecular Transport	6.13E-18 - 8.46E-03		201
Cellular Movement	1.05E-61 - 1.29E-09		316	Amino Acid Metabolism	4.78E-15 - 8.46E-03		60
Cell-to-Cell Signaling and Interaction	3.94E-55 - 9.97E-10		291	Small Molecule Biochemistry	4.78E-15 - 8.46E-03		258
Cellular Development	4.94E-45 - 1.14E-09		350	Lipid Metabolism	9.69E-11 - 8.46E-03		154
Cell Death	2.06E-42 - 1.20E-09		350	Nucleic Acid Metabolism	2.38E-08 - 5.73E-03		68
**Physiological System Development and Function**	**p-value**		**# molecules**	**Physiological System Development and Function**	**p-value**		**# molecules**
Hematological System Development and Function	1.78E-58 - 1.29E-09		353	Tissue Development	1.48E-05 - 8.46E-03		196
Immune Cell Trafficking	4.34E-57 - 1.29E-09		250	Skeletal and Muscular System Development and Function	2.00E-04 - 8.46E-03		67
Hematopoiesis	4.59E-46 - 6.80E-10		195	Visual System Development and Function	2.61E-04 - 8.46E-03		26
Tissue Morphology	1.02E-44 - 4.29E-10		204	Embryonic Development	2.83E-04 - 8.46E-03		137
Lymphoid Tissue Structure and Development	7.47E-41 - 3.63E-10		155	Organ Development	2.83E-04 - 8.46E-03		126

Top 5 networks and biological functions over-represented in the up-regulated (on the left) and down-regulated (on the right) data sets.

The top 5 networks and the top 5 biological functions that were over-represented in the data sets along with the number of genes assigned to each category are shown in Table 2. Moreover, the top functions and the top canonical pathways over-represented in the two data sets are shown in the histograms of Figures S1-S2.

Functional annotation was confirmed by DAVID tool, used to identify the most relevant biological processes and pathways significantly enriched in the up-regulated and down-regulated gene lists (Tables S6-S9).

### Gene expression levels of PTP4A3, CAV2, LAMA4, KCNJ1, SFRP1 and TCF21 in ccRCC

To confirm differentially expressed genes detected by the exon array analysis, qRT-PCR validations were performed on an extended set of specimens (18 paired tumor and NT samples) including the already analyzed eight out of ten pairs (see Table 1). Six genes (3 up-regulated and 3 down-regulated in ccRCC vs. NT) were selected from the list of differentially expressed genes depending on their fold change value, their low variability in expression levels among samples and their function somehow correlated with cancer and/or kidney. 

**Figure 1:**
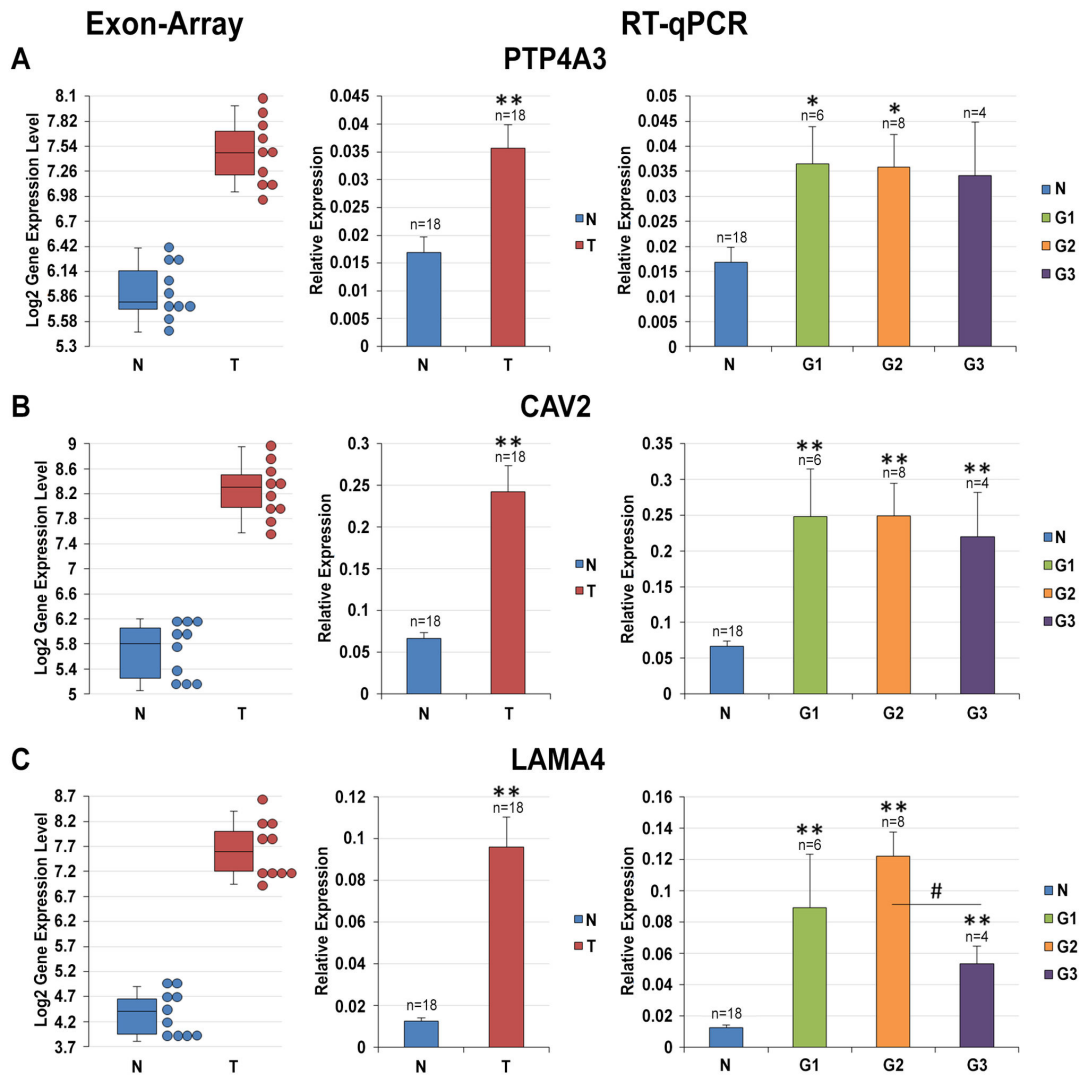
Gene Expression analysis of PTP4A3, CAV2 and LAMA4 genes. Expression levels of PTP4A3 (**A**), CAV2 (**B**) and LAMA4 (**C**) genes were calculated relative to the mean expression levels of ACTB and RPL13 genes. From left to right: box plot of log_2_ gene expression intensities resulting from microarray analysis (each dot stands for a sample); histogram of the expression levels (mean ± SE) of target genes in NT and ccRCC samples; histogram of the expression levels (mean ± SE) of target genes in NT and G1, G2 and G3 ccRCC samples. * = p-value < 0.05; # = p-value < 0.01; ** = p-value < 0.001.

**Figure 2:**
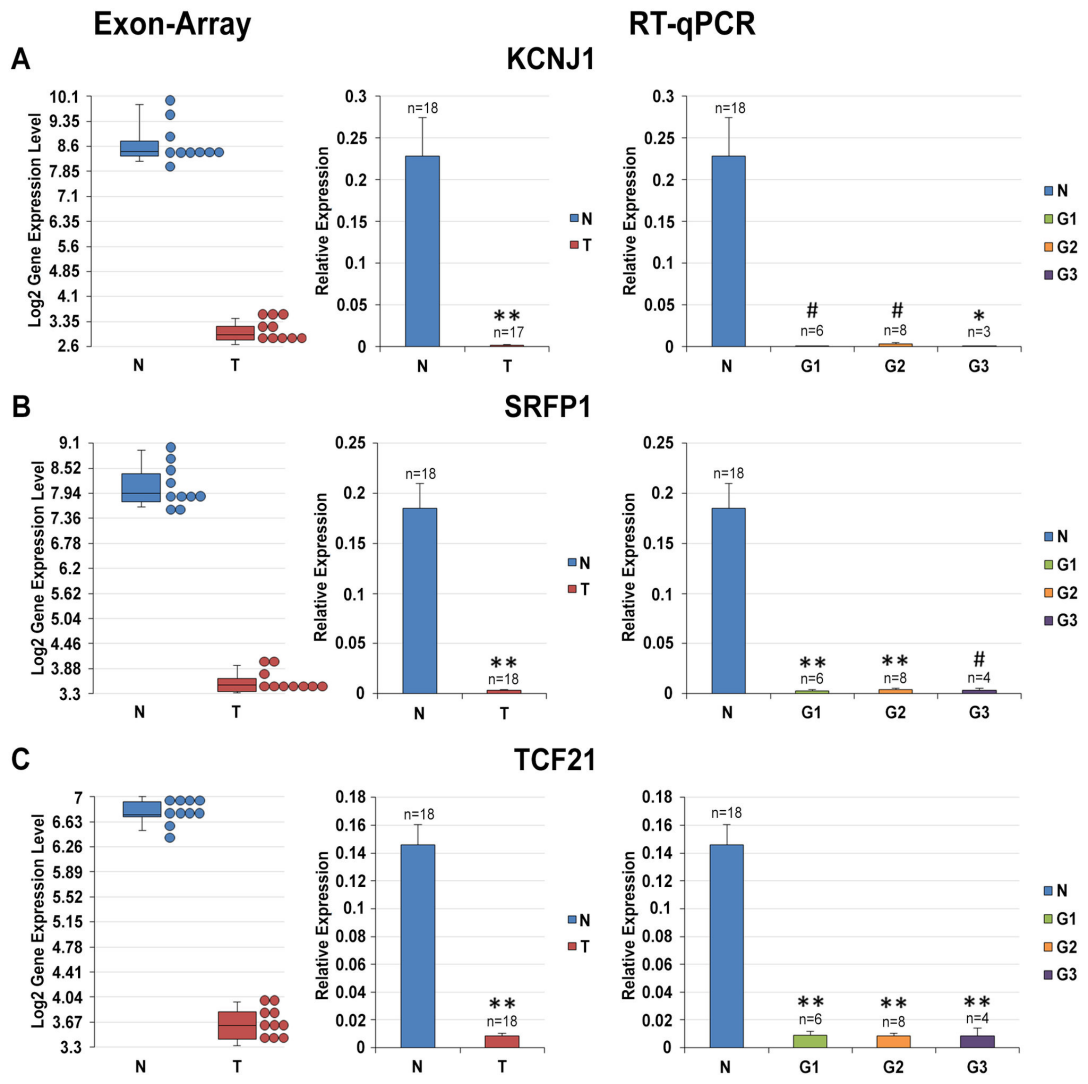
Gene Expression analysis of KCNJ1, SFRP1 and TCF21 genes. Expression levels of KCNJ1 (**A**), SFRP1 (**B**) and TCF21 (**C**) genes were calculated relative to the mean expression levels of ACTB and RPL13 genes. From left to right: box plot of log_2_ gene expression intensities resulting from microarray analysis (each dot stands for a sample); histogram of the expression levels (mean ± SE) of target genes in NT and ccRCC samples; histogram of the expression levels (mean ± SE) of target genes in NT and G1, G2 and G3 ccRCC samples. * = p-value < 0.05; # = p-value < 0.01; ** = p-value < 0.001.

The expression levels of these three up-regulated (PTP4A3, CAV2 and LAMA4) and three down-regulated genes (KCNJ1, SFRP1 and TCF21) evaluated by qRT-PCR were in line with those obtained by the expression array (in this case also considering the Fuhrman grade of the ccRCC; Figures 1 and 2). Table 3 summarizes the main functions of the genes analyzed, and Table S10 shows the IPA enriched networks and bio-functions these genes are associated to.

**Table 3:** Main functions of the genes further analyzed by qRT-PCR and confocal laser scanning microscopy.

Gene Name	Status (ccRCC vs NT)	Functions
**PTP4A3**	up-regulated	It encodes a tyrosine phosphatase involved in reconstructing of the cytoskeleton, regulating adhesion and cell cycle of the cancer cells, and in epithelial-mesenchymal transition [26].
**CAV2**	up-regulated	As one of the principal structural proteins of caveolae, it is involved in vescicular trafficking and signal transduction, so playing a role in many essential cellular functions (i.e., signal transduction, lipid metabolism, regulation of cell growth and apoptosis) [27].
**LAMA4**	up-regulated	This gene encodes a glycoprotein of the extracellular matrix involved in various biological processes, including cell adhesion, differentiation, migration, signaling, and metastasis.
**KCNJ1**	down-regulated	This integral membrane protein constitutes inward-rectifier type potassium channels, also known as ROMK, that are predominantly found in the kidney.
**SFRP1**	down-regulated	It encodes a biphasic modulator of Wnt signaling, counteracting Wnt-induced effects at high concentrations and promoting them at lower concentrations [31].
**TCF21**	down-regulated	a transcription factor known to be essential for differentiation of epithelial cells adjacent to mesenchyme [32].

Among the over expressed genes identified by exon arrays in ccRCC tissue samples, we focused our attention on three genes. PTP4A3 has been found have an enhanced expression in several cancers and its expression level increases significantly with tumor progression and severity (e.g., colorectal, gastric, ovarian and breast carcinomas) [26]; CAV2 was also selected due to the recently demonstrated significant association between its expression level and breast cancer basal-like and triple-negative immunophenotype, and because it was found to have a prognostic impact on breast cancer-specific survival [27]; the gene and protein expression of LAMA4 was found to correlate with tumor invasion and metastasis in hepatocellular carcinoma and in angiotropic melanoma areas [28,29]. As shown in Figure 1A-C, gene expression alterations measured by exon array (fold change = 3.004, p-value = 1.46E-08 for PTP4A3; fold change = 5.998, p-value = 5.97E-10 for CAV2; fold change = 9.751, p-value = 5.72E-11 for LAMA4) were confirmed by RT-qPCR validation. Indeed, even if slightly smaller fold changes were observed in the wider set of samples by qRT-PCR, the up-regulation of these genes still maintained a statistical significance. Of note, we detected 2.12 fold increase of their expression levels in tumoral samples compared to NT samples (p-value = 0.00094) for PTP4A3 (Figure 1A), 3.64 for CAV2 (p-value = 4.05E-06) (Figure 1B), and 7.72 (p-value = 1.63E-06) for LAMA4 (Figure 1C). Intriguingly, these gene expression alterations were confirmed in all but one sample pair for LAMA4 gene, while in 14 and 15 out of 18 samples for PTP4A3 and CAV2, respectively (data not shown). Although we detected some inter-individual variability, we noticed that the expression level of CAV2 gene was quite constant among samples in the NT condition and only in few cases were CAV2 mRNA levels higher than in non-paired tumor samples (data not shown). As far as the tumor grade was concerned, a significant alteration among the different histological grades was observed only for LAMA4, with a remarkable decrease of its expression levels passing from G2 to G3 ccRCC (Figure 1C).

Among the down-regulated genes identified by exon arrays in ccRCC tissue samples we selected the following genes: KCNJ1, SFRP1 and TCF21. This selection was mainly in view of the pivotal role of these genes in renal function (KCNJ1), cancer (SFRP1) and cell differentiation (TCF21). KCNJ1 is a kidney specific channel affecting not only the transport of potassium ions but indirectly also the normal balance of sodium and other ions in the body [30]. SFRP1 is a tumor suppressor, which has already been associated not only to carcinogenesis but also, to some extent, with the metastatic potential of cancer cells [31]. TCF21 is a transcription factor demonstrated in lung and head and neck cancers to be inactivated by epigenetic mechanisms [32]. Moreover, in exon array analysis these three genes showed a huge alteration of their expression levels in terms of down-regulation: we detected a fold change for KCNJ1 of -53.69 (p-value = 1.35E-15), for SFRP1 of -24.5 (p-value = 4.56E-15), and for TCF21 of -8.741 (p-value = 6.42E-17) as shown in Figure 2A-C. Increasing the number of the samples analyzed, these deregulations were fully validated in qRT-PCR assays. Indeed, comparing ccRCC samples to NT tissue samples, KCNJ1 showed a decrease of its expression levels to -133.8 (p-value = 2.94E-05) (Figure 2A), SFRP1 resulted down-regulated with a fold change of -55.1 (p-value = 1.74E-08) (Figure 2B), while TCF21 expression dropped -16.8 times (p-value = 6.70E-11) (Figure 2C). These genes proved to be very promising candidate biomarkers as their overall expression level was significantly different in NT and ccRCC conditions in all subjects investigated. No significant differences were found between different Fuhrman tumor grades.

### Protein expression levels of LAMA4, KCNJ1, TCF21 and PTP4A3 in ccRCC

**Figure 3:**
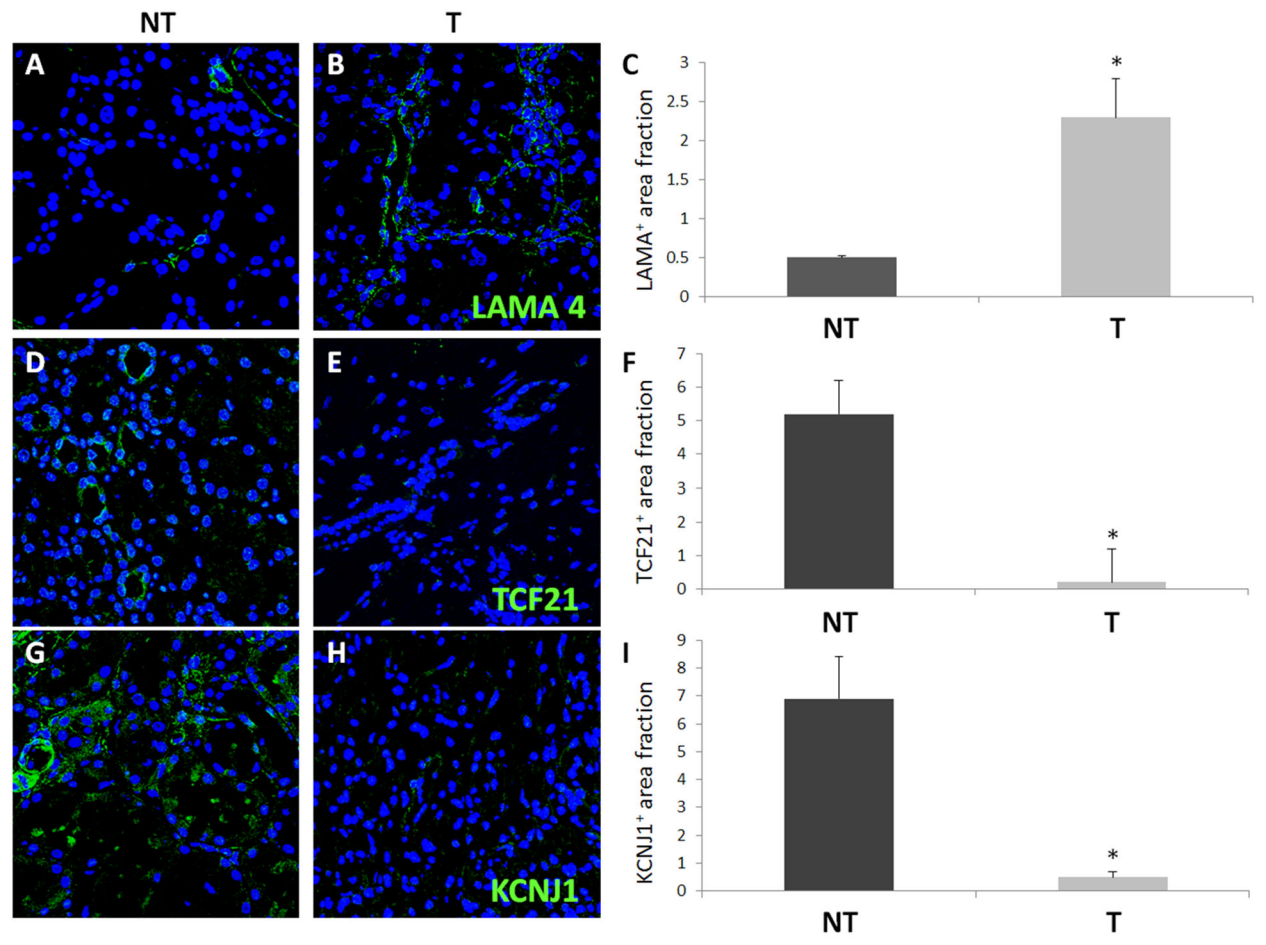
*In* *situ* analysis for LAMA4^+^, TCF21^+^ and KCNJ1^+^ using confocal laser scanning microscopy. RCC tumor tissues (T) were characterized by a significant increase of LAMA4 expression (**B**) and decrease of TCF21 (**E**) and KCNJ1 (**H**) expression as compared to non-tumoral (NT) kidney portion (**A**, **D**, **G**). Nuclei are highlighted with TO-PRO in blue, Quantification of specific protein expression was obtained as described in the Methods section, (**C**, **F**, **I**). Results are expressed as mean ± S.D. For each group, all images (magnification 63X) are from a single patient and are representative of the whole group of patients.

To further support the gene expression data we measured the corresponding intracellular protein levels of LAMA4, KCNJ1, TCF21 and PTP4A3 in ccRCC renal biopsies (n=6) compared with the corresponding NT kidney tissue, by means of immunofluorescence technique. We found that LAMA4+ cells were barely detectable in NT kidney and their expression was limited to the small and medium renal artery walls (Figure 3A), while we observed a significant increase (p < 0.005) of LAMA4 in ccRCC tumor tissue, with a distribution similar to the peritubular capillary network (Figure 3B). On the contrary, we detected a significant decrease of TCF21 (Figure 3D and E) and mostly KCNJ1 expression (Figure 3G and H) in RCC tissue when compared with normal kidney tissue (p < 0.005 and p < 0.0001, respectively). Moreover, the number of infiltrating PTP4A3^+^ cells was higher in the tumor compared with normal counterpart, although the difference did not reach statistical significance (data not shown).

### Differential alternative splicing in LIMK2, DAB2, PTPRF and FDXR transcripts

To validate our exon-level approach, we selected four alternative splicing events among the more statistically significant events resulting from the microarray data analysis. Technical issues related to the primer design for qRT-PCR were also considered in the selection process. In particular, we focused our attention on four cassette exons (CEs) that showed higher inclusion ratio in tumor tissues in order to evaluate their usage as tumor tissue biomarkers. 

**Figure 4:**
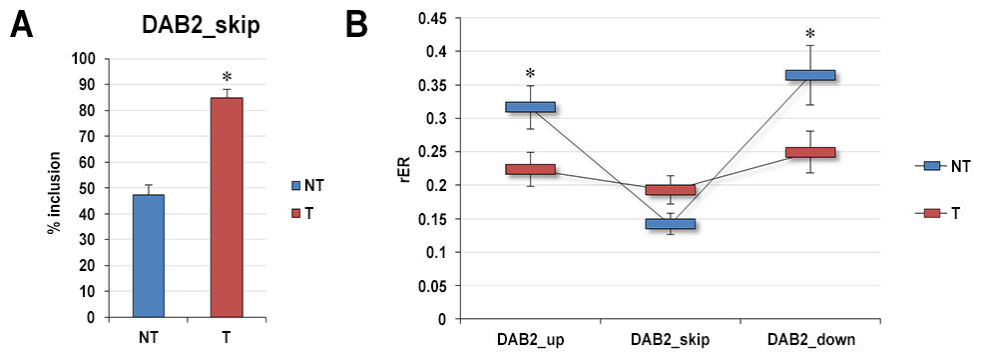
qRT-PCR analysis of differentially alternative splicing exons identified in DAB2 gene. Left panel (**A**) shows the percentage of inclusion of the skipped exon in tumoral (T) and non-tumoral (NT) tissues, calculated as the average of the expression ratio of skipped exon relative to both upstream exon and the downstream one. Right panel (**B**) shows the relative expression ratio (mean ± SE) of each exon of the triplet involved in the splicing event both in NT and T samples. Expression levels were calculated relative to the mean expression levels of ACTB and RPL13 genes. * = p-value < 0.001.

Only one of the four CEs analyzed resulted differentially included in ccRCC samples. This alternative splicing event occurs in DAB2 transcripts and gives rise to two known alternative spliced forms, p96 and p67, the latter lacking the CE was evaluated in our analysis. DAB2 gene encodes an adaptor molecule involved in multiple receptor-mediated signaling pathways that plays a pivotal role in cellular homeostasis. Moreover, DAB2 plays an important regulatory role in cellular differentiation and acts as a tumor suppressor, at least in part, by stabilizing the beta-catenin degradation complex, thus negatively regulating the Wnt signaling pathway [33]. DAB2 splice forms are expressed in a tissue-specific pattern suggesting that they possess non-overlapping cellular activities. In particular, in renal tissue, p96 is equally or more abundant than p67 [34]. Our analysis confirm this data as we measured a percentage of about 50% of inclusion of the CE in normal kidney tissues (Figure 4A). Interestingly, we observed a differential inclusion of the CE in NT and tumoral tissues. Indeed, as shown in Figure 4A, in ccRCC samples there was a significant increase (p-value = 4.48E-06) of the ratio of the isoforms (i.e., p96 splice variant) including the cassette exon analyzed, and only 15% of mature DAB2 transcripts resulted from the skipping of this exon. This scenario is clearly shown also in Figure 4B, and considering the single condition, it is evident that while no differences in the expression level of the three exons involved in the splicing event were detected for the tumor tissues, in their normal counterparts the skipped exon was two-fold less expressed than its adjacent exons (p-value = 3.25E-05 for the upstream exon and p-value = 4.2E-05 for the downstream exon).

A decreased expression of DAB2 has been demonstrated in several cancers including ovarian, breast, prostate, oesophagus, urinary bladder, colon and choriocarcinoma [33]. On the contrary, neither exon array nor qRT-PCR data supported a down-regulation at gene level for DAB2 in ccRCC. 

We were unable to validate a differential expression for the other three cassette exons (in LIMK2, PTPRF and FDXR genes). Even if a clear exon skipping event was detected in PTPRF and FDXR transcripts, in none of the three cases did our qRT-PCR experiments show any significant variation in the inclusion ratio of the CEs (Figure S3). 

## Discussion

In this work, we investigated the differential expression of the coding transcriptome in ccRCC compared to the matched NT renal epithelium counterpart. We used the exon array platform (Affymetrix) that explores the expression of all exons of the human genome, both annotated and putative. We limited the present data analysis to the subset of well-annotated probesets in order to obtain a more reliable characterization of expression alterations in ccRCC samples. We extended the analysis to the exon-level exploiting the full potential of this gene expression microarray platform using the Partek Genomic Suite workflow implemented with an ad hoc analysis pipeline allowing to evaluate, for each exon, the expression behavior of the adjacent upstream and downstream exons [17,18,35].

Our analysis showed that over 2,000 genes resulted differentially expressed in ccRCC, equally distributed between up-regulated and down-regulated genes. Nevertheless, for these two groups of genes we observed differences in their fold changes, with down-regulated genes showing a wider range of variation compared to the up-regulated ones, and most importantly, different biological functions. Indeed, while up-regulated genes seem to be mainly involved in cellular growth, proliferation and movement, down-regulated genes are mostly associated with renal processes and metabolic pathways, suggesting somehow a loss of the main functions of renal cells for the loss of cell differentiation, a hallmark of tumorigenesis. 

Next, we focused our attention on six differentially expressed genes, not previously associated to ccRCC, analyzing their expression levels on a larger cohort of samples. These genes (PTP4A3, CAV2, LAMA4, KCNJ1, SFRP1 and TCF21) deserved further analysis because of their known involvement in the regulation of proliferative processes and potential participation to carcinogenesis, besides to their kidney-linked functions. Intriguingly, besides their main biological functions, for PTP4A3, CAV2 and LAMA4 genes evidences were found for their involvement in immune response and inflammation, that represented the most altered networks for up-regulated genes in our study. Indeed, the phosphatase PTP4A3 regulates negatively the activity of p38α MAPK normally involved in the regulation of the transcription of several inflammatory cytokines including TNF-α [36]; CAV2 contributes to the constitution and regulation of signaling platforms, i.e., the caveolae, so regulating inflammatory response through several mechanisms (e.g., NO production, regulation of Cox-2 degradation and Ca^2+^ regulation and signaling) [37]; LAMA4, is involved in leukocyte recruitment to inflammatory loci supporting T-cell migration through the vessel wall [38]. However, during our study, evidence was provided for ccRCC association for two of these six genes, namely SFRP1 and TCF21 [39,40]. Remarkably, with the exception of potassium channel KCNJ1, all the other genes have already been implicated, in some way or another, in several types of cancer, as described in the Results section. For instance, it is known that the stable re-expression of SFRP1 in ccRCC cells results in decreased expression of Wnt target genes, decreased growth in cell culture, inhibition of anchorage-independent growth, and decreased tumor growth, supporting the hypothesis that loss of SFRP1 is an early, aberrant molecular event in renal cell carcinogenesis [39]. TCF21, already identified as a candidate tumor suppressor, epigenetically inactivated in lung and head and neck cancers [32], has been recently included in an innovative panel of biomarkers for a simultaneous detection of urological cancers, providing 36% sensitivity and 100% specificity for kidney cancer detection [41]. TCF21 has been reported down-regulated also in ccRCC, probably because of increased levels of miR-21 [40]. 

This study identifies four novel potential biomarkers for ccRCC (PTP4A3, CAV2, LAMA4 and KCNJ1) and other two genes recently established also by other authors to be implicated in ccRCC (SFRP1 and TCF21) support the reliability of our investigation. As some of these genes (e.g., PTP4A3 and CAV2) have also been included in cancer gene signatures for predicting recurrence and prognosis[26,42], it is worth to postulate the effectiveness of a restricted panel of ccRCC tumor biomarker even if further validations and comparisons with clinical standards are required. 

By expanding our ccRCC transcriptome analysis beyond the gene level we investigated the occurrence of alternatively spliced isoforms, and particularly cassette exons whose presence or absence resulted associated to the tumoral condition. To make a more stringent and accurate analysis we focused only on those differentially skipped exons identified in genes that are not differentially expressed in normal and tumoral conditions, considering not only the exon that was skipped but the entire exon triplet involved in the splicing event. Overall, we identified over 250 exons differentially expressed between the non tumoral and ccRCC conditions. Excluding skipping events located in UTRs, or involving very small exons or those when primer design was not easily feasible, we obtained a very restricted list of candidates, four of which were selected for validation based on both their statistical significance and functional annotation. However, we were able to validate only one of the four selected cases, involving DAB2 gene where the inclusion rate of the cassette exon was 50% in the normal condition and 85% in ccRCC. The limited success rate of qRT-PCR validation can be mostly ascribed to the intrinsic limitations of Exon Arrays as already suggested by other authors [43-45]. This could explain why our results did not overlap with those obtained by Zhao et al. using RNA-Seq data [16]. Interestingly, in our samples DAB2 was equally expressed in the normal and in the tumor condition, differently from what was observed in other tumors where it is significantly down-regulated. Thus, the variation of the relative abundance of DAB2 splice variants (i.e., p96 and 67) could be considered for further analysis to determine the functional relevance of this alteration in renal cancer, as these isoforms have non-overlapping cellular activities.

## Conclusions

In this study, we analyzed the protein coding transcriptome in patients affected by ccRCC in order to identify alterations in gene expression levels and/or alternative splicing patterns between tumoral and the matched non-tumoral renal epithelium. Our analyses indicated that gene-level regulation is somehow affected by the carcinogenesis process for a great number of genes, among which we identified four genes (PTP4A3, CAV2, LAMA4 and KCNJ1) whose expression was previously not known to be altered in ccRCC. Moreover, developing an ad hoc procedure for exon-level analysis, we identified at least one example of cassette exon differentially skipped in ccRCC, i.e., showing differential inclusion rates in DAB2 mature transcripts between tumoral and NT samples. Future studies screening a larger number of ccRCC patients for these expression and alternative splicing alterations (and also others identified so far only by exon array) may help a better characterization of this aggressive carcinoma in order to define ever more accurate and discriminative biomarkers panels to be used at different levels of clinical practice.

## Supporting Information

Table S1 **List of primers used in qRT-PCR experiments.** Sequences of primers used in qRT-PCR experiments for gene- and exon-level analyses are listed along with the size of the amplicon.
(DOCX)

Table S2 **List of up-regulated genes in ccRCC respect to non-tumoral samples as resulted by gene-level Partek analysis of Affymetrix Exon Arrays.** The list contains only genes showing a linear fold change > 1.4 for ccRCC vs NT comparison and FDR corrected p-value less or equal to 0.01. For each gene the Affymetrix transcript ID, the RefSeq ID, the fold-change as well as the p-value for the comparison are reported.
(DOCX)

Table S3 **List of down-regulated genes in ccRCC respect to non-tumoral samples as resulted by gene-level Partek analysis of Affymetrix Exon Arrays.** The list contains only genes showing a linear fold change < -1.4 for ccRCC vs NT comparison and FDR corrected p-value less or equal to 0.01. For each gene the Affymetrix transcript ID, the RefSeq ID, the fold change as well as the p-value for the comparison are reported.
(DOCX)

Table S4 **List of tumor-associated exon inclusion events in ccRCC respect to non-tumoral samples.** For each splicing event the genomic coordinates of the skipped, upstream and downstream exons are reported as well as the linear fold change and p-value for the comparison ccRCC vs NT samples for each probeset mapping on the genomic region involved in the splicing event. The value in the columns “InCDS” and “SkipInCDS” indicates if at least one of the exons involved in the splicing events or the skipped exon, respectively, are in the CDS (value = 1) or in the untranslated regions (value = 0).
(XLSX)

Table S5 **List of normal-associated exon inclusion events in ccRCC respect to non-tumoral samples.** For each splicing event the genomic coordinates of the skipped, upstream and downstream exons are reported as well as the linear fold change and p-value for the comparison ccRCC vs NT samples for each probeset mapping on the genomic region involved in the splicing event. The value in the columns “InCDS” and “SkipInCDS” indicates if at least one of the exons involved in the splicing events or the skipped exon, respectively, are in the CDS (value = 1) or in the untranslated regions (value = 0).
(XLSX)

Table S6 **Gene ontology enrichment analysis of genes up-regulated in ccRCC performed by DAVID.** Annotations were considered significantly over-represented when the p-value of the Fisher's exact test as used by DAVID (EASE Score) was < 0.05 and gene counts belonging to an annotation term was equal or greater than 2.
(DOCX)

Table S7 **KEGG pathway enrichment analysis of genes up-regulated in ccRCC performed by DAVID.** Annotations were considered significantly over-represented when the p-value of the Fisher's exact test as used by DAVID (EASE Score) was < 0.05 and gene counts belonging to an annotation term was equal or greater than 2.
(DOCX)

Table S8 **Gene ontology enrichment analysis of genes down-regulated in ccRCC performed by DAVID.** Annotations were considered significantly over-represented when the p-value of the Fisher's exact test as used by DAVID (EASE Score) was < 0.05 and gene counts belonging to an annotation term was equal or greater than 2.
(DOCX)

Table S9 **KEGG pathway enrichment analysis of genes down-regulated in ccRCC performed by DAVID.** Annotations were considered significantly over-represented when the p-value of the Fisher's exact test as used by DAVID (EASE Score) was < 0.05 and gene counts belonging to an annotation term was equal or greater than 2.
(DOCX)

Table S10 **Biological functions and networks associated to PTP4A3, CAV2, LAMA4, KCNJ1, SFRP1 and TCF21 as resulted by IPA analysis.**
(XLSX)

Figure S1 **IPA analysis of up-regulated genes in ccRCC.** Histograms show the top functions (**A**) and canonical pathways (**B**) enriched in the up-regulated data set, ranked for their significance. In the canonical pathway pane (**B**) the line graph shows the ratio of the number of molecules from the dataset that are in the pathway relative to the total number of molecules in the pathway (y-axis on the right). For both enrichment analyses significance threshold was set at -log(0.05).
(TIF)

Figure S2 **IPA analysis of down-regulated genes in ccRCC.** Histograms show the top functions (**A**) and canonical pathways (**B**) enriched in the down-regulated data set, ranked for their significance. In the canonical pathway pane (**B**) the line graph shows the ratio of the number of molecules from the dataset that are in the pathway relative to the total number of molecules in the pathway (y-axis on the right). For both enrichment analyses significance threshold was set at -log(0.05).
(TIF)

Figure S3 **qRT-PCR analysis of differentially alternative splicing exons identified in LIMK2, PTPRF, and FDXR genes.** Left panels (**A**, **C** and **E**) show the percentage of inclusion of the skipped exon in tumoral (T) and non-tumoral (NT) tissues, calculated as the average of the expression ratio of skipped exon relative to both upstream exon and the downstream one. Right panels (**B**, **D** and **F**) show the relative expression ratio (mean ± SE) of each exon of the triplet involved in the splicing event both in NT and T samples. Expression levels were calculated relative to the mean expression levels of ACTB and RPL13 genes. * = p-value < 0.01; ** = p-value < 0.05.
(TIF)
