# Supplementary material for: Genome-Wide Analysis of Differentially Expressed Genes and Splicing Isoforms in Clear Cell Renal Cell Carcinoma
Source: PLoS One. 2013 Oct 23;8(10):e78452. doi: 10.1371/journal.pone.0078452 (PMC3806822; doi:10.1371/journal.pone.0078452)
Supplement: Table S1 — List of primers used in qRT-PCR experiments. Sequences of primers used in qRT-PCR experiments for gene- and exon-level analyses are listed along with the size of the amplicon. (DOCX) [file pone.0078452.s001.docx]

| Primer Name | Sequence (5'-3') | Amplicon Size | Primer Name | Sequence (5'-3') | Amplicon Size |
| --- | --- | --- | --- | --- | --- |
| PTP4A3 For | GCTTCCTCATCACCCACAACC | 99bp | **KCNJ1 For** | TTTGTAGTTGACGCTGGGAATG | 90bp |
| PTP4A3 Rev | CTTCACACACACGCACCACAG |  | **KCNJ1 Rev** | GTGGAAGAAAGGGCTGTTGTG |  |
| CAV2 For | CTGCCTAATGGTTCTGCCTTC | 79bp | **SFRP1 For** | CCAGCGAGTACGACTACGTGA | 83bp |
| CAV2 Rev | GCTCGTACACAATGGAGCAATG |  | **SFRP1 Rev** | GCACTGAGGTGGCTTGGTGT |  |
| LAMA4 For | GAGGAGGCAGATGAGGCTTAC | 89bp | **TCF21 For** | GCCCACTTGAGGCAGATCCT | 85bp |
| LAMA4 Rev | ACGACAGGAAACAGAGTGCGG |  | **TCF21 Rev** | CCACCATAAAGGGCCACGTC |  |
| LIMK2_up For | CAGGAGCTGAGGGGAGTTGT | 71bp | **PTPRF_up For** | GGACCTGAAGCCTGACACAC | 85bp |
| LIMK2_up Rev | CGGAAATGGGGAGGAGGAG |  | **PTPRF_up Rev** | CTCAATGGTGGGGGTGAAGA |  |
| LIMK2_skip For | CCACATTGCTCCAAGCCAGA | 61bp | **PTPRF_skip For** | GTGAGCATGGGCTCCACCAC | 108bp |
| LIMK2_skip Rev | GCAAGAGCCGTGCCAGGTT |  | **PTPRF_skip Rev** | GCCGTCCACCGCCTCGTAG |  |
| LIMK2_down For | GAAGCTCTACTGCCCCAAGGA | 61bp | **PTPRF_down For** | CGGAAGGTGGAGGTGGAGC | 76bp |
| LIMK2_down Rev | GGAGCACCCATGACAGAACT |  | **PTPRF_down Rev** | GCTGCTTGCTGGGGACAGG |  |
| DAB2_up For | GGTGTTGACCAGATGGATTTGT | 60bp | **FDXR_up For** | CCTGGGTGTACTGAGGCAGAG | 80bp |
| DAB2_up Rev | TGGACTATTTAGGTCAGGAGGT |  | **FDXR_up Rev** | TGGTGAAGGCCACTTGCAGG |  |
| DAB2_skip For | CCCAGCCAGCAGTGAGAACT | 75bp | **FDXR_skip For** | GAGCTTCGGGAGATGATTCAG | 71bp |
| DAB2_skip Rev | TCGGGGAGGATTTGACAGAGA |  | **FDXR_skip Rev** | TGGAGACCCAAGAAATCCACAG |  |
| DAB2_down For | CATCTTTGCTCCTCCCGTCT | 65bp | **FDXR_down For** | GCCTCCGCTTTTTCCGAAGC | 81bp |
| DAB2_down Rev | GGGCTGTAGGTTGTCCTGTG |  | **FDXR_down Rev** | TGACTGCTAGGCGGACACC |  |
| RPL13 For | CCCCACTTCCACAAGGACTG | 81bp | **ACTB For** | ACTCTTCCAGCCTTCCTTCC | 117bp |
| RPL13 Rev | GGCCTTACGTCTGCGGATC |  | **ACTB Rev** | AGCACTGTGTTGGCGTACAG |  |
