# Supplementary material for: Genome-Wide Analysis of Differentially Expressed Genes and Splicing Isoforms in Clear Cell Renal Cell Carcinoma
Source: PLoS One. 2013 Oct 23;8(10):e78452. doi: 10.1371/journal.pone.0078452 (PMC3806822; doi:10.1371/journal.pone.0078452)
Supplement: Table S2 — List of up-regulated genes in ccRCC respect to non-tumoral samples as resulted by gene-level Partek analysis of Affymetrix Exon Arrays. The list contains only genes showing a linear fold change > 1.4 for ccRCC vs NT comparison and FDR corrected p-value less or equal to 0.01. For each gene the Affymetrix transcript ID, the RefSeq ID, the fold-change as well as the p-value for the comparison are reported. (DOCX) [file pone.0078452.s002.docx]

| Transcript ID | Gene Symbol | RefSeq | p-value (Status) | Fold-Change  (ccRCC vs. N) |
| --- | --- | --- | --- | --- |
| 3819474 | ANGPTL4 | NM_139314 | 9.06E-08 | 37.9546 |
| 2700244 | CP | NM_000096 | 1.50E-04 | 28.8001 |
| 3458400 | NDUFA4L2 | NM_020142 | 2.55E-08 | 28.7312 |
| 3022814 | C7orf68 | NM_013332 | 2.27E-07 | 27.9502 |
| 3122489 | ANGPT2 | NM_001147 | 3.15E-10 | 24.7958 |
| 3718902 | CCL18 | NM_002988 | 9.52E-06 | 22.4051 |
| 2510464 | TNFAIP6 | NM_007115 | 3.81E-06 | 21.9431 |
| 3168066 | CA9 | NM_001216 | 3.24E-07 | 21.4069 |
| 2872848 | LOX | NM_002317 | 3.00E-05 | 17.795 |
| 3349858 | NNMT | NM_006169 | 5.92E-08 | 16.6383 |
| 3690154 | NETO2 | NM_018092 | 3.27E-08 | 16.4869 |
| 2955827 | PLA2G7 | NM_001168357 | 1.33E-09 | 15.6397 |
| 3441685 | VWF | NM_000552 | 1.27E-08 | 15.0814 |
| 2773947 | CXCL9 | NM_002416 | 1.50E-06 | 14.5681 |
| 2845921 | SLC6A3 | NM_001044 | 3.09E-05 | 14.1608 |
| 2773972 | CXCL11 | NM_005409 | 6.42E-04 | 13.3455 |
| 2829947 | TGFBI | NM_000358 | 8.15E-06 | 12.9416 |
| 3581221 | AHNAK2 | NM_138420 | 1.30E-06 | 12.7699 |
| 3848039 | C3 | NM_000064 | 4.76E-07 | 11.989 |
| 3839920 | FPR3 | NM_002030 | 1.89E-07 | 11.798 |
| 3560403 | EGLN3 | NM_022073 | 1.25E-05 | 11.6384 |
| 2856995 | ESM1 | NM_007036 | 7.35E-08 | 11.2054 |
| 3403015 | ENO2 | NM_001975 | 6.63E-10 | 10.9645 |
| 3016148 | SERPINE1 | NM_000602 | 1.26E-05 | 10.8335 |
| 2489007 | ACTG2 | NM_001615 | 3.01E-06 | 10.2266 |
| 3049292 | IGFBP3 | NM_001013398 | 8.43E-09 | 10.0895 |
| 2415266 | CYP2J2 | NM_000775 | 5.19E-04 | 9.77793 |
| 2838116 | FABP6 | NM_001130958 | 1.12E-05 | 9.758 |
| 2970086 | LAMA4 | NM_001105206 | 5.72E-11 | 9.75141 |
| 3260586 | SCD | NM_005063 | 6.95E-06 | 9.67101 |
| 3296046 | KCNMA1 | NM_001161352 | 5.44E-06 | 9.62492 |
| 3738629 | SLC16A3 | NM_001042422 | 1.53E-06 | 9.6233 |
| 3127818 | LOXL2 | NM_002318 | 2.40E-06 | 9.40211 |
| 3125571 | MSR1 | NM_002445 | 2.74E-07 | 9.10335 |
| 3214227 | DIRAS2 | NM_017594 | 1.48E-04 | 9.02965 |
| 3642200 | PCSK6 | NM_002570 | 6.27E-06 | 9.02319 |
| 2440943 | FCGR3A | NM_000569 | 1.25E-08 | 8.81151 |
| 2598261 | FN1 | NM_212482 | 6.54E-06 | 8.42899 |
| 2792166 | MARCH1 | NM_001166373 | 1.18E-06 | 8.37039 |
| 2372781 | RGS1 | NM_002922 | 1.67E-06 | 8.34801 |
| 2898986 | SLC17A4 | NM_005495 | 6.18E-04 | 8.17041 |
| 2947077 | HIST1H3I | NM_003533 | 7.64E-07 | 8.16676 |
| 3834176 | TMEM91 | NM_001042595 | 5.24E-05 | 8.06271 |
| 3095223 | IDO1 | NM_002164 | 3.79E-06 | 8.04756 |
| 3759335 | GJC1 | NM_005497 | 2.76E-06 | 7.84832 |
| 2773958 | CXCL10 | NM_001565 | 7.07E-05 | 7.82932 |
| 3010503 | CD36 | NM_001001548 | 6.08E-05 | 7.67493 |
| 2992243 | DNAH11 | NM_003777 | 2.62E-05 | 7.61141 |
| 2404158 | LAPTM5 | NM_006762 | 2.91E-06 | 7.5682 |
| 3753568 | SLFN13 | NM_144682 | 4.04E-10 | 7.56681 |
| 2868904 | ST8SIA4 | NM_005668 | 2.94E-07 | 7.47631 |
| 2818517 | VCAN | NM_004385 | 2.27E-04 | 7.39167 |
| 2984884 | RNASET2 | NM_003730 | 2.59E-05 | 7.16961 |
| 3421511 | LYZ | NM_000239 | 3.78E-05 | 7.16431 |
| 3671202 | CDH13 | NM_001257 | 7.75E-06 | 7.12933 |
| 4011096 | EDA2R | NM_021783 | 3.33E-08 | 6.96544 |
| 3476665 | SCARB1 | NM_005505 | 1.05E-07 | 6.91547 |
| 2863885 | LHFPL2 | NM_005779 | 1.50E-08 | 6.91327 |
| 3456732 | ITGA5 | NM_002205 | 1.08E-06 | 6.70879 |
| 2324873 | C1QC | NM_001114101 | 4.92E-06 | 6.6231 |
| 3422855 | GLIPR1 | NM_006851 | 2.31E-05 | 6.59185 |
| 2858023 | PLK2 | NM_006622 | 5.18E-06 | 6.53101 |
| 3128731 | PNMA2 | NM_007257 | 3.43E-04 | 6.44668 |
| 3982612 | GPR174 | NM_032553 | 1.32E-03 | 6.44649 |
| 2888879 | DOK3 | NM_024872 | 1.48E-05 | 6.30574 |
| 3394264 | MCAM | NM_006500 | 2.84E-06 | 6.29095 |
| 3393744 | CD3D | NM_000732 | 1.78E-04 | 6.23365 |
| 3346548 | BIRC3 | NM_001165 | 1.35E-06 | 6.2236 |
| 2515707 | PDK1 | NM_002610 | 4.46E-08 | 6.22351 |
| 2422035 | GBP5 | NM_052942 | 1.83E-04 | 6.19436 |
| 3750767 | ALDOC | NM_005165 | 1.16E-04 | 6.18592 |
| 2486811 | PLEK | NM_002664 | 4.88E-06 | 6.1412 |
| 3020302 | CAV1 | NM_001172895 | 2.87E-09 | 6.07511 |
| 2351572 | CD53 | NM_000560 | 3.09E-05 | 6.04357 |
| 3721452 | FKBP10 | NM_021939 | 6.63E-06 | 6.0127 |
| 3020273 | CAV2 | NM_001233 | 5.97E-10 | 5.9977 |
| 3046062 | AOAH | NM_001177506 | 2.30E-05 | 5.91862 |
| 3232349 | PFKP | NM_002627 | 1.38E-06 | 5.91794 |
| 3945572 | APOBEC3C | NM_014508 | 1.38E-06 | 5.91651 |
| 2953501 | TREM2 | NM_018965 | 7.43E-10 | 5.91266 |
| 2809793 | GZMK | NM_002104 | 9.89E-04 | 5.86721 |
| 2434575 | CTSS | NM_004079 | 4.42E-07 | 5.8441 |
| 3762198 | COL1A1 | NM_000088 | 1.11E-04 | 5.83435 |
| 2807359 | OSMR | NM_003999 | 8.64E-07 | 5.82958 |
| 2775909 | PLAC8 | NM_016619 | 1.56E-04 | 5.77944 |
| 3300115 | PPP1R3C | NM_005398 | 5.19E-08 | 5.77468 |
| 3448088 | BHLHE41 | NM_030762 | 1.64E-07 | 5.72436 |
| 2692199 | SEMA5B | NM_001031702 | 2.46E-05 | 5.71771 |
| 3014347 | NPTX2 | NM_002523 | 1.62E-04 | 5.69477 |
| 3442941 | C3AR1 | NM_004054 | 1.89E-06 | 5.62441 |
| 3142217 | PAG1 | NM_018440 | 6.09E-07 | 5.61611 |
| 2578028 | CXCR4 | NM_001008540 | 3.08E-05 | 5.57767 |
| 3684486 | IGSF6 | NM_005849 | 3.44E-08 | 5.57688 |
| 2351854 | C1orf162 | NM_174896 | 3.39E-04 | 5.54925 |
| 2895244 | EDN1 | NM_001955 | 2.97E-04 | 5.53804 |
| 3286776 | C10orf10 | NM_007021 | 1.73E-08 | 5.44598 |
| 3140213 | MSC | NM_005098 | 8.37E-06 | 5.42288 |
| 3564210 | PYGL | NM_002863 | 1.28E-05 | 5.41302 |
| 3742627 | C17orf87 | NM_207103 | 5.77E-06 | 5.38696 |
| 3590239 | DLL4 | NM_019074 | 6.05E-06 | 5.3735 |
| 2363689 | FCGR2A | NM_001136219 | 1.50E-05 | 5.36989 |
| 2636125 | CD200 | NM_001004196 | 8.81E-05 | 5.3592 |
| 2886595 | LCP2 | NM_005565 | 3.57E-06 | 5.3479 |
| 2845973 | LPCAT1 | NM_024830 | 4.25E-06 | 5.32527 |
| 4006504 | CXorf36 | NM_176819 | 2.38E-05 | 5.32411 |
| 3320123 | ADM | NM_001124 | 5.82E-10 | 5.2943 |
| 3705491 | FAM57A | NM_024792 | 1.11E-06 | 5.25755 |
| 2324856 | C1QA | NM_015991 | 8.77E-06 | 5.24354 |
| 3396770 | CDON | NM_016952 | 2.69E-04 | 5.2412 |
| 3512874 | LCP1 | NM_002298 | 5.71E-05 | 5.19351 |
| 2950214 | TAP1 | NM_000593 | 2.62E-06 | 5.17803 |
| 2900974 | HLA-F | NM_001098479 | 3.70E-06 | 5.17133 |
| 3969081 | TLR7 | NM_016562 | 2.09E-06 | 5.1652 |
| 3870824 | LAIR1 | NM_002287 | 1.95E-06 | 5.13964 |
| 2887490 | STC2 | NM_003714 | 8.17E-05 | 5.10614 |
| 3416577 | NCKAP1L | NM_005337 | 2.07E-06 | 5.09731 |
| 3332298 | MS4A4A | NM_024021 | 1.10E-03 | 5.0782 |
| 2362746 | SLAMF8 | NM_020125 | 1.35E-05 | 5.02838 |
| 3259253 | ENTPD1 | NM_001776 | 8.06E-06 | 5.01094 |
| 3572209 | PGF | NM_002632 | 2.57E-04 | 5.00225 |
| 4011844 | IL2RG | NM_000206 | 4.43E-04 | 4.99957 |
| 2373842 | PTPRC | NM_002838 | 9.10E-06 | 4.92174 |
| 2900051 | HIST1H3H | NM_003536 | 1.54E-05 | 4.92044 |
| 3442706 | CD163 | NM_004244 | 3.17E-04 | 4.89414 |
| 2754937 | TLR3 | NM_003265 | 6.18E-05 | 4.89029 |
| 2919669 | PRDM1 | NM_001198 | 6.12E-07 | 4.87059 |
| 3838385 | CD37 | NM_001774 | 1.95E-05 | 4.85334 |
| 3733275 | KCNJ2 | NM_000891 | 1.68E-05 | 4.84544 |
| 2441386 | RGS5 | NM_003617 | 8.09E-04 | 4.78443 |
| 3755510 | PLXDC1 | NM_020405 | 1.53E-06 | 4.77935 |
| 2903285 | PSMB9 | NM_002800 | 1.32E-06 | 4.73345 |
| 2363562 | FCER1G | NM_004106 | 6.64E-06 | 4.70398 |
| 2440295 | CD84 | NM_003874 | 3.55E-08 | 4.69847 |
| 2489545 | HK2 | NM_000189 | 1.35E-05 | 4.68461 |
| 3973839 | CYBB | NM_000397 | 7.03E-05 | 4.67828 |
| 3860137 | TYROBP | NM_003332 | 2.93E-06 | 4.66648 |
| 3373845 | SLC43A3 | NM_017611 | 1.29E-05 | 4.66162 |
| 2900059 | HIST1H2BM | NM_003521 | 1.84E-05 | 4.64679 |
| 3405515 | APOLD1 | NM_030817 | 8.67E-04 | 4.60153 |
| 3470523 | SELPLG | NM_003006 | 2.11E-05 | 4.58875 |
| 2699564 | PLOD2 | NM_182943 | 3.03E-06 | 4.56441 |
| 3244622 | ALOX5 | NM_000698 | 3.64E-04 | 4.55321 |
| 3325503 | RCN1 | NM_002901 | 2.60E-06 | 4.54161 |
| 2409104 | SLC2A1 | NM_006516 | 6.18E-06 | 4.51715 |
| 3753860 | CCL5 | NM_002985 | 1.04E-04 | 4.49621 |
| 2900091 | HIST1H2AL | NM_003511 | 1.92E-05 | 4.46931 |
| 2838201 | PTTG1 | NM_004219 | 2.57E-06 | 4.40421 |
| 2899298 | BTN3A2 | NM_007047 | 2.31E-04 | 4.39385 |
| 3753500 | SLFN11 | NM_001104587 | 8.84E-05 | 4.38246 |
| 2601648 | DOCK10 | NM_014689 | 2.04E-08 | 4.37963 |
| 3896034 | RASSF2 | NM_014737 | 1.27E-05 | 4.36655 |
| 3837431 | EHD2 | NM_014601 | 2.78E-06 | 4.3595 |
| 3251393 | DDIT4 | NM_019058 | 1.73E-05 | 4.34115 |
| 4001556 | PHKA2 | NM_000292 | 1.14E-06 | 4.33062 |
| 2879739 | PRELID2 | NM_182960 | 9.97E-06 | 4.30321 |
| 3374934 | MS4A6A | NM_152852 | 8.82E-06 | 4.30088 |
| 2473936 | KCNK3 | NM_002246 | 9.85E-06 | 4.2857 |
| 3868998 | NKG7 | NM_005601 | 1.24E-04 | 4.28212 |
| 3752258 | EVI2B | NM_006495 | 1.14E-04 | 4.27666 |
| 2923661 | GJA1 | NM_000165 | 7.66E-06 | 4.27475 |
| 3402506 | CD27 | NM_001242 | 6.00E-04 | 4.26754 |
| 2357845 | FCGR1A | NM_000566 | 6.08E-08 | 4.25004 |
| 3864551 | PLAUR | NM_002659 | 3.23E-04 | 4.23317 |
| 2950263 | HLA-DMB | NM_002118 | 8.14E-06 | 4.19985 |
| 3115504 | MYC | NM_002467 | 3.83E-05 | 4.1948 |
| 2441043 | OLFML2B | NM_015441 | 1.12E-06 | 4.16144 |
| 2860178 | CD180 | NM_005582 | 2.53E-07 | 4.15021 |
| 3944129 | HMOX1 | NM_002133 | 2.74E-04 | 4.14397 |
| 3351300 | CD3G | NM_000073 | 1.20E-03 | 4.13735 |
| 3880629 | CST7 | NM_003650 | 2.66E-04 | 4.1187 |
| 3318443 | TRIM22 | NM_006074 | 5.93E-06 | 4.11699 |
| 3434726 | P2RX7 | NM_002562 | 1.48E-06 | 4.11318 |
| 2992814 | GPNMB | NM_001005340 | 2.30E-07 | 4.09775 |
| 2378937 | DTL | NM_016448 | 3.83E-09 | 4.08564 |
| 2395146 | TNFRSF9 | NM_001561 | 3.67E-04 | 4.07676 |
| 2840036 | DOCK2 | NM_004946 | 2.70E-06 | 4.05486 |
| 2324884 | C1QB | NM_000491 | 3.47E-06 | 4.04002 |
| 3329649 | DDB2 | NM_000107 | 5.57E-16 | 4.02824 |
| 2854327 | FYB | NM_001465 | 8.42E-07 | 4.00234 |
| 3351280 | CD3E | NM_000733 | 7.89E-04 | 3.99354 |
| 3389077 | PDGFD | NM_025208 | 4.14E-04 | 3.99187 |
| 3655986 | CORO1A | NM_007074 | 5.25E-05 | 3.9767 |
| 2469252 | RRM2 | NM_001165931 | 1.53E-03 | 3.92852 |
| 3389353 | CASP1 | NM_033292 | 3.88E-04 | 3.92742 |
| 3945651 | APOBEC3G | NM_021822 | 7.71E-07 | 3.91931 |
| 3687277 | SEZ6L2 | NM_012410 | 5.75E-05 | 3.91307 |
| 2908179 | VEGFA | NM_001025366 | 6.56E-06 | 3.90784 |
| 3486956 | C13orf15 | NM_014059 | 7.77E-04 | 3.89877 |
| 3847989 | CD70 | NM_001252 | 1.15E-04 | 3.89569 |
| 3161113 | PDCD1LG2 | NM_025239 | 2.46E-04 | 3.89538 |
| 3775842 | TYMS | NM_001071 | 8.15E-06 | 3.89426 |
| 3665230 | HSF4 | NM_001040667 | 1.69E-05 | 3.89206 |
| 3218528 | ABCA1 | NM_005502 | 1.78E-06 | 3.88829 |
| 3046197 | ELMO1 | NM_014800 | 7.89E-05 | 3.87887 |
| 2362394 | IFI16 | NM_005531 | 2.48E-05 | 3.8559 |
| 3075531 | ZC3HAV1L | NM_080660 | 3.10E-04 | 3.83716 |
| 3442854 | SLC2A3 | NM_006931 | 1.27E-03 | 3.83148 |
| 2326463 | CD52 | NM_001803 | 7.05E-04 | 3.81119 |
| 3428268 | GAS2L3 | NM_174942 | 8.38E-04 | 3.78381 |
| 2403557 | SNORA44 | NR_002976 | 1.15E-04 | 3.77322 |
| 3904691 | SAMHD1 | NM_015474 | 5.95E-05 | 3.77319 |
| 3820443 | ICAM1 | NM_000201 | 4.48E-05 | 3.75509 |
| 2638789 | CD86 | NM_175862 | 1.79E-06 | 3.75489 |
| 2777113 | SPARCL1 | NM_001128310 | 4.69E-04 | 3.75276 |
| 3351166 | IL10RA | NR_026691 | 2.99E-05 | 3.74213 |
| 3861948 | GMFG | NM_004877 | 7.12E-05 | 3.737 |
| 3883441 | SPAG4 | NM_003116 | 3.06E-07 | 3.73579 |
| 2806643 | SLC1A3 | NM_004172 | 6.26E-06 | 3.72963 |
| 3934729 | ITGB2 | NM_000211 | 1.39E-05 | 3.72584 |
| 3102096 | PREX2 | NM_024870 | 1.19E-03 | 3.72435 |
| 3507282 | FLT1 | NM_002019 | 3.03E-05 | 3.72367 |
| 2421883 | GBP1 | NM_002053 | 4.03E-04 | 3.71405 |
| 2887164 | SH3PXD2B | NM_001017995 | 2.52E-06 | 3.70954 |
| 4021250 | APLN | NM_017413 | 2.30E-05 | 3.70876 |
| 3300597 | MYOF | NM_013451 | 1.86E-08 | 3.70766 |
| 3491486 | PCDH17 | NM_001040429 | 1.02E-05 | 3.70574 |
| 3225855 | ANGPTL2 | NM_012098 | 9.51E-05 | 3.70365 |
| 3299585 | LIPA | NM_001127605 | 1.94E-10 | 3.7031 |
| 2947081 | HIST1H4L | NM_003546 | 1.07E-05 | 3.70176 |
| 3957429 | GAL3ST1 | NM_004861 | 8.08E-04 | 3.68676 |
| 3129304 | ZNF395 | NM_018660 | 1.24E-05 | 3.67497 |
| 3236958 | VIM | NM_003380 | 1.87E-05 | 3.66599 |
| 3564790 | ERO1L | NM_014584 | 3.12E-10 | 3.64912 |
| 3905145 | TGM2 | NM_004613 | 1.05E-03 | 3.64203 |
| 2893392 | LY86 | NM_004271 | 1.50E-05 | 3.6266 |
| 3209384 | TMEM2 | NM_013390 | 5.22E-07 | 3.6257 |
| 3944882 | LGALS1 | NM_002305 | 1.62E-05 | 3.60628 |
| 2809831 | GPX8 | NM_001008397 | 9.53E-06 | 3.59848 |
| 3335697 | CTSW | NM_001335 | 6.14E-04 | 3.59338 |
| 3012978 | GNG11 | NM_004126 | 5.03E-05 | 3.59255 |
| 2883283 | TIMD4 | NM_138379 | 3.76E-04 | 3.55823 |
| 3623031 | FBN1 | NM_000138 | 5.89E-05 | 3.54754 |
| 2791419 | FAM198B | NM_001128424 | 1.04E-03 | 3.54675 |
| 2440354 | CD48 | NM_001778 | 4.36E-04 | 3.54093 |
| 3992408 | FHL1 | NM_001159702 | 7.16E-04 | 3.53521 |
| 3657041 | ITGAX | NM_000887 | 3.96E-06 | 3.53176 |
| 2562932 | CD8A | NM_001145873 | 4.74E-04 | 3.52499 |
| 3945614 | APOBEC3F | NM_145298 | 1.14E-08 | 3.51671 |
| 3338192 | CCND1 | NM_053056 | 5.17E-05 | 3.50085 |
| 3945545 | APOBEC3B | NM_004900 | 5.95E-06 | 3.48543 |
| 3824471 | GLT25D1 | NM_024656 | 1.06E-07 | 3.48312 |
| 3468743 | NT5DC3 | NM_001031701 | 3.71E-04 | 3.47784 |
| 2783316 | SEC24D | NM_014822 | 6.97E-07 | 3.47754 |
| 2814756 | MAP1B | NM_005909 | 2.78E-06 | 3.47379 |
| 3734379 | CD300A | NM_007261 | 4.75E-05 | 3.46705 |
| 3445786 | ARHGDIB | NM_001175 | 1.89E-05 | 3.46542 |
| 3360800 | PRKCDBP | NM_145040 | 8.94E-06 | 3.458 |
| 3127703 | TNFRSF10B | NM_003842 | 8.45E-09 | 3.44559 |
| 3452231 | SLC38A1 | NM_030674 | 8.53E-06 | 3.42611 |
| 3432514 | OAS2 | NM_002535 | 3.51E-04 | 3.40428 |
| 2752006 | SAP30 | NM_003864 | 5.99E-08 | 3.40378 |
| 3123675 | PPP1R3B | NM_024607 | 9.87E-05 | 3.40333 |
| 2832403 | PCDHB9 | NM_019119 | 7.12E-04 | 3.40107 |
| 2657025 | RTP4 | NM_022147 | 1.07E-03 | 3.39848 |
| 2875348 | IRF1 | NM_002198 | 1.61E-04 | 3.38647 |
| 3901055 | CD93 | NM_012072 | 1.54E-04 | 3.36912 |
| 4027708 | MTCP1 | NM_001018025 | 9.53E-05 | 3.35655 |
| 2419432 | ELTD1 | NM_022159 | 6.97E-04 | 3.35249 |
| 3477917 | SLC15A4 | NM_145648 | 1.26E-07 | 3.34521 |
| 2879105 | SPRY4 | NM_030964 | 2.30E-04 | 3.33277 |
| 3141755 | HEY1 | NM_012258 | 8.39E-07 | 3.32942 |
| 3951768 | CECR1 | AK292689 | 2.20E-04 | 3.32928 |
| 3426502 | PLXNC1 | NM_005761 | 7.61E-07 | 3.32587 |
| 2541699 | FAM49A | NM_030797 | 1.34E-05 | 3.32536 |
| 3837257 | C5AR1 | NM_001736 | 1.06E-04 | 3.30959 |
| 3332334 | MS4A14 | NM_032597 | 3.35E-06 | 3.30814 |
| 3015519 | PILRA | NM_013439 | 2.46E-04 | 3.30705 |
| 3525313 | COL4A1 | NM_001845 | 3.99E-05 | 3.28684 |
| 3590388 | NUSAP1 | NM_016359 | 1.28E-08 | 3.2866 |
| 3527662 | RNASE6 | NM_005615 | 1.36E-04 | 3.28588 |
| 2639225 | PDIA5 | NM_006810 | 1.55E-04 | 3.27839 |
| 2946215 | HIST1H3B | NM_003537 | 2.80E-06 | 3.26809 |
| 3756193 | TOP2A | NM_001067 | 4.05E-06 | 3.26773 |
| 2518272 | ITGA4 | NM_000885 | 3.23E-06 | 3.25678 |
| 3027204 | TBXAS1 | NM_001130966 | 1.27E-08 | 3.25404 |
| 3708644 | FGF11 | NM_004112 | 5.79E-04 | 3.251 |
| 3569814 | ACTN1 | NM_001130004 | 5.28E-06 | 3.24914 |
| 2946714 | HIST1H2BK | NM_080593 | 1.42E-03 | 3.24802 |
| 2903258 | HLA-DQA2 | NM_020056 | 8.97E-05 | 3.24786 |
| 3960061 | RAC2 | NM_002872 | 5.69E-04 | 3.23961 |
| 3471005 | GIT2 | NM_057169 | 2.25E-07 | 3.23908 |
| 3976341 | TIMP1 | NM_003254 | 4.33E-04 | 3.22934 |
| 3657367 | ZNF267 | NM_003414 | 5.82E-09 | 3.2254 |
| 3798829 | FAM38B | NM_022068 | 2.17E-04 | 3.22535 |
| 2878437 | CD14 | NM_001040021 | 3.12E-04 | 3.22404 |
| 3090697 | CDCA2 | NM_152562 | 3.77E-06 | 3.21608 |
| 3948047 | PARVG | NM_022141 | 4.28E-06 | 3.21608 |
| 3792656 | CCDC102B | NM_024781 | 9.86E-05 | 3.21186 |
| 3629103 | KIAA0101 | NM_014736 | 8.39E-06 | 3.21004 |
| 3766796 | PECAM1 | NM_000442 | 6.08E-05 | 3.21004 |
| 3040897 | CDCA7L | NM_001127370 | 3.52E-07 | 3.2084 |
| 3103523 | LY96 | NM_015364 | 1.16E-04 | 3.20397 |
| 3154263 | SLA | NM_001045556 | 2.92E-04 | 3.20173 |
| 2488252 | DYSF | NM_001130978 | 4.20E-05 | 3.2008 |
| 3649890 | ABCC1 | NM_004996 | 6.08E-05 | 3.19838 |
| 2440258 | SLAMF6 | NM_052931 | 6.09E-04 | 3.18081 |
| 2950199 | PSMB8 | NM_004159 | 4.62E-07 | 3.17556 |
| 2988594 | SLC29A4 | NM_001040661 | 2.82E-04 | 3.15622 |
| 2635741 | CD96 | NM_198196 | 3.51E-04 | 3.15566 |
| 3808854 | TCF4 | NM_001083962 | 3.78E-04 | 3.15014 |
| 3023060 | CALU | NM_001219 | 1.27E-06 | 3.14827 |
| 3726691 | ABCC3 | NM_003786 | 2.13E-04 | 3.14783 |
| 2748346 | TLR2 | NM_003264 | 2.25E-05 | 3.13964 |
| 3944404 | APOL1 | NM_145343 | 1.02E-04 | 3.13644 |
| 3031556 | GIMAP2 | NM_015660 | 3.42E-04 | 3.11766 |
| 3799461 | SPIRE1 | NM_001128626 | 7.23E-07 | 3.11062 |
| 3752271 | EVI2A | NM_001003927 | 7.55E-05 | 3.10254 |
| 3977067 | PLP2 | NM_002668 | 1.05E-04 | 3.10053 |
| 3881443 | TPX2 | NM_012112 | 2.11E-04 | 3.09624 |
| 2447414 | NCF2 | NM_000433 | 2.56E-04 | 3.09432 |
| 2991150 | TSPAN13 | NM_014399 | 4.14E-04 | 3.09185 |
| 3863021 | TGFB1 | NM_000660 | 4.34E-04 | 3.08893 |
| 3257031 | STAMBPL1 | NM_020799 | 1.60E-07 | 3.08875 |
| 2835792 | GM2A | NM_000405 | 2.52E-10 | 3.0871 |
| 3453837 | TUBA1A | NM_006009 | 1.05E-04 | 3.07726 |
| 3687363 | DOC2A | NM_003586 | 7.34E-04 | 3.07486 |
| 3547375 | GPR65 | NM_003608 | 1.82E-04 | 3.073 |
| 3402786 | CD4 | NM_000616 | 1.32E-05 | 3.06954 |
| 2881187 | CSF1R | NM_005211 | 2.68E-04 | 3.06582 |
| 2591367 | CALCRL | NM_005795 | 6.51E-04 | 3.06159 |
| 3854417 | PLVAP | NM_031310 | 3.87E-04 | 3.05717 |
| 2742224 | SPRY1 | NM_005841 | 2.68E-04 | 3.05677 |
| 2427981 | ADORA3 | NM_020683 | 1.98E-06 | 3.05282 |
| 2927506 | TNFAIP3 | NM_006290 | 1.59E-04 | 3.05217 |
| 2353669 | CD2 | NM_001767 | 1.10E-03 | 3.05149 |
| 2403446 | PTAFR | NM_001164721 | 9.44E-05 | 3.0428 |
| 3852880 | EMR2 | NM_013447 | 1.15E-04 | 3.02914 |
| 3852565 | ASF1B | NM_018154 | 5.79E-08 | 3.02642 |
| 2899372 | BTN3A1 | NM_001145009 | 3.54E-05 | 3.02377 |
| 2524301 | NRP2 | NM_201266 | 1.02E-04 | 3.02262 |
| 2400793 | HSPG2 | NM_005529 | 4.19E-04 | 3.01357 |
| 3672489 | IRF8 | NM_002163 | 3.38E-04 | 3.0097 |
| 4011008 | VSIG4 | NM_007268 | 1.07E-03 | 3.0069 |
| 3118818 | PTP4A3 | NM_032611 | 1.46E-08 | 3.00383 |
| 3294159 | P4HA1 | NM_000917 | 1.75E-05 | 2.99681 |
| 3020192 | TES | NM_015641 | 2.07E-05 | 2.99061 |
| 3186966 | TLR4 | NR_024168 | 4.82E-05 | 2.98964 |
| 3444086 | KLRK1 | NM_007360 | 2.92E-04 | 2.98946 |
| 2363202 | SLAMF7 | NM_021181 | 9.12E-06 | 2.98914 |
| 3168385 | GLIPR2 | NM_022343 | 2.72E-04 | 2.98774 |
| 2463864 | CEP170 | NM_014812 | 3.56E-06 | 2.98499 |
| 3841621 | LILRB4 | NM_006847 | 1.91E-06 | 2.97245 |
| 3654175 | IL4R | NM_000418 | 1.58E-05 | 2.97169 |
| 3020343 | MET | NM_001127500 | 1.65E-08 | 2.96328 |
| 2997376 | ANLN | NM_018685 | 2.37E-04 | 2.96198 |
| 3959986 | IL2RB | NM_000878 | 7.47E-05 | 2.957 |
| 2486927 | ARHGAP25 | NM_014882 | 1.82E-06 | 2.94828 |
| 3061438 | SAMD9 | NM_017654 | 6.11E-04 | 2.93922 |
| 3301218 | PDLIM1 | NM_020992 | 2.34E-05 | 2.93724 |
| 2739160 | CCDC109B | NM_017918 | 5.91E-05 | 2.93154 |
| 3918447 | IFNAR2 | NM_207585 | 2.51E-06 | 2.92791 |
| 2832423 | PCDHB10 | NM_018930 | 1.01E-04 | 2.91578 |
| 2639054 | PARP14 | NM_017554 | 4.89E-06 | 2.90643 |
| 2866225 | MEF2C | NM_002397 | 7.72E-05 | 2.9047 |
| 3090294 | ADAMDEC1 | NM_014479 | 9.03E-04 | 2.90287 |
| 3378818 | PTPRCAP | NM_005608 | 7.70E-04 | 2.9 |
| 3331903 | FAM111B | NM_198947 | 2.18E-05 | 2.89705 |
| 2558612 | TGFA | NM_003236 | 9.09E-04 | 2.8944 |
| 3998766 | KAL1 | NM_000216 | 8.24E-04 | 2.89129 |
| 3779579 | TUBB6 | NM_032525 | 1.80E-05 | 2.88718 |
| 3381817 | UCP2 | NM_003355 | 1.58E-04 | 2.88457 |
| 3911217 | PMEPA1 | NM_020182 | 5.08E-05 | 2.88427 |
| 3838067 | BAX | NM_004324 | 5.48E-08 | 2.88246 |
| 2638962 | DTX3L | NM_138287 | 2.96E-07 | 2.87792 |
| 3708858 | CD68 | NM_001251 | 6.89E-05 | 2.86868 |
| 2421925 | GBP7 | NM_207398 | 1.73E-05 | 2.86401 |
| 2809810 | GZMA | NM_006144 | 4.34E-04 | 2.86176 |
| 2903189 | HLA-DRA | NM_019111 | 4.66E-06 | 2.85506 |
| 3222144 | TNFSF8 | NM_001244 | 4.78E-05 | 2.8537 |
| 3528864 | MMP14 | NM_004995 | 1.23E-05 | 2.85004 |
| 3917204 | C21orf7 | NM_020152 | 1.50E-05 | 2.84962 |
| 3873160 | TRIB3 | NM_021158 | 8.80E-05 | 2.84904 |
| 2808748 | PARP8 | NM_024615 | 2.87E-06 | 2.84578 |
| 3966000 | TYMP | NM_001113756 | 2.57E-06 | 2.84057 |
| 4023467 | ARHGEF6 | NM_004840 | 2.34E-05 | 2.83745 |
| 2489172 | MTHFD2 | NR_027405 | 3.64E-05 | 2.83559 |
| 3417842 | LRP1 | NM_002332 | 1.27E-04 | 2.83059 |
| 2327283 | C1orf38 | NM_001105556 | 6.06E-05 | 2.82236 |
| 3854454 | BST2 | NM_004335 | 3.68E-06 | 2.81568 |
| 3908631 | PREX1 | NM_020820 | 8.26E-06 | 2.81066 |
| 3018309 | PIK3CG | NM_002649 | 2.32E-04 | 2.81062 |
| 3454841 | BIN2 | NM_016293 | 3.49E-05 | 2.8074 |
| 3467315 | IKBIP | NM_153687 | 2.57E-08 | 2.80692 |
| 3869030 | SIGLEC10 | NM_033130 | 6.03E-08 | 2.8049 |
| 3945585 | APOBEC3D | NM_152426 | 6.18E-09 | 2.8028 |
| 2691668 | HCLS1 | NM_005335 | 8.65E-05 | 2.8023 |
| 3798778 | FAM38B | NM_022068 | 6.93E-05 | 2.80215 |
| 3239760 | APBB1IP | NM_019043 | 5.40E-04 | 2.79964 |
| 3078348 | EZH2 | NM_004456 | 1.12E-06 | 2.77861 |
| 3159330 | DOCK8 | NM_203447 | 1.18E-05 | 2.77727 |
| 3969115 | TLR8 | NM_138636 | 1.24E-04 | 2.77291 |
| 3375091 | SLC15A3 | NM_016582 | 9.68E-07 | 2.77224 |
| 3718555 | SLFN5 | NM_144975 | 1.27E-04 | 2.76792 |
| 2946369 | HIST1H3G | NM_003534 | 1.01E-05 | 2.76326 |
| 2845274 | CCDC127 | NM_145265 | 1.70E-05 | 2.75936 |
| 3444252 | CSDA | NM_003651 | 1.64E-06 | 2.75794 |
| 2404209 | SDC3 | NM_014654 | 2.51E-05 | 2.75429 |
| 3397589 | ETS1 | NM_001143820 | 9.21E-04 | 2.75316 |
| 2903782 | ITPR3 | NM_002224 | 7.12E-04 | 2.75312 |
| 3025740 | TMEM140 | NM_018295 | 4.30E-06 | 2.75164 |
| 2859667 | CENPK | NM_022145 | 3.94E-06 | 2.75124 |
| 3664664 | CDH5 | NM_001795 | 1.18E-03 | 2.73985 |
| 3560711 | BAZ1A | NM_013448 | 8.63E-07 | 2.73504 |
| 3778504 | RAB31 | NM_006868 | 6.53E-08 | 2.73291 |
| 2902326 | HCP5 | NM_006674 | 2.37E-05 | 2.73055 |
| 2950277 | HLA-DMA | NM_006120 | 9.70E-05 | 2.72898 |
| 2362333 | MNDA | NM_002432 | 4.57E-05 | 2.72681 |
| 3257204 | IFIT3 | NM_001031683 | 2.05E-04 | 2.72623 |
| 3922444 | ABCG1 | NM_207628 | 1.68E-06 | 2.72169 |
| 3031533 | GIMAP4 | NM_018326 | 3.45E-04 | 2.71536 |
| 2496727 | MAP4K4 | NM_145686 | 1.63E-05 | 2.71466 |
| 2777487 | FAM13A | NM_001015045 | 8.13E-06 | 2.71093 |
| 3098977 | LYN | NM_002350 | 1.54E-04 | 2.7078 |
| 3312490 | MKI67 | NM_002417 | 1.50E-05 | 2.70773 |
| 3355956 | BARX2 | NM_003658 | 1.13E-04 | 2.70306 |
| 3446796 | RECQL | NM_002907 | 1.25E-09 | 2.70094 |
| 4027769 | CLIC2 | NM_001289 | 1.45E-03 | 2.6934 |
| 3656223 | ITGAL | NM_002209 | 2.45E-04 | 2.69325 |
| 2428796 | PTPN22 | NM_015967 | 9.83E-05 | 2.69182 |
| 3837731 | EMP3 | NM_001425 | 1.71E-05 | 2.68833 |
| 2929127 | STX11 | NM_003764 | 4.44E-04 | 2.68623 |
| 3372174 | SPI1 | NM_001080547 | 4.35E-06 | 2.68584 |
| 4027176 | FLNA | NM_001456 | 1.79E-04 | 2.68534 |
| 3334257 | FERMT3 | NM_178443 | 3.10E-05 | 2.68452 |
| 3834046 | AXL | NM_021913 | 1.24E-04 | 2.68055 |
| 2655168 | YEATS2 | NM_018023 | 2.77E-07 | 2.68045 |
| 3160175 | VLDLR | NM_003383 | 1.08E-04 | 2.6804 |
| 3943207 | YWHAH | NM_003405 | 1.61E-05 | 2.67946 |
| 3500787 | TNFSF13B | NM_006573 | 4.20E-05 | 2.67593 |
| 3371003 | TP53I11 | NM_001076787 | 3.35E-06 | 2.6696 |
| 3832992 | PLEKHG2 | NM_022835 | 9.60E-06 | 2.66535 |
| 3051395 | SEC61G | NM_014302 | 2.04E-04 | 2.66422 |
| 3318173 | OR51E1 | NM_152430 | 4.60E-05 | 2.65907 |
| 2345929 | LRRC8C | NM_032270 | 9.53E-04 | 2.65453 |
| 2899206 | HIST1H2BF | NM_003522 | 7.25E-05 | 2.65185 |
| 3841076 | MYADM | NM_001020818 | 2.18E-05 | 2.65062 |
| 2754673 | ANKRD37 | NM_181726 | 6.56E-05 | 2.64686 |
| 3068097 | DOCK4 | NM_014705 | 3.55E-06 | 2.64653 |
| 2532699 | INPP5D | NM_001017915 | 2.31E-04 | 2.64442 |
| 2694817 | PLXND1 | NM_015103 | 7.17E-05 | 2.64389 |
| 3635198 | BCL2A1 | NM_001114735 | 1.10E-03 | 2.64005 |
| 3881651 | HCK | NM_001172129 | 1.08E-05 | 2.63674 |
| 3110317 | CTHRC1 | NM_138455 | 3.86E-04 | 2.63527 |
| 3188883 | OLFML2A | NM_182487 | 1.78E-04 | 2.63339 |
| 2882098 | SPARC | NM_003118 | 9.28E-07 | 2.63304 |
| 3293435 | PRF1 | NM_005041 | 5.90E-04 | 2.63225 |
| 3944690 | CYTH4 | NM_013385 | 4.01E-04 | 2.63182 |
| 2487412 | ANXA4 | NM_001153 | 2.82E-06 | 2.6252 |
| 2784113 | CCNA2 | NM_001237 | 5.61E-06 | 2.62349 |
| 3703112 | GINS2 | NM_016095 | 7.31E-06 | 2.62167 |
| 3526831 | RASA3 | NM_007368 | 8.90E-06 | 2.60481 |
| 3930360 | RUNX1 | NM_001001890 | 5.36E-04 | 2.59992 |
| 3501219 | COL4A2 | NM_001846 | 4.27E-04 | 2.59958 |
| 2567583 | RNF149 | NM_173647 | 8.10E-05 | 2.59878 |
| 2491745 | GNLY | NM_012483 | 5.15E-04 | 2.59681 |
| 3270270 | PTPRE | NM_006504 | 1.84E-05 | 2.5965 |
| 2961347 | FILIP1 | NM_015687 | 6.54E-04 | 2.59543 |
| 3522662 | GPR183 | NM_004951 | 8.32E-04 | 2.59531 |
| 2492496 | NCRNA00152 | NR_024204 | 7.37E-05 | 2.59197 |
| 3040967 | RAPGEF5 | NM_012294 | 3.31E-04 | 2.58368 |
| 3665262 | NOL3 | NM_003946 | 4.77E-06 | 2.5787 |
| 2499053 | LIMS1 | NM_004987 | 3.70E-07 | 2.57743 |
| 3801411 | NPC1 | NM_000271 | 1.41E-05 | 2.5709 |
| 3429566 | CHST11 | NM_018413 | 6.90E-05 | 2.56692 |
| 3128411 | EBF2 | NM_022659 | 7.91E-04 | 2.56598 |
| 3986647 | VSIG1 | NM_001170553 | 1.03E-03 | 2.56307 |
| 2635349 | TRAT1 | NM_016388 | 4.18E-04 | 2.56062 |
| 3851826 | DNASE2 | NM_001375 | 4.15E-06 | 2.55954 |
| 3688311 | PYCARD | NM_013258 | 4.02E-06 | 2.55242 |
| 3375545 | FADS1 | NM_013402 | 1.53E-05 | 2.55179 |
| 2532894 | DGKD | NM_152879 | 1.85E-04 | 2.55055 |
| 3317071 | LSP1 | NM_002339 | 3.22E-04 | 2.54846 |
| 2376849 | RASSF5 | NM_182663 | 1.88E-04 | 2.54427 |
| 3791996 | SERPINB8 | NM_002640 | 1.65E-05 | 2.54363 |
| 3971768 | PRDX4 | NM_006406 | 1.34E-06 | 2.54043 |
| 3469180 | SLC41A2 | NM_032148 | 4.09E-05 | 2.53782 |
| 2842157 | HRH2 | NM_001131055 | 3.70E-05 | 2.53492 |
| 2724671 | RHOH | NM_004310 | 8.40E-05 | 2.5342 |
| 3355733 | FLI1 | NM_002017 | 1.18E-03 | 2.52601 |
| 3454006 | FMNL3 | NM_175736 | 1.86E-04 | 2.52594 |
| 2648378 | RAP2B | NM_002886 | 6.26E-08 | 2.52417 |
| 3374402 | LPXN | NM_001143995 | 2.95E-05 | 2.52381 |
| 2878622 | TAF7 | NM_005642 | 3.05E-06 | 2.52336 |
| 3607332 | ACAN | NM_013227 | 6.31E-04 | 2.52102 |
| 3887302 | CD40 | NM_001250 | 5.07E-08 | 2.51807 |
| 2460817 | SIPA1L2 | NM_020808 | 3.28E-08 | 2.51693 |
| 2371873 | HMCN1 | NM_031935 | 5.66E-04 | 2.51164 |
| 3063795 | GAL3ST4 | NM_024637 | 5.76E-08 | 2.50886 |
| 2654967 | B3GNT5 | NM_032047 | 5.45E-06 | 2.50649 |
| 2706938 | GNB4 | NM_021629 | 3.99E-07 | 2.50535 |
| 2748198 | KIAA0922 | NM_001131007 | 2.35E-05 | 2.50131 |
| 3607537 | FANCI | NM_001113378 | 2.60E-06 | 2.49104 |
| 2523874 | ICOS | NM_012092 | 6.99E-04 | 2.49102 |
| 3527722 | RNASE2 | NM_002934 | 1.43E-03 | 2.48769 |
| 4021433 | ELF4 | NM_001421 | 4.95E-08 | 2.48343 |
| 3830993 | HCST | NM_014266 | 1.41E-03 | 2.47944 |
| 3897505 | JAG1 | NM_000214 | 2.07E-04 | 2.47854 |
| 3524570 | EFNB2 | NM_004093 | 2.24E-04 | 2.4753 |
| 3664785 | CKLF | NM_016951 | 2.09E-05 | 2.47259 |
| 2553771 | CCDC88A | NM_001135597 | 1.60E-04 | 2.47141 |
| 3983962 | DIAPH2 | NM_007309 | 1.02E-03 | 2.46845 |
| 3662444 | NLRC5 | NM_032206 | 4.56E-06 | 2.46356 |
| 2939034 | SERPINB9 | NM_004155 | 5.77E-04 | 2.46322 |
| 3326950 | LDLRAD3 | NM_174902 | 2.92E-04 | 2.46249 |
| 3153428 | ASAP1 | NM_018482 | 1.75E-04 | 2.46198 |
| 3432467 | OAS3 | NM_006187 | 7.10E-06 | 2.46187 |
| 3696016 | PSMB10 | NM_002801 | 9.67E-08 | 2.45916 |
| 3250990 | UNC5B | NM_170744 | 5.37E-05 | 2.45886 |
| 2902427 | LST1 | NM_205837 | 9.45E-04 | 2.45634 |
| 2362351 | PYHIN1 | NM_152501 | 7.69E-04 | 2.45415 |
| 3378183 | CD248 | NM_020404 | 2.48E-04 | 2.45306 |
| 2491271 | TMSB10 | NM_021103 | 7.18E-05 | 2.45241 |
| 2485636 | SLC1A4 | NM_003038 | 1.91E-10 | 2.44883 |
| 2371065 | LAMC1 | NM_002293 | 1.64E-05 | 2.44656 |
| 2709778 | BCL6 | NM_001706 | 3.88E-04 | 2.44017 |
| 2950329 | HLA-DPA1 | NM_033554 | 3.46E-05 | 2.43993 |
| 3645477 | PAQR4 | NM_152341 | 4.24E-05 | 2.43975 |
| 3390180 | KDELC2 | NM_153705 | 9.90E-08 | 2.43815 |
| 3824713 | ARRDC2 | NM_015683 | 1.39E-05 | 2.43705 |
| 2991233 | AHR | NM_001621 | 2.37E-05 | 2.43456 |
| 3234760 | CELF2 | NM_001025077 | 9.62E-05 | 2.434 |
| 2358044 | PLEKHO1 | NM_016274 | 1.39E-04 | 2.43037 |
| 2903401 | HLA-DPB1 | NM_002121 | 8.41E-04 | 2.42577 |
| 3257192 | IFIT2 | NM_001547 | 3.13E-05 | 2.4242 |
| 2320727 | TNFRSF1B | NM_001066 | 1.75E-04 | 2.4218 |
| 2638077 | PLA1A | NM_015900 | 1.53E-03 | 2.4213 |
| 2570616 | BUB1 | NM_004336 | 6.13E-04 | 2.41759 |
| 2623922 | STAB1 | NM_015136 | 4.62E-04 | 2.41523 |
| 3783723 | RNF125 | NM_017831 | 1.19E-03 | 2.41416 |
| 3904566 | DSN1 | NM_001145316 | 6.16E-07 | 2.41201 |
| 2525533 | MAP2 | NM_002374 | 2.38E-04 | 2.40619 |
| 3360142 | TRIM21 | NM_003141 | 7.64E-06 | 2.40175 |
| 2976113 | IFNGR1 | NM_000416 | 7.97E-06 | 2.39909 |
| 2948547 | NRM | NM_007243 | 5.24E-04 | 2.39886 |
| 3974948 | GPR34 | NM_001097579 | 4.41E-04 | 2.39782 |
| 3815268 | KISS1R | NM_032551 | 2.67E-04 | 2.39158 |
| 3723378 | FMNL1 | NM_005892 | 8.04E-05 | 2.38921 |
| 2950307 | HLA-DOA | NM_002119 | 4.87E-04 | 2.38881 |
| 2887048 | STK10 | NM_005990 | 1.19E-04 | 2.38627 |
| 2391687 | NADK | NM_023018 | 3.79E-04 | 2.38617 |
| 2752725 | NEIL3 | NM_018248 | 7.49E-07 | 2.38518 |
| 2587841 | WIPF1 | NM_003387 | 1.01E-06 | 2.38266 |
| 3633578 | CSPG4 | NM_001897 | 1.27E-04 | 2.37382 |
| 3064541 | PLOD3 | NM_001084 | 5.45E-05 | 2.3736 |
| 3417988 | NXPH4 | NM_007224 | 1.81E-04 | 2.37312 |
| 2503257 | INHBB | NM_002193 | 2.16E-06 | 2.36527 |
| 2374126 | NR5A2 | NM_205860 | 3.27E-04 | 2.36153 |
| 3354443 | SLC37A2 | NM_198277 | 2.00E-05 | 2.35315 |
| 2332528 | RIMKLA | NM_173642 | 1.04E-05 | 2.35283 |
| 3944147 | MCM5 | NM_006739 | 3.39E-08 | 2.34989 |
| 2502424 | INSIG2 | NM_016133 | 2.32E-05 | 2.34868 |
| 3909553 | NFATC2 | NM_012340 | 2.75E-05 | 2.34821 |
| 2908100 | POLH | NM_006502 | 1.95E-05 | 2.34795 |
| 2508611 | ARHGAP15 | NM_018460 | 8.60E-04 | 2.3451 |
| 3001479 | IKZF1 | NM_006060 | 1.73E-04 | 2.34373 |
| 2694931 | TMCC1 | NM_001017395 | 6.15E-09 | 2.34299 |
| 3174816 | ANXA1 | NM_000700 | 4.48E-04 | 2.34094 |
| 2868131 | ERAP1 | NM_001040458 | 1.87E-05 | 2.33903 |
| 2792800 | DDX60 | NM_017631 | 3.78E-05 | 2.33303 |
| 2327572 | RAB42 | NM_152304 | 5.60E-04 | 2.32341 |
| 2544484 | ADCY3 | NM_004036 | 1.61E-04 | 2.32228 |
| 2406926 | GRIK3 | NM_000831 | 3.01E-04 | 2.31728 |
| 3340589 | SERPINH1 | NM_001235 | 6.85E-06 | 2.31383 |
| 2830638 | KIF20A | NM_005733 | 7.94E-05 | 2.30579 |
| 2969810 | TRAF3IP2 | NR_028338 | 1.77E-04 | 2.30336 |
| 2948926 | HLA-B | NM_005514 | 1.67E-06 | 2.29701 |
| 3748659 | GRAP | NM_006613 | 8.81E-04 | 2.29185 |
| 3089215 | BMP1 | NR_033403 | 7.02E-04 | 2.29071 |
| 2646327 | C3orf58 | NM_173552 | 3.80E-07 | 2.29069 |
| 3447694 | BCAT1 | NM_005504 | 1.77E-04 | 2.28632 |
| 2889542 | COL23A1 | NM_173465 | 1.10E-05 | 2.27962 |
| 3639031 | PRC1 | NM_003981 | 8.65E-07 | 2.27925 |
| 2905169 | CDKN1A | NM_078467 | 1.28E-05 | 2.27911 |
| 3393720 | MPZL2 | NM_144765 | 1.95E-04 | 2.27906 |
| 3432438 | OAS1 | NM_016816 | 2.04E-04 | 2.27714 |
| 2856044 | EMB | NM_198449 | 2.28E-04 | 2.27172 |
| 2515933 | ZAK | NM_016653 | 1.57E-05 | 2.26858 |
| 3707095 | ARRB2 | NM_004313 | 1.14E-06 | 2.26587 |
| 3337390 | TCIRG1 | NM_006019 | 5.94E-05 | 2.26321 |
| 2331857 | SMAP2 | NM_022733 | 6.75E-05 | 2.26224 |
| 2492064 | KDM3A | NM_018433 | 2.98E-07 | 2.26179 |
| 3664843 | CMTM3 | NM_144601 | 1.35E-03 | 2.26143 |
| 2908261 | C6orf223 | NM_153246 | 7.62E-05 | 2.26106 |
| 2692060 | PARP9 | NM_001146106 | 5.50E-05 | 2.25482 |
| 3441542 | ANO2 | NM_020373 | 3.63E-05 | 2.25344 |
| 2881370 | CD74 | NM_001025159 | 4.91E-05 | 2.25223 |
| 3373962 | UBE2L6 | NM_004223 | 5.35E-06 | 2.25014 |
| 2881413 | RPS14 | NM_001025071 | 2.70E-04 | 2.2501 |
| 2880292 | DPYSL3 | NM_001387 | 1.44E-03 | 2.24551 |
| 3823982 | MYO9B | NM_004145 | 7.62E-06 | 2.2435 |
| 2515783 | RAPGEF4 | NM_007023 | 4.68E-04 | 2.24157 |
| 3849044 | MYO1F | NM_012335 | 5.93E-05 | 2.24053 |
| 3918535 | IL10RB | NM_000628 | 5.13E-06 | 2.24034 |
| 3421300 | MDM2 | NM_002392 | 7.41E-09 | 2.23979 |
| 2580955 | NMI | NM_004688 | 5.31E-04 | 2.23872 |
| 2463515 | CHML | NM_001821 | 2.79E-05 | 2.22985 |
| 3200611 | HAUS6 | NM_017645 | 1.04E-05 | 2.22722 |
| 2880905 | CSNK1A1 | NM_001025105 | 7.57E-08 | 2.22644 |
| 3963676 | C22orf9 | NM_001009880 | 5.95E-04 | 2.2263 |
| 3742285 | CXCL16 | NM_022059 | 1.47E-03 | 2.22595 |
| 3162529 | C9orf150 | NM_203403 | 2.23E-07 | 2.22308 |
| 2832467 | PCDHB18 | NR_001281 | 9.04E-04 | 2.22125 |
| 3260001 | MARVELD1 | NM_031484 | 8.55E-04 | 2.22124 |
| 2690956 | POPDC2 | NM_022135 | 8.39E-04 | 2.21469 |
| 3505319 | SACS | NM_014363 | 2.47E-06 | 2.20853 |
| 3894601 | FKBP1A | NM_000801 | 5.23E-05 | 2.20709 |
| 2447877 | FAM129A | NM_052966 | 3.01E-05 | 2.20358 |
| 3875908 | PLCB4 | NM_001172646 | 5.55E-04 | 2.20118 |
| 3653072 | PLK1 | NM_005030 | 7.55E-04 | 2.19797 |
| 3830216 | FXYD5 | NR_028406 | 4.12E-04 | 2.19698 |
| 3257098 | FAS | NM_000043 | 6.83E-07 | 2.19565 |
| 2899413 | BTN3A3 | NM_006994 | 3.61E-04 | 2.19491 |
| 3975869 | RP2 | NM_006915 | 6.50E-09 | 2.19321 |
| 3960478 | CSNK1E | NM_001894 | 3.77E-06 | 2.18883 |
| 3699080 | MLKL | NM_152649 | 2.66E-05 | 2.18878 |
| 2379754 | SMYD2 | NM_020197 | 8.60E-06 | 2.18554 |
| 3347658 | ATM | NM_000051 | 1.01E-07 | 2.18449 |
| 3562746 | C14orf106 | NM_018353 | 8.35E-05 | 2.18348 |
| 3605268 | TM6SF1 | NM_023003 | 3.20E-04 | 2.18054 |
| 2884216 | RNF145 | NM_144726 | 2.77E-08 | 2.1754 |
| 3063685 | MCM7 | NM_005916 | 4.01E-06 | 2.17538 |
| 3403595 | CLEC4A | NM_016184 | 8.92E-04 | 2.175 |
| 2901620 | HLA-E | NM_005516 | 2.04E-04 | 2.16933 |
| 3381925 | PGM2L1 | NM_173582 | 2.71E-04 | 2.16881 |
| 2839671 | RARS | NM_002887 | 1.22E-05 | 2.16665 |
| 3846926 | DPP9 | NM_139159 | 2.89E-07 | 2.16623 |
| 2796510 | MLF1IP | NM_024629 | 4.17E-06 | 2.16589 |
| 2966636 | ASCC3 | NM_006828 | 6.03E-07 | 2.16233 |
| 2435383 | S100A10 | NM_002966 | 1.03E-05 | 2.16202 |
| 3610982 | SYNM | NM_145728 | 2.70E-06 | 2.15872 |
| 3597338 | TPM1 | NM_000366 | 4.01E-05 | 2.151 |
| 3716950 | ADAP2 | NM_018404 | 7.82E-05 | 2.14994 |
| 2724094 | FAM114A1 | NM_138389 | 2.25E-04 | 2.149 |
| 3939470 | MMP11 | NM_005940 | 3.07E-04 | 2.14677 |
| 3601387 | PML | NM_033240 | 1.29E-07 | 2.14471 |
| 2873897 | MARCH3 | NM_178450 | 2.17E-04 | 2.14232 |
| 3404436 | CLEC2D | NM_001004419 | 4.70E-04 | 2.14043 |
| 3627248 | ANXA2 | NM_001002858 | 9.05E-06 | 2.13838 |
| 4049862 | EIF4EBP1 | NM_004095 | 2.30E-04 | 2.1373 |
| 3518977 | RNF219 | NM_024546 | 8.86E-07 | 2.13709 |
| 3665116 | CBFB | NM_001755 | 1.58E-05 | 2.13358 |
| 3545130 | VASH1 | NM_014909 | 3.08E-05 | 2.13352 |
| 3790361 | ZNF532 | NM_018181 | 3.80E-04 | 2.13337 |
| 2533999 | CXCR7 | NM_020311 | 1.24E-04 | 2.13091 |
| 3592023 | B2M | NM_004048 | 1.21E-07 | 2.12938 |
| 3354799 | CHEK1 | NM_001114122 | 1.63E-04 | 2.12734 |
| 2825629 | TNFAIP8 | NM_014350 | 5.36E-05 | 2.12254 |
| 2766262 | TLR6 | NM_006068 | 8.60E-04 | 2.1221 |
| 3389273 | CASP4 | NM_033306 | 3.32E-05 | 2.12068 |
| 2539821 | ADAM17 | NM_003183 | 2.44E-04 | 2.12059 |
| 3343452 | PRSS23 | NM_007173 | 3.04E-06 | 2.11769 |
| 3018535 | BCAP29 | NM_018844 | 2.17E-05 | 2.11655 |
| 3465409 | BTG1 | NM_001731 | 1.32E-04 | 2.11532 |
| 3784344 | MAPRE2 | NM_014268 | 2.53E-04 | 2.11286 |
| 3027503 | ADCK2 | NM_052853 | 9.09E-08 | 2.11138 |
| 3564250 | TRIM9 | NM_052978 | 3.45E-05 | 2.1085 |
| 3385752 | RAB38 | NM_022337 | 7.78E-05 | 2.10767 |
| 3106243 | RIPK2 | NM_003821 | 2.27E-04 | 2.10742 |
| 3228463 | RALGDS | NM_006266 | 4.10E-06 | 2.10198 |
| 3873185 | RBCK1 | NM_031229 | 6.78E-04 | 2.1019 |
| 3869714 | ZNF611 | NM_001161499 | 6.11E-07 | 2.10182 |
| 3631964 | PKM2 | NM_182470 | 1.87E-06 | 2.10118 |
| 3592511 | SQRDL | NM_021199 | 2.08E-04 | 2.10029 |
| 2901913 | TUBB | NM_178014 | 7.24E-05 | 2.09727 |
| 3815243 | CFD | NM_001928 | 1.27E-04 | 2.09679 |
| 2902178 | TCF19 | NM_007109 | 1.51E-03 | 2.09057 |
| 3419898 | RASSF3 | NM_178169 | 1.26E-03 | 2.08905 |
| 2690900 | CD80 | NM_005191 | 1.21E-05 | 2.08603 |
| 2922215 | MARCKS | NM_002356 | 4.74E-05 | 2.08493 |
| 3838004 | PPP1R15A | NM_014330 | 9.34E-04 | 2.08426 |
| 3056414 | RFC2 | NM_181471 | 1.91E-05 | 2.08244 |
| 2500615 | TMEM87B | NM_032824 | 4.23E-05 | 2.07966 |
| 3656990 | ITGAM | NM_001145808 | 6.65E-04 | 2.0785 |
| 2487082 | ANTXR1 | NM_032208 | 1.12E-03 | 2.07846 |
| 3404660 | KLRD1 | NM_002262 | 7.75E-04 | 2.07753 |
| 3416651 | PDE1B | NM_000924 | 1.38E-03 | 2.07744 |
| 2622547 | SEMA3F | NM_004186 | 1.09E-05 | 2.07576 |
| 3771602 | RHBDF2 | NM_024599 | 2.59E-07 | 2.07335 |
| 3225952 | FAM129B | NM_022833 | 4.11E-05 | 2.07303 |
| 2650199 | SMC4 | NM_005496 | 3.19E-07 | 2.07035 |
| 3712062 | TRPV2 | NM_016113 | 1.05E-04 | 2.06821 |
| 3435362 | KNTC1 | NM_014708 | 7.80E-06 | 2.06297 |
| 3305313 | ITPRIP | NM_033397 | 2.10E-04 | 2.06095 |
| 3757917 | PTRF | NM_012232 | 4.67E-04 | 2.05835 |
| 4015709 | BTK | NM_000061 | 6.98E-05 | 2.05718 |
| 3408831 | SSPN | NM_005086 | 1.27E-04 | 2.0521 |
| 3561868 | CLEC14A | NM_175060 | 1.51E-03 | 2.05185 |
| 3146012 | NIPAL2 | NM_024759 | 1.86E-04 | 2.05115 |
| 3634458 | TBC1D2B | NM_144572 | 9.26E-06 | 2.04499 |
| 2673312 | PFKFB4 | NM_004567 | 1.08E-04 | 2.04477 |
| 3968397 | WWC3 | NM_015691 | 7.28E-07 | 2.04402 |
| 3925473 | SAMSN1 | NM_022136 | 4.69E-04 | 2.04112 |
| 3914307 | RGS19 | NM_001039467 | 4.47E-05 | 2.03838 |
| 2950167 | TAP2 | NM_000544 | 1.52E-03 | 2.03813 |
| 3079005 | RARRES2 | NM_002889 | 1.26E-03 | 2.03565 |
| 3607232 | AEN | NM_022767 | 5.81E-06 | 2.03505 |
| 3911767 | CTSZ | NM_001336 | 1.06E-03 | 2.02974 |
| 2809128 | ITGA1 | NM_181501 | 2.37E-05 | 2.02883 |
| 3960110 | MFNG | NM_002405 | 6.21E-04 | 2.02667 |
| 3850725 | DOCK6 | NM_020812 | 4.04E-04 | 2.02578 |
| 2948522 | KIAA1949 | NM_133471 | 6.67E-06 | 2.0233 |
| 3029129 | ZYX | NM_003461 | 8.64E-04 | 2.02238 |
| 3082874 | ARHGEF10 | NM_014629 | 4.11E-04 | 2.02228 |
| 2645951 | TRPC1 | NM_003304 | 4.89E-04 | 2.02089 |
| 3601229 | CD276 | NM_001024736 | 7.56E-08 | 2.02064 |
| 3323413 | HTATIP2 | NM_001098522 | 6.43E-06 | 2.01827 |
| 2786657 | SETD7 | NM_030648 | 1.51E-04 | 2.01423 |
| 2681114 | C3orf64 | NM_173654 | 1.39E-05 | 2.01171 |
| 3944243 | APOL6 | NM_030641 | 1.29E-05 | 2.00914 |
| 2960872 | C6orf150 | NM_138441 | 7.79E-04 | 2.0075 |
| 3458133 | PRIM1 | NM_000946 | 4.83E-06 | 2.00639 |
| 3235516 | CAMK1D | NM_153498 | 2.16E-05 | 2.00531 |
| 2837479 | THG1L | NM_017872 | 4.40E-05 | 2.00352 |
| 2993727 | SNX10 | NM_013322 | 1.16E-03 | 2.00116 |
| 2591643 | COL5A2 | NM_000393 | 2.98E-05 | 2.0007 |
| 2708066 | KLHL6 | NM_130446 | 1.91E-06 | 1.99964 |
| 3360006 | RHOG | NM_001665 | 1.28E-04 | 1.99934 |
| 3695343 | RRAD | NM_001128850 | 6.14E-04 | 1.99729 |
| 2375706 | ATP2B4 | NM_001001396 | 1.22E-03 | 1.9968 |
| 2652410 | FNDC3B | NM_022763 | 2.51E-06 | 1.99588 |
| 3051655 | VOPP1 | NM_030796 | 1.80E-04 | 1.99526 |
| 2598099 | BARD1 | NM_000465 | 1.79E-05 | 1.99519 |
| 2442587 | CD247 | NM_198053 | 1.83E-04 | 1.99417 |
| 3472366 | SDS | NM_006843 | 2.54E-04 | 1.99102 |
| 2593464 | ANKRD44 | NM_153697 | 8.56E-05 | 1.98876 |
| 3870733 | LILRB2 | NM_005874 | 8.31E-04 | 1.98794 |
| 2796484 | CASP3 | NM_004346 | 1.15E-05 | 1.98781 |
| 3009959 | PTPN12 | NM_002835 | 2.87E-05 | 1.986 |
| 2560195 | PCGF1 | NM_032673 | 2.02E-06 | 1.98574 |
| 3777991 | KIAA0802 | BC040542 | 3.13E-04 | 1.9835 |
| 2949885 | GPSM3 | NM_022107 | 9.04E-05 | 1.98144 |
| 3967689 | STS | NM_000351 | 7.01E-04 | 1.98045 |
| 3619326 | PLCB2 | NM_004573 | 2.20E-04 | 1.97936 |
| 3841574 | LILRB1 | NM_006669 | 1.08E-05 | 1.97681 |
| 2603051 | SP110 | NM_080424 | 2.14E-05 | 1.97552 |
| 2949901 | NOTCH4 | NM_004557 | 6.27E-04 | 1.97374 |
| 3695424 | B3GNT9 | NM_033309 | 1.16E-04 | 1.97336 |
| 2950629 | TAPBP | NM_003190 | 1.62E-04 | 1.96891 |
| 2457261 | DUSP10 | NM_007207 | 4.06E-05 | 1.96707 |
| 2947975 | IFITM4P | NR_001590 | 3.81E-04 | 1.96689 |
| 2765865 | RELL1 | NM_001085400 | 3.16E-05 | 1.96501 |
| 2702724 | LXN | NM_020169 | 2.07E-05 | 1.96334 |
| 3728964 | PRR11 | NM_018304 | 9.16E-04 | 1.96144 |
| 2947073 | HIST1H1B | NM_005322 | 1.54E-04 | 1.95929 |
| 2881672 | TNIP1 | NM_006058 | 1.31E-03 | 1.95863 |
| 3743906 | TP53 | NM_000546 | 9.47E-05 | 1.95719 |
| 2370317 | MR1 | NM_001531 | 1.53E-04 | 1.95697 |
| 3874636 | SMOX | NM_175839 | 4.73E-04 | 1.95546 |
| 3591704 | WDR76 | NM_024908 | 4.26E-09 | 1.9547 |
| 3204648 | CD72 | NM_001782 | 6.13E-04 | 1.95454 |
| 2929699 | RAB32 | NM_006834 | 1.75E-06 | 1.95424 |
| 3441215 | C12orf4 | NM_020374 | 4.06E-07 | 1.95233 |
| 2379863 | CENPF | NM_016343 | 9.25E-04 | 1.95188 |
| 2588319 | KIAA1715 | NM_030650 | 3.32E-06 | 1.95032 |
| 3625271 | RAB27A | NM_004580 | 2.03E-04 | 1.95001 |
| 2871801 | FEM1C | NM_020177 | 1.03E-06 | 1.94773 |
| 3362263 | DENND5A | NM_015213 | 2.61E-05 | 1.94751 |
| 3853453 | RASAL3 | NM_022904 | 7.18E-05 | 1.94651 |
| 2938972 | SERPINB1 | NM_030666 | 1.90E-05 | 1.94448 |
| 2403215 | FGR | NM_001042747 | 2.82E-04 | 1.9436 |
| 2351063 | CSF1 | NM_000757 | 1.04E-03 | 1.94137 |
| 3925439 | HSPA13 | NM_006948 | 1.70E-06 | 1.94083 |
| 3371225 | CHST1 | NM_003654 | 1.46E-03 | 1.94062 |
| 2331771 | RLF | NM_012421 | 7.52E-06 | 1.94005 |
| 3942384 | MTP18 | NM_016498 | 9.22E-05 | 1.93902 |
| 3818547 | VAV1 | NM_005428 | 5.65E-05 | 1.93796 |
| 2577958 | DARS | NM_001349 | 8.82E-05 | 1.93656 |
| 3075932 | PARP12 | NM_022750 | 7.49E-05 | 1.93649 |
| 2476671 | RASGRP3 | NM_170672 | 1.47E-03 | 1.936 |
| 3151534 | ATAD2 | NM_014109 | 1.29E-05 | 1.93372 |
| 3815097 | FSTL3 | NM_005860 | 1.04E-03 | 1.93065 |
| 3744680 | PIK3R5 | NM_001142633 | 1.57E-04 | 1.9287 |
| 2709486 | RFC4 | NM_002916 | 5.88E-05 | 1.92826 |
| 3466369 | FGD6 | NM_018351 | 8.55E-04 | 1.92783 |
| 3772158 | TK1 | NM_003258 | 4.29E-04 | 1.92729 |
| 3344990 | PANX1 | NM_015368 | 1.05E-03 | 1.92241 |
| 3267036 | GRK5 | NM_005308 | 2.73E-04 | 1.92177 |
| 2862696 | ENC1 | NM_003633 | 5.96E-04 | 1.9216 |
| 3383227 | GAB2 | NM_080491 | 1.53E-03 | 1.91932 |
| 3990512 | SASH3 | NM_018990 | 6.91E-05 | 1.91923 |
| 2808290 | ZNF131 | NM_003432 | 1.34E-03 | 1.91677 |
| 3322775 | LDHA | NM_001165414 | 4.39E-07 | 1.91436 |
| 3735847 | SEPT9 | NM_006640 | 5.70E-06 | 1.91345 |
| 3410384 | C12orf35 | NM_018169 | 2.15E-04 | 1.91281 |
| 3094334 | GPR124 | NM_032777 | 1.35E-03 | 1.91024 |
| 3598165 | PLEKHO2 | NM_025201 | 5.04E-04 | 1.91021 |
| 3462843 | NAP1L1 | NM_004537 | 3.51E-04 | 1.90918 |
| 2359885 | SLC27A3 | NM_024330 | 1.21E-04 | 1.90626 |
| 3405032 | ETV6 | NM_001987 | 1.00E-03 | 1.90606 |
| 2821981 | FAM174A | NM_198507 | 1.30E-03 | 1.89762 |
| 3627076 | BNIP2 | NM_004330 | 1.98E-04 | 1.89739 |
| 2584207 | IFIH1 | NM_022168 | 1.71E-07 | 1.89739 |
| 2452977 | FAIM3 | NM_005449 | 8.02E-04 | 1.89546 |
| 4003895 | CXorf21 | NM_025159 | 1.19E-03 | 1.89392 |
| 3442812 | SLC2A14 | NM_153449 | 4.06E-04 | 1.89366 |
| 3490655 | CKAP2 | NM_018204 | 1.15E-04 | 1.89319 |
| 3936951 | SEPT5 | NM_002688 | 3.02E-04 | 1.88939 |
| 3010082 | PHTF2 | NM_001127358 | 1.40E-05 | 1.8885 |
| 2836856 | CNOT8 | NM_004779 | 3.80E-08 | 1.88574 |
| 2811656 | KIF2A | NM_001098511 | 7.60E-04 | 1.88544 |
| 2366798 | PRRX1 | NM_006902 | 1.07E-03 | 1.88497 |
| 3551566 | EVL | NM_016337 | 1.86E-04 | 1.88425 |
| 2334191 | PLK3 | NM_004073 | 8.12E-04 | 1.88176 |
| 2381368 | HLX | NM_021958 | 7.87E-06 | 1.88168 |
| 2562529 | ST3GAL5 | NM_003896 | 7.01E-05 | 1.87965 |
| 3417146 | CDK2 | NM_001798 | 4.00E-05 | 1.87863 |
| 3659858 | TMEM188 | NM_153261 | 1.21E-05 | 1.87824 |
| 2573786 | MKI67IP | NM_032390 | 5.03E-05 | 1.87567 |
| 2352228 | CAPZA1 | NM_006135 | 7.57E-06 | 1.87554 |
| 3275690 | IL15RA | NM_002189 | 1.07E-05 | 1.87459 |
| 2440549 | ARHGAP30 | NM_001025598 | 5.99E-05 | 1.8725 |
| 3153328 | FAM49B | BC017297 | 9.80E-06 | 1.86859 |
| 3918635 | IFNGR2 | NM_005534 | 3.21E-07 | 1.86754 |
| 3756046 | NR1D1 | NM_021724 | 4.05E-04 | 1.86555 |
| 2890859 | MGAT1 | NM_001114618 | 3.35E-07 | 1.86485 |
| 2879312 | NR3C1 | NM_000176 | 8.21E-05 | 1.86354 |
| 3536336 | CDKN3 | NM_005192 | 5.57E-04 | 1.86316 |
| 3839619 | SIGLEC9 | NM_014441 | 1.08E-04 | 1.85855 |
| 2990464 | ARL4A | NM_212460 | 7.85E-05 | 1.85811 |
| 2889916 | ADAMTS2 | NM_014244 | 6.05E-05 | 1.85648 |
| 2592356 | STAT4 | NM_003151 | 1.37E-03 | 1.85609 |
| 3886704 | STK4 | NM_006282 | 9.56E-07 | 1.85577 |
| 2694001 | MGLL | NM_007283 | 5.74E-05 | 1.85502 |
| 3940631 | ADRBK2 | NM_005160 | 6.63E-04 | 1.85499 |
| 2887633 | BOD1 | NM_138369 | 1.76E-06 | 1.85332 |
| 3822723 | PKN1 | NM_002741 | 5.04E-04 | 1.852 |
| 3388631 | TMEM123 | NM_052932 | 2.51E-05 | 1.8511 |
| 3611684 | LRRK1 | NM_024652 | 2.51E-04 | 1.85061 |
| 3044904 | KBTBD2 | NM_015483 | 9.90E-05 | 1.8505 |
| 2982076 | TAGAP | NM_054114 | 1.01E-04 | 1.84977 |
| 3655665 | MAZ | NM_001042539 | 9.81E-05 | 1.84923 |
| 3182199 | C9orf30 | AY598327 | 4.40E-04 | 1.84859 |
| 2800503 | PAPD7 | NM_006999 | 1.42E-04 | 1.8463 |
| 2563654 | EIF2AK3 | NM_004836 | 2.92E-06 | 1.84549 |
| 3130850 | RNF122 | NM_024787 | 2.70E-04 | 1.84544 |
| 3470549 | CORO1C | NM_014325 | 6.08E-04 | 1.84464 |
| 2874794 | RAPGEF6 | NM_001164386 | 5.05E-07 | 1.83802 |
| 2719361 | CPEB2 | NM_182485 | 2.94E-04 | 1.83545 |
| 2879509 | YIPF5 | NM_001024947 | 3.53E-07 | 1.83531 |
| 2853768 | NUP155 | NM_153485 | 9.08E-05 | 1.83382 |
| 2384401 | RHOU | NM_021205 | 1.73E-05 | 1.83282 |
| 3581442 | JAG2 | NM_002226 | 1.40E-04 | 1.83281 |
| 3504617 | SKA3 | NM_145061 | 2.72E-05 | 1.8303 |
| 3335338 | FAM89B | NM_001098785 | 3.33E-04 | 1.83027 |
| 3854627 | JAK3 | NM_000215 | 2.46E-04 | 1.82938 |
| 3339261 | IL18BP | NM_173042 | 1.85E-04 | 1.82937 |
| 3403092 | PTPN6 | NM_080549 | 4.18E-05 | 1.82898 |
| 3431892 | SH2B3 | NM_005475 | 1.18E-04 | 1.82821 |
| 2707876 | LAMP3 | NM_014398 | 9.79E-05 | 1.8273 |
| 3536706 | LGALS3 | NR_003225 | 8.33E-04 | 1.82624 |
| 3647504 | PMM2 | NM_000303 | 1.46E-03 | 1.82536 |
| 2695941 | TOPBP1 | NM_007027 | 5.45E-06 | 1.82521 |
| 3262490 | C10orf78 | NM_145247 | 2.46E-04 | 1.82307 |
| 2610707 | HRH1 | NM_001098213 | 1.34E-03 | 1.82295 |
| 3770361 | CD300LF | NM_139018 | 3.40E-05 | 1.82284 |
| 2779897 | MANBA | NM_005908 | 1.85E-04 | 1.81939 |
| 2955076 | NFKBIE | NM_004556 | 1.08E-03 | 1.81913 |
| 2899756 | HIST1H2AG | NM_021064 | 1.75E-04 | 1.81864 |
| 3756319 | CCR7 | NM_001838 | 1.50E-03 | 1.81693 |
| 3865344 | PPP1R13L | NM_006663 | 5.81E-06 | 1.81681 |
| 2531310 | SP140L | NM_138402 | 4.51E-04 | 1.81603 |
| 2997097 | SEPT7 | NM_001788 | 1.54E-04 | 1.8141 |
| 3607927 | SEMA4B | NM_020210 | 8.92E-04 | 1.814 |
| 2993590 | NFE2L3 | NM_004289 | 1.12E-03 | 1.81355 |
| 3335465 | SIPA1 | NM_153253 | 4.98E-04 | 1.81144 |
| 2842570 | FAF2 | NM_014613 | 4.70E-06 | 1.80837 |
| 2480992 | MSH2 | NM_000251 | 3.17E-04 | 1.80632 |
| 2780099 | NHEDC2 | NM_178833 | 9.51E-04 | 1.80552 |
| 3830002 | GRAMD1A | NM_020895 | 8.75E-07 | 1.80482 |
| 3676763 | ABCA3 | NM_001089 | 7.68E-04 | 1.80236 |
| 2841184 | ERGIC1 | NM_001031711 | 4.33E-08 | 1.80231 |
| 2834093 | TCERG1 | NM_006706 | 4.86E-06 | 1.80097 |
| 3352070 | CBL | NM_005188 | 1.24E-05 | 1.80037 |
| 2460189 | PGBD5 | NM_024554 | 1.21E-03 | 1.80021 |
| 3990727 | RAB33A | NM_004794 | 7.41E-06 | 1.79949 |
| 2899340 | BTN2A2 | NM_006995 | 2.33E-05 | 1.79803 |
| 2440700 | ADAMTS4 | NM_005099 | 9.86E-04 | 1.79739 |
| 3767339 | GNA13 | NM_006572 | 3.76E-06 | 1.79679 |
| 3865503 | GPR4 | NM_005282 | 1.08E-03 | 1.79528 |
| 3322251 | NUCB2 | NM_005013 | 4.10E-06 | 1.79482 |
| 3413950 | SPATS2 | NM_023071 | 1.82E-04 | 1.79366 |
| 2836451 | MFAP3 | NR_024152 | 3.23E-05 | 1.79273 |
| 2554448 | FANCL | NM_001114636 | 1.79E-05 | 1.79263 |
| 3250278 | HK1 | NM_033500 | 6.04E-04 | 1.79123 |
| 2644155 | NCK1 | NM_006153 | 1.85E-05 | 1.79011 |
| 3846900 | C19orf10 | NM_019107 | 5.70E-04 | 1.78925 |
| 2409004 | LEPRE1 | NM_022356 | 3.34E-04 | 1.78789 |
| 3404530 | CLEC12A | NM_138337 | 1.41E-03 | 1.7876 |
| 3836317 | VASP | NM_003370 | 1.24E-07 | 1.7863 |
| 3936009 | IL17RA | NM_014339 | 1.03E-06 | 1.7826 |
| 2881521 | RBM22 | NM_018047 | 4.93E-07 | 1.78188 |
| 2832533 | PCDHGC5 | NM_018929 | 3.41E-04 | 1.78076 |
| 2784027 | ANXA5 | NM_001154 | 1.15E-04 | 1.77586 |
| 3960782 | JOSD1 | NM_014876 | 3.54E-04 | 1.77576 |
| 2847264 | MED10 | NM_032286 | 2.17E-05 | 1.77494 |
| 2520225 | NAB1 | NM_005966 | 4.05E-04 | 1.76833 |
| 3851545 | MAN2B1 | NM_000528 | 1.44E-04 | 1.76762 |
| 2878246 | PFDN1 | NM_002622 | 1.46E-03 | 1.76713 |
| 2600218 | CHPF | NM_024536 | 5.83E-05 | 1.76692 |
| 4005627 | CXorf38 | NM_144970 | 1.76E-05 | 1.76681 |
| 3752002 | CRLF3 | NM_015986 | 3.64E-04 | 1.76562 |
| 3681705 | RRN3 | NM_018427 | 1.26E-03 | 1.76419 |
| 3151607 | FBXO32 | NM_058229 | 1.22E-03 | 1.76365 |
| 3643047 | RAB40C | NM_001172663 | 7.07E-04 | 1.76193 |
| 3589141 | SPRED1 | NM_152594 | 6.78E-04 | 1.76123 |
| 2831284 | UBE2D2 | NM_181838 | 5.66E-06 | 1.75688 |
| 3202421 | C9orf72 | NM_018325 | 1.89E-04 | 1.75671 |
| 3869650 | ZNF83 | NM_001105549 | 9.85E-05 | 1.75601 |
| 2829416 | SEC24A | NM_021982 | 1.97E-04 | 1.75536 |
| 3869396 | ZNF841 | NM_001136499 | 3.03E-04 | 1.7541 |
| 3127199 | DOK2 | NM_003974 | 5.22E-04 | 1.75001 |
| 2758043 | MFSD10 | NM_001146069 | 4.61E-05 | 1.74916 |
| 2328841 | LCK | NM_005356 | 7.06E-04 | 1.74765 |
| 3696057 | SLC12A4 | NM_005072 | 6.76E-04 | 1.74657 |
| 3607510 | FANCI | NM_001113378 | 1.25E-04 | 1.74558 |
| 3137530 | ASPH | NM_004318 | 1.10E-04 | 1.74532 |
| 3734413 | RAB37 | NM_175738 | 4.20E-05 | 1.74515 |
| 2510713 | FMNL2 | NM_052905 | 1.01E-04 | 1.74459 |
| 3028217 | TRBV27 | AK093303 | 1.44E-03 | 1.74275 |
| 2608725 | BHLHE40 | NM_003670 | 1.03E-03 | 1.73984 |
| 2362157 | CD1D | NM_001766 | 1.99E-05 | 1.73913 |
| 3608113 | IQGAP1 | NM_003870 | 2.92E-05 | 1.7372 |
| 3870054 | ZNF160 | NM_001102603 | 3.40E-07 | 1.73663 |
| 2899768 | HIST1H4I | NM_003495 | 8.37E-04 | 1.73612 |
| 2771718 | UBA6 | NM_018227 | 2.01E-04 | 1.73323 |
| 3818468 | TNFSF9 | NM_003811 | 1.18E-04 | 1.73068 |
| 3118651 | DENND3 | NM_014957 | 1.31E-03 | 1.72984 |
| 2899437 | BTN2A1 | NM_078476 | 3.64E-05 | 1.72682 |
| 2511432 | GPD2 | NM_001083112 | 1.20E-03 | 1.72675 |
| 2322389 | NECAP2 | NM_018090 | 2.79E-04 | 1.72225 |
| 3854954 | LRRC25 | NM_145256 | 7.53E-04 | 1.72134 |
| 3866958 | CARD8 | NM_014959 | 2.46E-04 | 1.71894 |
| 3563395 | POLE2 | NM_002692 | 2.68E-05 | 1.71534 |
| 3908831 | ZNFX1 | NM_021035 | 9.19E-05 | 1.71391 |
| 3855596 | NR2C2AP | NM_176880 | 2.08E-05 | 1.71283 |
| 3850261 | ICAM3 | NM_002162 | 1.78E-04 | 1.71282 |
| 2494484 | NCAPH | NM_015341 | 7.77E-04 | 1.71262 |
| 3802980 | DSC2 | NM_004949 | 1.17E-03 | 1.71185 |
| 3821908 | RNASEH2A | NM_006397 | 1.35E-04 | 1.71106 |
| 3284188 | ITGB1 | NM_002211 | 9.23E-05 | 1.71097 |
| 3043264 | JAZF1 | NM_175061 | 1.14E-03 | 1.70972 |
| 2531377 | SP100 | NM_003113 | 5.18E-05 | 1.70908 |
| 3776193 | SMCHD1 | NM_015295 | 6.12E-05 | 1.70902 |
| 3128372 | KCTD9 | NM_017634 | 1.37E-03 | 1.7073 |
| 2329077 | S100PBP | NM_022753 | 4.16E-04 | 1.7071 |
| 2437801 | ARHGEF2 | NM_001162383 | 8.16E-04 | 1.70585 |
| 3765580 | BRIP1 | NM_032043 | 6.21E-04 | 1.7051 |
| 3221916 | AKNA | NM_030767 | 1.56E-04 | 1.70488 |
| 2843804 | ZNF354B | NM_058230 | 4.13E-04 | 1.70451 |
| 4035833 | CD24 | NM_013230 | 1.31E-03 | 1.70409 |
| 2818454 | XRCC4 | NM_022550 | 6.60E-05 | 1.70222 |
| 3896370 | GPCPD1 | NM_019593 | 6.95E-04 | 1.70051 |
| 3072368 | ZC3HC1 | NM_016478 | 5.21E-04 | 1.70047 |
| 3151401 | DERL1 | NM_024295 | 4.72E-05 | 1.70046 |
| 3837132 | SAE1 | NR_027280 | 2.78E-04 | 1.69976 |
| 2752560 | SPCS3 | NM_021928 | 3.15E-04 | 1.69857 |
| 3457752 | STAT2 | NM_005419 | 3.46E-04 | 1.69818 |
| 2802398 | TRIO | NM_007118 | 4.09E-04 | 1.69614 |
| 2882897 | GEMIN5 | NM_015465 | 8.27E-06 | 1.69561 |
| 3817698 | UHRF1 | NM_001048201 | 1.33E-04 | 1.69508 |
| 2510884 | ARL6IP6 | NM_152522 | 6.95E-05 | 1.69318 |
| 2944068 | DEK | NM_003472 | 7.54E-05 | 1.69307 |
| 3126739 | LZTS1 | NM_021020 | 3.63E-05 | 1.69259 |
| 2648141 | MBNL1 | NM_021038 | 7.32E-07 | 1.69223 |
| 3201437 | CDKN2A | NM_058197 | 6.21E-07 | 1.68929 |
| 3261643 | NFKB2 | NM_001077494 | 7.86E-05 | 1.68897 |
| 2522728 | CASP8 | NM_001228 | 7.29E-05 | 1.68854 |
| 3558359 | GZMH | NM_033423 | 1.43E-03 | 1.68827 |
| 3426828 | VEZT | NM_017599 | 1.57E-04 | 1.6882 |
| 2863049 | POC5 | NM_001099271 | 2.93E-04 | 1.68757 |
| 3352485 | TMEM136 | NM_174926 | 8.12E-04 | 1.68626 |
| 3895614 | SIGLEC1 | NM_023068 | 6.40E-04 | 1.6851 |
| 2369557 | SOAT1 | NM_003101 | 4.87E-04 | 1.6829 |
| 3620515 | TMEM87A | NM_015497 | 6.45E-05 | 1.68122 |
| 3421177 | NUP107 | NM_020401 | 3.84E-04 | 1.68115 |
| 3850069 | DNMT1 | NM_001130823 | 9.81E-06 | 1.68053 |
| 3145107 | CCNE2 | NM_057749 | 1.72E-04 | 1.67963 |
| 2834957 | AFAP1L1 | NM_152406 | 2.46E-05 | 1.67883 |
| 2785282 | SCLT1 | NM_144643 | 5.96E-05 | 1.67849 |
| 3082248 | ESYT2 | NM_020728 | 8.72E-06 | 1.67799 |
| 2888519 | UIMC1 | NM_016290 | 3.12E-05 | 1.67519 |
| 3060051 | C7orf23 | NM_024315 | 8.87E-04 | 1.67218 |
| 3649811 | NDE1 | NM_001143979 | 4.23E-06 | 1.67092 |
| 3738901 | NARF | NM_001038618 | 8.29E-05 | 1.67058 |
| 3747236 | C17orf76 | NM_001113567 | 1.19E-03 | 1.67017 |
| 2824872 | AP3S1 | NM_001284 | 7.86E-04 | 1.66955 |
| 2924253 | RNF217 | NM_152553 | 1.44E-04 | 1.6681 |
| 2880932 | CSNK1A1 | NM_001025105 | 1.25E-06 | 1.6677 |
| 2871717 | CCDC112 | NM_001040440 | 1.79E-04 | 1.66664 |
| 3829768 | UBA2 | NM_005499 | 8.26E-04 | 1.66639 |
| 2577896 | MCM6 | NM_005915 | 2.86E-05 | 1.66633 |
| 2361279 | LMNA | NM_170707 | 1.77E-04 | 1.66621 |
| 2687979 | KIAA1524 | NM_020890 | 3.90E-04 | 1.66236 |
| 3846507 | DAPK3 | NM_001348 | 1.60E-04 | 1.66204 |
| 2784687 | ANKRD50 | NM_020337 | 5.03E-04 | 1.66175 |
| 3560617 | SNX6 | NM_021249 | 3.67E-04 | 1.66162 |
| 3830051 | SCN1B | NM_001037 | 1.01E-03 | 1.66026 |
| 3687308 | KCTD13 | NM_178863 | 4.17E-06 | 1.65942 |
| 3315607 | ATHL1 | NM_025092 | 9.97E-04 | 1.65922 |
| 3475679 | ZCCHC8 | NM_017612 | 9.41E-04 | 1.65798 |
| 2501697 | ACTR3 | NM_005721 | 3.04E-06 | 1.65647 |
| 3619116 | GPR176 | NM_007223 | 8.04E-04 | 1.65595 |
| 2954771 | GTPBP2 | NM_019096 | 2.64E-04 | 1.65573 |
| 3045004 | NT5C3 | NR_029372 | 2.12E-04 | 1.65394 |
| 2640855 | MCM2 | NM_004526 | 2.19E-05 | 1.65381 |
| 3042610 | SKAP2 | NM_003930 | 2.82E-06 | 1.65342 |
| 2510485 | RIF1 | NM_018151 | 2.20E-04 | 1.65331 |
| 3822551 | IL27RA | NM_004843 | 1.12E-03 | 1.65321 |
| 3589697 | BUB1B | NM_001211 | 3.96E-04 | 1.65312 |
| 2741768 | EXOSC9 | NM_001034194 | 8.49E-04 | 1.65281 |
| 2687255 | CBLB | NM_170662 | 1.20E-03 | 1.65224 |
| 3638188 | HAPLN3 | NM_178232 | 8.87E-04 | 1.65166 |
| 2639309 | SEC22A | NM_012430 | 3.49E-04 | 1.6512 |
| 3375735 | AHNAK | NM_001620 | 9.33E-04 | 1.65065 |
| 3587457 | ARHGAP11A | NM_014783 | 1.49E-04 | 1.65001 |
| 3996467 | PLXNA3 | NM_017514 | 2.37E-07 | 1.64777 |
| 3659966 | ADCY7 | NM_001114 | 1.22E-03 | 1.64586 |
| 3309602 | RGS10 | NM_001005339 | 4.49E-05 | 1.64556 |
| 2723710 | PGM2 | NM_018290 | 1.16E-03 | 1.64535 |
| 2886679 | KCNMB1 | NM_004137 | 6.35E-04 | 1.64517 |
| 2599433 | USP37 | NM_020935 | 8.61E-05 | 1.6451 |
| 3675308 | FBXL16 | NM_153350 | 4.44E-04 | 1.64476 |
| 3136782 | NSMAF | NM_003580 | 5.62E-05 | 1.64452 |
| 3565571 | WDHD1 | NM_007086 | 7.53E-05 | 1.64414 |
| 3940099 | ADORA2A | NM_000675 | 3.25E-04 | 1.64333 |
| 2714230 | PCGF3 | NM_006315 | 1.26E-05 | 1.64295 |
| 2401994 | RUNX3 | NM_001031680 | 1.09E-03 | 1.64292 |
| 2732942 | BMP2K | NM_198892 | 5.76E-05 | 1.6423 |
| 3919952 | MORC3 | NM_015358 | 6.82E-05 | 1.64207 |
| 3179359 | CENPP | NM_001012267 | 2.37E-05 | 1.63996 |
| 2873168 | CEP120 | NM_153223 | 2.69E-06 | 1.6396 |
| 2411228 | STIL | NM_001048166 | 6.08E-04 | 1.63717 |
| 3866135 | PRKD2 | NM_016457 | 8.68E-06 | 1.63466 |
| 2881554 | DCTN4 | NM_001135643 | 3.69E-05 | 1.63169 |
| 3021123 | ING3 | NM_019071 | 1.50E-03 | 1.63165 |
| 3489481 | PHF11 | NM_001040443 | 1.40E-03 | 1.63095 |
| 3432754 | PLBD2 | NM_173542 | 6.55E-04 | 1.62953 |
| 3873338 | FAM110A | NM_001042353 | 9.24E-05 | 1.62934 |
| 3944826 | SH3BP1 | NM_018957 | 3.93E-04 | 1.62857 |
| 3866845 | PLA2G4C | NM_001159323 | 1.32E-04 | 1.62834 |
| 3034449 | WDR60 | NM_018051 | 7.95E-05 | 1.62781 |
| 3951927 | BID | NM_197966 | 1.27E-03 | 1.62741 |
| 2871821 | TMED7 | NM_181836 | 5.14E-05 | 1.62692 |
| 3922664 | SLC37A1 | NM_018964 | 6.42E-04 | 1.62537 |
| 3976519 | RBM3 | NM_006743 | 1.96E-04 | 1.62238 |
| 3846860 | SEMA6B | NM_032108 | 1.82E-04 | 1.62114 |
| 2712040 | ACAP2 | NM_012287 | 7.74E-05 | 1.62084 |
| 3082181 | NCAPG2 | NM_017760 | 1.48E-03 | 1.62068 |
| 3623683 | GABPB1 | NM_005254 | 3.71E-04 | 1.61996 |
| 3345774 | JRKL | NM_003772 | 4.94E-05 | 1.61929 |
| 3642815 | NME4 | NM_005009 | 6.48E-05 | 1.6192 |
| 2879927 | LARS | NM_020117 | 2.00E-04 | 1.61798 |
| 2446047 | ABL2 | NM_007314 | 1.09E-05 | 1.61733 |
| 3496916 | GPR180 | NM_180989 | 5.58E-04 | 1.61691 |
| 2489440 | DOK1 | NM_001381 | 2.20E-04 | 1.61663 |
| 2483451 | VRK2 | NM_001130483 | 4.35E-04 | 1.61598 |
| 2687739 | CD47 | NM_001777 | 7.65E-04 | 1.61487 |
| 2831932 | IK | NM_006083 | 2.89E-04 | 1.61405 |
| 3976766 | WAS | NM_000377 | 1.28E-03 | 1.61349 |
| 2706297 | TBL1XR1 | NM_024665 | 1.12E-04 | 1.61276 |
| 2321779 | EFHD2 | NM_024329 | 1.43E-04 | 1.61189 |
| 3904747 | RBL1 | NM_002895 | 7.99E-04 | 1.60873 |
| 3845681 | MOBKL2A | NM_130807 | 3.70E-04 | 1.60827 |
| 3944922 | TRIOBP | NM_001039141 | 5.10E-04 | 1.60745 |
| 3348765 | HSPB2 | NM_001541 | 1.43E-03 | 1.60701 |
| 4000704 | AP1S2 | NM_003916 | 4.11E-04 | 1.60684 |
| 3386814 | TAF1D | NM_024116 | 4.14E-04 | 1.60608 |
| 3392871 | ZNF259 | NM_003904 | 5.17E-04 | 1.60535 |
| 3132016 | FGFR1 | NM_001174064 | 7.56E-04 | 1.60448 |
| 2915491 | CYB5R4 | NM_016230 | 2.29E-04 | 1.60433 |
| 3960440 | TMEM184B | NM_012264 | 2.49E-04 | 1.60288 |
| 2327542 | TRNAU1AP | NR_003109 | 1.71E-04 | 1.60265 |
| 3031967 | CHPF2 | NM_019015 | 1.39E-03 | 1.60138 |
| 3565663 | DLGAP5 | NM_001146015 | 1.09E-03 | 1.60138 |
| 3147020 | ZNF706 | NM_001042510 | 4.19E-04 | 1.60102 |
| 3032017 | NUB1 | NM_016118 | 1.16E-04 | 1.60018 |
| 3611744 | LRRK1 | NM_024652 | 2.83E-04 | 1.59987 |
| 3759704 | MAP3K14 | NM_003954 | 8.15E-04 | 1.59977 |
| 3227696 | RAPGEF1 | NM_198679 | 1.27E-04 | 1.59973 |
| 3452970 | SENP1 | NM_014554 | 4.90E-04 | 1.59958 |
| 2884647 | C5orf54 | NM_022090 | 5.28E-04 | 1.59887 |
| 4025339 | IDS | NM_000202 | 5.26E-07 | 1.59869 |
| 3825013 | SSBP4 | NM_032627 | 1.47E-03 | 1.59832 |
| 3810472 | LMAN1 | NM_005570 | 2.43E-05 | 1.59804 |
| 3645565 | THOC6 | NM_024339 | 9.84E-04 | 1.59774 |
| 3948461 | NUP50 | NM_007172 | 8.95E-04 | 1.5971 |
| 3992148 | DDX26B | NM_182540 | 5.15E-04 | 1.59709 |
| 3651018 | CP110 | NM_014711 | 5.35E-04 | 1.59691 |
| 3905875 | MAFB | NM_005461 | 4.15E-04 | 1.59647 |
| 3227574 | FAM78A | NM_033387 | 9.22E-04 | 1.59567 |
| 3875423 | BMP2 | NM_001200 | 5.21E-04 | 1.59311 |
| 3016692 | PRKRIP1 | NM_024653 | 1.09E-03 | 1.5923 |
| 2494709 | CNNM4 | NM_020184 | 6.48E-05 | 1.59187 |
| 2416522 | JAK1 | NM_002227 | 7.00E-04 | 1.59142 |
| 2397948 | EPHA2 | NM_004431 | 5.56E-04 | 1.58986 |
| 2527856 | RQCD1 | NM_005444 | 1.01E-06 | 1.58957 |
| 2339414 | USP1 | NM_003368 | 4.27E-04 | 1.58937 |
| 2620641 | LIMD1 | NM_014240 | 7.10E-05 | 1.58887 |
| 2502300 | DDX18 | NM_006773 | 4.51E-04 | 1.58817 |
| 2812273 | PPWD1 | NM_015342 | 1.09E-03 | 1.58736 |
| 3451670 | PUS7L | NM_001098615 | 4.63E-05 | 1.5871 |
| 3484641 | BRCA2 | NM_000059 | 4.91E-06 | 1.58639 |
| 3022465 | SND1 | NM_014390 | 2.86E-05 | 1.58627 |
| 2871923 | ATG12 | NR_033363 | 1.32E-04 | 1.58585 |
| 3427876 | APAF1 | NM_181861 | 4.12E-05 | 1.58247 |
| 3009441 | ZP3 | NM_001110354 | 1.15E-03 | 1.58186 |
| 3358174 | IRF7 | NM_004031 | 2.23E-04 | 1.58181 |
| 3870494 | TFPT | NM_013342 | 1.04E-04 | 1.58157 |
| 2999710 | DBNL | NM_014063 | 2.05E-05 | 1.58064 |
| 3258625 | GPR120 | NM_181745 | 1.34E-03 | 1.5798 |
| 3601840 | CSK | NM_004383 | 3.48E-04 | 1.57923 |
| 2884658 | SLU7 | NM_006425 | 1.25E-04 | 1.57792 |
| 2633930 | PCNP | NM_020357 | 8.37E-04 | 1.57706 |
| 3835966 | RELB | NM_006509 | 4.25E-05 | 1.5769 |
| 3468103 | GNPTAB | NM_024312 | 2.92E-05 | 1.57613 |
| 3960005 | C1QTNF6 | NM_031910 | 1.19E-03 | 1.57486 |
| 3597476 | RAB8B | NM_016530 | 1.43E-03 | 1.57454 |
| 3432267 | TRAFD1 | NM_001143906 | 2.22E-05 | 1.57443 |
| 3107661 | INTS8 | NM_017864 | 1.99E-04 | 1.57296 |
| 3936913 | CDC45 | NM_001178010 | 3.11E-04 | 1.57175 |
| 2951859 | ETV7 | NM_016135 | 6.78E-04 | 1.57167 |
| 3595979 | CCNB2 | NM_004701 | 7.76E-04 | 1.57023 |
| 3939545 | MIF | NM_002415 | 7.68E-05 | 1.57012 |
| 3655920 | ALDOA | NM_000034 | 4.05E-06 | 1.56925 |
| 3825141 | C19orf50 | NM_001171948 | 3.29E-04 | 1.56771 |
| 2407191 | GNL2 | NM_013285 | 2.09E-05 | 1.56607 |
| 2589255 | FKBP7 | NM_181342 | 1.32E-04 | 1.56604 |
| 3240012 | MASTL | NM_001172303 | 5.42E-05 | 1.56603 |
| 2863535 | WDR41 | NM_018268 | 1.22E-03 | 1.56566 |
| 3168508 | MELK | NM_014791 | 2.37E-04 | 1.56498 |
| 4013730 | BRWD3 | NM_153252 | 2.58E-04 | 1.56455 |
| 3719515 | DUSP14 | NM_007026 | 2.89E-04 | 1.56438 |
| 2481271 | FOXN2 | NM_002158 | 7.78E-04 | 1.56273 |
| 3419807 | XPOT | NM_007235 | 1.67E-04 | 1.56126 |
| 3687752 | SEPT1 | NM_052838 | 6.77E-04 | 1.55996 |
| 3896200 | PCNA | NM_002592 | 8.69E-04 | 1.55922 |
| 3868183 | NUP62 | NM_153719 | 1.81E-06 | 1.55909 |
| 2365872 | RCSD1 | NM_052862 | 3.07E-04 | 1.55864 |
| 3855868 | GMIP | NM_016573 | 2.44E-04 | 1.55859 |
| 2714955 | TACC3 | NM_006342 | 2.35E-04 | 1.55821 |
| 2434776 | CDC42SE1 | NM_001038707 | 5.92E-04 | 1.55722 |
| 3456049 | ITGB7 | NM_000889 | 4.02E-05 | 1.5553 |
| 2454485 | LPGAT1 | NM_014873 | 2.37E-04 | 1.55401 |
| 3458551 | ARHGAP9 | NM_032496 | 7.10E-04 | 1.55245 |
| 3186207 | C9orf91 | NM_153045 | 2.59E-05 | 1.55244 |
| 3660213 | CYLD | NM_015247 | 1.10E-05 | 1.55116 |
| 3869761 | ZNF600 | NM_198457 | 2.10E-04 | 1.55037 |
| 3561952 | SEC23A | NM_006364 | 8.60E-04 | 1.54979 |
| 2737717 | NFKB1 | NM_003998 | 7.71E-04 | 1.54898 |
| 2973856 | SAMD3 | NM_001017373 | 1.39E-03 | 1.54832 |
| 3630099 | TIPIN | NM_017858 | 9.80E-04 | 1.5483 |
| 3839142 | ZNF473 | NM_015428 | 2.03E-04 | 1.54817 |
| 3646277 | MGRN1 | NM_001142290 | 1.43E-03 | 1.54662 |
| 2990043 | PHF14 | NM_014660 | 7.31E-05 | 1.54451 |
| 3536434 | SAMD4A | NM_015589 | 1.62E-05 | 1.54272 |
| 4007617 | PIM2 | NM_006875 | 3.06E-04 | 1.5415 |
| 3476741 | UBC | NM_021009 | 4.80E-05 | 1.54035 |
| 3452743 | HDAC7 | NM_015401 | 1.29E-03 | 1.54024 |
| 3969455 | OFD1 | NM_003611 | 9.12E-04 | 1.53977 |
| 2744674 | RAB33B | NM_031296 | 4.47E-04 | 1.53923 |
| 3376560 | ATL3 | NM_015459 | 2.16E-05 | 1.53764 |
| 2622638 | GNAI2 | NM_002070 | 8.56E-04 | 1.53693 |
| 2883380 | MED7 | NM_001100816 | 3.17E-04 | 1.53693 |
| 3645204 | KCTD5 | NM_018992 | 4.71E-04 | 1.53665 |
| 3471819 | NAA25 | NM_024953 | 2.90E-06 | 1.53659 |
| 2782230 | TIFA | NM_052864 | 7.92E-04 | 1.53631 |
| 3667281 | SF3B3 | NM_012426 | 2.07E-06 | 1.53485 |
| 2632036 | ZNF654 | NM_018293 | 1.27E-03 | 1.53339 |
| 3204019 | C9orf25 | NM_147202 | 1.40E-06 | 1.53281 |
| 2744597 | NAA15 | NM_057175 | 5.11E-06 | 1.53176 |
| 3695541 | FHOD1 | NM_013241 | 1.20E-04 | 1.53059 |
| 3707214 | PLD2 | NM_002663 | 1.42E-03 | 1.52784 |
| 3873699 | STK35 | NM_080836 | 1.07E-03 | 1.52601 |
| 3752437 | UTP6 | NM_018428 | 2.65E-05 | 1.52597 |
| 3944210 | RASD2 | NM_014310 | 3.02E-05 | 1.5256 |
| 3230697 | NPDC1 | NM_015392 | 2.24E-05 | 1.52412 |
| 3881282 | HM13 | NM_178580 | 3.48E-04 | 1.52313 |
| 3029030 | CASP2 | NM_032982 | 2.88E-05 | 1.52254 |
| 3829687 | GPI | NM_000175 | 1.78E-04 | 1.52192 |
| 3564997 | DDHD1 | NM_001160148 | 4.81E-05 | 1.52071 |
| 2965739 | C6orf167 | NM_198468 | 1.43E-03 | 1.51991 |
| 3544605 | BATF | NM_006399 | 5.81E-04 | 1.5196 |
| 2450855 | PHLDA3 | NM_012396 | 3.23E-05 | 1.5191 |
| 3161167 | KIAA1432 | NM_020829 | 2.43E-04 | 1.519 |
| 3293887 | ASCC1 | NM_015947 | 7.84E-04 | 1.51894 |
| 3371673 | ARHGAP1 | NM_004308 | 7.84E-04 | 1.51884 |
| 2902348 | MICB | NM_005931 | 1.17E-03 | 1.51777 |
| 3833443 | PLD3 | NM_012268 | 1.69E-04 | 1.51729 |
| 3195174 | MAN1B1 | NM_016219 | 4.26E-04 | 1.51648 |
| 3791341 | ZCCHC2 | NM_017742 | 9.90E-06 | 1.51539 |
| 3326826 | FJX1 | NM_014344 | 9.77E-04 | 1.5141 |
| 2648305 | P2RY1 | NM_002563 | 5.79E-04 | 1.51308 |
| 2638059 | ADPRH | NM_001125 | 1.12E-04 | 1.51302 |
| 3434760 | P2RX4 | NM_002560 | 1.28E-03 | 1.51291 |
| 3181193 | TDRD7 | NM_014290 | 1.64E-06 | 1.51175 |
| 2765590 | ARAP2 | NM_015230 | 2.72E-04 | 1.50867 |
| 3918574 | IFNAR1 | NM_000629 | 3.96E-04 | 1.50838 |
| 3717452 | SH3GL1P1 | NR_033412 | 1.01E-04 | 1.50807 |
| 3765642 | INTS2 | NR_026641 | 6.99E-06 | 1.50707 |
| 3844978 | SBNO2 | NM_014963 | 1.09E-04 | 1.50659 |
| 3815493 | HMHA1 | NM_012292 | 2.80E-04 | 1.50565 |
| 2512601 | TANK | NM_004180 | 2.67E-04 | 1.50532 |
| 2900074 | HIST1H2BN | NM_003520 | 1.46E-04 | 1.50484 |
| 3933331 | C2CD2 | NM_015500 | 9.69E-04 | 1.50331 |
| 4054481 | GABRD | NM_000815 | 2.19E-04 | 1.50312 |
| 3892941 | OGFR | NM_007346 | 1.10E-03 | 1.50244 |
| 3401259 | TEAD4 | NM_003213 | 1.26E-03 | 1.50134 |
| 3375894 | EML3 | NM_153265 | 9.92E-04 | 1.50096 |
| 3863046 | B9D2 | NM_030578 | 6.75E-04 | 1.50052 |
| 2685908 | CLDND1 | NM_001040199 | 4.74E-04 | 1.50022 |
| 3037344 | DAGLB | NM_139179 | 3.86E-04 | 1.49973 |
| 2385343 | DISC1 | NM_001164537 | 1.63E-04 | 1.49828 |
| 2426559 | SLC25A24 | NM_213651 | 4.53E-04 | 1.49646 |
| 3471327 | HVCN1 | NM_001040107 | 1.21E-04 | 1.49578 |
| 3602116 | C15orf39 | NM_015492 | 3.35E-04 | 1.49516 |
| 3655806 | TMEM219 | NM_001083613 | 1.36E-03 | 1.49476 |
| 3607275 | ISG20 | NM_002201 | 6.58E-04 | 1.49256 |
| 3387771 | CCDC82 | NM_024725 | 8.99E-05 | 1.49164 |
| 3511031 | ELF1 | NM_172373 | 5.68E-04 | 1.49152 |
| 4052881 | FAM72D | AB096683 | 4.86E-04 | 1.49043 |
| 2779486 | H2AFZ | NM_002106 | 1.13E-03 | 1.49012 |
| 3894995 | SNRPB | NM_003091 | 1.14E-03 | 1.4892 |
| 3371339 | PHF21A | NM_001101802 | 1.04E-03 | 1.48904 |
| 2482230 | ERLEC1 | NM_015701 | 8.26E-05 | 1.48717 |
| 2888698 | LMAN2 | NM_006816 | 1.54E-04 | 1.48516 |
| 3019981 | MDFIC | NM_199072 | 6.40E-05 | 1.48362 |
| 2878726 | HDAC3 | NM_003883 | 2.27E-04 | 1.48076 |
| 3008164 | LAT2 | NM_032464 | 9.28E-05 | 1.48027 |
| 2949330 | CLIC1 | NM_001288 | 1.49E-03 | 1.47919 |
| 3685610 | ARHGAP17 | NM_001006634 | 1.69E-04 | 1.47911 |
| 3000167 | CCM2 | NM_031443 | 6.31E-04 | 1.47874 |
| 3849267 | ZNF558 | NM_144693 | 4.36E-05 | 1.47749 |
| 3847590 | RFX2 | NM_000635 | 6.92E-04 | 1.47712 |
| 3839642 | SIGLEC7 | NM_014385 | 1.42E-03 | 1.47681 |
| 3632298 | ADPGK | NR_023318 | 1.09E-04 | 1.47606 |
| 2555490 | XPO1 | NM_003400 | 9.60E-05 | 1.47501 |
| 3867538 | GYS1 | NM_002103 | 1.01E-03 | 1.47463 |
| 3476457 | NCOR2 | NM_006312 | 2.33E-04 | 1.47405 |
| 3071213 | ZNF800 | NM_176814 | 2.53E-04 | 1.47241 |
| 3770457 | FDXR | NM_024417 | 1.51E-06 | 1.4713 |
| 3726325 | XYLT2 | NM_022167 | 9.27E-04 | 1.46906 |
| 2911413 | PRIM2 | NM_000947 | 4.82E-04 | 1.46848 |
| 3092663 | WRN | NM_000553 | 3.05E-04 | 1.46826 |
| 2829562 | TXNDC15 | NM_024715 | 9.46E-05 | 1.46805 |
| 2446567 | STX6 | NM_005819 | 1.12E-03 | 1.46782 |
| 3718382 | ZNF830 | NM_052857 | 4.75E-04 | 1.46738 |
| 3535395 | TMX1 | NM_030755 | 8.67E-04 | 1.46592 |
| 3428447 | UTP20 | NM_014503 | 7.12E-04 | 1.46592 |
| 3589972 | CHST14 | NM_130468 | 2.54E-04 | 1.4645 |
| 3214749 | NOL8 | NR_024020 | 1.11E-03 | 1.4644 |
| 3917305 | BACH1 | NM_206866 | 6.67E-05 | 1.46348 |
| 3439305 | ZNF84 | NM_003428 | 6.25E-04 | 1.46194 |
| 2868523 | CHD1 | NM_001270 | 1.11E-04 | 1.46067 |
| 3161566 | KDM4C | NM_001146694 | 5.33E-05 | 1.46043 |
| 3673723 | TRAPPC2L | NM_016209 | 1.07E-03 | 1.45721 |
| 3452145 | SFRS2IP | NM_004719 | 7.88E-05 | 1.45682 |
| 3344897 | MED17 | NM_004268 | 4.66E-04 | 1.45598 |
| 3740998 | TSR1 | NM_018128 | 9.82E-04 | 1.45479 |
| 2728224 | SRP72 | NM_006947 | 8.94E-04 | 1.45281 |
| 2454532 | INTS7 | NM_015434 | 3.77E-05 | 1.45147 |
| 3336801 | ADRBK1 | NM_001619 | 1.46E-03 | 1.45121 |
| 3843058 | ZNF264 | NM_003417 | 3.04E-04 | 1.45008 |
| 3471753 | C12orf47 | NR_015404 | 8.14E-05 | 1.44774 |
| 3729002 | C17orf71 | NM_018149 | 3.21E-04 | 1.44762 |
| 3233322 | C10orf18 | NM_017782 | 3.74E-05 | 1.4471 |
| 2360647 | EFNA3 | NM_004952 | 7.99E-04 | 1.44653 |
| 3332663 | CD6 | NM_006725 | 2.05E-04 | 1.44535 |
| 3972093 | POLA1 | NM_016937 | 7.41E-04 | 1.44411 |
| 3910260 | ZNF217 | NM_006526 | 1.13E-03 | 1.44364 |
| 3833728 | ITPKC | NM_025194 | 1.94E-04 | 1.44323 |
| 2847292 | NSUN2 | NM_017755 | 8.91E-04 | 1.44201 |
| 2412529 | NRD1 | NM_002525 | 2.24E-04 | 1.44116 |
| 2808180 | LOC153684 | AK123995 | 7.77E-04 | 1.44027 |
| 3155901 | KCNK9 | NM_016601 | 8.05E-04 | 1.43931 |
| 2350287 | PRPF38B | NM_018061 | 7.13E-04 | 1.43791 |
| 3147286 | RRM2B | NM_015713 | 9.11E-04 | 1.43729 |
| 2676009 | TWF2 | NM_007284 | 7.00E-04 | 1.43684 |
| 2824354 | DCP2 | NM_152624 | 1.21E-03 | 1.43559 |
| 3359529 | CARS | NM_001014438 | 6.33E-04 | 1.43558 |
| 3663228 | GINS3 | NM_001126129 | 6.43E-04 | 1.43519 |
| 3696142 | DPEP2 | NM_022355 | 1.47E-03 | 1.43514 |
| 2458338 | ENAH | NM_001008493 | 1.47E-03 | 1.43472 |
| 2891241 | DUSP22 | NM_020185 | 4.91E-04 | 1.43412 |
| 3795680 | THOC1 | NM_005131 | 6.43E-04 | 1.43316 |
| 2882555 | FAM114A2 | NM_018691 | 7.94E-05 | 1.43225 |
| 3833583 | SHKBP1 | NM_138392 | 1.00E-04 | 1.43225 |
| 3453774 | LMBR1L | NM_018113 | 1.09E-04 | 1.43211 |
| 2890413 | RNF130 | NM_018434 | 1.81E-07 | 1.43148 |
| 3217807 | TEX10 | NM_017746 | 2.71E-04 | 1.43057 |
| 2358736 | TNFAIP8L2 | NM_024575 | 1.05E-03 | 1.42917 |
| 3937587 | MED15 | NM_001003891 | 1.43E-05 | 1.42906 |
| 2758686 | LYAR | NM_017816 | 4.06E-05 | 1.42813 |
| 3893520 | RTEL1 | NM_032957 | 9.59E-04 | 1.42658 |
| 3706000 | RPA1 | NM_002945 | 3.12E-04 | 1.42616 |
| 3954238 | MAPK1 | NM_002745 | 8.02E-04 | 1.42572 |
| 3854000 | SLC35E1 | NM_024881 | 7.42E-05 | 1.42484 |
| 3089285 | POLR3D | NM_001722 | 4.39E-05 | 1.4219 |
| 2379280 | FLVCR1 | NM_014053 | 1.06E-04 | 1.42092 |
| 2774049 | SCARB2 | NM_005506 | 8.71E-06 | 1.42091 |
| 2539125 | CMPK2 | NM_207315 | 8.71E-04 | 1.41939 |
| 3191147 | TOR1B | NM_014506 | 3.01E-05 | 1.41807 |
| 2633390 | COL8A1 | NM_001850 | 1.41E-03 | 1.41768 |
| 3957445 | PES1 | NM_014303 | 3.29E-04 | 1.41715 |
| 3795184 | NFATC1 | NM_172387 | 1.29E-03 | 1.41495 |
| 3050367 | FIGNL1 | NM_001042762 | 8.12E-04 | 1.41295 |
| 2554975 | BCL11A | NM_022893 | 3.41E-04 | 1.41104 |
| 2420808 | BCL10 | NM_003921 | 2.29E-04 | 1.40882 |
| 2454818 | BATF3 | NM_018664 | 6.91E-05 | 1.40634 |
| 3562671 | KLHL28 | NM_017658 | 3.47E-05 | 1.40559 |
| 3893849 | PRPF6 | NM_012469 | 1.40E-03 | 1.40405 |
| 3290210 | ZWINT | NM_032997 | 1.53E-05 | 1.40403 |
| 2370355 | IER5 | NM_016545 | 1.23E-04 | 1.40376 |
| 3836044 | GEMIN7 | NM_024707 | 4.90E-04 | 1.40283 |
| 3448428 | C12orf11 | NM_018164 | 1.49E-03 | 1.40152 |
| 3245881 | WDFY4 | NM_020945 | 3.84E-04 | 1.4008 |
| 3736162 | TMC8 | NM_152468 | 6.78E-04 | 1.40024 |
