# Supplementary material for: Genome-Wide Analysis of Differentially Expressed Genes and Splicing Isoforms in Clear Cell Renal Cell Carcinoma
Source: PLoS One. 2013 Oct 23;8(10):e78452. doi: 10.1371/journal.pone.0078452 (PMC3806822; doi:10.1371/journal.pone.0078452)
Supplement: Table S3 — List of down-regulated genes in ccRCC respect to non-tumoral samples as resulted by gene-level Partek analysis of Affymetrix Exon Arrays. The list contains only genes showing a linear fold change < -1.4 for ccRCC vs NT comparison and FDR corrected p-value less or equal to 0.01. For each gene the Affymetrix transcript ID, the RefSeq ID, the fold change as well as the p-value for the comparison are reported. (DOCX) [file pone.0078452.s003.docx]

| Transcript ID | Gene Symbol | RefSeq | p-value  (Status) | Fold-Change  (RCC vs. N) |
| --- | --- | --- | --- | --- |
| 3593065 | SLC12A1 | NM_000338 | 3,59E-17 | -232,945 |
| 3683549 | UMOD | NM_003361 | 1,61E-15 | -199,332 |
| 3144033 | CALB1 | NM_004929 | 4,97E-11 | -195,99 |
| 2656683 | KNG1 | NM_000893 | 1,00E-10 | -153,195 |
| 2881950 | SLC36A2 | NM_181776 | 5,70E-08 | -100,868 |
| 3376367 | SLC22A8 | NM_004254 | 9,43E-08 | -99,5614 |
| 3990460 | XPNPEP2 | NM_003399 | 1,99E-07 | -73,4499 |
| 2657808 | CLDN16 | NM_006580 | 1,42E-16 | -69,8656 |
| 2446137 | NPHS2 | NM_014625 | 4,84E-09 | -63,051 |
| 2934682 | PLG | NM_000301 | 3,72E-06 | -57,684 |
| 3692999 | MT1G | NM_005950 | 8,44E-08 | -55,9918 |
| 3907987 | SLC13A3 | NM_022829 | 2,15E-06 | -54,5709 |
| 3397774 | KCNJ1 | NM_153767 | 1,35E-15 | -53,6939 |
| 3218077 | ALDOB | NM_000035 | 1,10E-05 | -50,591 |
| 3414296 | AQP2 | NM_000486 | 3,58E-15 | -47,1624 |
| 3404626 | C12orf59 | NM_153022 | 5,34E-08 | -44,3078 |
| 3715614 | SLC13A2 | NM_001145975 | 1,15E-08 | -43,6843 |
| 3726406 | ACSF2 | NM_025149 | 1,67E-09 | -42,0789 |
| 2731257 | AFM | NM_001133 | 8,06E-08 | -42,0399 |
| 2807716 | C7 | NM_000587 | 1,58E-07 | -40,6344 |
| 2843131 | SLC34A1 | NM_003052 | 3,03E-06 | -39,1481 |
| 3662333 | SLC12A3 | NM_000339 | 6,38E-08 | -37,9215 |
| 2511712 | UPP2 | NM_001135098 | 8,34E-08 | -36,8177 |
| 2656650 | HRG | NM_000412 | 2,49E-11 | -36,1172 |
| 2563536 | FABP1 | NM_001443 | 1,34E-06 | -35,4763 |
| 2336891 | DIO1 | NM_000792 | 5,05E-07 | -35,1942 |
| 3468473 | PAH | NM_000277 | 4,53E-06 | -34,9441 |
| 3549740 | SERPINA5 | NM_000624 | 5,89E-13 | -33,7928 |
| 2322264 | CLCNKB | NM_000085 | 1,27E-07 | -33,4448 |
| 3304522 | CYP17A1 | NM_000102 | 2,00E-07 | -33,3139 |
| 3366903 | MUC15 | NM_001135091 | 1,32E-11 | -32,2368 |
| 2731192 | ALB | NM_000477 | 1,53E-07 | -32,011 |
| 2439960 | KCNJ10 | NM_002241 | 8,91E-16 | -30,9725 |
| 3652867 | SCNN1G | NM_001039 | 1,71E-14 | -30,51 |
| 3075381 | ATP6V0A4 | NM_020632 | 1,00E-05 | -29,573 |
| 3674249 | DPEP1 | NM_004413 | 1,23E-05 | -27,8858 |
| 3748909 | SLC47A2 | NM_152908 | 1,38E-07 | -26,275 |
| 2354365 | HAO2 | NM_001005783 | 5,02E-06 | -25,8561 |
| 2739308 | EGF | NM_001963 | 5,77E-07 | -25,1961 |
| 3132782 | SFRP1 | NM_003012 | 4,56E-15 | -24,5012 |
| 3833926 | CYP2B6 | NM_000767 | 6,63E-08 | -23,573 |
| 2573570 | TFCP2L1 | NM_014553 | 2,79E-07 | -22,9809 |
| 3722248 | G6PC | NM_000151 | 4,86E-06 | -22,9808 |
| 2783886 | C4orf31 | NM_024574 | 4,63E-08 | -22,3203 |
| 2413846 | FAM151A | NM_147161 | 8,94E-07 | -22,0034 |
| 4022447 | GPC3 | NM_001164617 | 1,39E-10 | -21,6017 |
| 3475383 | HPD | NM_001171993 | 2,91E-06 | -18,7559 |
| 3928415 | CLDN8 | NM_199328 | 1,09E-06 | -18,6262 |
| 3959862 | PVALB | NM_002854 | 3,83E-04 | -18,1649 |
| 3715935 | PIPOX | NM_016518 | 4,89E-05 | -18,1507 |
| 3430868 | DAO | NM_001917 | 2,91E-06 | -18,1495 |
| 2755154 | F11 | NM_000128 | 2,39E-11 | -17,475 |
| 3461981 | TSPAN8 | NM_004616 | 3,52E-08 | -17,3544 |
| 3923075 | CRYAA | NM_000394 | 1,06E-06 | -17,0206 |
| 3657219 | SLC5A2 | NM_003041 | 1,79E-07 | -16,0355 |
| 3662201 | MT1H | NM_005951 | 8,82E-08 | -15,9562 |
| 3105749 | ATP6V0D2 | NM_152565 | 5,42E-05 | -15,857 |
| 3376326 | SLC22A6 | NM_004790 | 1,78E-04 | -15,8337 |
| 2322226 | CLCNKA | NM_004070 | 7,20E-07 | -15,7571 |
| 2845362 | SLC9A3 | NM_004174 | 3,14E-09 | -15,4368 |
| 2621032 | PTH1R | NM_000316 | 2,23E-09 | -15,2051 |
| 3143282 | SLC7A13 | NM_138817 | 8,46E-08 | -15,0984 |
| 3175971 | PSAT1 | NM_058179 | 6,44E-05 | -14,8315 |
| 3890640 | PCK1 | NM_002591 | 2,01E-05 | -14,7382 |
| 3557209 | SLC7A8 | NM_012244 | 1,83E-06 | -14,3925 |
| 3122678 | DEFB1 | NM_005218 | 3,16E-05 | -14,2575 |
| 2955556 | CLIC5 | NM_001114086 | 4,79E-08 | -14,0552 |
| 3742182 | GGT6 | NM_001122890 | 8,25E-10 | -13,6008 |
| 3868681 | KLK1 | NM_002257 | 6,71E-04 | -13,475 |
| 3853609 | CYP4F2 | NM_001082 | 4,89E-06 | -13,4371 |
| 3334659 | SLC22A12 | NM_144585 | 1,26E-06 | -13,3196 |
| 3638411 | RHCG | NM_016321 | 1,95E-04 | -13,2369 |
| 3465274 | DCN | NM_001920 | 2,21E-07 | -12,9667 |
| 2597552 | ERBB4 | NM_005235 | 1,62E-07 | -12,6587 |
| 2853426 | RANBP3L | NM_001161429 | 2,25E-06 | -12,624 |
| 2339454 | ANGPTL3 | NM_014495 | 6,00E-04 | -12,5386 |
| 3441885 | SCNN1A | NM_001038 | 2,09E-05 | -12,517 |
| 2949488 | SLC44A4 | NM_025257 | 7,93E-05 | -12,3203 |
| 3244008 | FXYD4 | NM_173160 | 6,48E-08 | -12,2257 |
| 2711604 | CPN2 | NM_001080513 | 5,35E-04 | -12,0568 |
| 3182310 | LPPR1 | NM_207299 | 1,89E-08 | -11,9624 |
| 3215570 | FBP1 | NM_000507 | 1,15E-06 | -11,7946 |
| 2815238 | TMEM174 | NM_153217 | 2,68E-04 | -11,7497 |
| 3950846 | MIOX | NM_017584 | 8,79E-06 | -11,7464 |
| 3063589 | AZGP1 | NM_001185 | 9,95E-04 | -11,6832 |
| 2617630 | SLC22A13 | NM_004256 | 5,21E-08 | -11,67 |
| 2710632 | TMEM207 | NM_207316 | 2,31E-11 | -11,6026 |
| 2796224 | ENPP6 | NM_153343 | 1,12E-07 | -11,4091 |
| 3921599 | PCP4 | NM_006198 | 3,33E-04 | -11,2627 |
| 3061484 | HEPACAM2 | NM_001039372 | 7,43E-11 | -11,1999 |
| 3933039 | TMPRSS2 | NM_001135099 | 4,78E-05 | -11,1509 |
| 3767709 | APOH | NM_000042 | 2,80E-04 | -11,0558 |
| 2740067 | ANK2 | NM_001148 | 2,11E-07 | -10,9386 |
| 2779163 | ADH6 | NM_001102470 | 7,12E-05 | -10,9221 |
| 3571727 | ALDH6A1 | NM_005589 | 2,82E-06 | -10,8716 |
| 3823304 | CYP4F3 | NM_000896 | 4,62E-07 | -10,8471 |
| 3541450 | RDH12 | NM_152443 | 1,83E-05 | -10,8146 |
| 3197955 | GLDC | NM_000170 | 5,14E-08 | -10,3651 |
| 2692883 | MUC13 | NM_033049 | 7,43E-04 | -10,2871 |
| 2487918 | ATP6V1B1 | NM_001692 | 8,71E-06 | -10,2616 |
| 2376548 | MFSD4 | NM_181644 | 1,87E-08 | -10,1554 |
| 3414419 | GPD1 | NM_005276 | 9,75E-04 | -9,83833 |
| 3300749 | RBP4 | NM_006744 | 7,06E-04 | -9,83051 |
| 2853102 | PRLR | NM_000949 | 2,35E-05 | -9,70802 |
| 2779271 | ADH1C | NM_000669 | 4,66E-10 | -9,44777 |
| 3576749 | FBLN5 | NM_006329 | 2,05E-05 | -9,40011 |
| 3261859 | SFXN2 | NM_178858 | 2,12E-08 | -9,31622 |
| 2493858 | MAL | NM_002371 | 6,80E-06 | -9,16615 |
| 3860003 | PRODH2 | NM_021232 | 2,90E-06 | -9,15487 |
| 3625052 | WDR72 | NM_182758 | 8,96E-05 | -9,11857 |
| 2451870 | ETNK2 | NM_018208 | 6,94E-06 | -9,08006 |
| 2794408 | HPGD | NM_000860 | 2,10E-05 | -9,04314 |
| 2955932 | GPR110 | NM_153840 | 1,15E-03 | -9,03474 |
| 2623441 | ACY1 | NM_000666 | 7,13E-07 | -8,97587 |
| 3106559 | SLC26A7 | NM_052832 | 4,07E-04 | -8,97235 |
| 3191877 | AIF1L | NM_031426 | 3,58E-08 | -8,90426 |
| 3198346 | PTPRD | NM_002839 | 6,13E-05 | -8,88465 |
| 3326461 | EHF | NM_012153 | 4,57E-08 | -8,87832 |
| 3502475 | PROZ | NM_003891 | 2,00E-06 | -8,86591 |
| 2907887 | SLC22A7 | NM_006672 | 5,07E-08 | -8,76213 |
| 2926447 | TCF21 | NM_003206 | 6,42E-17 | -8,74077 |
| 3445768 | ERP27 | NM_152321 | 3,85E-05 | -8,72148 |
| 3598267 | OSTBETA | NM_178859 | 1,29E-04 | -8,71049 |
| 2358591 | ANXA9 | NM_003568 | 5,30E-12 | -8,68837 |
| 3665501 | HSD11B2 | NM_000196 | 2,90E-05 | -8,59647 |
| 2453006 | PIGR | NM_002644 | 9,43E-04 | -8,54941 |
| 2433232 | FMO5 | NM_001461 | 2,53E-08 | -8,49702 |
| 2638728 | SLC15A2 | NM_021082 | 5,76E-10 | -8,44556 |
| 2931172 | IYD | NM_001164694 | 1,67E-06 | -8,43423 |
| 4054204 | APOD | NM_001647 | 1,03E-08 | -8,39896 |
| 2583374 | PLA2R1 | NM_007366 | 1,40E-06 | -8,32208 |
| 3523118 | A2LD1 | NM_033110 | 3,64E-07 | -8,30856 |
| 3652902 | SCNN1B | NM_000336 | 3,60E-06 | -8,27966 |
| 4007550 | PCSK1N | NM_013271 | 3,68E-08 | -8,278 |
| 2361257 | RAB25 | NM_020387 | 2,93E-05 | -8,27712 |
| 3486096 | FREM2 | NM_207361 | 1,35E-04 | -8,18986 |
| 2648098 | SUCNR1 | NM_033050 | 2,63E-07 | -8,18861 |
| 3480885 | FGF9 | NM_002010 | 3,54E-04 | -8,18468 |
| 3868400 | NAPSA | NM_004851 | 1,80E-04 | -8,18002 |
| 3456840 | PPP1R1A | NM_006741 | 1,74E-05 | -8,17183 |
| 3288803 | OGDHL | NM_018245 | 4,19E-05 | -8,13146 |
| 2879166 | FGF1 | NR_026695 | 1,51E-06 | -8,05613 |
| 2734047 | AGPAT9 | NM_032717 | 1,89E-05 | -7,98646 |
| 2678400 | ACOX2 | NM_003500 | 9,61E-07 | -7,80899 |
| 3050388 | DDC | NM_001082971 | 3,20E-04 | -7,7857 |
| 2902531 | APOM | NM_019101 | 9,85E-04 | -7,78549 |
| 3259937 | DHDPSL | NM_138413 | 7,92E-09 | -7,77144 |
| 3393891 | TREH | NM_007180 | 3,67E-06 | -7,68849 |
| 3811000 | RNF152 | NM_173557 | 1,80E-05 | -7,67448 |
| 2642720 | ACPP | NM_001099 | 3,09E-11 | -7,56092 |
| 3529508 | PCK2 | NM_004563 | 2,08E-05 | -7,51849 |
| 2431031 | HMGCS2 | NM_005518 | 5,75E-04 | -7,43254 |
| 3939875 | SUSD2 | NM_019601 | 9,92E-05 | -7,42393 |
| 3195034 | PTGDS | NM_000954 | 5,44E-09 | -7,41202 |
| 2737257 | MTTP | NM_000253 | 5,14E-04 | -7,4047 |
| 2999334 | HECW1 | NM_015052 | 1,73E-06 | -7,27785 |
| 2816536 | CRHBP | NM_001882 | 3,80E-07 | -7,25651 |
| 3190796 | PHYHD1 | NM_001100876 | 1,39E-06 | -7,24944 |
| 3105271 | RALYL | NM_001100392 | 9,50E-13 | -7,1412 |
| 2779199 | ADH1A | NM_000667 | 2,08E-07 | -7,04167 |
| 2397732 | AGMAT | NM_024758 | 2,48E-04 | -7,0121 |
| 3208494 | C9orf71 | NM_153237 | 6,57E-04 | -7,00378 |
| 3497195 | CLDN10 | NM_182848 | 1,34E-03 | -6,99664 |
| 3595315 | CGNL1 | NM_032866 | 8,65E-07 | -6,93837 |
| 2377094 | PFKFB2 | NM_006212 | 7,47E-07 | -6,91601 |
| 2367086 | FMO4 | NM_002022 | 3,50E-05 | -6,87315 |
| 3356038 | TMEM45B | NM_138788 | 4,26E-07 | -6,77838 |
| 3272761 | PRAP1 | NM_145202 | 6,12E-04 | -6,75283 |
| 3021696 | ASB15 | NM_080928 | 1,63E-05 | -6,67406 |
| 3268588 | ACADSB | NM_001609 | 6,88E-06 | -6,66129 |
| 3858757 | SLC7A9 | NM_014270 | 7,10E-04 | -6,64226 |
| 2427208 | GSTM3 | NM_000849 | 9,27E-06 | -6,61175 |
| 3759006 | SLC4A1 | NM_000342 | 3,47E-04 | -6,60475 |
| 3406329 | PTPRO | NM_030667 | 1,75E-06 | -6,60231 |
| 3424218 | ACSS3 | NM_024560 | 1,85E-04 | -6,557 |
| 2430163 | VTCN1 | NM_024626 | 3,06E-10 | -6,553 |
| 3662150 | MT1M | NM_176870 | 7,03E-07 | -6,46084 |
| 2975257 | ALDH8A1 | NM_022568 | 3,75E-04 | -6,4092 |
| 2402517 | SLC30A2 | NM_001004434 | 6,19E-07 | -6,40913 |
| 2607568 | CHL1 | NM_006614 | 1,60E-04 | -6,24886 |
| 3868783 | KLK7 | NM_139277 | 7,90E-08 | -6,14242 |
| 3186491 | PAPPA | NM_002581 | 2,18E-09 | -6,04116 |
| 2455933 | ESRRG | NR_024099 | 6,35E-05 | -6,02583 |
| 2853293 | UGT3A1 | NM_152404 | 9,35E-04 | -6,0061 |
| 2478269 | TMEM178 | NM_152390 | 1,28E-06 | -5,9993 |
| 3815936 | REEP6 | NM_138393 | 1,41E-06 | -5,99461 |
| 2676854 | CHDH | NM_018397 | 7,49E-05 | -5,93762 |
| 2354634 | PHGDH | NM_006623 | 5,33E-06 | -5,89989 |
| 2797311 | MTNR1A | NM_005958 | 1,33E-05 | -5,87769 |
| 2469529 | PDIA6 | NM_005742 | 5,43E-04 | -5,87278 |
| 2450762 | TNNT2 | NM_000364 | 9,05E-08 | -5,872 |
| 3847462 | FUT6 | NM_000150 | 6,00E-04 | -5,85122 |
| 2693409 | ALDH1L1 | NM_012190 | 9,05E-05 | -5,84259 |
| 3807965 | MRO | NM_031939 | 1,91E-05 | -5,83557 |
| 2779231 | ADH1B | NM_000668 | 1,23E-07 | -5,80394 |
| 3850457 | AP1M2 | NM_005498 | 9,84E-06 | -5,77282 |
| 2521574 | PLCL1 | NM_006226 | 4,36E-08 | -5,75041 |
| 3292590 | PBLD | NM_022129 | 9,29E-04 | -5,73588 |
| 3202224 | LRRC19 | NM_022901 | 4,34E-05 | -5,71181 |
| 3975935 | RGN | NM_152869 | 3,58E-05 | -5,69842 |
| 2532480 | EFHD1 | NM_025202 | 1,85E-05 | -5,69677 |
| 3406880 | PIK3C2G | NM_004570 | 8,13E-11 | -5,61551 |
| 2638824 | CASR | NM_000388 | 3,28E-06 | -5,61052 |
| 2851511 | CDH9 | NM_016279 | 3,24E-06 | -5,49885 |
| 2956904 | PKHD1 | NM_138694 | 3,80E-04 | -5,49482 |
| 3995765 | DUSP9 | NM_001395 | 4,61E-10 | -5,48216 |
| 2877861 | SLC23A1 | NM_005847 | 2,84E-04 | -5,3883 |
| 3935192 | FTCD | NM_006657 | 1,37E-04 | -5,37504 |
| 2435005 | SELENBP1 | NM_003944 | 6,20E-08 | -5,37199 |
| 2450798 | LAD1 | NM_005558 | 2,32E-10 | -5,33184 |
| 3695315 | CDH16 | NM_004062 | 1,25E-03 | -5,33164 |
| 3085403 | MSRA | NM_012331 | 8,92E-05 | -5,31809 |
| 3378541 | PC | NM_001040716 | 7,20E-07 | -5,28119 |
| 2892393 | BPHL | NR_026650 | 1,88E-05 | -5,22935 |
| 3234277 | GATA3 | NM_001002295 | 1,31E-10 | -5,21117 |
| 3517251 | DACH1 | NM_080759 | 2,20E-10 | -5,19143 |
| 3929458 | C21orf62 | NM_001162495 | 2,33E-04 | -5,19025 |
| 3169331 | ALDH1B1 | NM_000692 | 1,65E-05 | -5,17156 |
| 3748323 | SHMT1 | NM_004169 | 9,58E-05 | -5,13857 |
| 3411721 | CNTN1 | NM_001843 | 1,33E-11 | -5,13051 |
| 3540136 | HSPA2 | NM_021979 | 4,83E-06 | -5,11783 |
| 2995254 | C7orf41 | NM_152793 | 1,35E-06 | -5,10812 |
| 2504595 | PROC | NM_000312 | 4,48E-05 | -5,0939 |
| 3415320 | KRT7 | NM_005556 | 1,05E-08 | -5,08691 |
| 3203990 | KIAA1161 | NM_020702 | 3,12E-06 | -5,04861 |
| 3292413 | DNAJC12 | NM_021800 | 7,44E-06 | -4,96863 |
| 3434193 | CCDC64 | NM_207311 | 2,68E-08 | -4,96779 |
| 3216671 | CTSL2 | NM_001333 | 7,72E-06 | -4,95044 |
| 3977299 | CLCN5 | NM_001127899 | 1,11E-04 | -4,94297 |
| 2909948 | TFAP2B | NM_003221 | 3,21E-10 | -4,94061 |
| 3323556 | NELL1 | NM_006157 | 2,94E-09 | -4,92649 |
| 2731636 | PARM1 | NM_015393 | 1,79E-04 | -4,92186 |
| 2725013 | UCHL1 | NM_004181 | 1,17E-03 | -4,91201 |
| 3070183 | AASS | NM_005763 | 5,54E-08 | -4,88773 |
| 2674501 | AMT | NM_000481 | 6,78E-09 | -4,87924 |
| 3946944 | CSDC2 | NM_014460 | 4,71E-08 | -4,84773 |
| 3137875 | GGH | NM_003878 | 3,45E-04 | -4,83705 |
| 3774535 | DCXR | NM_016286 | 1,53E-07 | -4,83246 |
| 2561955 | SUCLG1 | NM_003849 | 5,08E-08 | -4,80794 |
| 2414958 | TACSTD2 | NM_002353 | 5,53E-05 | -4,79654 |
| 3771675 | ST6GALNAC2 | NM_006456 | 8,11E-07 | -4,79618 |
| 2368590 | PAPPA2 | NM_020318 | 2,53E-07 | -4,79239 |
| 2341663 | CTH | NM_001902 | 1,12E-06 | -4,78551 |
| 3529467 | CPNE6 | NM_006032 | 2,78E-04 | -4,78273 |
| 3842755 | LOC100288114 | AK302988 | 1,42E-03 | -4,75403 |
| 3859026 | PEPD | NM_000285 | 3,43E-06 | -4,72089 |
| 3592755 | SEMA6D | NM_153618 | 4,34E-05 | -4,7183 |
| 2670975 | CYP8B1 | NM_004391 | 6,25E-06 | -4,71794 |
| 2690715 | IGSF11 | NM_001015887 | 3,16E-05 | -4,6488 |
| 3921992 | FAM3B | NM_058186 | 4,53E-07 | -4,6442 |
| 3353640 | GRAMD1B | NM_020716 | 1,20E-05 | -4,64175 |
| 3653516 | SLC5A11 | NM_052944 | 1,17E-06 | -4,63899 |
| 3687475 | GDPD3 | NM_024307 | 3,83E-04 | -4,63789 |
| 2738378 | NPNT | NM_001033047 | 4,70E-04 | -4,62493 |
| 3722084 | WNK4 | NM_032387 | 6,70E-09 | -4,5929 |
| 2974935 | SLC2A12 | NM_145176 | 9,00E-05 | -4,58174 |
| 3699335 | LDHD | NM_153486 | 3,11E-06 | -4,57617 |
| 2845351 | LOC25845 | NR_024158 | 4,52E-06 | -4,56741 |
| 3369117 | ELF5 | NM_198381 | 1,08E-06 | -4,53991 |
| 3738224 | SLC25A10 | NM_012140 | 7,83E-05 | -4,53898 |
| 3394356 | USP2 | NM_004205 | 2,30E-05 | -4,50771 |
| 2952834 | KCNK5 | NM_003740 | 9,89E-05 | -4,50328 |
| 3446137 | LMO3 | NM_018640 | 5,75E-09 | -4,46867 |
| 2326049 | MAN1C1 | NM_020379 | 6,92E-06 | -4,45777 |
| 3592109 | SORD | NM_003104 | 3,16E-05 | -4,42539 |
| 2498911 | SULT1C2 | NM_001056 | 1,95E-04 | -4,41653 |
| 3571667 | ENTPD5 | NM_001249 | 8,73E-04 | -4,40837 |
| 3367673 | MPPED2 | NM_001584 | 1,34E-03 | -4,39597 |
| 3632806 | STRA6 | NM_022369 | 1,63E-04 | -4,39472 |
| 3868768 | KLK6 | NM_002774 | 8,35E-07 | -4,39332 |
| 3743883 | SAT2 | NM_133491 | 1,48E-07 | -4,39164 |
| 2637831 | UPK1B | NM_006952 | 3,40E-05 | -4,38955 |
| 2590582 | PDE1A | NM_005019 | 6,58E-05 | -4,36704 |
| 2738723 | HADH | NM_005327 | 6,29E-07 | -4,34027 |
| 3959918 | TST | NM_003312 | 1,81E-04 | -4,33892 |
| 3750785 | SPAG5 | NM_006461 | 1,46E-06 | -4,31059 |
| 2615808 | GPD1L | NM_015141 | 1,03E-04 | -4,24885 |
| 3332838 | DAK | NM_015533 | 1,16E-06 | -4,24185 |
| 2453036 | FCAMR | NM_001170631 | 2,25E-05 | -4,2346 |
| 2364677 | PBX1 | NM_002585 | 4,71E-05 | -4,22764 |
| 3269065 | LHPP | NM_022126 | 4,20E-06 | -4,21956 |
| 2319340 | SLC25A33 | NM_032315 | 3,74E-06 | -4,21655 |
| 3221395 | ALAD | NM_000031 | 3,74E-08 | -4,20943 |
| 3039671 | SOSTDC1 | NM_015464 | 2,12E-05 | -4,20795 |
| 2726542 | CWH43 | NM_025087 | 1,19E-03 | -4,19173 |
| 3060182 | ABCB1 | NM_000927 | 3,74E-04 | -4,17932 |
| 3696554 | TMED6 | NM_144676 | 4,83E-04 | -4,15195 |
| 2452667 | RAB7L1 | NM_003929 | 1,33E-04 | -4,13316 |
| 3163818 | SH3GL2 | NM_003026 | 1,29E-04 | -4,13213 |
| 3091403 | EPHX2 | NM_001979 | 4,65E-05 | -4,13004 |
| 2921402 | SLC16A10 | NM_018593 | 1,65E-04 | -4,11 |
| 2945440 | DCDC2 | NM_016356 | 2,90E-04 | -4,08448 |
| 3730698 | KCNH6 | NM_030779 | 3,25E-06 | -4,06912 |
| 2905025 | PNPLA1 | NM_173676 | 4,74E-06 | -4,0349 |
| 2399743 | AKR7A3 | NM_012067 | 6,07E-04 | -4,03009 |
| 2896545 | GMPR | NM_006877 | 1,52E-03 | -4,00592 |
| 2445982 | ANGPTL1 | NM_004673 | 2,65E-04 | -3,97563 |
| 2840270 | KCNIP1 | NM_001034837 | 4,87E-07 | -3,96685 |
| 2402431 | PAQR7 | NM_178422 | 1,89E-08 | -3,96346 |
| 4014029 | RPS6KA6 | NM_014496 | 4,85E-05 | -3,96197 |
| 3986087 | NRK | NM_198465 | 6,48E-06 | -3,93744 |
| 3176933 | C9orf103 | NM_001001551 | 1,31E-06 | -3,93159 |
| 3947952 | PNPLA3 | NM_025225 | 1,07E-04 | -3,90723 |
| 2471233 | VSNL1 | NM_003385 | 1,54E-06 | -3,90035 |
| 3063501 | CYP3A4 | NM_017460 | 3,54E-05 | -3,89726 |
| 7385641 | CLSTN2 | NM_022131 | 1,01E-08 | -3,87642 |
| 3376512 | HRASLS2 | NM_017878 | 9,29E-05 | -3,8741 |
| 2622607 | SLC38A3 | NM_006841 | 4,34E-04 | -3,86184 |
| 2617687 | XYLB | NM_005108 | 1,49E-05 | -3,85749 |
| 2493943 | PROM2 | NM_001165978 | 1,88E-07 | -3,83892 |
| 2422722 | TGFBR3 | NM_003243 | 1,11E-05 | -3,83007 |
| 2497252 | SLC9A2 | NM_003048 | 9,93E-05 | -3,81705 |
| 3209060 | TRPM3 | NM_001007471 | 1,27E-03 | -3,80332 |
| 2498274 | C2orf40 | NM_032411 | 5,03E-06 | -3,7987 |
| 3378433 | SPTBN2 | NM_006946 | 5,69E-06 | -3,79778 |
| 3975227 | MAOA | NM_000240 | 3,70E-04 | -3,79102 |
| 2602770 | DNER | NM_139072 | 3,97E-08 | -3,78785 |
| 3942766 | INPP5J | NM_001002837 | 2,16E-04 | -3,76511 |
| 2669533 | ACAA1 | NM_001607 | 4,68E-06 | -3,76444 |
| 2477933 | GALM | NM_138801 | 1,09E-04 | -3,74418 |
| 2693511 | KLF15 | NM_014079 | 1,63E-04 | -3,7431 |
| 2678298 | DNASE1L3 | NM_004944 | 1,82E-05 | -3,74209 |
| 2998638 | C7orf10 | NM_024728 | 4,87E-04 | -3,7347 |
| 3833893 | CYP2B7P1 | NR_001278 | 1,44E-07 | -3,72708 |
| 3868518 | ASPDH | NM_001114598 | 6,17E-06 | -3,72672 |
| 3864519 | CADM4 | NM_145296 | 3,77E-06 | -3,71569 |
| 3566495 | C14orf37 | AK292513 | 4,78E-07 | -3,68556 |
| 3886453 | HNF4A | NM_000457 | 1,28E-03 | -3,6852 |
| 3543673 | ACOT2 | NM_006821 | 5,49E-07 | -3,66629 |
| 3339406 | FOLR1 | NM_016724 | 7,71E-05 | -3,66065 |
| 2966587 | SIM1 | NM_005068 | 6,68E-04 | -3,65759 |
| 3664952 | PDP2 | NM_020786 | 4,92E-06 | -3,64314 |
| 3324447 | FIBIN | NM_203371 | 4,41E-05 | -3,63853 |
| 3315231 | ECHS1 | NM_004092 | 2,06E-05 | -3,62448 |
| 3847486 | FUT3 | NM_000149 | 3,38E-06 | -3,61491 |
| 2337147 | ACOT11 | NM_147161 | 4,55E-09 | -3,59893 |
| 3504226 | CRYL1 | NM_015974 | 2,24E-04 | -3,59705 |
| 3930212 | KCNE1 | NM_000219 | 2,79E-05 | -3,59449 |
| 3498837 | PCCA | NM_000282 | 9,04E-05 | -3,59308 |
| 3719210 | DHRS11 | NM_024308 | 4,39E-07 | -3,59304 |
| 3739147 | FN3K | NM_022158 | 2,60E-04 | -3,5927 |
| 2727116 | RASL11B | NM_023940 | 1,83E-07 | -3,59087 |
| 3550139 | TCL1B | NM_199206 | 2,26E-07 | -3,58904 |
| 3400384 | HSN2 | NM_213655 | 3,95E-04 | -3,56997 |
| 3101629 | ADHFE1 | NM_144650 | 1,39E-04 | -3,56855 |
| 2892277 | NQO2 | NM_000904 | 6,72E-05 | -3,55575 |
| 2762334 | QDPR | NM_000320 | 2,02E-04 | -3,53313 |
| 2590452 | CERKL | NM_201548 | 7,83E-04 | -3,52355 |
| 3452818 | VDR | NM_001017535 | 5,13E-04 | -3,52249 |
| 3166477 | ACO1 | NM_002197 | 3,15E-05 | -3,50248 |
| 3768627 | ABCA8 | NM_007168 | 2,85E-10 | -3,49996 |
| 4022370 | GPC4 | NM_001448 | 1,09E-03 | -3,49104 |
| 3788097 | MAPK4 | NM_002747 | 1,44E-11 | -3,4905 |
| 4019160 | KLHL13 | NM_001168302 | 8,01E-05 | -3,48401 |
| 2853325 | UGT3A2 | NM_174914 | 9,67E-06 | -3,48395 |
| 3886143 | SGK2 | NM_016276 | 9,90E-04 | -3,46915 |
| 3860045 | NPHS1 | NM_004646 | 6,04E-05 | -3,46459 |
| 3432090 | ALDH2 | NM_000690 | 4,53E-05 | -3,44879 |
| 3203569 | AQP3 | NM_004925 | 5,42E-04 | -3,44472 |
| 3821995 | GCDH | NM_000159 | 2,23E-06 | -3,44291 |
| 3162486 | TYRP1 | NM_000550 | 2,13E-06 | -3,43976 |
| 2451593 | CHI3L1 | NM_001276 | 4,98E-04 | -3,4194 |
| 3590498 | TYRO3 | NM_006293 | 6,96E-06 | -3,40946 |
| 2877028 | KLHL3 | NM_017415 | 1,47E-03 | -3,39855 |
| 3023318 | TSPAN33 | NM_178562 | 4,24E-05 | -3,38069 |
| 3496409 | GPC5 | NM_004466 | 9,88E-09 | -3,38023 |
| 3577940 | CLMN | NM_024734 | 7,15E-06 | -3,37849 |
| 2584712 | GRB14 | NM_004490 | 2,41E-04 | -3,37625 |
| 3949017 | TTC38 | NM_017931 | 1,03E-03 | -3,374 |
| 2538600 | ADI1 | NM_018269 | 7,75E-06 | -3,36356 |
| 3972929 | GK | NM_001128127 | 8,91E-05 | -3,3584 |
| 2793401 | MFAP3L | NM_021647 | 3,01E-04 | -3,35424 |
| 2427500 | HBXIP | NM_006402 | 3,68E-05 | -3,33628 |
| 3065480 | NAPEPLD | NM_001122838 | 2,44E-07 | -3,31992 |
| 3761441 | HOXB8 | NM_024016 | 2,38E-05 | -3,31953 |
| 3884830 | PPP1R16B | NM_015568 | 1,04E-04 | -3,31161 |
| 3522327 | SLC15A1 | NM_005073 | 7,59E-05 | -3,30027 |
| 2592005 | HIBCH | NM_014362 | 6,43E-05 | -3,28096 |
| 3368304 | WT1 | NM_024424 | 5,20E-06 | -3,27865 |
| 3744463 | MYH10 | NM_005964 | 1,55E-04 | -3,27238 |
| 3351200 | TMPRSS4 | NM_019894 | 7,63E-08 | -3,2704 |
| 2678714 | FHIT | ENST00000341848 | 3,52E-06 | -3,26645 |
| 3235373 | DHTKD1 | NM_018706 | 8,53E-05 | -3,26341 |
| 2644014 | PCCB | NM_001178014 | 1,20E-05 | -3,26113 |
| 3599758 | PAQR5 | NM_001104554 | 5,13E-05 | -3,24822 |
| 2704441 | MECOM | NM_001105077 | 9,09E-04 | -3,24572 |
| 3592304 | SLC28A2 | NM_004212 | 4,88E-05 | -3,24293 |
| 3771215 | ACOX1 | NM_004035 | 1,24E-05 | -3,23077 |
| 3626312 | ALDH1A2 | NM_003888 | 7,51E-04 | -3,21891 |
| 2588889 | LOC100130691 | NR_026966 | 3,07E-05 | -3,21613 |
| 2400655 | RAP1GAP | NM_001145657 | 1,25E-03 | -3,21328 |
| 3190737 | TBC1D13 | NM_018201 | 1,27E-05 | -3,20537 |
| 2672442 | MYL3 | NM_000258 | 1,77E-07 | -3,19858 |
| 3282601 | MPP7 | NM_173496 | 3,63E-04 | -3,18326 |
| 4026798 | L1CAM | NM_000425 | 1,62E-04 | -3,18247 |
| 3344861 | C11orf54 | NM_014039 | 1,41E-03 | -3,1737 |
| 2516834 | HOXD10 | NM_002148 | 5,04E-04 | -3,17172 |
| 3290875 | ANK3 | NM_020987 | 7,45E-04 | -3,16452 |
| 3191352 | NCS1 | NM_014286 | 2,83E-04 | -3,14313 |
| 4021469 | AIFM1 | NM_001130847 | 1,52E-04 | -3,1416 |
| 3920171 | SIM2 | NM_009586 | 3,92E-06 | -3,13889 |
| 3722535 | ARL4D | NM_001661 | 9,32E-04 | -3,13131 |
| 3678542 | C16orf89 | NM_152459 | 8,51E-05 | -3,11337 |
| 2504645 | MYO7B | NM_001080527 | 6,43E-05 | -3,10975 |
| 2873785 | ALDH7A1 | NM_001182 | 7,94E-05 | -3,1058 |
| 3666366 | CDH3 | NM_001793 | 5,49E-06 | -3,09984 |
| 2635998 | TAGLN3 | NM_013259 | 1,16E-06 | -3,09598 |
| 3196034 | C9orf66 | NM_152569 | 6,94E-04 | -3,09172 |
| 3058209 | MAGI2 | NM_012301 | 3,02E-06 | -3,0915 |
| 3762753 | CA10 | NM_001082533 | 6,64E-05 | -3,08965 |
| 2775735 | SCD5 | NM_001037582 | 1,05E-04 | -3,08812 |
| 3838052 | DHDH | NM_014475 | 1,34E-05 | -3,08625 |
| 2349863 | NTNG1 | NM_001113226 | 3,68E-04 | -3,07981 |
| 2587790 | GPR155 | NM_001033045 | 3,00E-04 | -3,07629 |
| 2767972 | GABRA2 | NM_000807 | 6,11E-09 | -3,07489 |
| 2381249 | C1orf115 | NM_024709 | 6,44E-05 | -3,05897 |
| 2713382 | BDH1 | NM_203314 | 3,79E-05 | -3,03192 |
| 3980867 | GJB1 | NM_000166 | 3,78E-06 | -3,01807 |
| 2734421 | ARHGAP24 | NM_001025616 | 3,93E-05 | -3,01014 |
| 3883064 | ACSS2 | NM_001076552 | 1,29E-05 | -3,00535 |
| 2401670 | MYOM3 | NM_152372 | 5,08E-04 | -3,00283 |
| 3351498 | TMEM25 | NM_032780 | 6,46E-06 | -2,99053 |
| 2884845 | GABRB2 | NM_021911 | 9,18E-07 | -2,98475 |
| 2527253 | IGFBP2 | NM_000597 | 4,55E-04 | -2,98205 |
| 3761451 | HOXB9 | NM_024017 | 6,47E-04 | -2,97886 |
| 3203524 | AQP7 | NM_001170 | 2,40E-04 | -2,97621 |
| 2470838 | MYCN | NM_005378 | 3,38E-06 | -2,96272 |
| 2437363 | PKLR | NM_000298 | 1,31E-03 | -2,9553 |
| 2550325 | OXER1 | NM_148962 | 3,59E-05 | -2,95489 |
| 3113280 | DEPDC6 | NM_022783 | 5,26E-04 | -2,94752 |
| 4022183 | HS6ST2 | NM_001077188 | 3,56E-08 | -2,9463 |
| 2340078 | CACHD1 | NM_020925 | 2,51E-04 | -2,94127 |
| 3481890 | ATP12A | NM_001676 | 6,18E-06 | -2,937 |
| 2452724 | PM20D1 | NM_152491 | 6,39E-04 | -2,93547 |
| 2984616 | BRP44L | NM_016098 | 2,24E-05 | -2,92666 |
| 2673181 | PLXNB1 | NM_002673 | 5,61E-09 | -2,92582 |
| 2777333 | PPM1K | NM_152542 | 4,35E-05 | -2,92525 |
| 2376168 | NFASC | NM_001005388 | 2,16E-08 | -2,9155 |
| 3262535 | GSTO2 | NM_183239 | 1,26E-05 | -2,90609 |
| 2735815 | FAM190A | NM_001145065 | 1,08E-06 | -2,90346 |
| 2535976 | AGXT | NM_000030 | 5,87E-06 | -2,89683 |
| 2675150 | HYAL1 | NM_007312 | 9,66E-05 | -2,89554 |
| 2914820 | BCKDHB | NM_183050 | 7,22E-08 | -2,89549 |
| 2437118 | MUC1 | NM_002456 | 3,63E-04 | -2,89197 |
| 3274898 | TUBAL3 | NM_024803 | 9,19E-05 | -2,88967 |
| 3634811 | CTSH | NM_004390 | 2,30E-05 | -2,88585 |
| 2451958 | PLEKHA6 | NM_014935 | 2,70E-07 | -2,88456 |
| 2361488 | RHBG | NM_020407 | 4,36E-05 | -2,88292 |
| 3896621 | FERMT1 | NM_017671 | 7,16E-04 | -2,88179 |
| 2453881 | IRF6 | NM_006147 | 8,19E-04 | -2,8765 |
| 3311157 | OAT | NM_000274 | 5,11E-05 | -2,87565 |
| 3719329 | LHX1 | NM_005568 | 2,01E-09 | -2,87227 |
| 3695157 | CMTM4 | NM_181521 | 3,82E-05 | -2,86415 |
| 3602004 | SCAMP5 | NM_138967 | 1,08E-05 | -2,85701 |
| 3414739 | METTL7A | NM_014033 | 2,63E-04 | -2,8511 |
| 3293840 | SPOCK2 | NM_014767 | 1,60E-05 | -2,82334 |
| 3265565 | ATRNL1 | NM_207303 | 3,40E-05 | -2,81879 |
| 3894365 | C20orf54 | NM_033409 | 5,68E-06 | -2,81853 |
| 3923426 | AGPAT3 | NM_020132 | 1,29E-05 | -2,8149 |
| 2776670 | MAPK10 | NM_138982 | 1,89E-06 | -2,81126 |
| 3658980 | GPT2 | NM_001142466 | 8,92E-04 | -2,80997 |
| 3549708 | SERPINA4 | NM_006215 | 2,80E-06 | -2,80657 |
| 3221633 | HDHD3 | NM_031219 | 1,15E-05 | -2,80648 |
| 3683502 | GP2 | NM_001007240 | 6,13E-05 | -2,8014 |
| 2584787 | COBLL1 | NM_014900 | 3,76E-04 | -2,79698 |
| 2417390 | WLS | NM_024911 | 2,22E-04 | -2,79407 |
| 2612401 | BTD | NM_000060 | 6,78E-05 | -2,7906 |
| 2401609 | HMGCL | NM_000191 | 2,37E-05 | -2,79015 |
| 3490892 | OLFM4 | NM_006418 | 3,20E-04 | -2,77646 |
| 2676471 | TMEM110 | NM_198563 | 8,26E-07 | -2,77343 |
| 2342576 | ACADM | NM_000016 | 7,97E-04 | -2,77135 |
| 3348773 | C11orf52 | NM_080659 | 2,98E-04 | -2,77046 |
| 3835777 | BCAM | NM_005581 | 6,52E-05 | -2,76938 |
| 3210013 | TRPM6 | NM_017662 | 2,18E-04 | -2,76394 |
| 3981959 | SLC16A2 | NM_006517 | 2,66E-04 | -2,75794 |
| 3430462 | BTBD11 | NM_001018072 | 2,63E-06 | -2,75549 |
| 3329099 | GYLTL1B | NM_152312 | 1,18E-04 | -2,75089 |
| 2672190 | LRRC2 | NM_024512 | 1,67E-06 | -2,746 |
| 3120682 | MFSD3 | NM_138431 | 3,05E-06 | -2,74402 |
| 3589905 | IVD | NM_002225 | 7,77E-06 | -2,73353 |
| 2353988 | FAM46C | NM_017709 | 7,04E-09 | -2,7252 |
| 3852735 | PTGER1 | NM_000955 | 3,17E-07 | -2,725 |
| 3318666 | SMPD1 | NM_000543 | 1,15E-06 | -2,71759 |
| 3432678 | TPCN1 | NM_001143819 | 1,75E-04 | -2,71603 |
| 3018605 | SLC26A4 | NM_000441 | 5,19E-06 | -2,71514 |
| 3446297 | RERGL | NM_024730 | 1,14E-03 | -2,71432 |
| 3521816 | OXGR1 | NM_080818 | 5,52E-04 | -2,71241 |
| 2336539 | ZYG11A | NM_001004339 | 1,65E-04 | -2,71207 |
| 3424030 | C12orf64 | NM_173591 | 9,57E-05 | -2,70768 |
| 3948953 | PPARA | NM_005036 | 5,72E-05 | -2,70347 |
| 3682182 | ABCC6 | NM_001171 | 6,19E-04 | -2,70186 |
| 3461341 | CPM | NM_001874 | 3,66E-04 | -2,68621 |
| 2673547 | SLC26A6 | NM_001040454 | 7,99E-06 | -2,68046 |
| 3357303 | ACAD8 | NM_014384 | 4,63E-06 | -2,67553 |
| 3228813 | SARDH | NM_001134707 | 8,15E-05 | -2,67071 |
| 2623426 | ABHD14A | NM_015407 | 2,88E-06 | -2,66861 |
| 3726211 | PDK2 | NM_002611 | 8,33E-04 | -2,66758 |
| 3760957 | SCRN2 | NM_001145023 | 3,25E-05 | -2,66634 |
| 3011675 | ZNF804B | NM_181646 | 8,78E-04 | -2,66551 |
| 3473480 | FBXO21 | NM_033624 | 6,83E-05 | -2,65796 |
| 2387711 | FMN2 | NM_020066 | 1,41E-06 | -2,65209 |
| 2509988 | LYPD6B | NM_177964 | 7,25E-05 | -2,6505 |
| 2340350 | DNAJC6 | NM_014787 | 2,71E-04 | -2,64061 |
| 2680819 | SUCLG2 | NM_003848 | 8,05E-05 | -2,63941 |
| 3408733 | RASSF8 | NM_007211 | 1,13E-07 | -2,63703 |
| 2417362 | DIRAS3 | NM_004675 | 5,08E-08 | -2,63122 |
| 3032647 | DPP6 | NM_001039350 | 1,27E-04 | -2,616 |
| 3235255 | ECHDC3 | NM_024693 | 8,46E-04 | -2,6131 |
| 2864796 | ACOT12 | NM_130767 | 4,30E-07 | -2,6008 |
| 2488596 | EMX1 | NM_004097 | 1,65E-08 | -2,59983 |
| 2622696 | SEMA3B | NM_004636 | 6,89E-06 | -2,59353 |
| 3223776 | C5 | NM_001735 | 3,16E-04 | -2,59165 |
| 2939593 | PECI | NR_028588 | 7,95E-05 | -2,58683 |
| 3825523 | SLC25A42 | NM_178526 | 1,18E-03 | -2,57438 |
| 2638676 | EAF2 | NM_018456 | 6,95E-05 | -2,57408 |
| 3266408 | EMX2 | NM_004098 | 4,60E-04 | -2,57332 |
| 2542795 | SDC1 | NM_001006946 | 4,80E-04 | -2,56637 |
| 3310041 | FGFR2 | NM_000141 | 1,49E-03 | -2,56178 |
| 3432798 | SDSL | NM_138432 | 9,39E-05 | -2,56101 |
| 2333599 | IPO13 | NM_014652 | 2,45E-06 | -2,55749 |
| 3452690 | RAPGEF3 | NM_001098532 | 9,76E-09 | -2,55034 |
| 2781736 | CFI | NM_000204 | 5,80E-04 | -2,55026 |
| 3563814 | L2HGDH | NM_024884 | 3,07E-05 | -2,54655 |
| 2503109 | EPB41L5 | NM_020909 | 8,00E-07 | -2,54549 |
| 2666147 | THRB | NM_001128177 | 1,35E-03 | -2,54433 |
| 2734629 | PTPN13 | NM_080683 | 1,07E-04 | -2,54266 |
| 4026925 | RENBP | NM_002910 | 5,86E-05 | -2,53758 |
| 3923537 | C21orf33 | NM_004649 | 4,83E-04 | -2,536 |
| 2994835 | CHN2 | NM_004067 | 2,10E-05 | -2,5332 |
| 3103293 | RDH10 | NM_172037 | 6,43E-05 | -2,52109 |
| 3427032 | AMDHD1 | NM_152435 | 6,18E-04 | -2,52004 |
| 2788003 | GYPA | NM_002099 | 2,29E-04 | -2,51394 |
| 2531589 | ITM2C | NM_030926 | 1,16E-03 | -2,5067 |
| 3720402 | ERBB2 | NM_001005862 | 2,62E-05 | -2,5047 |
| 3322904 | TMEM86A | NM_153347 | 4,81E-06 | -2,50157 |
| 3482572 | WASF3 | NM_006646 | 1,60E-07 | -2,4963 |
| 3299705 | PANK1 | NM_148977 | 5,32E-04 | -2,4946 |
| 3768791 | ABCA6 | NM_080284 | 1,02E-06 | -2,48922 |
| 3168841 | GRHPR | NM_012203 | 3,07E-04 | -2,48687 |
| 3341362 | AQP11 | NM_173039 | 5,10E-06 | -2,4818 |
| 3364306 | SOX6 | NM_017508 | 3,23E-04 | -2,48137 |
| 2924330 | TPD52L1 | NM_001003395 | 5,35E-04 | -2,47953 |
| 3527514 | PNP | NM_000270 | 5,81E-05 | -2,47752 |
| 3417184 | SUOX | NM_000456 | 7,84E-06 | -2,47689 |
| 2514745 | MYO3B | NM_138995 | 3,67E-04 | -2,47412 |
| 3179646 | SUSD3 | NM_145006 | 1,99E-04 | -2,46494 |
| 3073013 | PODXL | NM_001018111 | 1,43E-03 | -2,46267 |
| 3729419 | CA4 | NM_000717 | 1,37E-06 | -2,46187 |
| 3553389 | AMN | NM_030943 | 1,27E-03 | -2,45784 |
| 2440744 | NR1I3 | NM_001077482 | 8,68E-06 | -2,45555 |
| 3707041 | SMTNL2 | NM_001114974 | 3,61E-05 | -2,44868 |
| 2425212 | DBT | NM_001918 | 7,97E-07 | -2,44623 |
| 3708938 | ATP1B2 | NM_001678 | 2,47E-06 | -2,44536 |
| 3295376 | ZNF503 | NM_032772 | 1,02E-05 | -2,44294 |
| 3305801 | SORCS1 | NM_052918 | 3,23E-06 | -2,44075 |
| 2622196 | APEH | NM_001640 | 3,33E-05 | -2,43817 |
| 3061964 | PON3 | NM_000940 | 9,04E-05 | -2,43303 |
| 3809621 | FECH | NM_001012515 | 6,93E-05 | -2,43285 |
| 3599495 | CORO2B | NM_006091 | 9,02E-12 | -2,42848 |
| 3199511 | FREM1 | NM_144966 | 6,76E-05 | -2,42801 |
| 3031181 | ATP6V0E2 | NM_145230 | 1,00E-05 | -2,42125 |
| 3750842 | SGK494 | NM_001174103 | 1,82E-04 | -2,41427 |
| 3109687 | GRHL2 | NM_024915 | 1,83E-04 | -2,41059 |
| 2513471 | SCN2A | NM_021007 | 3,68E-06 | -2,4061 |
| 2678367 | PDHB | NR_033384 | 1,72E-04 | -2,4009 |
| 3352040 | PDZD3 | NR_033122 | 4,91E-06 | -2,39075 |
| 3417531 | COQ10A | NM_144576 | 3,14E-05 | -2,39022 |
| 3863669 | CEACAM1 | NM_001712 | 2,99E-04 | -2,38728 |
| 2353477 | ATP1A1 | NM_000701 | 1,80E-06 | -2,3852 |
| 2347502 | ABCD3 | NM_002858 | 1,77E-04 | -2,38481 |
| 3895330 | SLC4A11 | NM_032034 | 1,40E-04 | -2,37196 |
| 4000456 | ASB9 | NM_024087 | 4,81E-07 | -2,3703 |
| 2956438 | MUT | NM_000255 | 1,85E-04 | -2,36852 |
| 3158581 | SLC39A4 | NM_017767 | 1,96E-05 | -2,36731 |
| 3308397 | HSPA12A | NM_025015 | 4,42E-04 | -2,36279 |
| 2898499 | ALDH5A1 | NM_170740 | 7,61E-05 | -2,36182 |
| 2707824 | MCCC1 | NM_020166 | 7,71E-05 | -2,36173 |
| 2721633 | SOD3 | NM_003102 | 4,88E-05 | -2,35475 |
| 2427007 | SORT1 | NM_002959 | 7,01E-05 | -2,34751 |
| 3735089 | LOC643008 | NR_028439 | 2,22E-04 | -2,34728 |
| 3544346 | DLST | NM_001933 | 2,29E-04 | -2,34274 |
| 3793827 | CNDP1 | NM_032649 | 2,12E-06 | -2,34019 |
| 3734903 | LLGL2 | NM_001031803 | 5,29E-08 | -2,32996 |
| 2559386 | SFXN5 | NM_144579 | 1,55E-05 | -2,32665 |
| 3815116 | PALM | NM_002579 | 2,00E-05 | -2,32471 |
| 2769063 | USP46 | NM_022832 | 4,25E-09 | -2,32441 |
| 2585129 | GALNT3 | NM_004482 | 7,19E-05 | -2,32406 |
| 3824963 | PGPEP1 | NM_017712 | 3,43E-04 | -2,3229 |
| 2715016 | FGFR3 | NM_000142 | 9,46E-04 | -2,32279 |
| 3568603 | GPX2 | NM_002083 | 8,95E-05 | -2,32264 |
| 2623611 | GLYCTK | NM_145262 | 5,71E-06 | -2,32217 |
| 3278305 | BEND7 | NM_152751 | 3,79E-04 | -2,32173 |
| 2317686 | AJAP1 | NM_018836 | 1,17E-04 | -2,31866 |
| 2562233 | RETSAT | NM_017750 | 2,46E-04 | -2,31824 |
| 3418303 | PIP4K2C | NM_024779 | 4,96E-07 | -2,3142 |
| 3740367 | SLC43A2 | NM_152346 | 2,73E-05 | -2,31274 |
| 2994981 | PRR15 | NM_175887 | 2,87E-05 | -2,31126 |
| 3018375 | PRKAR2B | NM_002736 | 1,23E-04 | -2,30996 |
| 2408929 | ZMYND12 | NM_032257 | 7,15E-05 | -2,30652 |
| 3970833 | PDHA1 | NM_001173454 | 2,52E-04 | -2,30082 |
| 3662086 | MT4 | NM_032935 | 6,78E-04 | -2,29867 |
| 2442424 | ILDR2 | NM_199351 | 3,60E-04 | -2,29818 |
| 2381264 | MOSC2 | NM_017898 | 1,85E-05 | -2,29785 |
| 3996755 | BRCC3 | ENST00000454406 | 2,90E-04 | -2,29356 |
| 3150289 | SAMD12 | NM_207506 | 4,66E-04 | -2,28105 |
| 3173673 | PIP5K1B | NM_003558 | 1,30E-03 | -2,28029 |
| 3124388 | FAM167A | NM_053279 | 2,44E-07 | -2,27838 |
| 3473524 | NOS1 | NM_000620 | 8,75E-10 | -2,27481 |
| 2361384 | SLC25A44 | NM_014655 | 1,13E-04 | -2,27455 |
| 2914070 | MYO6 | NM_004999 | 4,86E-04 | -2,27249 |
| 2380440 | SPATA17 | NM_138796 | 7,63E-05 | -2,2686 |
| 2983142 | PARK2 | NM_004562 | 6,07E-07 | -2,26675 |
| 2453065 | C1orf116 | NM_023938 | 6,15E-06 | -2,26407 |
| 3903361 | AHCY | NM_000687 | 3,03E-04 | -2,26249 |
| 3432030 | ACAD10 | NM_001136538 | 9,04E-06 | -2,26162 |
| 3997738 | ARSD | NM_001669 | 2,24E-06 | -2,25633 |
| 2727226 | PDGFRA | NM_006206 | 1,79E-04 | -2,25633 |
| 3959613 | FOXRED2 | NM_024955 | 1,90E-04 | -2,25582 |
| 2608309 | LRRN1 | NM_020873 | 3,41E-09 | -2,25172 |
| 3452417 | SLC38A4 | NM_018018 | 3,81E-04 | -2,25046 |
| 3074577 | FAM180A | NM_205855 | 7,72E-06 | -2,25042 |
| 3029016 | TMEM139 | NM_153345 | 2,78E-04 | -2,24292 |
| 3967018 | ARSF | NM_004042 | 4,88E-04 | -2,24239 |
| 3136178 | PLAG1 | NM_002655 | 2,19E-07 | -2,24226 |
| 3219788 | EPB41L4B | NM_019114 | 2,80E-05 | -2,23784 |
| 3565524 | GCH1 | NM_000161 | 1,81E-04 | -2,23433 |
| 2350714 | SYPL2 | NM_001040709 | 8,39E-05 | -2,23328 |
| 3957207 | GATSL3 | NM_001037666 | 3,72E-04 | -2,23091 |
| 2454661 | TMEM206 | NM_018252 | 2,88E-05 | -2,23046 |
| 2814642 | MCCC2 | NM_022132 | 1,76E-04 | -2,22962 |
| 3315380 | SPRN | NM_001012508 | 6,35E-04 | -2,22753 |
| 2443120 | DPT | NM_001937 | 7,20E-04 | -2,22666 |
| 2831664 | SLC4A9 | NM_031467 | 1,24E-03 | -2,22607 |
| 2664395 | HACL1 | NM_012260 | 4,82E-05 | -2,22097 |
| 3056163 | MLXIPL | NM_032951 | 8,04E-04 | -2,21557 |
| 2911372 | BAG2 | NM_004282 | 7,12E-05 | -2,21098 |
| 2444529 | SERPINC1 | NM_000488 | 1,21E-03 | -2,20911 |
| 3976848 | HDAC6 | NM_006044 | 1,31E-04 | -2,20745 |
| 3696226 | ESRP2 | NM_024939 | 2,22E-04 | -2,20416 |
| 2590017 | ZNF385B | NM_152520 | 1,16E-03 | -2,20344 |
| 3337196 | NDUFV1 | NM_007103 | 1,87E-04 | -2,20167 |
| 3667652 | MARVELD3 | NM_001017967 | 6,39E-05 | -2,20063 |
| 2443335 | SLC19A2 | NM_006996 | 8,76E-04 | -2,19771 |
| 2467211 | COLEC11 | NM_199235 | 1,91E-05 | -2,19617 |
| 3529309 | DHRS4 | NM_021004 | 1,98E-04 | -2,19609 |
| 3724969 | PNPO | NM_018129 | 5,08E-05 | -2,1948 |
| 3731826 | PRKCA | NM_002737 | 1,09E-03 | -2,1939 |
| 2620018 | C3orf23 | NM_173826 | 1,43E-06 | -2,19271 |
| 3590853 | CAPN3 | NR_027911 | 2,07E-04 | -2,19236 |
| 3213847 | SHC3 | NM_016848 | 6,23E-07 | -2,19076 |
| 2398820 | PADI2 | NM_007365 | 3,76E-04 | -2,18912 |
| 2329041 | KIAA1522 | NM_020888 | 1,44E-06 | -2,1878 |
| 3610958 | IGF1R | NM_000875 | 1,44E-03 | -2,18716 |
| 3569401 | RDH11 | NM_016026 | 2,31E-04 | -2,18244 |
| 3541073 | MPP5 | NM_022474 | 6,80E-04 | -2,17022 |
| 2527895 | PLCD4 | NM_032726 | 2,44E-05 | -2,1647 |
| 3824983 | PGPEP1 | NM_017712 | 1,54E-03 | -2,16423 |
| 3433796 | PEBP1 | NM_002567 | 1,99E-05 | -2,16406 |
| 2516879 | HOXD8 | NM_019558 | 1,09E-05 | -2,16325 |
| 3998632 | PNPLA4 | NM_004650 | 3,53E-04 | -2,16175 |
| 2671101 | ANO10 | NM_018075 | 8,06E-06 | -2,15939 |
| 3948259 | PRR5-ARHGAP8 | NM_181334 | 3,42E-04 | -2,15802 |
| 3714068 | ALDH3A2 | NM_001031806 | 8,52E-04 | -2,15239 |
| 2413032 | ECHDC2 | NM_018281 | 3,52E-04 | -2,15171 |
| 2358949 | CGN | NM_020770 | 1,86E-07 | -2,15015 |
| 3490073 | FAM124A | BC051771 | 3,53E-08 | -2,14665 |
| 3446868 | LDHB | NM_001174097 | 9,14E-04 | -2,14416 |
| 2915571 | MRAP2 | NM_138409 | 7,43E-07 | -2,14337 |
| 3734648 | SLC16A5 | NM_004695 | 1,84E-05 | -2,14064 |
| 4015397 | TSPAN6 | NM_003270 | 1,92E-04 | -2,13658 |
| 3261971 | CNNM2 | NM_017649 | 3,95E-05 | -2,134 |
| 2792420 | TMEM192 | NM_001100389 | 1,00E-04 | -2,13096 |
| 3149528 | TRPS1 | NM_014112 | 1,35E-03 | -2,1294 |
| 3339971 | PLEKHB1 | NM_021200 | 6,22E-04 | -2,12917 |
| 2487696 | PCYOX1 | NM_016297 | 4,78E-05 | -2,12662 |
| 3333711 | SLC3A2 | NM_001012661 | 5,74E-04 | -2,12603 |
| 3077128 | TRPV5 | NM_019841 | 2,94E-04 | -2,1228 |
| 2757427 | LETM1 | NM_012318 | 8,81E-06 | -2,12274 |
| 3378344 | CTSF | NM_003793 | 1,09E-03 | -2,12092 |
| 3129588 | KIF13B | NM_015254 | 1,89E-05 | -2,11773 |
| 3288845 | AGAP8 | NM_001077686 | 2,93E-06 | -2,11114 |
| 2408994 | CLDN19 | NM_001123395 | 7,85E-05 | -2,1094 |
| 2719440 | C1QTNF7 | NM_001135170 | 9,27E-06 | -2,10568 |
| 3845944 | GNG7 | NM_052847 | 2,05E-06 | -2,10329 |
| 4000155 | GPM6B | NM_001001995 | 1,43E-04 | -2,10004 |
| 3529547 | DCAF11 | NM_025230 | 4,18E-06 | -2,09622 |
| 3197318 | AK3 | NM_016282 | 2,62E-05 | -2,0948 |
| 2382970 | EPHX1 | NM_000120 | 2,37E-04 | -2,09306 |
| 2691850 | ILDR1 | NM_175924 | 1,25E-03 | -2,09174 |
| 2653114 | NAALADL2 | NM_207015 | 1,24E-04 | -2,08774 |
| 2800026 | ADAMTS16 | NM_139056 | 7,56E-07 | -2,08504 |
| 2969350 | DDO | NM_003649 | 1,27E-05 | -2,08296 |
| 3761806 | PHB | NM_002634 | 1,16E-06 | -2,07932 |
| 2581349 | CACNB4 | NM_000726 | 4,24E-07 | -2,07731 |
| 2793054 | CBR4 | NM_032783 | 4,19E-04 | -2,07237 |
| 2316245 | PRKCZ | NM_001033582 | 1,16E-04 | -2,07131 |
| 2977949 | EPM2A | NM_005670 | 5,28E-07 | -2,06829 |
| 2517588 | OSBPL6 | NM_032523 | 3,28E-05 | -2,06706 |
| 2394841 | DNAJC11 | NM_018198 | 1,44E-04 | -2,06668 |
| 2511820 | PKP4 | NM_003628 | 8,64E-04 | -2,06452 |
| 3357885 | SIGIRR | NM_001135053 | 4,20E-05 | -2,0621 |
| 3906062 | ZHX3 | NM_015035 | 1,88E-06 | -2,06117 |
| 3728037 | SCPEP1 | NM_021626 | 9,53E-05 | -2,05816 |
| 2582701 | CCDC148 | NM_138803 | 1,37E-04 | -2,04831 |
| 3757329 | JUP | NM_002230 | 2,35E-05 | -2,04756 |
| 3326400 | CAT | NM_001752 | 9,86E-05 | -2,04516 |
| 2926323 | EYA4 | NM_004100 | 7,05E-05 | -2,04419 |
| 3764471 | MTMR4 | NM_004687 | 9,99E-05 | -2,04254 |
| 2442103 | ALDH9A1 | NM_000696 | 2,29E-04 | -2,04065 |
| 2380785 | LYPLAL1 | NM_138794 | 1,67E-04 | -2,03658 |
| 3341155 | CAPN5 | NM_004055 | 2,29E-04 | -2,03233 |
| 3348189 | FDX1 | NM_004109 | 6,33E-05 | -2,03159 |
| 3400034 | WNK1 | NM_018979 | 2,99E-04 | -2,02305 |
| 3962781 | MCAT | NM_173467 | 5,79E-05 | -2,02116 |
| 3987607 | ZCCHC16 | NM_001004308 | 1,10E-05 | -2,0155 |
| 3255220 | GHITM | NM_014394 | 1,55E-04 | -2,01519 |
| 2734352 | C4orf12 | AY250185 | 3,32E-05 | -2,0134 |
| 3372368 | MTCH2 | NM_014342 | 1,94E-04 | -2,01256 |
| 3361420 | OVCH2 | NM_198185 | 5,11E-06 | -2,00823 |
| 2677723 | ARHGEF3 | NM_001128615 | 4,01E-04 | -2,00738 |
| 2539607 | MBOAT2 | NM_138799 | 1,69E-05 | -2,00627 |
| 2768145 | COMMD8 | NM_017845 | 1,70E-04 | -2,00107 |
| 2973168 | ECHDC1 | NM_001002030 | 5,28E-04 | -2,00061 |
| 3901955 | NINL | NM_025176 | 5,55E-06 | -1,99964 |
| 3919101 | KCNE2 | NM_172201 | 5,34E-05 | -1,9906 |
| 3190366 | SLC27A4 | NM_005094 | 9,70E-08 | -1,9906 |
| 3289948 | PCDH15 | NM_001142763 | 1,01E-05 | -1,99021 |
| 3137901 | TTPA | NM_000370 | 5,14E-05 | -1,98877 |
| 4013828 | HMGN5 | NM_030763 | 2,01E-04 | -1,98862 |
| 2750198 | NPY5R | NM_006174 | 1,20E-04 | -1,98762 |
| 2516912 | HOXD4 | NM_014621 | 1,17E-04 | -1,98743 |
| 2411642 | AGBL4 | NM_032785 | 1,43E-07 | -1,98243 |
| 3638760 | IDH2 | NM_002168 | 1,80E-04 | -1,97918 |
| 3611049 | LRRC28 | NM_144598 | 6,83E-05 | -1,97502 |
| 3748026 | TOM1L2 | NM_001082968 | 7,28E-06 | -1,97456 |
| 3837895 | SPHK2 | NM_020126 | 3,97E-08 | -1,96995 |
| 3887479 | EYA2 | NM_005244 | 6,36E-04 | -1,96944 |
| 3502570 | LAMP1 | NM_005561 | 2,81E-07 | -1,96941 |
| 3558118 | DHRS1 | NM_001136050 | 3,28E-05 | -1,96933 |
| 3750625 | POLDIP2 | NM_015584 | 1,05E-07 | -1,96869 |
| 3082990 | MYOM2 | NM_003970 | 2,28E-06 | -1,96741 |
| 4012511 | NAP1L2 | NM_021963 | 9,04E-04 | -1,96695 |
| 3135452 | ATP6V1H | NM_015941 | 2,25E-04 | -1,96549 |
| 3526454 | GRTP1 | NM_024719 | 1,04E-04 | -1,96529 |
| 3806253 | ATP5A1 | NM_001001937 | 1,32E-03 | -1,9643 |
| 2698565 | TFDP2 | NM_006286 | 1,07E-04 | -1,96107 |
| 2610972 | SYN2 | NM_133625 | 3,52E-05 | -1,95275 |
| 3361621 | RIC3 | NM_024557 | 9,76E-04 | -1,95068 |
| 4009288 | HSD17B10 | NM_004493 | 1,28E-04 | -1,95058 |
| 2596201 | NDUFS1 | NM_005006 | 6,67E-04 | -1,94882 |
| 2673509 | UQCRC1 | NM_003365 | 6,46E-04 | -1,94813 |
| 3523156 | TMTC4 | NM_032813 | 1,28E-05 | -1,9344 |
| 3127610 | PEBP4 | NM_144962 | 5,87E-05 | -1,93398 |
| 2781387 | AGXT2L1 | NM_031279 | 4,69E-04 | -1,93074 |
| 3261820 | TRIM8 | NM_030912 | 3,17E-05 | -1,92801 |
| 2666103 | NKIRAS1 | NM_020345 | 1,80E-04 | -1,92425 |
| 2574646 | BIN1 | NM_139343 | 1,07E-04 | -1,92342 |
| 3929705 | DNAJC28 | NM_017833 | 1,68E-04 | -1,92286 |
| 2577028 | NCKAP5 | NM_207363 | 1,47E-03 | -1,9192 |
| 2317434 | TPRG1L | NM_182752 | 1,52E-03 | -1,91811 |
| 3676165 | HAGH | NM_001040427 | 7,00E-04 | -1,91719 |
| 2674526 | NICN1 | NM_032316 | 6,27E-07 | -1,91558 |
| 2617276 | CTDSPL | NM_001008392 | 1,05E-03 | -1,9151 |
| 3281358 | C10orf67 | NM_153714 | 8,93E-06 | -1,91478 |
| 2409770 | TMEM53 | NM_024587 | 1,44E-03 | -1,91383 |
| 3415148 | ACVR1B | NM_004302 | 3,62E-07 | -1,90996 |
| 4009893 | FAM104B | NM_001166703 | 9,62E-06 | -1,90821 |
| 3280787 | C10orf140 | NM_207371 | 5,68E-08 | -1,9053 |
| 3489350 | CDADC1 | NM_030911 | 8,24E-04 | -1,90447 |
| 3335571 | OVOL1 | NM_004561 | 2,83E-04 | -1,90201 |
| 3755323 | PCGF2 | NM_007144 | 1,20E-07 | -1,89728 |
| 3980745 | FOXO4 | NM_005938 | 6,87E-07 | -1,89013 |
| 3727499 | TOM1L1 | AB065085 | 8,92E-04 | -1,88982 |
| 3797032 | EPB41L3 | NM_012307 | 8,21E-04 | -1,88934 |
| 3568616 | RAB15 | NM_198686 | 3,04E-04 | -1,88611 |
| 2336585 | SCP2 | NM_002979 | 8,31E-05 | -1,8851 |
| 3516639 | PCDH9 | NM_203487 | 1,05E-05 | -1,88469 |
| 3185593 | BSPRY | NM_017688 | 5,60E-04 | -1,88392 |
| 2530425 | COL4A3 | NM_000091 | 3,32E-05 | -1,88064 |
| 2930753 | C6orf72 | AY358952 | 4,31E-04 | -1,87999 |
| 2766788 | RBM47 | NM_001098634 | 1,42E-04 | -1,87552 |
| 2829864 | LOC153328 | NM_145282 | 3,41E-07 | -1,87461 |
| 3413212 | SLC48A1 | NM_017842 | 1,06E-06 | -1,87361 |
| 3294854 | CAMK2G | NM_172171 | 3,25E-05 | -1,87271 |
| 3624448 | GNB5 | NM_006578 | 1,72E-06 | -1,8696 |
| 3504791 | EFHA1 | NM_152726 | 4,58E-04 | -1,86957 |
| 2326157 | FAM54B | NM_019557 | 9,63E-06 | -1,86833 |
| 3482845 | RASL11A | NM_206827 | 3,32E-05 | -1,86757 |
| 2675836 | ABHD14B | NM_032750 | 2,23E-07 | -1,86345 |
| 2558976 | MCEE | NM_032601 | 2,18E-04 | -1,86228 |
| 2682729 | PDZRN3 | NM_015009 | 6,39E-06 | -1,86225 |
| 3768703 | ABCA9 | NM_080283 | 1,32E-05 | -1,86191 |
| 3910980 | BMP7 | NM_001719 | 7,57E-09 | -1,86085 |
| 3354896 | DDX25 | NM_013264 | 3,74E-07 | -1,84956 |
| 3636391 | HOMER2 | NM_199330 | 2,41E-04 | -1,84944 |
| 3309383 | PRDX3 | NM_006793 | 6,25E-05 | -1,84916 |
| 3687803 | SEPHS2 | NM_012248 | 1,15E-03 | -1,84872 |
| 3741171 | KIAA0664 | NM_015229 | 2,01E-04 | -1,84831 |
| 2367843 | DARS2 | NM_018122 | 2,72E-05 | -1,84622 |
| 3032243 | GALNT11 | NM_022087 | 3,02E-04 | -1,84499 |
| 3750662 | VTN | NM_000638 | 3,77E-04 | -1,84361 |
| 3339311 | LRTOMT | NM_001145309 | 3,67E-07 | -1,84328 |
| 3708919 | SHBG | NM_001040 | 7,69E-04 | -1,84068 |
| 2622239 | RNF123 | NM_022064 | 1,16E-05 | -1,8383 |
| 2527672 | PNKD | NM_015488 | 1,23E-04 | -1,83705 |
| 3226661 | ZER1 | NM_006336 | 5,19E-05 | -1,83303 |
| 2957384 | GSTA5 | NM_153699 | 7,96E-05 | -1,82972 |
| 3851293 | ZNF44 | NM_001164276 | 2,17E-05 | -1,82918 |
| 3226844 | CRAT | NR_028048 | 4,76E-04 | -1,82859 |
| 3340269 | POLD3 | NM_006591 | 8,63E-04 | -1,82733 |
| 3400236 | B4GALNT3 | NM_173593 | 9,83E-04 | -1,82663 |
| 3707431 | KIF1C | NM_006612 | 9,67E-08 | -1,82578 |
| 2949352 | C6orf27 | NM_025258 | 6,82E-06 | -1,82575 |
| 2971801 | MAN1A1 | NM_005907 | 1,93E-04 | -1,82375 |
| 2673773 | SLC25A20 | NM_000387 | 1,50E-05 | -1,82234 |
| 3356328 | ADAMTS15 | NM_139055 | 5,07E-04 | -1,82142 |
| 3133345 | SLC20A2 | NM_006749 | 1,84E-04 | -1,82037 |
| 2334986 | CYP4X1 | NM_178033 | 8,25E-04 | -1,8181 |
| 3761378 | HOXB5 | NM_002147 | 6,53E-04 | -1,81651 |
| 2524016 | PARD3B | NM_152526 | 8,54E-06 | -1,81476 |
| 3310413 | ATE1 | NM_001001976 | 9,24E-04 | -1,81405 |
| 2959197 | LGSN | NM_016571 | 1,03E-06 | -1,81323 |
| 3706842 | CYB5D2 | NM_144611 | 2,00E-04 | -1,81065 |
| 2695453 | CPNE4 | NM_130808 | 4,47E-05 | -1,80803 |
| 3985260 | GPRASP1 | NM_014710 | 6,60E-04 | -1,80634 |
| 3300242 | CPEB3 | NM_014912 | 3,65E-05 | -1,80388 |
| 2677853 | IL17RD | NM_017563 | 1,33E-03 | -1,80049 |
| 2971014 | TSPYL1 | NM_003309 | 1,02E-04 | -1,79976 |
| 2756831 | SLC26A1 | NM_213613 | 6,28E-04 | -1,79847 |
| 3737697 | BAIAP2 | NM_006340 | 4,11E-04 | -1,79822 |
| 3758606 | SOST | NM_025237 | 6,30E-05 | -1,7975 |
| 3992546 | VGLL1 | NM_016267 | 2,84E-04 | -1,79713 |
| 3384704 | DLG2 | NM_001142699 | 8,02E-04 | -1,79705 |
| 3662139 | MT1E | NM_175617 | 6,04E-04 | -1,79423 |
| 3883690 | EPB41L1 | NM_012156 | 1,11E-05 | -1,79063 |
| 2903488 | HSD17B8 | NM_014234 | 1,13E-03 | -1,78884 |
| 2609904 | OGG1 | NM_016819 | 2,94E-06 | -1,78759 |
| 2318257 | ESPN | NM_031475 | 3,82E-04 | -1,78677 |
| 3872053 | PEG3 | NM_006210 | 9,94E-05 | -1,78609 |
| 3226253 | FAM102A | NM_001035254 | 1,04E-03 | -1,78088 |
| 2933522 | GTF2H5 | NM_207118 | 2,99E-04 | -1,78001 |
| 2516953 | HOXD3 | NM_006898 | 3,95E-04 | -1,77965 |
| 2939298 | SLC22A23 | NM_015482 | 3,25E-05 | -1,77907 |
| 3706700 | CTNS | NM_004937 | 2,77E-05 | -1,77727 |
| 2322004 | SLC25A34 | NM_207348 | 5,48E-04 | -1,77016 |
| 2443305 | C1orf114 | BC026073 | 1,58E-06 | -1,76867 |
| 3733911 | SSTR2 | NM_001050 | 4,36E-08 | -1,76669 |
| 3379777 | MRGPRF | NM_001098515 | 3,06E-07 | -1,76406 |
| 3716783 | RAB11FIP4 | NM_032932 | 7,79E-06 | -1,76381 |
| 2363525 | NDUFS2 | NM_004550 | 9,01E-05 | -1,76349 |
| 3174967 | RORB | NM_006914 | 5,98E-04 | -1,75964 |
| 3637818 | NTRK3 | NM_001007156 | 4,56E-05 | -1,75913 |
| 2792459 | GK3P | NR_026575 | 1,37E-03 | -1,75905 |
| 3689981 | MYLK3 | NM_182493 | 1,33E-05 | -1,7574 |
| 3918098 | C21orf119 | NR_026845 | 1,24E-03 | -1,75574 |
| 3325634 | WIT1 | NR_023920 | 1,09E-04 | -1,75425 |
| 2324097 | PINK1 | NM_032409 | 1,46E-05 | -1,75381 |
| 3473586 | KSR2 | NM_173598 | 3,84E-05 | -1,75335 |
| 3718236 | TMEM132E | NM_207313 | 9,61E-05 | -1,75332 |
| 2396201 | CASZ1 | NM_001079843 | 3,11E-06 | -1,75302 |
| 3188514 | CRB2 | NM_173689 | 6,14E-06 | -1,7527 |
| 2607923 | CNTN4 | NM_175607 | 1,65E-06 | -1,75233 |
| 3557756 | NRL | NM_006177 | 1,02E-05 | -1,75174 |
| 2316953 | PRDM16 | NM_022114 | 3,74E-04 | -1,74982 |
| 3863380 | GRIK5 | NM_002088 | 1,63E-04 | -1,74902 |
| 3813604 | ZADH2 | NM_175907 | 9,74E-06 | -1,74778 |
| 3755934 | ORMDL3 | NM_139280 | 1,02E-06 | -1,7469 |
| 3392996 | SIK3 | NM_025164 | 5,38E-06 | -1,74552 |
| 3317352 | KCNQ1 | NM_000218 | 1,22E-04 | -1,74433 |
| 3039247 | DGKB | NM_004080 | 1,42E-03 | -1,74395 |
| 3358742 | TOLLIP | NM_019009 | 7,27E-04 | -1,74391 |
| 2394588 | ICMT | NM_012405 | 5,78E-06 | -1,74365 |
| 2925237 | LAMA2 | NM_000426 | 3,36E-04 | -1,7433 |
| 3622436 | SLC30A4 | NM_013309 | 5,77E-04 | -1,74295 |
| 2622095 | TCTA | NM_022171 | 3,78E-05 | -1,7398 |
| 3696454 | CHTF8 | NM_001039690 | 5,28E-04 | -1,73821 |
| 4045665 | S100A14 | NM_020672 | 8,41E-05 | -1,73767 |
| 2662397 | RPUSD3 | NM_173659 | 2,11E-05 | -1,7367 |
| 2402892 | C1orf172 | NM_152365 | 2,82E-04 | -1,73504 |
| 3501415 | CARKD | NM_018210 | 3,76E-05 | -1,73455 |
| 3939183 | BCR | NM_004327 | 3,53E-08 | -1,73376 |
| 3913775 | CHRNA4 | NM_000744 | 4,29E-06 | -1,73336 |
| 2999948 | OGDH | NM_002541 | 1,35E-03 | -1,73331 |
| 2495758 | C2orf15 | BC021264 | 2,88E-05 | -1,7333 |
| 3740664 | C17orf91 | NR_028502 | 1,38E-03 | -1,73288 |
| 2623249 | TEX264 | NM_001129884 | 3,97E-04 | -1,73065 |
| 3759077 | SLC25A39 | NM_001143780 | 8,64E-04 | -1,72901 |
| 3572041 | KIAA0317 | NM_001039479 | 1,33E-03 | -1,72814 |
| 3527641 | EDDM3A | NM_006683 | 1,62E-05 | -1,72763 |
| 3009229 | POR | NM_000941 | 6,35E-05 | -1,72762 |
| 3494465 | BTF3L1 | NR_026983 | 1,45E-03 | -1,72658 |
| 2626258 | KCTD6 | NM_153331 | 1,39E-03 | -1,72399 |
| 2356818 | BCL9 | NM_004326 | 2,06E-04 | -1,72185 |
| 3911485 | APCDD1L | NM_153360 | 9,88E-07 | -1,72166 |
| 3563861 | CDKL1 | NM_004196 | 1,89E-05 | -1,72142 |
| 3709244 | CHD3 | NM_001005273 | 7,08E-04 | -1,72072 |
| 3750872 | KIAA0100 | NM_014680 | 1,44E-04 | -1,72023 |
| 3950668 | SELO | NM_031454 | 5,99E-06 | -1,72022 |
| 3077072 | TRPV6 | NM_018646 | 6,78E-06 | -1,71887 |
| 3764399 | RNF43 | NM_017763 | 3,79E-04 | -1,71819 |
| 3742384 | SLC25A11 | NM_003562 | 8,58E-04 | -1,71638 |
| 3213120 | GAS1 | NM_002048 | 1,38E-09 | -1,7135 |
| 3383322 | NARS2 | NM_024678 | 3,62E-04 | -1,71284 |
| 2912649 | COL19A1 | NM_001858 | 1,17E-06 | -1,71144 |
| 2327375 | ATPIF1 | NM_178191 | 3,46E-04 | -1,70817 |
| 3741502 | SHPK | NM_013276 | 3,23E-04 | -1,70543 |
| 3939498 | SLC2A11 | NM_030807 | 1,54E-06 | -1,70307 |
| 2510056 | LYPD6 | NM_194317 | 1,41E-03 | -1,70252 |
| 3204833 | GBA2 | NM_020944 | 7,47E-04 | -1,70237 |
| 3307939 | ABLIM1 | NM_002313 | 1,33E-03 | -1,69947 |
| 3923257 | PDXK | NM_003681 | 2,87E-05 | -1,69844 |
| 3728509 | DYNLL2 | NM_080677 | 4,56E-06 | -1,69775 |
| 3738490 | GPS1 | NM_212492 | 8,26E-07 | -1,69558 |
| 2449693 | DENND1B | NM_001142795 | 9,99E-04 | -1,69487 |
| 2635812 | PLCXD2 | NM_153268 | 1,06E-03 | -1,69395 |
| 3835935 | CLPTM1 | NM_001294 | 1,14E-05 | -1,69374 |
| 3734355 | GPRC5C | NM_022036 | 6,63E-05 | -1,69369 |
| 2624110 | SPCS1 | NM_014041 | 9,29E-04 | -1,69339 |
| 3721548 | CNP | NM_033133 | 1,03E-04 | -1,69175 |
| 3751859 | TMIGD1 | NM_206832 | 3,82E-04 | -1,68808 |
| 3761395 | HOXB6 | NM_018952 | 8,17E-04 | -1,68603 |
| 3550077 | GLRX5 | NM_016417 | 6,47E-05 | -1,68506 |
| 2672230 | ALS2CL | NM_147129 | 1,83E-04 | -1,68411 |
| 3304624 | NT5C2 | NM_012229 | 7,66E-05 | -1,68307 |
| 3600283 | THSD4 | NM_024817 | 5,24E-04 | -1,68099 |
| 3009198 | RHBDD2 | NM_001040457 | 2,11E-04 | -1,67918 |
| 2450823 | TNNI1 | NM_003281 | 1,08E-05 | -1,67849 |
| 3584443 | SNRPN | NM_022807 | 1,20E-04 | -1,67774 |
| 2953139 | MOCS1 | NM_005943 | 3,24E-04 | -1,67748 |
| 2451493 | CYB5R1 | NM_016243 | 8,93E-05 | -1,67526 |
| 2449104 | B3GALT2 | NM_003783 | 7,21E-05 | -1,6747 |
| 3152558 | FAM84B | NM_174911 | 2,66E-04 | -1,67355 |
| 3319352 | TUB | NM_003320 | 3,64E-04 | -1,67334 |
| 2672857 | SMARCC1 | NM_003074 | 2,82E-04 | -1,67094 |
| 2846522 | IRX2 | NM_033267 | 3,08E-05 | -1,67081 |
| 3537164 | PELI2 | NM_021255 | 2,12E-04 | -1,67043 |
| 2784352 | FGF2 | NM_002006 | 6,23E-04 | -1,66809 |
| 2672712 | SCAP | NM_012235 | 1,38E-06 | -1,66767 |
| 2402493 | PAFAH2 | NM_000437 | 7,11E-05 | -1,66707 |
| 2513554 | CSRNP3 | NM_001172173 | 7,02E-07 | -1,66354 |
| 3575241 | KCNK10 | NM_021161 | 1,43E-05 | -1,6594 |
| 3766373 | FTSJ3 | NM_017647 | 2,87E-07 | -1,65906 |
| 2663244 | RAF1 | NM_002880 | 1,33E-05 | -1,65779 |
| 3311715 | UROS | NM_000375 | 2,98E-04 | -1,65776 |
| 2430994 | ZNF697 | NM_001080470 | 2,00E-05 | -1,65681 |
| 2358671 | C1orf56 | BC002469 | 1,08E-04 | -1,65516 |
| 2326327 | CNKSR1 | NM_006314 | 4,42E-05 | -1,65398 |
| 3756723 | KRTAP2-4 | NM_033184 | 3,53E-04 | -1,65071 |
| 3944620 | MPST | NR_024038 | 5,96E-04 | -1,64673 |
| 2799758 | IRX1 | NM_024337 | 6,24E-05 | -1,64653 |
| 2880051 | PPP2R2B | NM_181674 | 6,28E-06 | -1,64623 |
| 2958172 | BMP5 | NM_021073 | 1,66E-04 | -1,64616 |
| 2942578 | CCDC90A | NM_001031713 | 1,24E-05 | -1,64531 |
| 2875685 | FSTL4 | NM_015082 | 4,28E-05 | -1,64489 |
| 2559494 | C2orf7 | NM_032319 | 5,05E-04 | -1,64442 |
| 4014759 | NAP1L3 | NM_004538 | 4,89E-05 | -1,63979 |
| 3623771 | TRPM7 | NM_017672 | 3,61E-04 | -1,63906 |
| 3011317 | CROT | NM_001143935 | 5,14E-05 | -1,63466 |
| 2363372 | KLHDC9 | NM_152366 | 4,19E-07 | -1,63442 |
| 2651835 | GPR160 | NM_014373 | 1,34E-03 | -1,63317 |
| 3718185 | CCL11 | NM_002986 | 3,57E-04 | -1,62869 |
| 2621917 | WDR6 | NM_018031 | 7,70E-05 | -1,62868 |
| 2648535 | SGEF | NM_015595 | 1,36E-05 | -1,62787 |
| 3401099 | FKBP4 | NM_002014 | 1,54E-03 | -1,62758 |
| 2671032 | C3orf39 | NM_032806 | 1,22E-04 | -1,62757 |
| 2351121 | AHCYL1 | NM_006621 | 9,70E-07 | -1,62663 |
| 3625440 | PYGO1 | NM_015617 | 1,49E-03 | -1,62428 |
| 3174121 | MAMDC2 | NM_153267 | 1,32E-03 | -1,62414 |
| 3642875 | RAB11FIP3 | NM_014700 | 8,95E-04 | -1,62386 |
| 3725456 | ATP5G1 | NM_005175 | 2,10E-04 | -1,62217 |
| 3376490 | HRASLS5 | NM_054108 | 1,15E-03 | -1,62193 |
| 3915569 | CHODL | NM_024944 | 2,52E-05 | -1,62039 |
| 3112584 | SLC30A8 | NM_001172813 | 5,90E-04 | -1,62024 |
| 2585476 | SCN7A | NM_002976 | 3,39E-05 | -1,6188 |
| 2398789 | SDHB | NM_003000 | 1,23E-03 | -1,6178 |
| 3543935 | COQ6 | NM_182476 | 8,28E-04 | -1,61635 |
| 2612813 | PLCL2 | NM_015184 | 7,67E-04 | -1,61534 |
| 2758298 | LRPAP1 | NM_002337 | 2,42E-04 | -1,61494 |
| 2401384 | ASAP3 | NM_017707 | 2,94E-04 | -1,61379 |
| 2772968 | COX18 | NM_173827 | 6,28E-04 | -1,61353 |
| 2420521 | SSX2IP | NM_001166417 | 1,51E-03 | -1,6133 |
| 3505449 | MIPEP | NM_005932 | 4,43E-04 | -1,61313 |
| 3441280 | AKAP3 | NM_006422 | 8,03E-06 | -1,61194 |
| 2653673 | KCNMB2 | NM_005832 | 1,32E-04 | -1,61176 |
| 2967818 | PDSS2 | NM_020381 | 1,24E-03 | -1,6098 |
| 3333647 | TAF6L | NM_006473 | 2,21E-05 | -1,60961 |
| 3529725 | REC8 | NM_001048205 | 1,32E-05 | -1,60905 |
| 3815143 | C19orf21 | BC052236 | 1,37E-04 | -1,60887 |
| 3860189 | C19orf46 | NM_001039876 | 7,39E-05 | -1,60745 |
| 3824197 | MRPL34 | NM_023937 | 1,33E-03 | -1,60648 |
| 4001785 | MAP3K15 | NM_001001671 | 3,59E-04 | -1,60173 |
| 3185522 | SLC31A1 | NM_001859 | 1,27E-03 | -1,59938 |
| 2326912 | WDTC1 | NM_015023 | 2,92E-06 | -1,59845 |
| 3671727 | ATP2C2 | NM_014861 | 1,06E-05 | -1,59479 |
| 2505957 | PLEKHB2 | NM_017958 | 1,10E-04 | -1,59297 |
| 3382410 | MAP6 | NM_207577 | 2,57E-06 | -1,59215 |
| 3334783 | SNX15 | NM_013306 | 1,70E-04 | -1,58652 |
| 3745504 | SCO1 | NM_004589 | 5,13E-05 | -1,58467 |
| 3221598 | WDR31 | NM_001012361 | 8,33E-05 | -1,58339 |
| 3985305 | GPRASP2 | NM_001004051 | 1,48E-03 | -1,5806 |
| 2397695 | CASP9 | NM_001229 | 1,46E-03 | -1,57944 |
| 3252170 | ADK | NM_006721 | 1,20E-03 | -1,5791 |
| 3524999 | LIG4 | NM_002312 | 1,58E-04 | -1,57892 |
| 2351004 | GSTM5 | NM_000851 | 7,94E-04 | -1,57804 |
| 3893610 | ZGPAT | NM_032527 | 3,53E-05 | -1,57644 |
| 2985368 | FRMD1 | NM_024919 | 8,77E-05 | -1,57495 |
| 3945014 | GCAT | NM_001171690 | 1,17E-03 | -1,57373 |
| 3730731 | DCAF7 | NM_005828 | 2,17E-04 | -1,57217 |
| 3774593 | DUS1L | NM_022156 | 5,22E-05 | -1,56924 |
| 3431220 | MVK | NM_000431 | 5,50E-04 | -1,56882 |
| 3082759 | DLGAP2 | NM_004745 | 4,07E-05 | -1,5676 |
| 3303530 | NDUFB8 | NM_005004 | 1,46E-03 | -1,5674 |
| 3261165 | BTRC | NM_033637 | 1,52E-04 | -1,56457 |
| 2669488 | PLCD1 | NR_024071 | 1,05E-05 | -1,56298 |
| 3087990 | NAT2 | NM_000015 | 2,37E-04 | -1,56268 |
| 2741206 | MYOZ2 | NM_016599 | 7,43E-04 | -1,56211 |
| 3946762 | ZC3H7B | NM_017590 | 7,07E-04 | -1,56054 |
| 3512294 | TSC22D1 | NM_183422 | 9,56E-04 | -1,55922 |
| 2359036 | SNX27 | NM_030918 | 9,43E-05 | -1,55862 |
| 2396537 | MTOR | NM_004958 | 2,34E-04 | -1,55741 |
| 3726537 | EPN3 | NM_017957 | 4,36E-05 | -1,5573 |
| 3824124 | OCEL1 | NM_024578 | 6,70E-04 | -1,55729 |
| 2904329 | ANKS1A | NM_015245 | 1,76E-06 | -1,55725 |
| 2480168 | PRKCE | NM_005400 | 2,13E-04 | -1,55484 |
| 3874198 | OXT | NM_000915 | 9,57E-04 | -1,54959 |
| 3660075 | NKD1 | NM_033119 | 2,82E-07 | -1,54758 |
| 3957224 | TBC1D10A | NM_031937 | 2,45E-04 | -1,5466 |
| 3675935 | CLCN7 | NM_001287 | 5,91E-04 | -1,54383 |
| 3711899 | TTC19 | NM_017775 | 7,23E-04 | -1,54357 |
| 2780296 | TACR3 | NM_001059 | 1,10E-03 | -1,5428 |
| 2662087 | SRGAP3 | NM_014850 | 1,02E-03 | -1,54258 |
| 3435241 | LRRC43 | NM_001098519 | 3,31E-07 | -1,54109 |
| 3557268 | PPP1R3E | NR_026862 | 1,28E-03 | -1,53875 |
| 2622121 | DAG1 | NM_001177634 | 1,80E-04 | -1,53674 |
| 2967151 | HACE1 | NM_020771 | 8,68E-04 | -1,53634 |
| 3750939 | SDF2 | NM_006923 | 3,79E-04 | -1,53556 |
| 3259978 | PI4K2A | NM_018425 | 3,21E-05 | -1,53494 |
| 3239380 | THNSL1 | NM_024838 | 1,33E-03 | -1,53321 |
| 2674673 | IP6K1 | NM_153273 | 4,60E-04 | -1,53132 |
| 3750430 | C17orf108 | NM_001076680 | 2,20E-04 | -1,52968 |
| 3305198 | C10orf79 | NM_025145 | 4,00E-05 | -1,52892 |
| 2489806 | MRPL19 | NM_014763 | 9,57E-06 | -1,52883 |
| 2918037 | KLHL32 | NM_052904 | 7,44E-05 | -1,52822 |
| 3282268 | ACBD5 | NM_145698 | 2,43E-04 | -1,52821 |
| 3221800 | AMBP | NM_001633 | 8,13E-04 | -1,52625 |
| 3188478 | CRB2 | NM_173689 | 1,70E-06 | -1,52346 |
| 3119200 | PSCA | NM_005672 | 1,58E-04 | -1,51878 |
| 3695699 | ATP6V0D1 | NM_004691 | 9,36E-04 | -1,51768 |
| 3460127 | GNS | NM_002076 | 7,40E-04 | -1,51622 |
| 3376714 | MACROD1 | NM_014067 | 5,76E-04 | -1,51379 |
| 3831006 | LRFN3 | NM_024509 | 1,34E-03 | -1,51022 |
| 3380365 | SHANK2 | NM_012309 | 1,17E-03 | -1,50969 |
| 3825383 | UPF1 | NM_002911 | 9,75E-04 | -1,50851 |
| 2931763 | ESR1 | NM_001122742 | 7,06E-05 | -1,50783 |
| 4010646 | ASB12 | NM_130388 | 8,67E-04 | -1,50717 |
| 4011189 | OPHN1 | NM_002547 | 1,06E-03 | -1,50695 |
| 2623859 | NISCH | NM_007184 | 5,21E-04 | -1,50662 |
| 3721795 | NAGLU | NM_000263 | 3,40E-04 | -1,50615 |
| 3665949 | PSKH1 | NM_006742 | 9,84E-04 | -1,50557 |
| 3303300 | CHUK | NM_001278 | 9,43E-05 | -1,505 |
| 3934439 | DNMT3L | NM_013369 | 6,36E-05 | -1,50481 |
| 2891768 | FOXC1 | NM_001453 | 1,01E-03 | -1,5042 |
| 3806689 | HDHD2 | NM_032124 | 1,35E-03 | -1,50239 |
| 3034987 | ADAP1 | NM_006869 | 5,39E-04 | -1,50121 |
| 2693357 | SLC41A3 | NM_017836 | 1,13E-03 | -1,50001 |
| 2981676 | SERAC1 | NM_032861 | 8,03E-04 | -1,49686 |
| 2540007 | CYS1 | NM_001037160 | 1,71E-06 | -1,49588 |
| 3050170 | ZPBP | NM_007009 | 3,33E-05 | -1,49456 |
| 3989259 | GLUD2 | NM_012084 | 1,36E-03 | -1,49364 |
| 3623472 | C15orf33 | NM_152647 | 5,01E-05 | -1,49343 |
| 3803500 | C18orf34 | NM_001105528 | 9,10E-04 | -1,49168 |
| 2558150 | AAK1 | NM_014911 | 1,34E-05 | -1,49085 |
| 3205019 | OR2S2 | NM_019897 | 4,59E-04 | -1,48913 |
| 3302495 | AVPI1 | NM_021732 | 6,33E-05 | -1,48819 |
| 3202528 | LINGO2 | NM_152570 | 7,27E-05 | -1,48773 |
| 3759540 | DCAKD | NM_024819 | 6,34E-04 | -1,48701 |
| 3410322 | C12orf72 | NM_173802 | 6,01E-05 | -1,48518 |
| 3046739 | AMPH | NM_001635 | 7,70E-04 | -1,48513 |
| 2675315 | CACNA2D2 | NM_001005505 | 1,24E-03 | -1,48235 |
| 2776126 | OK | AB064670 | 1,36E-03 | -1,48135 |
| 3589756 | PAK6 | NM_020168 | 1,69E-05 | -1,47677 |
| 2331158 | AKIRIN1 | NM_024595 | 1,96E-04 | -1,47511 |
| 2881300 | CAMK2A | NM_015981 | 8,96E-05 | -1,47473 |
| 2756673 | GAK | NM_005255 | 1,14E-03 | -1,47318 |
| 3709590 | RANGRF | NM_001177802 | 2,15E-04 | -1,47032 |
| 3874313 | ATRN | NM_139321 | 5,07E-04 | -1,4699 |
| 2621949 | NDUFAF3 | NM_199069 | 8,62E-04 | -1,46853 |
| 3832865 | NCCRP1 | NM_001001414 | 3,38E-04 | -1,46823 |
| 3895274 | ProSAPiP1 | NM_014731 | 8,61E-06 | -1,4668 |
| 2525989 | CPS1 | NM_001122633 | 3,11E-04 | -1,46614 |
| 2675304 | TMEM115 | NM_007024 | 9,98E-04 | -1,46342 |
| 3571810 | ABCD4 | NM_005050 | 5,76E-05 | -1,462 |
| 3167511 | GALT | NM_000155 | 5,74E-04 | -1,4608 |
| 3358492 | POLR2L | NM_021128 | 3,23E-04 | -1,45881 |
| 3271220 | CTAGEP | BC036527 | 2,18E-04 | -1,45762 |
| 2448710 | FAM5C | NM_199051 | 5,79E-06 | -1,45494 |
| 2566586 | TSGA10 | NM_182911 | 1,53E-03 | -1,45487 |
| 3645901 | NAT15 | NM_024845 | 9,21E-04 | -1,45476 |
| 3259836 | ZDHHC16 | NM_198046 | 1,33E-04 | -1,4547 |
| 2971724 | FAM184A | NM_024581 | 1,48E-05 | -1,45411 |
| 3023279 | TPI1P2 | NR_002187 | 2,69E-04 | -1,45392 |
| 3722700 | NAGS | NM_153006 | 1,44E-03 | -1,45328 |
| 3226005 | PTRH1 | NM_001002913 | 5,94E-04 | -1,45323 |
| 3935243 | LSS | NM_001145437 | 5,25E-04 | -1,45051 |
| 2727976 | CEP135 | NM_025009 | 1,44E-03 | -1,45028 |
| 3847252 | SAFB2 | NM_014649 | 9,67E-05 | -1,44952 |
| 3721851 | COASY | NM_025233 | 2,36E-04 | -1,44904 |
| 3304475 | ARL3 | NM_004311 | 3,82E-04 | -1,44835 |
| 3985320 | BHLHB9 | NM_001142524 | 1,54E-05 | -1,44784 |
| 2621333 | PTPN23 | NM_015466 | 2,73E-04 | -1,44673 |
| 4000538 | FIGF | NM_004469 | 1,74E-04 | -1,44626 |
| 3725035 | NFE2L1 | NM_003204 | 3,88E-04 | -1,4446 |
| 3302467 | MORN4 | NM_178832 | 4,97E-04 | -1,43989 |
| 2793137 | SH3RF1 | NM_020870 | 4,98E-04 | -1,43851 |
| 2779095 | ADH5 | NM_000671 | 9,94E-04 | -1,43676 |
| 3787675 | KIAA0427 | NM_001142397 | 7,86E-05 | -1,43676 |
| 4051226 | SEMA4D | NM_001142287 | 1,58E-04 | -1,43665 |
| 2952497 | BTBD9 | NM_052893 | 1,14E-04 | -1,43576 |
| 3259959 | C10orf62 | NM_001009997 | 1,43E-03 | -1,43532 |
| 3374698 | OSBP | NM_002556 | 1,08E-03 | -1,43167 |
| 2367963 | RABGAP1L | NM_014857 | 1,52E-03 | -1,43056 |
| 3245682 | MAPK8 | NM_002750 | 3,45E-04 | -1,42875 |
| 3459801 | DPY19L2 | NM_173812 | 6,93E-05 | -1,4269 |
| 3695107 | TK2 | NM_004614 | 4,63E-04 | -1,42452 |
| 3511817 | ENOX1 | NM_017993 | 1,18E-04 | -1,42448 |
| 3278234 | SEPHS1 | NM_012247 | 1,28E-03 | -1,4242 |
| 3266583 | CASC2 | NR_026939 | 2,46E-04 | -1,42198 |
| 3224556 | C9orf45 | NR_026677 | 1,21E-04 | -1,42161 |
| 2452311 | TMEM81 | NM_203376 | 6,27E-04 | -1,4212 |
| 2391647 | SSU72 | NM_014188 | 3,73E-04 | -1,41977 |
| 3371964 | PACSIN3 | NM_016223 | 3,31E-04 | -1,41925 |
| 3294361 | TTC18 | NM_145170 | 4,59E-05 | -1,41754 |
| 3623751 | USP50 | NM_203494 | 9,57E-05 | -1,41627 |
| 3359432 | CDKN1C | NM_000076 | 1,34E-04 | -1,4153 |
| 4026722 | IDH3G | NM_174869 | 5,81E-05 | -1,41409 |
| 3157147 | LYNX1 | NM_177457 | 1,20E-04 | -1,41254 |
| 3396084 | VSIG2 | NM_014312 | 6,91E-04 | -1,41219 |
| 3361245 | ZNF214 | NM_013249 | 6,47E-05 | -1,4117 |
| 3755820 | PGAP3 | NM_033419 | 2,09E-04 | -1,41142 |
| 3718791 | TAF15 | NM_139215 | 2,81E-05 | -1,41077 |
| 2910477 | FBXO9 | NM_033480 | 4,10E-04 | -1,41031 |
| 2941757 | ERVFRDE1 | NM_207582 | 6,54E-04 | -1,40988 |
| 4026263 | CETN2 | NM_004344 | 2,18E-04 | -1,40735 |
| 2414558 | DAB1 | NM_021080 | 8,18E-06 | -1,40732 |
| 2602110 | COL4A4 | NM_000092 | 8,17E-04 | -1,40728 |
| 3511135 | KBTBD6 | NM_152903 | 3,69E-04 | -1,40684 |
| 3757664 | RAB5C | NM_201434 | 7,34E-04 | -1,40615 |
| 2673730 | PRKAR2A | NM_004157 | 1,76E-04 | -1,4045 |
| 2408028 | NT5C1A | NM_032526 | 1,09E-04 | -1,40353 |
| 2469825 | GREB1 | NM_014668 | 9,23E-04 | -1,40284 |
| 3138929 | LRRC67 | NM_001013626 | 4,51E-04 | -1,40257 |
| 2824581 | KCNN2 | NM_021614 | 8,67E-05 | -1,4011 |
