# Supplementary material for: Genome-Wide Analysis of Differentially Expressed Genes and Splicing Isoforms in Clear Cell Renal Cell Carcinoma
Source: PLoS One. 2013 Oct 23;8(10):e78452. doi: 10.1371/journal.pone.0078452 (PMC3806822; doi:10.1371/journal.pone.0078452)
Supplement: Table S6 — Gene ontology enrichment analysis of genes up-regulated in ccRCC performed by DAVID. Annotations were considered significantly over-represented when the p-value of the Fisher's exact test as used by DAVID (EASE Score) was < 0.05 and gene counts belonging to an annotation term was equal or greater than 2. (DOCX) [file pone.0078452.s006.docx]

| \| [Category](http://david.abcc.ncifcrf.gov/chartReport.jsp?visited=yes&d-16544-s=1&cbBenjamini=true&rowids=&count=2&d-16544-o=2&cbFC=true&d-16544-p=1&annot=25&ease=0.1&numRecords=1000&heading=) \| [Term](http://david.abcc.ncifcrf.gov/chartReport.jsp?visited=yes&d-16544-s=2&cbBenjamini=true&rowids=&count=2&d-16544-o=2&cbFC=true&d-16544-p=1&annot=25&ease=0.1&numRecords=1000&heading=) \| [Count](http://david.abcc.ncifcrf.gov/chartReport.jsp?visited=yes&d-16544-s=5&cbBenjamini=true&rowids=&count=2&d-16544-o=1&cbFC=true&d-16544-p=1&annot=25&ease=0.1&numRecords=1000&heading=) \| [%](http://david.abcc.ncifcrf.gov/chartReport.jsp?visited=yes&d-16544-s=6&cbBenjamini=true&rowids=&count=2&d-16544-o=1&cbFC=true&d-16544-p=1&annot=25&ease=0.1&numRecords=1000&heading=) \| [P-Value](http://david.abcc.ncifcrf.gov/chartReport.jsp?visited=yes&d-16544-s=7&cbBenjamini=true&rowids=&count=2&d-16544-o=1&cbFC=true&d-16544-p=1&annot=25&ease=0.1&numRecords=1000&heading=) \| [Fold Enrichment](http://david.abcc.ncifcrf.gov/chartReport.jsp?visited=yes&d-16544-s=8&cbBenjamini=true&rowids=&count=2&d-16544-o=1&cbFC=true&d-16544-p=1&annot=25&ease=0.1&numRecords=1000&heading=) \| [Benjamini](http://david.abcc.ncifcrf.gov/chartReport.jsp?visited=yes&d-16544-s=9&cbBenjamini=true&rowids=&count=2&d-16544-o=1&cbFC=true&d-16544-p=1&annot=25&ease=0.1&numRecords=1000&heading=) \| \| --- \| --- \| --- \| --- \| --- \| --- \| --- \| \| GOTERM_BP_FAT \| [immune response](http://www.ebi.ac.uk/QuickGO/GTerm?id=GO:0006955) \| 149 \| 11.4 \| 2.7E-32 \| 2.8 \| 9.7E-29 \| \| GOTERM_BP_FAT \| [cell activation](http://www.ebi.ac.uk/QuickGO/GTerm?id=GO:0001775) \| 80 \| 6.1 \| 1.2E-24 \| 3.6 \| 2.1E-21 \| \| GOTERM_BP_FAT \| [leukocyte activation](http://www.ebi.ac.uk/QuickGO/GTerm?id=GO:0045321) \| 72 \| 5.5 \| 4.7E-24 \| 3.9 \| 5.6E-21 \| \| GOTERM_BP_FAT \| [lymphocyte activation](http://www.ebi.ac.uk/QuickGO/GTerm?id=GO:0046649) \| 58 \| 4.4 \| 5.3E-19 \| 3.8 \| 4.7E-16 \| \| GOTERM_BP_FAT \| [positive regulation of immune system process](http://www.ebi.ac.uk/QuickGO/GTerm?id=GO:0002684) \| 64 \| 4.9 \| 6.0E-19 \| 3.5 \| 4.3E-16 \| \| GOTERM_BP_FAT \| [T cell activation](http://www.ebi.ac.uk/QuickGO/GTerm?id=GO:0042110) \| 45 \| 3.4 \| 1.4E-18 \| 4.6 \| 8.2E-16 \| \| GOTERM_BP_FAT \| [defense response](http://www.ebi.ac.uk/QuickGO/GTerm?id=GO:0006952) \| 111 \| 8.5 \| 1.1E-17 \| 2.3 \| 5.5E-15 \| \| GOTERM_BP_FAT \| [intracellular signaling cascade](http://www.ebi.ac.uk/QuickGO/GTerm?id=GO:0007242) \| 168 \| 12.9 \| 3.1E-13 \| 1.7 \| 1.4E-10 \| \| GOTERM_BP_FAT \| [regulation of lymphocyte activation](http://www.ebi.ac.uk/QuickGO/GTerm?id=GO:0051249) \| 41 \| 3.1 \| 9.5E-13 \| 3.6 \| 3.8E-10 \| \| GOTERM_BP_FAT \| [regulation of cell activation](http://www.ebi.ac.uk/QuickGO/GTerm?id=GO:0050865) \| 45 \| 3.4 \| 1.1E-12 \| 3.3 \| 3.8E-10 \| \| GOTERM_BP_FAT \| [regulation of leukocyte activation](http://www.ebi.ac.uk/QuickGO/GTerm?id=GO:0002694) \| 43 \| 3.3 \| 2.8E-12 \| 3.4 \| 9.2E-10 \| \| GOTERM_BP_FAT \| [response to wounding](http://www.ebi.ac.uk/QuickGO/GTerm?id=GO:0009611) \| 88 \| 6.7 \| 6.9E-12 \| 2.2 \| 2.1E-9 \| \| GOTERM_BP_FAT \| [cell proliferation](http://www.ebi.ac.uk/QuickGO/GTerm?id=GO:0008283) \| 76 \| 5.8 \| 2.2E-11 \| 2.3 \| 6.0E-9 \| \| GOTERM_BP_FAT \| [regulation of T cell activation](http://www.ebi.ac.uk/QuickGO/GTerm?id=GO:0050863) \| 34 \| 2.6 \| 2.6E-11 \| 3.8 \| 6.8E-9 \| \| GOTERM_BP_FAT \| [hemopoiesis](http://www.ebi.ac.uk/QuickGO/GTerm?id=GO:0030097) \| 51 \| 3.9 \| 3.0E-11 \| 2.8 \| 7.3E-9 \| \| GOTERM_BP_FAT \| [hemopoietic or lymphoid organ development](http://www.ebi.ac.uk/QuickGO/GTerm?id=GO:0048534) \| 53 \| 4.1 \| 1.2E-10 \| 2.6 \| 2.6E-8 \| \| GOTERM_BP_FAT \| [inflammatory response](http://www.ebi.ac.uk/QuickGO/GTerm?id=GO:0006954) \| 61 \| 4.7 \| 1.3E-10 \| 2.4 \| 2.8E-8 \| \| GOTERM_BP_FAT \| [positive regulation of cell activation](http://www.ebi.ac.uk/QuickGO/GTerm?id=GO:0050867) \| 32 \| 2.5 \| 1.4E-10 \| 3.7 \| 2.8E-8 \| \| GOTERM_BP_FAT \| [positive regulation of immune response](http://www.ebi.ac.uk/QuickGO/GTerm?id=GO:0050778) \| 37 \| 2.8 \| 1.8E-10 \| 3.3 \| 3.4E-8 \| \| GOTERM_BP_FAT \| [positive regulation of response to stimulus](http://www.ebi.ac.uk/QuickGO/GTerm?id=GO:0048584) \| 49 \| 3.8 \| 3.3E-10 \| 2.7 \| 6.0E-8 \| \| GOTERM_BP_FAT \| [lymphocyte differentiation](http://www.ebi.ac.uk/QuickGO/GTerm?id=GO:0030098) \| 30 \| 2.3 \| 4.4E-10 \| 3.8 \| 7.6E-8 \| \| GOTERM_BP_FAT \| [positive regulation of lymphocyte activation](http://www.ebi.ac.uk/QuickGO/GTerm?id=GO:0051251) \| 29 \| 2.2 \| 4.7E-10 \| 3.9 \| 7.7E-8 \| \| GOTERM_BP_FAT \| [regulation of apoptosis](http://www.ebi.ac.uk/QuickGO/GTerm?id=GO:0042981) \| 112 \| 8.6 \| 5.5E-10 \| 1.8 \| 8.6E-8 \| \| GOTERM_BP_FAT \| [positive regulation of leukocyte activation](http://www.ebi.ac.uk/QuickGO/GTerm?id=GO:0002696) \| 30 \| 2.3 \| 9.3E-10 \| 3.7 \| 1.4E-7 \| \| GOTERM_BP_FAT \| [regulation of programmed cell death](http://www.ebi.ac.uk/QuickGO/GTerm?id=GO:0043067) \| 112 \| 8.6 \| 9.9E-10 \| 1.8 \| 1.4E-7 \| \| GOTERM_BP_FAT \| [immune system development](http://www.ebi.ac.uk/QuickGO/GTerm?id=GO:0002520) \| 53 \| 4.1 \| 1.1E-9 \| 2.5 \| 1.5E-7 \| \| GOTERM_BP_FAT \| [regulation of cell death](http://www.ebi.ac.uk/QuickGO/GTerm?id=GO:0010941) \| 112 \| 8.6 \| 1.2E-9 \| 1.8 \| 1.6E-7 \| \| GOTERM_BP_FAT \| [adaptive immune response based on somatic recombination of immune receptors built from immunoglobulin superfamily domains](http://www.ebi.ac.uk/QuickGO/GTerm?id=GO:0002460) \| 25 \| 1.9 \| 1.5E-9 \| 4.2 \| 1.9E-7 \| \| GOTERM_BP_FAT \| [adaptive immune response](http://www.ebi.ac.uk/QuickGO/GTerm?id=GO:0002250) \| 25 \| 1.9 \| 1.5E-9 \| 4.2 \| 1.9E-7 \| \| GOTERM_BP_FAT \| [regulation of cytokine production](http://www.ebi.ac.uk/QuickGO/GTerm?id=GO:0001817) \| 40 \| 3.1 \| 2.8E-9 \| 2.9 \| 3.4E-7 \| \| GOTERM_BP_FAT \| [leukocyte differentiation](http://www.ebi.ac.uk/QuickGO/GTerm?id=GO:0002521) \| 33 \| 2.5 \| 2.9E-9 \| 3.3 \| 3.4E-7 \| \| GOTERM_BP_FAT \| [regulation of cell proliferation](http://www.ebi.ac.uk/QuickGO/GTerm?id=GO:0042127) \| 107 \| 8.2 \| 5.6E-9 \| 1.8 \| 6.5E-7 \| \| GOTERM_BP_FAT \| [T cell differentiation](http://www.ebi.ac.uk/QuickGO/GTerm?id=GO:0030217) \| 22 \| 1.7 \| 7.6E-9 \| 4.4 \| 8.5E-7 \| \| GOTERM_BP_FAT \| [innate immune response](http://www.ebi.ac.uk/QuickGO/GTerm?id=GO:0045087) \| 33 \| 2.5 \| 1.1E-8 \| 3.1 \| 1.2E-6 \| \| GOTERM_BP_FAT \| [cell activation during immune response](http://www.ebi.ac.uk/QuickGO/GTerm?id=GO:0002263) \| 16 \| 1.2 \| 2.1E-8 \| 5.8 \| 2.2E-6 \| \| GOTERM_BP_FAT \| [leukocyte activation during immune response](http://www.ebi.ac.uk/QuickGO/GTerm?id=GO:0002366) \| 16 \| 1.2 \| 2.1E-8 \| 5.8 \| 2.2E-6 \| \| GOTERM_BP_FAT \| [positive regulation of T cell activation](http://www.ebi.ac.uk/QuickGO/GTerm?id=GO:0050870) \| 23 \| 1.8 \| 3.2E-8 \| 3.9 \| 3.3E-6 \| \| GOTERM_BP_FAT \| [antigen processing and presentation](http://www.ebi.ac.uk/QuickGO/GTerm?id=GO:0019882) \| 24 \| 1.8 \| 3.9E-8 \| 3.8 \| 3.9E-6 \| \| GOTERM_BP_FAT \| [immune effector process](http://www.ebi.ac.uk/QuickGO/GTerm?id=GO:0002252) \| 31 \| 2.4 \| 7.5E-8 \| 3.0 \| 7.3E-6 \| \| GOTERM_BP_FAT \| [leukocyte mediated immunity](http://www.ebi.ac.uk/QuickGO/GTerm?id=GO:0002443) \| 24 \| 1.8 \| 8.0E-8 \| 3.6 \| 7.6E-6 \| \| GOTERM_BP_FAT \| [positive regulation of apoptosis](http://www.ebi.ac.uk/QuickGO/GTerm?id=GO:0043065) \| 66 \| 5.1 \| 9.2E-8 \| 2.0 \| 8.4E-6 \| \| GOTERM_BP_FAT \| [programmed cell death](http://www.ebi.ac.uk/QuickGO/GTerm?id=GO:0012501) \| 85 \| 6.5 \| 9.6E-8 \| 1.8 \| 8.6E-6 \| \| GOTERM_BP_FAT \| [immune response-regulating signal transduction](http://www.ebi.ac.uk/QuickGO/GTerm?id=GO:0002764) \| 19 \| 1.5 \| 9.7E-8 \| 4.4 \| 8.5E-6 \| \| GOTERM_BP_FAT \| [positive regulation of programmed cell death](http://www.ebi.ac.uk/QuickGO/GTerm?id=GO:0043068) \| 66 \| 5.1 \| 1.2E-7 \| 2.0 \| 1.0E-5 \| \| GOTERM_BP_FAT \| [negative regulation of leukocyte activation](http://www.ebi.ac.uk/QuickGO/GTerm?id=GO:0002695) \| 19 \| 1.5 \| 1.3E-7 \| 4.3 \| 1.1E-5 \| \| GOTERM_BP_FAT \| [positive regulation of cell death](http://www.ebi.ac.uk/QuickGO/GTerm?id=GO:0010942) \| 66 \| 5.1 \| 1.4E-7 \| 2.0 \| 1.2E-5 \| \| GOTERM_BP_FAT \| [negative regulation of immune system process](http://www.ebi.ac.uk/QuickGO/GTerm?id=GO:0002683) \| 23 \| 1.8 \| 1.8E-7 \| 3.6 \| 1.5E-5 \| \| GOTERM_BP_FAT \| [apoptosis](http://www.ebi.ac.uk/QuickGO/GTerm?id=GO:0006915) \| 83 \| 6.4 \| 2.1E-7 \| 1.8 \| 1.6E-5 \| \| GOTERM_BP_FAT \| [regulation of mononuclear cell proliferation](http://www.ebi.ac.uk/QuickGO/GTerm?id=GO:0032944) \| 23 \| 1.8 \| 2.3E-7 \| 3.6 \| 1.7E-5 \| \| GOTERM_BP_FAT \| [regulation of leukocyte proliferation](http://www.ebi.ac.uk/QuickGO/GTerm?id=GO:0070663) \| 23 \| 1.8 \| 2.3E-7 \| 3.6 \| 1.7E-5 \| \| GOTERM_BP_FAT \| [macromolecular complex assembly](http://www.ebi.ac.uk/QuickGO/GTerm?id=GO:0065003) \| 89 \| 6.8 \| 2.7E-7 \| 1.7 \| 2.0E-5 \| \| GOTERM_BP_FAT \| [negative regulation of lymphocyte activation](http://www.ebi.ac.uk/QuickGO/GTerm?id=GO:0051250) \| 18 \| 1.4 \| 3.1E-7 \| 4.3 \| 2.3E-5 \| \| GOTERM_BP_FAT \| [cell death](http://www.ebi.ac.uk/QuickGO/GTerm?id=GO:0008219) \| 94 \| 7.2 \| 3.4E-7 \| 1.7 \| 2.5E-5 \| \| GOTERM_BP_FAT \| [DNA replication](http://www.ebi.ac.uk/QuickGO/GTerm?id=GO:0006260) \| 37 \| 2.8 \| 3.5E-7 \| 2.5 \| 2.4E-5 \| \| GOTERM_BP_FAT \| [antigen processing and presentation of peptide antigen](http://www.ebi.ac.uk/QuickGO/GTerm?id=GO:0048002) \| 13 \| 1.0 \| 3.8E-7 \| 6.0 \| 2.6E-5 \| \| GOTERM_BP_FAT \| [regulation of phosphorylation](http://www.ebi.ac.uk/QuickGO/GTerm?id=GO:0042325) \| 68 \| 5.2 \| 3.9E-7 \| 1.9 \| 2.7E-5 \| \| GOTERM_BP_FAT \| [negative regulation of cell activation](http://www.ebi.ac.uk/QuickGO/GTerm?id=GO:0050866) \| 19 \| 1.5 \| 4.2E-7 \| 4.0 \| 2.8E-5 \| \| GOTERM_BP_FAT \| [regulation of adaptive immune response based on somatic recombination of immune receptors built from immunoglobulin superfamily domains](http://www.ebi.ac.uk/QuickGO/GTerm?id=GO:0002822) \| 18 \| 1.4 \| 4.2E-7 \| 4.3 \| 2.7E-5 \| \| GOTERM_BP_FAT \| [death](http://www.ebi.ac.uk/QuickGO/GTerm?id=GO:0016265) \| 94 \| 7.2 \| 4.7E-7 \| 1.7 \| 3.0E-5 \| \| GOTERM_BP_FAT \| [regulation of adaptive immune response](http://www.ebi.ac.uk/QuickGO/GTerm?id=GO:0002819) \| 18 \| 1.4 \| 5.6E-7 \| 4.2 \| 3.5E-5 \| \| GOTERM_BP_FAT \| [macromolecular complex subunit organization](http://www.ebi.ac.uk/QuickGO/GTerm?id=GO:0043933) \| 92 \| 7.0 \| 6.8E-7 \| 1.7 \| 4.2E-5 \| \| GOTERM_BP_FAT \| [regulation of lymphocyte proliferation](http://www.ebi.ac.uk/QuickGO/GTerm?id=GO:0050670) \| 22 \| 1.7 \| 8.0E-7 \| 3.4 \| 4.9E-5 \| \| GOTERM_BP_FAT \| [regulation of phosphate metabolic process](http://www.ebi.ac.uk/QuickGO/GTerm?id=GO:0019220) \| 69 \| 5.3 \| 8.2E-7 \| 1.8 \| 4.9E-5 \| \| GOTERM_BP_FAT \| [regulation of phosphorus metabolic process](http://www.ebi.ac.uk/QuickGO/GTerm?id=GO:0051174) \| 69 \| 5.3 \| 8.2E-7 \| 1.8 \| 4.9E-5 \| \| GOTERM_BP_FAT \| [lymphocyte mediated immunity](http://www.ebi.ac.uk/QuickGO/GTerm?id=GO:0002449) \| 20 \| 1.5 \| 8.4E-7 \| 3.7 \| 4.9E-5 \| \| GOTERM_BP_FAT \| [myeloid leukocyte activation](http://www.ebi.ac.uk/QuickGO/GTerm?id=GO:0002274) \| 16 \| 1.2 \| 9.3E-7 \| 4.5 \| 5.3E-5 \| \| GOTERM_BP_FAT \| [immune response-activating signal transduction](http://www.ebi.ac.uk/QuickGO/GTerm?id=GO:0002757) \| 17 \| 1.3 \| 9.8E-7 \| 4.2 \| 5.5E-5 \| \| GOTERM_BP_FAT \| [induction of apoptosis](http://www.ebi.ac.uk/QuickGO/GTerm?id=GO:0006917) \| 51 \| 3.9 \| 1.1E-6 \| 2.1 \| 5.9E-5 \| \| GOTERM_BP_FAT \| [positive regulation of cell communication](http://www.ebi.ac.uk/QuickGO/GTerm?id=GO:0010647) \| 52 \| 4.0 \| 1.1E-6 \| 2.1 \| 5.9E-5 \| \| GOTERM_BP_FAT \| [induction of programmed cell death](http://www.ebi.ac.uk/QuickGO/GTerm?id=GO:0012502) \| 51 \| 3.9 \| 1.2E-6 \| 2.1 \| 6.3E-5 \| \| GOTERM_BP_FAT \| [positive regulation of signal transduction](http://www.ebi.ac.uk/QuickGO/GTerm?id=GO:0009967) \| 48 \| 3.7 \| 1.3E-6 \| 2.1 \| 6.8E-5 \| \| GOTERM_BP_FAT \| [regulation of alpha-beta T cell activation](http://www.ebi.ac.uk/QuickGO/GTerm?id=GO:0046634) \| 14 \| 1.1 \| 1.3E-6 \| 5.1 \| 7.0E-5 \| \| GOTERM_BP_FAT \| [protein kinase cascade](http://www.ebi.ac.uk/QuickGO/GTerm?id=GO:0007243) \| 56 \| 4.3 \| 1.6E-6 \| 2.0 \| 8.0E-5 \| \| GOTERM_BP_FAT \| [negative regulation of T cell activation](http://www.ebi.ac.uk/QuickGO/GTerm?id=GO:0050868) \| 15 \| 1.1 \| 1.6E-6 \| 4.6 \| 8.1E-5 \| \| GOTERM_BP_FAT \| [immune response-regulating cell surface receptor signaling pathway](http://www.ebi.ac.uk/QuickGO/GTerm?id=GO:0002768) \| 15 \| 1.1 \| 1.6E-6 \| 4.6 \| 8.1E-5 \| \| GOTERM_BP_FAT \| [phagocytosis](http://www.ebi.ac.uk/QuickGO/GTerm?id=GO:0006909) \| 16 \| 1.2 \| 1.7E-6 \| 4.3 \| 8.6E-5 \| \| GOTERM_BP_FAT \| [activation of immune response](http://www.ebi.ac.uk/QuickGO/GTerm?id=GO:0002253) \| 23 \| 1.8 \| 1.8E-6 \| 3.2 \| 9.2E-5 \| \| GOTERM_BP_FAT \| [cellular defense response](http://www.ebi.ac.uk/QuickGO/GTerm?id=GO:0006968) \| 18 \| 1.4 \| 2.1E-6 \| 3.8 \| 1.0E-4 \| \| GOTERM_BP_FAT \| [cell cycle](http://www.ebi.ac.uk/QuickGO/GTerm?id=GO:0007049) \| 96 \| 7.4 \| 3.0E-6 \| 1.6 \| 1.5E-4 \| \| GOTERM_BP_FAT \| [positive regulation of cytokine production](http://www.ebi.ac.uk/QuickGO/GTerm?id=GO:0001819) \| 22 \| 1.7 \| 3.3E-6 \| 3.2 \| 1.6E-4 \| \| GOTERM_BP_FAT \| [regulation of lymphocyte differentiation](http://www.ebi.ac.uk/QuickGO/GTerm?id=GO:0045619) \| 18 \| 1.4 \| 3.4E-6 \| 3.7 \| 1.6E-4 \| \| GOTERM_BP_FAT \| [antigen processing and presentation of exogenous peptide antigen](http://www.ebi.ac.uk/QuickGO/GTerm?id=GO:0002478) \| 8 \| 0.6 \| 3.9E-6 \| 9.5 \| 1.8E-4 \| \| GOTERM_BP_FAT \| [T cell selection](http://www.ebi.ac.uk/QuickGO/GTerm?id=GO:0045058) \| 10 \| 0.8 \| 4.1E-6 \| 6.8 \| 1.9E-4 \| \| GOTERM_BP_FAT \| [regulation of I-kappaB kinase/NF-kappaB cascade](http://www.ebi.ac.uk/QuickGO/GTerm?id=GO:0043122) \| 24 \| 1.8 \| 5.1E-6 \| 2.9 \| 2.3E-4 \| \| GOTERM_BP_FAT \| [regulation of protein kinase cascade](http://www.ebi.ac.uk/QuickGO/GTerm?id=GO:0010627) \| 41 \| 3.1 \| 6.4E-6 \| 2.1 \| 2.8E-4 \| \| GOTERM_BP_FAT \| [positive regulation of defense response](http://www.ebi.ac.uk/QuickGO/GTerm?id=GO:0031349) \| 19 \| 1.5 \| 7.2E-6 \| 3.4 \| 3.2E-4 \| \| GOTERM_BP_FAT \| [vasculature development](http://www.ebi.ac.uk/QuickGO/GTerm?id=GO:0001944) \| 41 \| 3.1 \| 7.7E-6 \| 2.1 \| 3.4E-4 \| \| GOTERM_BP_FAT \| [response to oxygen levels](http://www.ebi.ac.uk/QuickGO/GTerm?id=GO:0070482) \| 28 \| 2.1 \| 8.1E-6 \| 2.6 \| 3.5E-4 \| \| GOTERM_BP_FAT \| [negative regulation of apoptosis](http://www.ebi.ac.uk/QuickGO/GTerm?id=GO:0043066) \| 52 \| 4.0 \| 9.2E-6 \| 1.9 \| 3.9E-4 \| \| GOTERM_BP_FAT \| [blood vessel development](http://www.ebi.ac.uk/QuickGO/GTerm?id=GO:0001568) \| 40 \| 3.1 \| 1.0E-5 \| 2.1 \| 4.3E-4 \| \| GOTERM_BP_FAT \| [blood vessel morphogenesis](http://www.ebi.ac.uk/QuickGO/GTerm?id=GO:0048514) \| 36 \| 2.8 \| 1.2E-5 \| 2.2 \| 4.9E-4 \| \| GOTERM_BP_FAT \| [positive regulation of alpha-beta T cell activation](http://www.ebi.ac.uk/QuickGO/GTerm?id=GO:0046635) \| 11 \| 0.8 \| 1.2E-5 \| 5.5 \| 4.8E-4 \| \| GOTERM_BP_FAT \| [protein complex biogenesis](http://www.ebi.ac.uk/QuickGO/GTerm?id=GO:0070271) \| 67 \| 5.1 \| 1.3E-5 \| 1.7 \| 5.4E-4 \| \| GOTERM_BP_FAT \| [protein complex assembly](http://www.ebi.ac.uk/QuickGO/GTerm?id=GO:0006461) \| 67 \| 5.1 \| 1.3E-5 \| 1.7 \| 5.4E-4 \| \| GOTERM_BP_FAT \| [negative regulation of programmed cell death](http://www.ebi.ac.uk/QuickGO/GTerm?id=GO:0043069) \| 52 \| 4.0 \| 1.4E-5 \| 1.9 \| 5.5E-4 \| \| GOTERM_BP_FAT \| [response to defenses of other organism during symbiotic interaction](http://www.ebi.ac.uk/QuickGO/GTerm?id=GO:0052173) \| 7 \| 0.5 \| 1.4E-5 \| 10.1 \| 5.6E-4 \| \| GOTERM_BP_FAT \| [response to host defenses](http://www.ebi.ac.uk/QuickGO/GTerm?id=GO:0052200) \| 7 \| 0.5 \| 1.4E-5 \| 10.1 \| 5.6E-4 \| \| GOTERM_BP_FAT \| [response to host](http://www.ebi.ac.uk/QuickGO/GTerm?id=GO:0075136) \| 7 \| 0.5 \| 1.4E-5 \| 10.1 \| 5.6E-4 \| \| GOTERM_BP_FAT \| [negative regulation of cell death](http://www.ebi.ac.uk/QuickGO/GTerm?id=GO:0060548) \| 52 \| 4.0 \| 1.5E-5 \| 1.9 \| 5.8E-4 \| \| GOTERM_BP_FAT \| [modulation by symbiont of host defense response](http://www.ebi.ac.uk/QuickGO/GTerm?id=GO:0052031) \| 6 \| 0.5 \| 1.5E-5 \| 13.0 \| 5.8E-4 \| \| GOTERM_BP_FAT \| [positive regulation by symbiont of host defense response](http://www.ebi.ac.uk/QuickGO/GTerm?id=GO:0052509) \| 6 \| 0.5 \| 1.5E-5 \| 13.0 \| 5.8E-4 \| \| GOTERM_BP_FAT \| [modulation by organism of defense response of other organism during symbiotic interaction](http://www.ebi.ac.uk/QuickGO/GTerm?id=GO:0052255) \| 6 \| 0.5 \| 1.5E-5 \| 13.0 \| 5.8E-4 \| \| GOTERM_BP_FAT \| [positive regulation by organism of defense response of other organism during symbiotic interaction](http://www.ebi.ac.uk/QuickGO/GTerm?id=GO:0052510) \| 6 \| 0.5 \| 1.5E-5 \| 13.0 \| 5.8E-4 \| \| GOTERM_BP_FAT \| [T cell proliferation](http://www.ebi.ac.uk/QuickGO/GTerm?id=GO:0042098) \| 11 \| 0.8 \| 1.7E-5 \| 5.3 \| 6.7E-4 \| \| GOTERM_BP_FAT \| [T cell differentiation in the thymus](http://www.ebi.ac.uk/QuickGO/GTerm?id=GO:0033077) \| 11 \| 0.8 \| 1.7E-5 \| 5.3 \| 6.7E-4 \| \| GOTERM_BP_FAT \| [thymic T cell selection](http://www.ebi.ac.uk/QuickGO/GTerm?id=GO:0045061) \| 8 \| 0.6 \| 1.8E-5 \| 8.0 \| 6.7E-4 \| \| GOTERM_BP_FAT \| [response to DNA damage stimulus](http://www.ebi.ac.uk/QuickGO/GTerm?id=GO:0006974) \| 53 \| 4.1 \| 1.9E-5 \| 1.8 \| 7.2E-4 \| \| GOTERM_BP_FAT \| [regulation of B cell activation](http://www.ebi.ac.uk/QuickGO/GTerm?id=GO:0050864) \| 15 \| 1.1 \| 2.0E-5 \| 3.8 \| 7.5E-4 \| \| GOTERM_BP_FAT \| [regulation of protein kinase activity](http://www.ebi.ac.uk/QuickGO/GTerm?id=GO:0045859) \| 50 \| 3.8 \| 2.0E-5 \| 1.9 \| 7.4E-4 \| \| GOTERM_BP_FAT \| [angiogenesis](http://www.ebi.ac.uk/QuickGO/GTerm?id=GO:0001525) \| 28 \| 2.1 \| 2.0E-5 \| 2.5 \| 7.4E-4 \| \| GOTERM_BP_FAT \| [positive regulation of multicellular organismal process](http://www.ebi.ac.uk/QuickGO/GTerm?id=GO:0051240) \| 39 \| 3.0 \| 2.2E-5 \| 2.1 \| 8.0E-4 \| \| GOTERM_BP_FAT \| [immune response-activating cell surface receptor signaling pathway](http://www.ebi.ac.uk/QuickGO/GTerm?id=GO:0002429) \| 13 \| 1.0 \| 2.2E-5 \| 4.3 \| 8.0E-4 \| \| GOTERM_BP_FAT \| [DNA-dependent DNA replication](http://www.ebi.ac.uk/QuickGO/GTerm?id=GO:0006261) \| 16 \| 1.2 \| 2.2E-5 \| 3.6 \| 7.9E-4 \| \| GOTERM_BP_FAT \| [response to virus](http://www.ebi.ac.uk/QuickGO/GTerm?id=GO:0009615) \| 23 \| 1.8 \| 2.3E-5 \| 2.7 \| 8.2E-4 \| \| GOTERM_BP_FAT \| [regulation of kinase activity](http://www.ebi.ac.uk/QuickGO/GTerm?id=GO:0043549) \| 51 \| 3.9 \| 2.4E-5 \| 1.9 \| 8.4E-4 \| \| GOTERM_BP_FAT \| [response to organic substance](http://www.ebi.ac.uk/QuickGO/GTerm?id=GO:0010033) \| 87 \| 6.7 \| 2.4E-5 \| 1.6 \| 8.3E-4 \| \| GOTERM_BP_FAT \| [cellular response to stress](http://www.ebi.ac.uk/QuickGO/GTerm?id=GO:0033554) \| 72 \| 5.5 \| 2.5E-5 \| 1.7 \| 8.7E-4 \| \| GOTERM_BP_FAT \| [myeloid cell activation during immune response](http://www.ebi.ac.uk/QuickGO/GTerm?id=GO:0002275) \| 9 \| 0.7 \| 2.6E-5 \| 6.5 \| 8.7E-4 \| \| GOTERM_BP_FAT \| [aging](http://www.ebi.ac.uk/QuickGO/GTerm?id=GO:0007568) \| 23 \| 1.8 \| 2.7E-5 \| 2.7 \| 9.1E-4 \| \| GOTERM_BP_FAT \| [response to hypoxia](http://www.ebi.ac.uk/QuickGO/GTerm?id=GO:0001666) \| 26 \| 2.0 \| 2.8E-5 \| 2.5 \| 9.1E-4 \| \| GOTERM_BP_FAT \| [regulation of cell motion](http://www.ebi.ac.uk/QuickGO/GTerm?id=GO:0051270) \| 33 \| 2.5 \| 2.8E-5 \| 2.2 \| 9.1E-4 \| \| GOTERM_BP_FAT \| [regulation of cell cycle](http://www.ebi.ac.uk/QuickGO/GTerm?id=GO:0051726) \| 48 \| 3.7 \| 2.9E-5 \| 1.9 \| 9.6E-4 \| \| GOTERM_BP_FAT \| [chemotaxis](http://www.ebi.ac.uk/QuickGO/GTerm?id=GO:0006935) \| 29 \| 2.2 \| 3.2E-5 \| 2.4 \| 1.0E-3 \| \| GOTERM_BP_FAT \| [taxis](http://www.ebi.ac.uk/QuickGO/GTerm?id=GO:0042330) \| 29 \| 2.2 \| 3.2E-5 \| 2.4 \| 1.0E-3 \| \| GOTERM_BP_FAT \| [antigen processing and presentation of exogenous antigen](http://www.ebi.ac.uk/QuickGO/GTerm?id=GO:0019884) \| 8 \| 0.6 \| 3.3E-5 \| 7.4 \| 1.1E-3 \| \| GOTERM_BP_FAT \| [small GTPase mediated signal transduction](http://www.ebi.ac.uk/QuickGO/GTerm?id=GO:0007264) \| 45 \| 3.4 \| 3.6E-5 \| 1.9 \| 1.1E-3 \| \| GOTERM_BP_FAT \| [positive regulation of lymphocyte differentiation](http://www.ebi.ac.uk/QuickGO/GTerm?id=GO:0045621) \| 12 \| 0.9 \| 3.9E-5 \| 4.5 \| 1.2E-3 \| \| GOTERM_BP_FAT \| [positive regulation of I-kappaB kinase/NF-kappaB cascade](http://www.ebi.ac.uk/QuickGO/GTerm?id=GO:0043123) \| 21 \| 1.6 \| 3.9E-5 \| 2.8 \| 1.2E-3 \| \| GOTERM_BP_FAT \| [positive regulation of cell proliferation](http://www.ebi.ac.uk/QuickGO/GTerm?id=GO:0008284) \| 56 \| 4.3 \| 4.4E-5 \| 1.8 \| 1.4E-3 \| \| GOTERM_BP_FAT \| [positive regulation of adaptive immune response based on somatic recombination of immune receptors built from immunoglobulin superfamily domains](http://www.ebi.ac.uk/QuickGO/GTerm?id=GO:0002824) \| 11 \| 0.8 \| 5.0E-5 \| 4.8 \| 1.5E-3 \| \| GOTERM_BP_FAT \| [cell cycle checkpoint](http://www.ebi.ac.uk/QuickGO/GTerm?id=GO:0000075) \| 20 \| 1.5 \| 5.1E-5 \| 2.9 \| 1.5E-3 \| \| GOTERM_BP_FAT \| [regulation of T cell proliferation](http://www.ebi.ac.uk/QuickGO/GTerm?id=GO:0042129) \| 16 \| 1.2 \| 5.2E-5 \| 3.4 \| 1.6E-3 \| \| GOTERM_BP_FAT \| [mitotic cell cycle](http://www.ebi.ac.uk/QuickGO/GTerm?id=GO:0000278) \| 51 \| 3.9 \| 6.2E-5 \| 1.8 \| 1.9E-3 \| \| GOTERM_BP_FAT \| [B cell mediated immunity](http://www.ebi.ac.uk/QuickGO/GTerm?id=GO:0019724) \| 15 \| 1.1 \| 6.3E-5 \| 3.5 \| 1.9E-3 \| \| GOTERM_BP_FAT \| [positive regulation of leukocyte proliferation](http://www.ebi.ac.uk/QuickGO/GTerm?id=GO:0070665) \| 15 \| 1.1 \| 6.3E-5 \| 3.5 \| 1.9E-3 \| \| GOTERM_BP_FAT \| [positive regulation of mononuclear cell proliferation](http://www.ebi.ac.uk/QuickGO/GTerm?id=GO:0032946) \| 15 \| 1.1 \| 6.3E-5 \| 3.5 \| 1.9E-3 \| \| GOTERM_BP_FAT \| [negative regulation of cell adhesion](http://www.ebi.ac.uk/QuickGO/GTerm?id=GO:0007162) \| 13 \| 1.0 \| 6.6E-5 \| 3.9 \| 1.9E-3 \| \| GOTERM_BP_FAT \| [positive regulation of adaptive immune response](http://www.ebi.ac.uk/QuickGO/GTerm?id=GO:0002821) \| 11 \| 0.8 \| 6.9E-5 \| 4.6 \| 2.0E-3 \| \| GOTERM_BP_FAT \| [positive regulation of protein kinase cascade](http://www.ebi.ac.uk/QuickGO/GTerm?id=GO:0010740) \| 29 \| 2.2 \| 7.0E-5 \| 2.3 \| 2.0E-3 \| \| GOTERM_BP_FAT \| [regulation of transferase activity](http://www.ebi.ac.uk/QuickGO/GTerm?id=GO:0051338) \| 51 \| 3.9 \| 7.2E-5 \| 1.8 \| 2.0E-3 \| \| GOTERM_BP_FAT \| [regulation of immune effector process](http://www.ebi.ac.uk/QuickGO/GTerm?id=GO:0002697) \| 21 \| 1.6 \| 7.2E-5 \| 2.7 \| 2.0E-3 \| \| GOTERM_BP_FAT \| [response to molecule of bacterial origin](http://www.ebi.ac.uk/QuickGO/GTerm?id=GO:0002237) \| 19 \| 1.5 \| 7.7E-5 \| 2.9 \| 2.2E-3 \| \| GOTERM_BP_FAT \| [cell cycle process](http://www.ebi.ac.uk/QuickGO/GTerm?id=GO:0022402) \| 70 \| 5.4 \| 7.7E-5 \| 1.6 \| 2.2E-3 \| \| GOTERM_BP_FAT \| [leukocyte proliferation](http://www.ebi.ac.uk/QuickGO/GTerm?id=GO:0070661) \| 13 \| 1.0 \| 8.4E-5 \| 3.8 \| 2.3E-3 \| \| GOTERM_BP_FAT \| [mononuclear cell proliferation](http://www.ebi.ac.uk/QuickGO/GTerm?id=GO:0032943) \| 13 \| 1.0 \| 8.4E-5 \| 3.8 \| 2.3E-3 \| \| GOTERM_BP_FAT \| [cell cycle phase](http://www.ebi.ac.uk/QuickGO/GTerm?id=GO:0022403) \| 55 \| 4.2 \| 8.4E-5 \| 1.7 \| 2.3E-3 \| \| GOTERM_BP_FAT \| [DNA metabolic process](http://www.ebi.ac.uk/QuickGO/GTerm?id=GO:0006259) \| 64 \| 4.9 \| 8.9E-5 \| 1.6 \| 2.4E-3 \| \| GOTERM_BP_FAT \| [regulation of T cell differentiation](http://www.ebi.ac.uk/QuickGO/GTerm?id=GO:0045580) \| 14 \| 1.1 \| 9.2E-5 \| 3.6 \| 2.5E-3 \| \| GOTERM_BP_FAT \| [negative regulation of mononuclear cell proliferation](http://www.ebi.ac.uk/QuickGO/GTerm?id=GO:0032945) \| 11 \| 0.8 \| 9.3E-5 \| 4.5 \| 2.5E-3 \| \| GOTERM_BP_FAT \| [negative regulation of lymphocyte proliferation](http://www.ebi.ac.uk/QuickGO/GTerm?id=GO:0050672) \| 11 \| 0.8 \| 9.3E-5 \| 4.5 \| 2.5E-3 \| \| GOTERM_BP_FAT \| [negative regulation of leukocyte proliferation](http://www.ebi.ac.uk/QuickGO/GTerm?id=GO:0070664) \| 11 \| 0.8 \| 9.3E-5 \| 4.5 \| 2.5E-3 \| \| GOTERM_BP_FAT \| [anti-apoptosis](http://www.ebi.ac.uk/QuickGO/GTerm?id=GO:0006916) \| 33 \| 2.5 \| 1.0E-4 \| 2.1 \| 2.7E-3 \| \| GOTERM_BP_FAT \| [macrophage activation during immune response](http://www.ebi.ac.uk/QuickGO/GTerm?id=GO:0002281) \| 6 \| 0.5 \| 1.2E-4 \| 9.7 \| 3.2E-3 \| \| GOTERM_BP_FAT \| [regulation of protein amino acid phosphorylation](http://www.ebi.ac.uk/QuickGO/GTerm?id=GO:0001932) \| 29 \| 2.2 \| 1.3E-4 \| 2.2 \| 3.5E-3 \| \| GOTERM_BP_FAT \| [positive regulation of molecular function](http://www.ebi.ac.uk/QuickGO/GTerm?id=GO:0044093) \| 71 \| 5.4 \| 1.3E-4 \| 1.6 \| 3.5E-3 \| \| GOTERM_BP_FAT \| [antigen processing and presentation of peptide antigen via MHC class I](http://www.ebi.ac.uk/QuickGO/GTerm?id=GO:0002474) \| 8 \| 0.6 \| 1.5E-4 \| 6.1 \| 3.9E-3 \| \| GOTERM_BP_FAT \| [response to bacterium](http://www.ebi.ac.uk/QuickGO/GTerm?id=GO:0009617) \| 31 \| 2.4 \| 1.6E-4 \| 2.1 \| 4.2E-3 \| \| GOTERM_BP_FAT \| [modulation by symbiont of host innate immunity](http://www.ebi.ac.uk/QuickGO/GTerm?id=GO:0052167) \| 5 \| 0.4 \| 1.6E-4 \| 13.0 \| 4.2E-3 \| \| GOTERM_BP_FAT \| [microglial cell activation during immune response](http://www.ebi.ac.uk/QuickGO/GTerm?id=GO:0002282) \| 5 \| 0.4 \| 1.6E-4 \| 13.0 \| 4.2E-3 \| \| GOTERM_BP_FAT \| [modulation by organism of innate immunity in other organism during symbiotic interaction](http://www.ebi.ac.uk/QuickGO/GTerm?id=GO:0052306) \| 5 \| 0.4 \| 1.6E-4 \| 13.0 \| 4.2E-3 \| \| GOTERM_BP_FAT \| [modulation by organism of immune response of other organism during symbiotic interaction](http://www.ebi.ac.uk/QuickGO/GTerm?id=GO:0052552) \| 5 \| 0.4 \| 1.6E-4 \| 13.0 \| 4.2E-3 \| \| GOTERM_BP_FAT \| [positive regulation by symbiont of host innate immunity](http://www.ebi.ac.uk/QuickGO/GTerm?id=GO:0052166) \| 5 \| 0.4 \| 1.6E-4 \| 13.0 \| 4.2E-3 \| \| GOTERM_BP_FAT \| [positive regulation by organism of innate immunity in other organism during symbiotic interaction](http://www.ebi.ac.uk/QuickGO/GTerm?id=GO:0052305) \| 5 \| 0.4 \| 1.6E-4 \| 13.0 \| 4.2E-3 \| \| GOTERM_BP_FAT \| [positive regulation by organism of immune response of other organism during symbiotic interaction](http://www.ebi.ac.uk/QuickGO/GTerm?id=GO:0052555) \| 5 \| 0.4 \| 1.6E-4 \| 13.0 \| 4.2E-3 \| \| GOTERM_BP_FAT \| [modulation by symbiont of host immune response](http://www.ebi.ac.uk/QuickGO/GTerm?id=GO:0052553) \| 5 \| 0.4 \| 1.6E-4 \| 13.0 \| 4.2E-3 \| \| GOTERM_BP_FAT \| [positive regulation by symbiont of host immune response](http://www.ebi.ac.uk/QuickGO/GTerm?id=GO:0052556) \| 5 \| 0.4 \| 1.6E-4 \| 13.0 \| 4.2E-3 \| \| GOTERM_BP_FAT \| [leukocyte adhesion](http://www.ebi.ac.uk/QuickGO/GTerm?id=GO:0007159) \| 10 \| 0.8 \| 1.6E-4 \| 4.6 \| 4.2E-3 \| \| GOTERM_BP_FAT \| [regulation of B cell proliferation](http://www.ebi.ac.uk/QuickGO/GTerm?id=GO:0030888) \| 10 \| 0.8 \| 1.6E-4 \| 4.6 \| 4.2E-3 \| \| GOTERM_BP_FAT \| [regulation of innate immune response](http://www.ebi.ac.uk/QuickGO/GTerm?id=GO:0045088) \| 14 \| 1.1 \| 1.7E-4 \| 3.4 \| 4.3E-3 \| \| GOTERM_BP_FAT \| [immunoglobulin mediated immune response](http://www.ebi.ac.uk/QuickGO/GTerm?id=GO:0016064) \| 14 \| 1.1 \| 1.7E-4 \| 3.4 \| 4.3E-3 \| \| GOTERM_BP_FAT \| [B cell activation](http://www.ebi.ac.uk/QuickGO/GTerm?id=GO:0042113) \| 17 \| 1.3 \| 1.8E-4 \| 2.9 \| 4.4E-3 \| \| GOTERM_BP_FAT \| [homeostasis of number of cells](http://www.ebi.ac.uk/QuickGO/GTerm?id=GO:0048872) \| 20 \| 1.5 \| 1.9E-4 \| 2.6 \| 4.8E-3 \| \| GOTERM_BP_FAT \| [positive regulation of cytokine biosynthetic process](http://www.ebi.ac.uk/QuickGO/GTerm?id=GO:0042108) \| 13 \| 1.0 \| 2.1E-4 \| 3.5 \| 5.1E-3 \| \| GOTERM_BP_FAT \| [positive regulation of lymphocyte proliferation](http://www.ebi.ac.uk/QuickGO/GTerm?id=GO:0050671) \| 14 \| 1.1 \| 2.1E-4 \| 3.3 \| 5.1E-3 \| \| GOTERM_BP_FAT \| [I-kappaB kinase/NF-kappaB cascade](http://www.ebi.ac.uk/QuickGO/GTerm?id=GO:0007249) \| 15 \| 1.1 \| 2.4E-4 \| 3.1 \| 5.9E-3 \| \| GOTERM_BP_FAT \| [lymphocyte proliferation](http://www.ebi.ac.uk/QuickGO/GTerm?id=GO:0046651) \| 12 \| 0.9 \| 2.4E-4 \| 3.7 \| 5.9E-3 \| \| GOTERM_BP_FAT \| [negative regulation of cell proliferation](http://www.ebi.ac.uk/QuickGO/GTerm?id=GO:0008285) \| 48 \| 3.7 \| 2.5E-4 \| 1.7 \| 6.0E-3 \| \| GOTERM_BP_FAT \| [modification by symbiont of host morphology or physiology](http://www.ebi.ac.uk/QuickGO/GTerm?id=GO:0044003) \| 6 \| 0.5 \| 2.6E-4 \| 8.7 \| 6.1E-3 \| \| GOTERM_BP_FAT \| [actin cytoskeleton organization](http://www.ebi.ac.uk/QuickGO/GTerm?id=GO:0030036) \| 34 \| 2.6 \| 2.6E-4 \| 2.0 \| 6.2E-3 \| \| GOTERM_BP_FAT \| [cell motion](http://www.ebi.ac.uk/QuickGO/GTerm?id=GO:0006928) \| 59 \| 4.5 \| 2.8E-4 \| 1.6 \| 6.6E-3 \| \| GOTERM_BP_FAT \| [cytoskeleton organization](http://www.ebi.ac.uk/QuickGO/GTerm?id=GO:0007010) \| 55 \| 4.2 \| 3.2E-4 \| 1.6 \| 7.4E-3 \| \| GOTERM_BP_FAT \| [DNA damage response. signal transduction](http://www.ebi.ac.uk/QuickGO/GTerm?id=GO:0042770) \| 17 \| 1.3 \| 3.3E-4 \| 2.8 \| 7.6E-3 \| \| GOTERM_BP_FAT \| [positive regulation of catalytic activity](http://www.ebi.ac.uk/QuickGO/GTerm?id=GO:0043085) \| 63 \| 4.8 \| 3.3E-4 \| 1.6 \| 7.7E-3 \| \| GOTERM_BP_FAT \| [organelle fission](http://www.ebi.ac.uk/QuickGO/GTerm?id=GO:0048285) \| 34 \| 2.6 \| 3.3E-4 \| 1.9 \| 7.7E-3 \| \| GOTERM_BP_FAT \| [activation of innate immune response](http://www.ebi.ac.uk/QuickGO/GTerm?id=GO:0002218) \| 8 \| 0.6 \| 3.4E-4 \| 5.5 \| 7.8E-3 \| \| GOTERM_BP_FAT \| [innate immune response-activating signal transduction](http://www.ebi.ac.uk/QuickGO/GTerm?id=GO:0002758) \| 8 \| 0.6 \| 3.4E-4 \| 5.5 \| 7.8E-3 \| \| GOTERM_BP_FAT \| [nuclear division](http://www.ebi.ac.uk/QuickGO/GTerm?id=GO:0000280) \| 33 \| 2.5 \| 3.5E-4 \| 1.9 \| 7.8E-3 \| \| GOTERM_BP_FAT \| [mitosis](http://www.ebi.ac.uk/QuickGO/GTerm?id=GO:0007067) \| 33 \| 2.5 \| 3.5E-4 \| 1.9 \| 7.8E-3 \| \| GOTERM_BP_FAT \| [protein oligomerization](http://www.ebi.ac.uk/QuickGO/GTerm?id=GO:0051259) \| 28 \| 2.1 \| 3.5E-4 \| 2.1 \| 7.8E-3 \| \| GOTERM_BP_FAT \| [toll-like receptor signaling pathway](http://www.ebi.ac.uk/QuickGO/GTerm?id=GO:0002224) \| 7 \| 0.5 \| 3.6E-4 \| 6.5 \| 7.9E-3 \| \| GOTERM_BP_FAT \| [regulation of homeostatic process](http://www.ebi.ac.uk/QuickGO/GTerm?id=GO:0032844) \| 21 \| 1.6 \| 4.0E-4 \| 2.4 \| 8.8E-3 \| \| GOTERM_BP_FAT \| [M phase](http://www.ebi.ac.uk/QuickGO/GTerm?id=GO:0000279) \| 44 \| 3.4 \| 4.2E-4 \| 1.7 \| 9.2E-3 \| \| GOTERM_BP_FAT \| [response to host immune response](http://www.ebi.ac.uk/QuickGO/GTerm?id=GO:0052572) \| 5 \| 0.4 \| 4.6E-4 \| 10.8 \| 1.0E-2 \| \| GOTERM_BP_FAT \| [response to immune response of other organism during symbiotic interaction](http://www.ebi.ac.uk/QuickGO/GTerm?id=GO:0052564) \| 5 \| 0.4 \| 4.6E-4 \| 10.8 \| 1.0E-2 \| \| GOTERM_BP_FAT \| [regulation of response to external stimulus](http://www.ebi.ac.uk/QuickGO/GTerm?id=GO:0032101) \| 26 \| 2.0 \| 4.6E-4 \| 2.1 \| 1.0E-2 \| \| GOTERM_BP_FAT \| [M phase of mitotic cell cycle](http://www.ebi.ac.uk/QuickGO/GTerm?id=GO:0000087) \| 33 \| 2.5 \| 4.8E-4 \| 1.9 \| 1.0E-2 \| \| GOTERM_BP_FAT \| [regulation of T-helper 2 type immune response](http://www.ebi.ac.uk/QuickGO/GTerm?id=GO:0002828) \| 6 \| 0.5 \| 4.8E-4 \| 7.8 \| 1.0E-2 \| \| GOTERM_BP_FAT \| [regulation of calcium-mediated signaling](http://www.ebi.ac.uk/QuickGO/GTerm?id=GO:0050848) \| 8 \| 0.6 \| 4.9E-4 \| 5.2 \| 1.1E-2 \| \| GOTERM_BP_FAT \| [protein amino acid phosphorylation](http://www.ebi.ac.uk/QuickGO/GTerm?id=GO:0006468) \| 76 \| 5.8 \| 5.0E-4 \| 1.5 \| 1.1E-2 \| \| GOTERM_BP_FAT \| [positive regulation of T cell differentiation](http://www.ebi.ac.uk/QuickGO/GTerm?id=GO:0045582) \| 10 \| 0.8 \| 5.0E-4 \| 4.1 \| 1.1E-2 \| \| GOTERM_BP_FAT \| [cell adhesion](http://www.ebi.ac.uk/QuickGO/GTerm?id=GO:0007155) \| 79 \| 6.0 \| 5.1E-4 \| 1.5 \| 1.1E-2 \| \| GOTERM_BP_FAT \| [biological adhesion](http://www.ebi.ac.uk/QuickGO/GTerm?id=GO:0022610) \| 79 \| 6.0 \| 5.3E-4 \| 1.5 \| 1.1E-2 \| \| GOTERM_BP_FAT \| [regulation of CD4-positive. alpha beta T cell differentiation](http://www.ebi.ac.uk/QuickGO/GTerm?id=GO:0043370) \| 7 \| 0.5 \| 5.6E-4 \| 6.1 \| 1.2E-2 \| \| GOTERM_BP_FAT \| [positive regulation of tumor necrosis factor production](http://www.ebi.ac.uk/QuickGO/GTerm?id=GO:0032760) \| 7 \| 0.5 \| 5.6E-4 \| 6.1 \| 1.2E-2 \| \| GOTERM_BP_FAT \| [macrophage activation](http://www.ebi.ac.uk/QuickGO/GTerm?id=GO:0042116) \| 7 \| 0.5 \| 5.6E-4 \| 6.1 \| 1.2E-2 \| \| GOTERM_BP_FAT \| [regulation of mitotic cell cycle](http://www.ebi.ac.uk/QuickGO/GTerm?id=GO:0007346) \| 25 \| 1.9 \| 5.6E-4 \| 2.1 \| 1.2E-2 \| \| GOTERM_BP_FAT \| [cell division](http://www.ebi.ac.uk/QuickGO/GTerm?id=GO:0051301) \| 40 \| 3.1 \| 6.0E-4 \| 1.8 \| 1.2E-2 \| \| GOTERM_BP_FAT \| [regulation of protein modification process](http://www.ebi.ac.uk/QuickGO/GTerm?id=GO:0031399) \| 40 \| 3.1 \| 6.0E-4 \| 1.8 \| 1.2E-2 \| \| GOTERM_BP_FAT \| [regulation of leukocyte mediated immunity](http://www.ebi.ac.uk/QuickGO/GTerm?id=GO:0002703) \| 14 \| 1.1 \| 6.2E-4 \| 3.0 \| 1.3E-2 \| \| GOTERM_BP_FAT \| [positive regulation of B cell activation](http://www.ebi.ac.uk/QuickGO/GTerm?id=GO:0050871) \| 10 \| 0.8 \| 6.5E-4 \| 3.9 \| 1.3E-2 \| \| GOTERM_BP_FAT \| [regulation of lymphocyte mediated immunity](http://www.ebi.ac.uk/QuickGO/GTerm?id=GO:0002706) \| 13 \| 1.0 \| 6.7E-4 \| 3.1 \| 1.3E-2 \| \| GOTERM_BP_FAT \| [induction of apoptosis by intracellular signals](http://www.ebi.ac.uk/QuickGO/GTerm?id=GO:0008629) \| 13 \| 1.0 \| 6.7E-4 \| 3.1 \| 1.3E-2 \| \| GOTERM_BP_FAT \| [negative regulation of T cell proliferation](http://www.ebi.ac.uk/QuickGO/GTerm?id=GO:0042130) \| 9 \| 0.7 \| 7.0E-4 \| 4.3 \| 1.4E-2 \| \| GOTERM_BP_FAT \| [regulation of cell adhesion](http://www.ebi.ac.uk/QuickGO/GTerm?id=GO:0030155) \| 23 \| 1.8 \| 7.4E-4 \| 2.2 \| 1.5E-2 \| \| GOTERM_BP_FAT \| [integrin-mediated signaling pathway](http://www.ebi.ac.uk/QuickGO/GTerm?id=GO:0007229) \| 15 \| 1.1 \| 7.6E-4 \| 2.8 \| 1.5E-2 \| \| GOTERM_BP_FAT \| [regulation of cellular response to stress](http://www.ebi.ac.uk/QuickGO/GTerm?id=GO:0080135) \| 19 \| 1.5 \| 8.0E-4 \| 2.4 \| 1.6E-2 \| \| GOTERM_BP_FAT \| [positive regulation of CD4-positive. alpha beta T cell differentiation](http://www.ebi.ac.uk/QuickGO/GTerm?id=GO:0043372) \| 6 \| 0.5 \| 8.3E-4 \| 7.1 \| 1.6E-2 \| \| GOTERM_BP_FAT \| [DNA replication initiation](http://www.ebi.ac.uk/QuickGO/GTerm?id=GO:0006270) \| 7 \| 0.5 \| 8.3E-4 \| 5.7 \| 1.6E-2 \| \| GOTERM_BP_FAT \| [actin filament-based process](http://www.ebi.ac.uk/QuickGO/GTerm?id=GO:0030029) \| 34 \| 2.6 \| 8.4E-4 \| 1.8 \| 1.6E-2 \| \| GOTERM_BP_FAT \| [positive regulation of protein kinase activity](http://www.ebi.ac.uk/QuickGO/GTerm?id=GO:0045860) \| 32 \| 2.5 \| 9.2E-4 \| 1.9 \| 1.8E-2 \| \| GOTERM_BP_FAT \| [positive thymic T cell selection](http://www.ebi.ac.uk/QuickGO/GTerm?id=GO:0045059) \| 5 \| 0.4 \| 1.0E-3 \| 9.3 \| 1.9E-2 \| \| GOTERM_BP_FAT \| [microglial cell activation](http://www.ebi.ac.uk/QuickGO/GTerm?id=GO:0001774) \| 5 \| 0.4 \| 1.0E-3 \| 9.3 \| 1.9E-2 \| \| GOTERM_BP_FAT \| [regulation of MAP kinase activity](http://www.ebi.ac.uk/QuickGO/GTerm?id=GO:0043405) \| 23 \| 1.8 \| 1.1E-3 \| 2.1 \| 2.1E-2 \| \| GOTERM_BP_FAT \| [regulation of cell migration](http://www.ebi.ac.uk/QuickGO/GTerm?id=GO:0030334) \| 26 \| 2.0 \| 1.2E-3 \| 2.0 \| 2.2E-2 \| \| GOTERM_BP_FAT \| [pattern recognition receptor signaling pathway](http://www.ebi.ac.uk/QuickGO/GTerm?id=GO:0002221) \| 7 \| 0.5 \| 1.2E-3 \| 5.4 \| 2.3E-2 \| \| GOTERM_BP_FAT \| [positive regulation of calcium-mediated signaling](http://www.ebi.ac.uk/QuickGO/GTerm?id=GO:0050850) \| 7 \| 0.5 \| 1.2E-3 \| 5.4 \| 2.3E-2 \| \| GOTERM_BP_FAT \| [positive regulation of cell motion](http://www.ebi.ac.uk/QuickGO/GTerm?id=GO:0051272) \| 18 \| 1.4 \| 1.2E-3 \| 2.4 \| 2.2E-2 \| \| GOTERM_BP_FAT \| [cell migration](http://www.ebi.ac.uk/QuickGO/GTerm?id=GO:0016477) \| 37 \| 2.8 \| 1.2E-3 \| 1.7 \| 2.3E-2 \| \| GOTERM_BP_FAT \| [regulation of hydrolase activity](http://www.ebi.ac.uk/QuickGO/GTerm?id=GO:0051336) \| 43 \| 3.3 \| 1.3E-3 \| 1.7 \| 2.3E-2 \| \| GOTERM_BP_FAT \| [response to protein stimulus](http://www.ebi.ac.uk/QuickGO/GTerm?id=GO:0051789) \| 19 \| 1.5 \| 1.3E-3 \| 2.3 \| 2.3E-2 \| \| GOTERM_BP_FAT \| [response to abiotic stimulus](http://www.ebi.ac.uk/QuickGO/GTerm?id=GO:0009628) \| 46 \| 3.5 \| 1.3E-3 \| 1.6 \| 2.3E-2 \| \| GOTERM_BP_FAT \| [mitotic cell cycle checkpoint](http://www.ebi.ac.uk/QuickGO/GTerm?id=GO:0007093) \| 11 \| 0.8 \| 1.3E-3 \| 3.3 \| 2.3E-2 \| \| GOTERM_BP_FAT \| [regulation of antigen receptor-mediated signaling pathway](http://www.ebi.ac.uk/QuickGO/GTerm?id=GO:0050854) \| 6 \| 0.5 \| 1.3E-3 \| 6.5 \| 2.4E-2 \| \| GOTERM_BP_FAT \| [regulation of lymphocyte apoptosis](http://www.ebi.ac.uk/QuickGO/GTerm?id=GO:0070228) \| 6 \| 0.5 \| 1.3E-3 \| 6.5 \| 2.4E-2 \| \| GOTERM_BP_FAT \| [regulation of cytokine biosynthetic process](http://www.ebi.ac.uk/QuickGO/GTerm?id=GO:0042035) \| 15 \| 1.1 \| 1.3E-3 \| 2.6 \| 2.4E-2 \| \| GOTERM_BP_FAT \| [positive regulation of developmental process](http://www.ebi.ac.uk/QuickGO/GTerm?id=GO:0051094) \| 37 \| 2.8 \| 1.4E-3 \| 1.7 \| 2.5E-2 \| \| GOTERM_BP_FAT \| [positive regulation of cell differentiation](http://www.ebi.ac.uk/QuickGO/GTerm?id=GO:0045597) \| 32 \| 2.5 \| 1.4E-3 \| 1.8 \| 2.5E-2 \| \| GOTERM_BP_FAT \| [regulation of interleukin-2 production](http://www.ebi.ac.uk/QuickGO/GTerm?id=GO:0032663) \| 9 \| 0.7 \| 1.5E-3 \| 3.9 \| 2.7E-2 \| \| GOTERM_BP_FAT \| [regulation of interferon-gamma production](http://www.ebi.ac.uk/QuickGO/GTerm?id=GO:0032649) \| 9 \| 0.7 \| 1.5E-3 \| 3.9 \| 2.7E-2 \| \| GOTERM_BP_FAT \| [membrane invagination](http://www.ebi.ac.uk/QuickGO/GTerm?id=GO:0010324) \| 31 \| 2.4 \| 1.5E-3 \| 1.8 \| 2.7E-2 \| \| GOTERM_BP_FAT \| [endocytosis](http://www.ebi.ac.uk/QuickGO/GTerm?id=GO:0006897) \| 31 \| 2.4 \| 1.5E-3 \| 1.8 \| 2.7E-2 \| \| GOTERM_BP_FAT \| [positive regulation of immune effector process](http://www.ebi.ac.uk/QuickGO/GTerm?id=GO:0002699) \| 11 \| 0.8 \| 1.5E-3 \| 3.2 \| 2.7E-2 \| \| GOTERM_BP_FAT \| [positive regulation of kinase activity](http://www.ebi.ac.uk/QuickGO/GTerm?id=GO:0033674) \| 32 \| 2.5 \| 1.6E-3 \| 1.8 \| 2.9E-2 \| \| GOTERM_BP_FAT \| [lymphocyte activation during immune response](http://www.ebi.ac.uk/QuickGO/GTerm?id=GO:0002285) \| 7 \| 0.5 \| 1.7E-3 \| 5.1 \| 2.9E-2 \| \| GOTERM_BP_FAT \| [DNA integrity checkpoint](http://www.ebi.ac.uk/QuickGO/GTerm?id=GO:0031570) \| 12 \| 0.9 \| 1.7E-3 \| 3.0 \| 2.9E-2 \| \| GOTERM_BP_FAT \| [regulation of mast cell cytokine production](http://www.ebi.ac.uk/QuickGO/GTerm?id=GO:0032763) \| 4 \| 0.3 \| 1.7E-3 \| 13.0 \| 2.9E-2 \| \| GOTERM_BP_FAT \| [regulation of inflammatory response](http://www.ebi.ac.uk/QuickGO/GTerm?id=GO:0050727) \| 15 \| 1.1 \| 1.8E-3 \| 2.6 \| 3.0E-2 \| \| GOTERM_BP_FAT \| [response to ionizing radiation](http://www.ebi.ac.uk/QuickGO/GTerm?id=GO:0010212) \| 13 \| 1.0 \| 1.8E-3 \| 2.8 \| 3.0E-2 \| \| GOTERM_BP_FAT \| [myeloid cell differentiation](http://www.ebi.ac.uk/QuickGO/GTerm?id=GO:0030099) \| 17 \| 1.3 \| 1.8E-3 \| 2.4 \| 3.1E-2 \| \| GOTERM_BP_FAT \| [antigen processing and presentation of exogenous peptide antigen via MHC class II](http://www.ebi.ac.uk/QuickGO/GTerm?id=GO:0019886) \| 5 \| 0.4 \| 1.9E-3 \| 8.1 \| 3.2E-2 \| \| GOTERM_BP_FAT \| [regulation of B cell apoptosis](http://www.ebi.ac.uk/QuickGO/GTerm?id=GO:0002902) \| 5 \| 0.4 \| 1.9E-3 \| 8.1 \| 3.2E-2 \| \| GOTERM_BP_FAT \| [antigen processing and presentation of peptide antigen via MHC class II](http://www.ebi.ac.uk/QuickGO/GTerm?id=GO:0002495) \| 5 \| 0.4 \| 1.9E-3 \| 8.1 \| 3.2E-2 \| \| GOTERM_BP_FAT \| [negative thymic T cell selection](http://www.ebi.ac.uk/QuickGO/GTerm?id=GO:0045060) \| 5 \| 0.4 \| 1.9E-3 \| 8.1 \| 3.2E-2 \| \| GOTERM_BP_FAT \| [antigen processing and presentation of endogenous antigen](http://www.ebi.ac.uk/QuickGO/GTerm?id=GO:0019883) \| 5 \| 0.4 \| 1.9E-3 \| 8.1 \| 3.2E-2 \| \| GOTERM_BP_FAT \| [T cell activation during immune response](http://www.ebi.ac.uk/QuickGO/GTerm?id=GO:0002286) \| 6 \| 0.5 \| 2.0E-3 \| 6.0 \| 3.4E-2 \| \| GOTERM_BP_FAT \| [regulation of alpha-beta T cell proliferation](http://www.ebi.ac.uk/QuickGO/GTerm?id=GO:0046640) \| 6 \| 0.5 \| 2.0E-3 \| 6.0 \| 3.4E-2 \| \| GOTERM_BP_FAT \| [regulation of stress-activated protein kinase signaling pathway](http://www.ebi.ac.uk/QuickGO/GTerm?id=GO:0070302) \| 14 \| 1.1 \| 2.1E-3 \| 2.6 \| 3.4E-2 \| \| GOTERM_BP_FAT \| [regulation of alpha-beta T cell differentiation](http://www.ebi.ac.uk/QuickGO/GTerm?id=GO:0046637) \| 8 \| 0.6 \| 2.2E-3 \| 4.2 \| 3.6E-2 \| \| GOTERM_BP_FAT \| [positive regulation of innate immune response](http://www.ebi.ac.uk/QuickGO/GTerm?id=GO:0045089) \| 11 \| 0.8 \| 2.2E-3 \| 3.1 \| 3.6E-2 \| \| GOTERM_BP_FAT \| [cytokine production](http://www.ebi.ac.uk/QuickGO/GTerm?id=GO:0001816) \| 11 \| 0.8 \| 2.2E-3 \| 3.1 \| 3.6E-2 \| \| GOTERM_BP_FAT \| [protein homooligomerization](http://www.ebi.ac.uk/QuickGO/GTerm?id=GO:0051260) \| 17 \| 1.3 \| 2.3E-3 \| 2.3 \| 3.7E-2 \| \| GOTERM_BP_FAT \| [positive regulation of T cell proliferation](http://www.ebi.ac.uk/QuickGO/GTerm?id=GO:0042102) \| 10 \| 0.8 \| 2.3E-3 \| 3.3 \| 3.8E-2 \| \| GOTERM_BP_FAT \| [regulation of myeloid cell differentiation](http://www.ebi.ac.uk/QuickGO/GTerm?id=GO:0045637) \| 14 \| 1.1 \| 2.3E-3 \| 2.6 \| 3.8E-2 \| \| GOTERM_BP_FAT \| [cell motility](http://www.ebi.ac.uk/QuickGO/GTerm?id=GO:0048870) \| 39 \| 3.0 \| 2.4E-3 \| 1.7 \| 3.8E-2 \| \| GOTERM_BP_FAT \| [localization of cell](http://www.ebi.ac.uk/QuickGO/GTerm?id=GO:0051674) \| 39 \| 3.0 \| 2.4E-3 \| 1.7 \| 3.8E-2 \| \| GOTERM_BP_FAT \| [regulation of organelle organization](http://www.ebi.ac.uk/QuickGO/GTerm?id=GO:0033043) \| 30 \| 2.3 \| 2.4E-3 \| 1.8 \| 3.9E-2 \| \| GOTERM_BP_FAT \| [membrane organization](http://www.ebi.ac.uk/QuickGO/GTerm?id=GO:0016044) \| 46 \| 3.5 \| 2.5E-3 \| 1.6 \| 4.0E-2 \| \| GOTERM_BP_FAT \| [regulation of body fluid levels](http://www.ebi.ac.uk/QuickGO/GTerm?id=GO:0050878) \| 22 \| 1.7 \| 2.5E-3 \| 2.0 \| 4.0E-2 \| \| GOTERM_BP_FAT \| [Ras protein signal transduction](http://www.ebi.ac.uk/QuickGO/GTerm?id=GO:0007265) \| 18 \| 1.4 \| 2.6E-3 \| 2.2 \| 4.1E-2 \| \| GOTERM_BP_FAT \| [regulation of angiogenesis](http://www.ebi.ac.uk/QuickGO/GTerm?id=GO:0045765) \| 13 \| 1.0 \| 2.7E-3 \| 2.7 \| 4.3E-2 \| \| GOTERM_BP_FAT \| [cell aging](http://www.ebi.ac.uk/QuickGO/GTerm?id=GO:0007569) \| 9 \| 0.7 \| 2.9E-3 \| 3.5 \| 4.6E-2 \| \| GOTERM_BP_FAT \| [antigen receptor-mediated signaling pathway](http://www.ebi.ac.uk/QuickGO/GTerm?id=GO:0050851) \| 9 \| 0.7 \| 2.9E-3 \| 3.5 \| 4.6E-2 \| \| GOTERM_BP_FAT \| [antigen processing and presentation of peptide or polysaccharide antigen via MHC class II](http://www.ebi.ac.uk/QuickGO/GTerm?id=GO:0002504) \| 9 \| 0.7 \| 2.9E-3 \| 3.5 \| 4.6E-2 \| \| GOTERM_BP_FAT \| [positive regulation of transferase activity](http://www.ebi.ac.uk/QuickGO/GTerm?id=GO:0051347) \| 32 \| 2.5 \| 3.0E-3 \| 1.7 \| 4.7E-2 \| \| GOTERM_BP_FAT \| [DNA damage checkpoint](http://www.ebi.ac.uk/QuickGO/GTerm?id=GO:0000077) \| 11 \| 0.8 \| 3.1E-3 \| 3.0 \| 4.7E-2 \| \| GOTERM_BP_FAT \| [B cell differentiation](http://www.ebi.ac.uk/QuickGO/GTerm?id=GO:0030183) \| 11 \| 0.8 \| 3.1E-3 \| 3.0 \| 4.7E-2 \| \| GOTERM_BP_FAT \| [positive regulation of alpha-beta T cell differentiation](http://www.ebi.ac.uk/QuickGO/GTerm?id=GO:0046638) \| 7 \| 0.5 \| 3.1E-3 \| 4.5 \| 4.8E-2 \| \| GOTERM_BP_FAT \| [positive regulation of alpha-beta T cell proliferation](http://www.ebi.ac.uk/QuickGO/GTerm?id=GO:0046641) \| 5 \| 0.4 \| 3.2E-3 \| 7.2 \| 4.9E-2 \| \| GOTERM_BP_FAT \| [positive T cell selection](http://www.ebi.ac.uk/QuickGO/GTerm?id=GO:0043368) \| 5 \| 0.4 \| 3.2E-3 \| 7.2 \| 4.9E-2 \| \| GOTERM_BP_FAT \| [negative T cell selection](http://www.ebi.ac.uk/QuickGO/GTerm?id=GO:0043383) \| 5 \| 0.4 \| 3.2E-3 \| 7.2 \| 4.9E-2 \| \| GOTERM_BP_FAT \| [negative regulation of cell cycle](http://www.ebi.ac.uk/QuickGO/GTerm?id=GO:0045786) \| 15 \| 1.1 \| 3.3E-3 \| 2.4 \| 5.0E-2 \| \| GOTERM_BP_FAT \| [regulation of locomotion](http://www.ebi.ac.uk/QuickGO/GTerm?id=GO:0040012) \| 27 \| 2.1 \| 3.3E-3 \| 1.8 \| 5.0E-2 \| \| GOTERM_BP_FAT \| [regulation of production of molecular mediator of immune response](http://www.ebi.ac.uk/QuickGO/GTerm?id=GO:0002700) \| 10 \| 0.8 \| 3.4E-3 \| 3.2 \| 5.1E-2 \| \| GOTERM_BP_FAT \| [hemostasis](http://www.ebi.ac.uk/QuickGO/GTerm?id=GO:0007599) \| 18 \| 1.4 \| 3.5E-3 \| 2.2 \| 5.3E-2 \| \| GOTERM_BP_FAT \| [positive regulation of lymphocyte mediated immunity](http://www.ebi.ac.uk/QuickGO/GTerm?id=GO:0002708) \| 9 \| 0.7 \| 3.6E-3 \| 3.4 \| 5.3E-2 \| \| GOTERM_BP_FAT \| [positive regulation of leukocyte mediated immunity](http://www.ebi.ac.uk/QuickGO/GTerm?id=GO:0002705) \| 9 \| 0.7 \| 3.6E-3 \| 3.4 \| 5.3E-2 \| \| GOTERM_BP_FAT \| [negative regulation of multicellular organismal process](http://www.ebi.ac.uk/QuickGO/GTerm?id=GO:0051241) \| 24 \| 1.8 \| 3.6E-3 \| 1.9 \| 5.3E-2 \| \| GOTERM_BP_FAT \| [regulation of JNK cascade](http://www.ebi.ac.uk/QuickGO/GTerm?id=GO:0046328) \| 13 \| 1.0 \| 3.6E-3 \| 2.6 \| 5.3E-2 \| \| GOTERM_BP_FAT \| [T cell homeostasis](http://www.ebi.ac.uk/QuickGO/GTerm?id=GO:0043029) \| 7 \| 0.5 \| 4.0E-3 \| 4.3 \| 6.0E-2 \| \| GOTERM_BP_FAT \| [cytolysis](http://www.ebi.ac.uk/QuickGO/GTerm?id=GO:0019835) \| 7 \| 0.5 \| 4.0E-3 \| 4.3 \| 6.0E-2 \| \| GOTERM_BP_FAT \| [positive regulation of stress-activated protein kinase signaling pathway](http://www.ebi.ac.uk/QuickGO/GTerm?id=GO:0070304) \| 7 \| 0.5 \| 4.0E-3 \| 4.3 \| 6.0E-2 \| \| GOTERM_BP_FAT \| [regulation of viral reproduction](http://www.ebi.ac.uk/QuickGO/GTerm?id=GO:0050792) \| 7 \| 0.5 \| 4.0E-3 \| 4.3 \| 6.0E-2 \| \| GOTERM_BP_FAT \| [patterning of blood vessels](http://www.ebi.ac.uk/QuickGO/GTerm?id=GO:0001569) \| 7 \| 0.5 \| 4.0E-3 \| 4.3 \| 6.0E-2 \| \| GOTERM_BP_FAT \| [positive regulation of organelle organization](http://www.ebi.ac.uk/QuickGO/GTerm?id=GO:0010638) \| 15 \| 1.1 \| 4.1E-3 \| 2.3 \| 6.0E-2 \| \| GOTERM_BP_FAT \| [positive regulation of T cell mediated immunity](http://www.ebi.ac.uk/QuickGO/GTerm?id=GO:0002711) \| 6 \| 0.5 \| 4.2E-3 \| 5.2 \| 6.1E-2 \| \| GOTERM_BP_FAT \| [DNA damage response. signal transduction by p53 class mediator resulting in induction of apoptosis](http://www.ebi.ac.uk/QuickGO/GTerm?id=GO:0042771) \| 6 \| 0.5 \| 4.2E-3 \| 5.2 \| 6.1E-2 \| \| GOTERM_BP_FAT \| [lymphocyte homeostasis](http://www.ebi.ac.uk/QuickGO/GTerm?id=GO:0002260) \| 8 \| 0.6 \| 4.4E-3 \| 3.7 \| 6.4E-2 \| \| GOTERM_BP_FAT \| [negative regulation of cell differentiation](http://www.ebi.ac.uk/QuickGO/GTerm?id=GO:0045596) \| 29 \| 2.2 \| 4.4E-3 \| 1.7 \| 6.4E-2 \| \| GOTERM_BP_FAT \| [response to UV](http://www.ebi.ac.uk/QuickGO/GTerm?id=GO:0009411) \| 12 \| 0.9 \| 4.8E-3 \| 2.6 \| 6.9E-2 \| \| GOTERM_BP_FAT \| [negative regulation of cell communication](http://www.ebi.ac.uk/QuickGO/GTerm?id=GO:0010648) \| 32 \| 2.5 \| 4.9E-3 \| 1.7 \| 7.0E-2 \| \| GOTERM_BP_FAT \| [regulation of T cell receptor signaling pathway](http://www.ebi.ac.uk/QuickGO/GTerm?id=GO:0050856) \| 5 \| 0.4 \| 5.0E-3 \| 6.5 \| 7.1E-2 \| \| GOTERM_BP_FAT \| [regulation of T-helper cell differentiation](http://www.ebi.ac.uk/QuickGO/GTerm?id=GO:0045622) \| 5 \| 0.4 \| 5.0E-3 \| 6.5 \| 7.1E-2 \| \| GOTERM_BP_FAT \| [T-helper 1 type immune response](http://www.ebi.ac.uk/QuickGO/GTerm?id=GO:0042088) \| 5 \| 0.4 \| 5.0E-3 \| 6.5 \| 7.1E-2 \| \| GOTERM_BP_FAT \| [induction of apoptosis by extracellular signals](http://www.ebi.ac.uk/QuickGO/GTerm?id=GO:0008624) \| 18 \| 1.4 \| 5.1E-3 \| 2.1 \| 7.3E-2 \| \| GOTERM_BP_FAT \| [collagen fibril organization](http://www.ebi.ac.uk/QuickGO/GTerm?id=GO:0030199) \| 8 \| 0.6 \| 5.4E-3 \| 3.6 \| 7.6E-2 \| \| GOTERM_BP_FAT \| [nucleus organization](http://www.ebi.ac.uk/QuickGO/GTerm?id=GO:0006997) \| 11 \| 0.8 \| 5.6E-3 \| 2.7 \| 7.9E-2 \| \| GOTERM_BP_FAT \| [regulation of cellular protein metabolic process](http://www.ebi.ac.uk/QuickGO/GTerm?id=GO:0032268) \| 53 \| 4.1 \| 5.7E-3 \| 1.5 \| 7.9E-2 \| \| GOTERM_BP_FAT \| [negative regulation of signal transduction](http://www.ebi.ac.uk/QuickGO/GTerm?id=GO:0009968) \| 29 \| 2.2 \| 6.1E-3 \| 1.7 \| 8.4E-2 \| \| GOTERM_BP_FAT \| [regulation of cell cycle process](http://www.ebi.ac.uk/QuickGO/GTerm?id=GO:0010564) \| 18 \| 1.4 \| 6.2E-3 \| 2.1 \| 8.5E-2 \| \| GOTERM_BP_FAT \| [regulation of DNA metabolic process](http://www.ebi.ac.uk/QuickGO/GTerm?id=GO:0051052) \| 18 \| 1.4 \| 6.2E-3 \| 2.1 \| 8.5E-2 \| \| GOTERM_BP_FAT \| [regulation of small GTPase mediated signal transduction](http://www.ebi.ac.uk/QuickGO/GTerm?id=GO:0051056) \| 32 \| 2.5 \| 6.2E-3 \| 1.7 \| 8.5E-2 \| \| GOTERM_BP_FAT \| [negative regulation of protein kinase activity](http://www.ebi.ac.uk/QuickGO/GTerm?id=GO:0006469) \| 15 \| 1.1 \| 6.3E-3 \| 2.2 \| 8.6E-2 \| \| GOTERM_BP_FAT \| [negative regulation of phosphorylation](http://www.ebi.ac.uk/QuickGO/GTerm?id=GO:0042326) \| 10 \| 0.8 \| 6.5E-3 \| 2.9 \| 8.8E-2 \| \| GOTERM_BP_FAT \| [positive regulation of cytoskeleton organization](http://www.ebi.ac.uk/QuickGO/GTerm?id=GO:0051495) \| 10 \| 0.8 \| 6.5E-3 \| 2.9 \| 8.8E-2 \| \| GOTERM_BP_FAT \| [response to gamma radiation](http://www.ebi.ac.uk/QuickGO/GTerm?id=GO:0010332) \| 7 \| 0.5 \| 6.6E-3 \| 4.0 \| 8.9E-2 \| \| GOTERM_BP_FAT \| [T cell receptor signaling pathway](http://www.ebi.ac.uk/QuickGO/GTerm?id=GO:0050852) \| 7 \| 0.5 \| 6.6E-3 \| 4.0 \| 8.9E-2 \| \| GOTERM_BP_FAT \| [positive regulation of inflammatory response](http://www.ebi.ac.uk/QuickGO/GTerm?id=GO:0050729) \| 8 \| 0.6 \| 6.6E-3 \| 3.5 \| 8.9E-2 \| \| GOTERM_BP_FAT \| [locomotory behavior](http://www.ebi.ac.uk/QuickGO/GTerm?id=GO:0007626) \| 34 \| 2.6 \| 6.7E-3 \| 1.6 \| 9.0E-2 \| \| GOTERM_BP_FAT \| [positive regulation of protein metabolic process](http://www.ebi.ac.uk/QuickGO/GTerm?id=GO:0051247) \| 31 \| 2.4 \| 6.7E-3 \| 1.7 \| 9.0E-2 \| \| GOTERM_BP_FAT \| [negative regulation of alpha-beta T cell activation](http://www.ebi.ac.uk/QuickGO/GTerm?id=GO:0046636) \| 5 \| 0.4 \| 7.4E-3 \| 5.9 \| 9.8E-2 \| \| GOTERM_BP_FAT \| [regulation of cholesterol storage](http://www.ebi.ac.uk/QuickGO/GTerm?id=GO:0010885) \| 5 \| 0.4 \| 7.4E-3 \| 5.9 \| 9.8E-2 \| \| GOTERM_BP_FAT \| [positive regulation of interferon-gamma production](http://www.ebi.ac.uk/QuickGO/GTerm?id=GO:0032729) \| 5 \| 0.4 \| 7.4E-3 \| 5.9 \| 9.8E-2 \| \| GOTERM_BP_FAT \| [homeostasis of number of cells within a tissue](http://www.ebi.ac.uk/QuickGO/GTerm?id=GO:0048873) \| 6 \| 0.5 \| 7.5E-3 \| 4.6 \| 1.0E-1 \| \| GOTERM_BP_FAT \| [positive regulation of JNK cascade](http://www.ebi.ac.uk/QuickGO/GTerm?id=GO:0046330) \| 6 \| 0.5 \| 7.5E-3 \| 4.6 \| 1.0E-1 \| \| GOTERM_BP_FAT \| [regulation of T-helper 2 cell differentiation](http://www.ebi.ac.uk/QuickGO/GTerm?id=GO:0045628) \| 4 \| 0.3 \| 7.6E-3 \| 8.7 \| 1.0E-1 \| \| GOTERM_BP_FAT \| [negative regulation of viral genome replication](http://www.ebi.ac.uk/QuickGO/GTerm?id=GO:0045071) \| 4 \| 0.3 \| 7.6E-3 \| 8.7 \| 1.0E-1 \| \| GOTERM_BP_FAT \| [positive regulation of T-helper cell differentiation](http://www.ebi.ac.uk/QuickGO/GTerm?id=GO:0045624) \| 4 \| 0.3 \| 7.6E-3 \| 8.7 \| 1.0E-1 \| \| GOTERM_BP_FAT \| [second-messenger-mediated signaling](http://www.ebi.ac.uk/QuickGO/GTerm?id=GO:0019932) \| 30 \| 2.3 \| 7.6E-3 \| 1.7 \| 1.0E-1 \| \| GOTERM_BP_FAT \| [positive regulation of cell migration](http://www.ebi.ac.uk/QuickGO/GTerm?id=GO:0030335) \| 15 \| 1.1 \| 7.7E-3 \| 2.2 \| 1.0E-1 \| \| GOTERM_BP_FAT \| [protein secretion](http://www.ebi.ac.uk/QuickGO/GTerm?id=GO:0009306) \| 8 \| 0.6 \| 8.0E-3 \| 3.4 \| 1.0E-1 \| \| GOTERM_BP_FAT \| [apoptotic mitochondrial changes](http://www.ebi.ac.uk/QuickGO/GTerm?id=GO:0008637) \| 8 \| 0.6 \| 8.0E-3 \| 3.4 \| 1.0E-1 \| \| GOTERM_BP_FAT \| [regulation of cytoskeleton organization](http://www.ebi.ac.uk/QuickGO/GTerm?id=GO:0051493) \| 20 \| 1.5 \| 8.0E-3 \| 1.9 \| 1.0E-1 \| \| GOTERM_BP_FAT \| [DNA packaging](http://www.ebi.ac.uk/QuickGO/GTerm?id=GO:0006323) \| 18 \| 1.4 \| 8.0E-3 \| 2.0 \| 1.0E-1 \| \| GOTERM_BP_FAT \| [regulation of B cell mediated immunity](http://www.ebi.ac.uk/QuickGO/GTerm?id=GO:0002712) \| 7 \| 0.5 \| 8.2E-3 \| 3.8 \| 1.1E-1 \| \| GOTERM_BP_FAT \| [regulation of immunoglobulin mediated immune response](http://www.ebi.ac.uk/QuickGO/GTerm?id=GO:0002889) \| 7 \| 0.5 \| 8.2E-3 \| 3.8 \| 1.1E-1 \| \| GOTERM_BP_FAT \| [phosphorylation](http://www.ebi.ac.uk/QuickGO/GTerm?id=GO:0016310) \| 81 \| 6.2 \| 8.4E-3 \| 1.3 \| 1.1E-1 \| \| GOTERM_BP_FAT \| [negative regulation of kinase activity](http://www.ebi.ac.uk/QuickGO/GTerm?id=GO:0033673) \| 15 \| 1.1 \| 8.5E-3 \| 2.2 \| 1.1E-1 \| \| GOTERM_BP_FAT \| [glycolysis](http://www.ebi.ac.uk/QuickGO/GTerm?id=GO:0006096) \| 10 \| 0.8 \| 8.6E-3 \| 2.8 \| 1.1E-1 \| \| GOTERM_BP_FAT \| [regulation of MAPKKK cascade](http://www.ebi.ac.uk/QuickGO/GTerm?id=GO:0043408) \| 17 \| 1.3 \| 9.0E-3 \| 2.0 \| 1.1E-1 \| \| GOTERM_BP_FAT \| [positive regulation of response to external stimulus](http://www.ebi.ac.uk/QuickGO/GTerm?id=GO:0032103) \| 12 \| 0.9 \| 9.0E-3 \| 2.4 \| 1.1E-1 \| \| GOTERM_BP_FAT \| [negative regulation of B cell activation](http://www.ebi.ac.uk/QuickGO/GTerm?id=GO:0050869) \| 6 \| 0.5 \| 9.8E-3 \| 4.3 \| 1.2E-1 \| \| GOTERM_BP_FAT \| [detection of biotic stimulus](http://www.ebi.ac.uk/QuickGO/GTerm?id=GO:0009595) \| 6 \| 0.5 \| 9.8E-3 \| 4.3 \| 1.2E-1 \| \| GOTERM_BP_FAT \| [negative regulation of phosphorus metabolic process](http://www.ebi.ac.uk/QuickGO/GTerm?id=GO:0010563) \| 10 \| 0.8 \| 9.9E-3 \| 2.7 \| 1.2E-1 \| \| GOTERM_BP_FAT \| [negative regulation of phosphate metabolic process](http://www.ebi.ac.uk/QuickGO/GTerm?id=GO:0045936) \| 10 \| 0.8 \| 9.9E-3 \| 2.7 \| 1.2E-1 \| \| GOTERM_BP_FAT \| [regulation of endothelial cell migration](http://www.ebi.ac.uk/QuickGO/GTerm?id=GO:0010594) \| 7 \| 0.5 \| 1.0E-2 \| 3.6 \| 1.3E-1 \| \| GOTERM_BP_FAT \| [positive regulation of interleukin-1 beta secretion](http://www.ebi.ac.uk/QuickGO/GTerm?id=GO:0050718) \| 5 \| 0.4 \| 1.0E-2 \| 5.4 \| 1.3E-1 \| \| GOTERM_BP_FAT \| [leukocyte migration](http://www.ebi.ac.uk/QuickGO/GTerm?id=GO:0050900) \| 11 \| 0.8 \| 1.1E-2 \| 2.5 \| 1.3E-1 \| \| GOTERM_BP_FAT \| [coagulation](http://www.ebi.ac.uk/QuickGO/GTerm?id=GO:0050817) \| 16 \| 1.2 \| 1.1E-2 \| 2.0 \| 1.3E-1 \| \| GOTERM_BP_FAT \| [positive regulation of MAP kinase activity](http://www.ebi.ac.uk/QuickGO/GTerm?id=GO:0043406) \| 16 \| 1.2 \| 1.1E-2 \| 2.0 \| 1.3E-1 \| \| GOTERM_BP_FAT \| [blood coagulation](http://www.ebi.ac.uk/QuickGO/GTerm?id=GO:0007596) \| 16 \| 1.2 \| 1.1E-2 \| 2.0 \| 1.3E-1 \| \| GOTERM_BP_FAT \| [DNA damage response. signal transduction resulting in induction of apoptosis](http://www.ebi.ac.uk/QuickGO/GTerm?id=GO:0008630) \| 8 \| 0.6 \| 1.1E-2 \| 3.2 \| 1.4E-1 \| \| GOTERM_BP_FAT \| [positive regulation of macromolecule metabolic process](http://www.ebi.ac.uk/QuickGO/GTerm?id=GO:0010604) \| 85 \| 6.5 \| 1.2E-2 \| 1.3 \| 1.4E-1 \| \| GOTERM_BP_FAT \| [wound healing](http://www.ebi.ac.uk/QuickGO/GTerm?id=GO:0042060) \| 25 \| 1.9 \| 1.2E-2 \| 1.7 \| 1.4E-1 \| \| GOTERM_BP_FAT \| [positive regulation of NF-kappaB transcription factor activity](http://www.ebi.ac.uk/QuickGO/GTerm?id=GO:0051092) \| 9 \| 0.7 \| 1.2E-2 \| 2.9 \| 1.4E-1 \| \| GOTERM_BP_FAT \| [hexose metabolic process](http://www.ebi.ac.uk/QuickGO/GTerm?id=GO:0019318) \| 25 \| 1.9 \| 1.2E-2 \| 1.7 \| 1.5E-1 \| \| GOTERM_BP_FAT \| [DNA damage response. signal transduction by p53 class mediator](http://www.ebi.ac.uk/QuickGO/GTerm?id=GO:0030330) \| 7 \| 0.5 \| 1.2E-2 \| 3.5 \| 1.5E-1 \| \| GOTERM_BP_FAT \| [regulation of immunoglobulin production](http://www.ebi.ac.uk/QuickGO/GTerm?id=GO:0002637) \| 7 \| 0.5 \| 1.2E-2 \| 3.5 \| 1.5E-1 \| \| GOTERM_BP_FAT \| [apoptotic nuclear changes](http://www.ebi.ac.uk/QuickGO/GTerm?id=GO:0030262) \| 7 \| 0.5 \| 1.2E-2 \| 3.5 \| 1.5E-1 \| \| GOTERM_BP_FAT \| [regulation of DNA recombination](http://www.ebi.ac.uk/QuickGO/GTerm?id=GO:0000018) \| 7 \| 0.5 \| 1.2E-2 \| 3.5 \| 1.5E-1 \| \| GOTERM_BP_FAT \| [organ regeneration](http://www.ebi.ac.uk/QuickGO/GTerm?id=GO:0031100) \| 7 \| 0.5 \| 1.2E-2 \| 3.5 \| 1.5E-1 \| \| GOTERM_BP_FAT \| [positive regulation of interleukin-6 production](http://www.ebi.ac.uk/QuickGO/GTerm?id=GO:0032755) \| 6 \| 0.5 \| 1.2E-2 \| 4.1 \| 1.5E-1 \| \| GOTERM_BP_FAT \| [cholesterol efflux](http://www.ebi.ac.uk/QuickGO/GTerm?id=GO:0033344) \| 6 \| 0.5 \| 1.2E-2 \| 4.1 \| 1.5E-1 \| \| GOTERM_BP_FAT \| [negative regulation of viral reproduction](http://www.ebi.ac.uk/QuickGO/GTerm?id=GO:0048525) \| 4 \| 0.3 \| 1.3E-2 \| 7.4 \| 1.5E-1 \| \| GOTERM_BP_FAT \| [regulation of granulocyte differentiation](http://www.ebi.ac.uk/QuickGO/GTerm?id=GO:0030852) \| 4 \| 0.3 \| 1.3E-2 \| 7.4 \| 1.5E-1 \| \| GOTERM_BP_FAT \| [positive regulation of T cell mediated cytotoxicity](http://www.ebi.ac.uk/QuickGO/GTerm?id=GO:0001916) \| 4 \| 0.3 \| 1.3E-2 \| 7.4 \| 1.5E-1 \| \| GOTERM_BP_FAT \| [membrane raft organization](http://www.ebi.ac.uk/QuickGO/GTerm?id=GO:0031579) \| 4 \| 0.3 \| 1.3E-2 \| 7.4 \| 1.5E-1 \| \| GOTERM_BP_FAT \| [regulation of T-helper 1 type immune response](http://www.ebi.ac.uk/QuickGO/GTerm?id=GO:0002825) \| 4 \| 0.3 \| 1.3E-2 \| 7.4 \| 1.5E-1 \| \| GOTERM_BP_FAT \| [lymphocyte chemotaxis](http://www.ebi.ac.uk/QuickGO/GTerm?id=GO:0048247) \| 4 \| 0.3 \| 1.3E-2 \| 7.4 \| 1.5E-1 \| \| GOTERM_BP_FAT \| [cell adhesion mediated by integrin](http://www.ebi.ac.uk/QuickGO/GTerm?id=GO:0033627) \| 4 \| 0.3 \| 1.3E-2 \| 7.4 \| 1.5E-1 \| \| GOTERM_BP_FAT \| [positive regulation of interleukin-8 biosynthetic process](http://www.ebi.ac.uk/QuickGO/GTerm?id=GO:0045416) \| 4 \| 0.3 \| 1.3E-2 \| 7.4 \| 1.5E-1 \| \| GOTERM_BP_FAT \| [extracellular matrix organization](http://www.ebi.ac.uk/QuickGO/GTerm?id=GO:0030198) \| 16 \| 1.2 \| 1.3E-2 \| 2.0 \| 1.5E-1 \| \| GOTERM_BP_FAT \| [ER to Golgi vesicle-mediated transport](http://www.ebi.ac.uk/QuickGO/GTerm?id=GO:0006888) \| 9 \| 0.7 \| 1.3E-2 \| 2.8 \| 1.6E-1 \| \| GOTERM_BP_FAT \| [glucose metabolic process](http://www.ebi.ac.uk/QuickGO/GTerm?id=GO:0006006) \| 21 \| 1.6 \| 1.3E-2 \| 1.8 \| 1.6E-1 \| \| GOTERM_BP_FAT \| [positive regulation of peptidase activity](http://www.ebi.ac.uk/QuickGO/GTerm?id=GO:0010952) \| 11 \| 0.8 \| 1.4E-2 \| 2.4 \| 1.6E-1 \| \| GOTERM_BP_FAT \| [positive regulation of caspase activity](http://www.ebi.ac.uk/QuickGO/GTerm?id=GO:0043280) \| 11 \| 0.8 \| 1.4E-2 \| 2.4 \| 1.6E-1 \| \| GOTERM_BP_FAT \| [regulation of peptidase activity](http://www.ebi.ac.uk/QuickGO/GTerm?id=GO:0052547) \| 14 \| 1.1 \| 1.4E-2 \| 2.1 \| 1.6E-1 \| \| GOTERM_BP_FAT \| [response to lipopolysaccharide](http://www.ebi.ac.uk/QuickGO/GTerm?id=GO:0032496) \| 13 \| 1.0 \| 1.4E-2 \| 2.2 \| 1.6E-1 \| \| GOTERM_BP_FAT \| [MAPKKK cascade](http://www.ebi.ac.uk/QuickGO/GTerm?id=GO:0000165) \| 24 \| 1.8 \| 1.4E-2 \| 1.7 \| 1.6E-1 \| \| GOTERM_BP_FAT \| [positive regulation of endothelial cell migration](http://www.ebi.ac.uk/QuickGO/GTerm?id=GO:0010595) \| 5 \| 0.4 \| 1.4E-2 \| 5.0 \| 1.6E-1 \| \| GOTERM_BP_FAT \| [regulation of isotype switching](http://www.ebi.ac.uk/QuickGO/GTerm?id=GO:0045191) \| 5 \| 0.4 \| 1.4E-2 \| 5.0 \| 1.6E-1 \| \| GOTERM_BP_FAT \| [regulation of interleukin-1 beta secretion](http://www.ebi.ac.uk/QuickGO/GTerm?id=GO:0050706) \| 5 \| 0.4 \| 1.4E-2 \| 5.0 \| 1.6E-1 \| \| GOTERM_BP_FAT \| [negative regulation of transferase activity](http://www.ebi.ac.uk/QuickGO/GTerm?id=GO:0051348) \| 15 \| 1.1 \| 1.5E-2 \| 2.0 \| 1.7E-1 \| \| GOTERM_BP_FAT \| [response to drug](http://www.ebi.ac.uk/QuickGO/GTerm?id=GO:0042493) \| 27 \| 2.1 \| 1.5E-2 \| 1.6 \| 1.7E-1 \| \| GOTERM_BP_FAT \| [virus-host interaction](http://www.ebi.ac.uk/QuickGO/GTerm?id=GO:0019048) \| 6 \| 0.5 \| 1.6E-2 \| 3.9 \| 1.7E-1 \| \| GOTERM_BP_FAT \| [defense response to Gram-positive bacterium](http://www.ebi.ac.uk/QuickGO/GTerm?id=GO:0050830) \| 6 \| 0.5 \| 1.6E-2 \| 3.9 \| 1.7E-1 \| \| GOTERM_BP_FAT \| [positive regulation of B cell proliferation](http://www.ebi.ac.uk/QuickGO/GTerm?id=GO:0030890) \| 6 \| 0.5 \| 1.6E-2 \| 3.9 \| 1.7E-1 \| \| GOTERM_BP_FAT \| [positive regulation of phosphorylation](http://www.ebi.ac.uk/QuickGO/GTerm?id=GO:0042327) \| 15 \| 1.1 \| 1.6E-2 \| 2.0 \| 1.8E-1 \| \| GOTERM_BP_FAT \| [cellular macromolecular complex subunit organization](http://www.ebi.ac.uk/QuickGO/GTerm?id=GO:0034621) \| 40 \| 3.1 \| 1.6E-2 \| 1.5 \| 1.8E-1 \| \| GOTERM_BP_FAT \| [regulation of interleukin-1 alpha secretion](http://www.ebi.ac.uk/QuickGO/GTerm?id=GO:0050705) \| 3 \| 0.2 \| 1.7E-2 \| 13.0 \| 1.8E-1 \| \| GOTERM_BP_FAT \| [positive regulation of interleukin-1 alpha secretion](http://www.ebi.ac.uk/QuickGO/GTerm?id=GO:0050717) \| 3 \| 0.2 \| 1.7E-2 \| 13.0 \| 1.8E-1 \| \| GOTERM_BP_FAT \| [detection of lipopolysaccharide](http://www.ebi.ac.uk/QuickGO/GTerm?id=GO:0032497) \| 3 \| 0.2 \| 1.7E-2 \| 13.0 \| 1.8E-1 \| \| GOTERM_BP_FAT \| [humoral immune response](http://www.ebi.ac.uk/QuickGO/GTerm?id=GO:0006959) \| 13 \| 1.0 \| 1.7E-2 \| 2.1 \| 1.9E-1 \| \| GOTERM_BP_FAT \| [positive regulation of locomotion](http://www.ebi.ac.uk/QuickGO/GTerm?id=GO:0040017) \| 15 \| 1.1 \| 1.7E-2 \| 2.0 \| 1.9E-1 \| \| GOTERM_BP_FAT \| [regulation of interleukin-6 production](http://www.ebi.ac.uk/QuickGO/GTerm?id=GO:0032675) \| 8 \| 0.6 \| 1.8E-2 \| 2.9 \| 2.0E-1 \| \| GOTERM_BP_FAT \| [leukocyte homeostasis](http://www.ebi.ac.uk/QuickGO/GTerm?id=GO:0001776) \| 8 \| 0.6 \| 1.8E-2 \| 2.9 \| 2.0E-1 \| \| GOTERM_BP_FAT \| [positive regulation of protein amino acid phosphorylation](http://www.ebi.ac.uk/QuickGO/GTerm?id=GO:0001934) \| 14 \| 1.1 \| 1.8E-2 \| 2.0 \| 1.9E-1 \| \| GOTERM_BP_FAT \| [cytosolic calcium ion homeostasis](http://www.ebi.ac.uk/QuickGO/GTerm?id=GO:0051480) \| 17 \| 1.3 \| 1.8E-2 \| 1.9 \| 2.0E-1 \| \| GOTERM_BP_FAT \| [DNA repair](http://www.ebi.ac.uk/QuickGO/GTerm?id=GO:0006281) \| 33 \| 2.5 \| 1.9E-2 \| 1.5 \| 2.0E-1 \| \| GOTERM_BP_FAT \| [positive regulation of interleukin-1 beta production](http://www.ebi.ac.uk/QuickGO/GTerm?id=GO:0032731) \| 5 \| 0.4 \| 1.9E-2 \| 4.6 \| 2.0E-1 \| \| GOTERM_BP_FAT \| [regulation of viral genome replication](http://www.ebi.ac.uk/QuickGO/GTerm?id=GO:0045069) \| 5 \| 0.4 \| 1.9E-2 \| 4.6 \| 2.0E-1 \| \| GOTERM_BP_FAT \| [positive regulation of interleukin-1 secretion](http://www.ebi.ac.uk/QuickGO/GTerm?id=GO:0050716) \| 5 \| 0.4 \| 1.9E-2 \| 4.6 \| 2.0E-1 \| \| GOTERM_BP_FAT \| [mast cell activation](http://www.ebi.ac.uk/QuickGO/GTerm?id=GO:0045576) \| 4 \| 0.3 \| 1.9E-2 \| 6.5 \| 2.0E-1 \| \| GOTERM_BP_FAT \| [Rac protein signal transduction](http://www.ebi.ac.uk/QuickGO/GTerm?id=GO:0016601) \| 4 \| 0.3 \| 1.9E-2 \| 6.5 \| 2.0E-1 \| \| GOTERM_BP_FAT \| [lymphocyte costimulation](http://www.ebi.ac.uk/QuickGO/GTerm?id=GO:0031294) \| 4 \| 0.3 \| 1.9E-2 \| 6.5 \| 2.0E-1 \| \| GOTERM_BP_FAT \| [negative regulation of B cell proliferation](http://www.ebi.ac.uk/QuickGO/GTerm?id=GO:0030889) \| 4 \| 0.3 \| 1.9E-2 \| 6.5 \| 2.0E-1 \| \| GOTERM_BP_FAT \| [T cell costimulation](http://www.ebi.ac.uk/QuickGO/GTerm?id=GO:0031295) \| 4 \| 0.3 \| 1.9E-2 \| 6.5 \| 2.0E-1 \| \| GOTERM_BP_FAT \| [regulation of survival gene product expression](http://www.ebi.ac.uk/QuickGO/GTerm?id=GO:0045884) \| 6 \| 0.5 \| 1.9E-2 \| 3.7 \| 2.0E-1 \| \| GOTERM_BP_FAT \| [regulation of lipid storage](http://www.ebi.ac.uk/QuickGO/GTerm?id=GO:0010883) \| 6 \| 0.5 \| 1.9E-2 \| 3.7 \| 2.0E-1 \| \| GOTERM_BP_FAT \| [natural killer cell activation](http://www.ebi.ac.uk/QuickGO/GTerm?id=GO:0030101) \| 6 \| 0.5 \| 1.9E-2 \| 3.7 \| 2.0E-1 \| \| GOTERM_BP_FAT \| [regulation of T cell mediated immunity](http://www.ebi.ac.uk/QuickGO/GTerm?id=GO:0002709) \| 6 \| 0.5 \| 1.9E-2 \| 3.7 \| 2.0E-1 \| \| GOTERM_BP_FAT \| [positive regulation of hydrolase activity](http://www.ebi.ac.uk/QuickGO/GTerm?id=GO:0051345) \| 23 \| 1.8 \| 1.9E-2 \| 1.7 \| 2.0E-1 \| \| GOTERM_BP_FAT \| [vesicle-mediated transport](http://www.ebi.ac.uk/QuickGO/GTerm?id=GO:0016192) \| 59 \| 4.5 \| 2.0E-2 \| 1.3 \| 2.1E-1 \| \| GOTERM_BP_FAT \| [cellular macromolecular complex assembly](http://www.ebi.ac.uk/QuickGO/GTerm?id=GO:0034622) \| 36 \| 2.8 \| 2.0E-2 \| 1.5 \| 2.1E-1 \| \| GOTERM_BP_FAT \| [positive regulation of phosphorus metabolic process](http://www.ebi.ac.uk/QuickGO/GTerm?id=GO:0010562) \| 15 \| 1.1 \| 2.1E-2 \| 1.9 \| 2.1E-1 \| \| GOTERM_BP_FAT \| [positive regulation of phosphate metabolic process](http://www.ebi.ac.uk/QuickGO/GTerm?id=GO:0045937) \| 15 \| 1.1 \| 2.1E-2 \| 1.9 \| 2.1E-1 \| \| GOTERM_BP_FAT \| [cholesterol transport](http://www.ebi.ac.uk/QuickGO/GTerm?id=GO:0030301) \| 8 \| 0.6 \| 2.1E-2 \| 2.8 \| 2.2E-1 \| \| GOTERM_BP_FAT \| [sterol transport](http://www.ebi.ac.uk/QuickGO/GTerm?id=GO:0015918) \| 8 \| 0.6 \| 2.1E-2 \| 2.8 \| 2.2E-1 \| \| GOTERM_BP_FAT \| [leukocyte chemotaxis](http://www.ebi.ac.uk/QuickGO/GTerm?id=GO:0030595) \| 8 \| 0.6 \| 2.1E-2 \| 2.8 \| 2.2E-1 \| \| GOTERM_BP_FAT \| [activation of caspase activity](http://www.ebi.ac.uk/QuickGO/GTerm?id=GO:0006919) \| 10 \| 0.8 \| 2.1E-2 \| 2.4 \| 2.1E-1 \| \| GOTERM_BP_FAT \| [regulation of cell shape](http://www.ebi.ac.uk/QuickGO/GTerm?id=GO:0008360) \| 10 \| 0.8 \| 2.1E-2 \| 2.4 \| 2.1E-1 \| \| GOTERM_BP_FAT \| [regulation of calcium ion transport into cytosol](http://www.ebi.ac.uk/QuickGO/GTerm?id=GO:0010522) \| 7 \| 0.5 \| 2.1E-2 \| 3.1 \| 2.1E-1 \| \| GOTERM_BP_FAT \| [actin filament organization](http://www.ebi.ac.uk/QuickGO/GTerm?id=GO:0007015) \| 12 \| 0.9 \| 2.1E-2 \| 2.2 \| 2.1E-1 \| \| GOTERM_BP_FAT \| [positive regulation of cellular protein metabolic process](http://www.ebi.ac.uk/QuickGO/GTerm?id=GO:0032270) \| 28 \| 2.1 \| 2.1E-2 \| 1.6 \| 2.1E-1 \| \| GOTERM_BP_FAT \| [regulation of endopeptidase activity](http://www.ebi.ac.uk/QuickGO/GTerm?id=GO:0052548) \| 13 \| 1.0 \| 2.2E-2 \| 2.1 \| 2.3E-1 \| \| GOTERM_BP_FAT \| [regulation of interleukin-8 production](http://www.ebi.ac.uk/QuickGO/GTerm?id=GO:0032677) \| 5 \| 0.4 \| 2.4E-2 \| 4.3 \| 2.4E-1 \| \| GOTERM_BP_FAT \| [response to X-ray](http://www.ebi.ac.uk/QuickGO/GTerm?id=GO:0010165) \| 5 \| 0.4 \| 2.4E-2 \| 4.3 \| 2.4E-1 \| \| GOTERM_BP_FAT \| [regulation of interleukin-1 secretion](http://www.ebi.ac.uk/QuickGO/GTerm?id=GO:0050704) \| 5 \| 0.4 \| 2.4E-2 \| 4.3 \| 2.4E-1 \| \| GOTERM_BP_FAT \| [sterol homeostasis](http://www.ebi.ac.uk/QuickGO/GTerm?id=GO:0055092) \| 8 \| 0.6 \| 2.4E-2 \| 2.7 \| 2.4E-1 \| \| GOTERM_BP_FAT \| [negative regulation of cytokine production](http://www.ebi.ac.uk/QuickGO/GTerm?id=GO:0001818) \| 8 \| 0.6 \| 2.4E-2 \| 2.7 \| 2.4E-1 \| \| GOTERM_BP_FAT \| [cholesterol homeostasis](http://www.ebi.ac.uk/QuickGO/GTerm?id=GO:0042632) \| 8 \| 0.6 \| 2.4E-2 \| 2.7 \| 2.4E-1 \| \| GOTERM_BP_FAT \| [negative regulation of myeloid cell differentiation](http://www.ebi.ac.uk/QuickGO/GTerm?id=GO:0045638) \| 7 \| 0.5 \| 2.4E-2 \| 3.0 \| 2.4E-1 \| \| GOTERM_BP_FAT \| [tissue remodeling](http://www.ebi.ac.uk/QuickGO/GTerm?id=GO:0048771) \| 10 \| 0.8 \| 2.6E-2 \| 2.3 \| 2.5E-1 \| \| GOTERM_BP_FAT \| [B cell receptor signaling pathway](http://www.ebi.ac.uk/QuickGO/GTerm?id=GO:0050853) \| 4 \| 0.3 \| 2.7E-2 \| 5.8 \| 2.6E-1 \| \| GOTERM_BP_FAT \| [regulation of T cell mediated cytotoxicity](http://www.ebi.ac.uk/QuickGO/GTerm?id=GO:0001914) \| 4 \| 0.3 \| 2.7E-2 \| 5.8 \| 2.6E-1 \| \| GOTERM_BP_FAT \| [positive regulation of lipid storage](http://www.ebi.ac.uk/QuickGO/GTerm?id=GO:0010884) \| 4 \| 0.3 \| 2.7E-2 \| 5.8 \| 2.6E-1 \| \| GOTERM_BP_FAT \| [regulation of defense response to virus by host](http://www.ebi.ac.uk/QuickGO/GTerm?id=GO:0050691) \| 4 \| 0.3 \| 2.7E-2 \| 5.8 \| 2.6E-1 \| \| GOTERM_BP_FAT \| [regulation of interleukin-8 biosynthetic process](http://www.ebi.ac.uk/QuickGO/GTerm?id=GO:0045414) \| 4 \| 0.3 \| 2.7E-2 \| 5.8 \| 2.6E-1 \| \| GOTERM_BP_FAT \| [phosphorus metabolic process](http://www.ebi.ac.uk/QuickGO/GTerm?id=GO:0006793) \| 92 \| 7.0 \| 2.7E-2 \| 1.2 \| 2.6E-1 \| \| GOTERM_BP_FAT \| [phosphate metabolic process](http://www.ebi.ac.uk/QuickGO/GTerm?id=GO:0006796) \| 92 \| 7.0 \| 2.7E-2 \| 1.2 \| 2.6E-1 \| \| GOTERM_BP_FAT \| [cell chemotaxis](http://www.ebi.ac.uk/QuickGO/GTerm?id=GO:0060326) \| 8 \| 0.6 \| 2.7E-2 \| 2.7 \| 2.6E-1 \| \| GOTERM_BP_FAT \| [cell structure disassembly during apoptosis](http://www.ebi.ac.uk/QuickGO/GTerm?id=GO:0006921) \| 6 \| 0.5 \| 2.8E-2 \| 3.4 \| 2.7E-1 \| \| GOTERM_BP_FAT \| [positive regulation of smooth muscle cell proliferation](http://www.ebi.ac.uk/QuickGO/GTerm?id=GO:0048661) \| 7 \| 0.5 \| 2.8E-2 \| 2.9 \| 2.7E-1 \| \| GOTERM_BP_FAT \| [regulation of tumor necrosis factor production](http://www.ebi.ac.uk/QuickGO/GTerm?id=GO:0032680) \| 7 \| 0.5 \| 2.8E-2 \| 2.9 \| 2.7E-1 \| \| GOTERM_BP_FAT \| [negative regulation of gene-specific transcription](http://www.ebi.ac.uk/QuickGO/GTerm?id=GO:0032582) \| 9 \| 0.7 \| 2.9E-2 \| 2.4 \| 2.7E-1 \| \| GOTERM_BP_FAT \| [positive regulation of protein modification process](http://www.ebi.ac.uk/QuickGO/GTerm?id=GO:0031401) \| 23 \| 1.8 \| 3.0E-2 \| 1.6 \| 2.8E-1 \| \| GOTERM_BP_FAT \| [regulation of B cell differentiation](http://www.ebi.ac.uk/QuickGO/GTerm?id=GO:0045577) \| 5 \| 0.4 \| 3.0E-2 \| 4.1 \| 2.8E-1 \| \| GOTERM_BP_FAT \| [regulation of blood vessel endothelial cell migration](http://www.ebi.ac.uk/QuickGO/GTerm?id=GO:0043535) \| 5 \| 0.4 \| 3.0E-2 \| 4.1 \| 2.8E-1 \| \| GOTERM_BP_FAT \| [lipoprotein particle clearance](http://www.ebi.ac.uk/QuickGO/GTerm?id=GO:0034381) \| 5 \| 0.4 \| 3.0E-2 \| 4.1 \| 2.8E-1 \| \| GOTERM_BP_FAT \| ['de novo' protein folding](http://www.ebi.ac.uk/QuickGO/GTerm?id=GO:0006458) \| 5 \| 0.4 \| 3.0E-2 \| 4.1 \| 2.8E-1 \| \| GOTERM_BP_FAT \| [glucose catabolic process](http://www.ebi.ac.uk/QuickGO/GTerm?id=GO:0006007) \| 10 \| 0.8 \| 3.2E-2 \| 2.2 \| 3.0E-1 \| \| GOTERM_BP_FAT \| [positive regulation of inflammatory response to antigenic stimulus](http://www.ebi.ac.uk/QuickGO/GTerm?id=GO:0002863) \| 3 \| 0.2 \| 3.2E-2 \| 9.7 \| 3.0E-1 \| \| GOTERM_BP_FAT \| [positive regulation of interferon-alpha biosynthetic process](http://www.ebi.ac.uk/QuickGO/GTerm?id=GO:0045356) \| 3 \| 0.2 \| 3.2E-2 \| 9.7 \| 3.0E-1 \| \| GOTERM_BP_FAT \| [regulation of interleukin-1 alpha production](http://www.ebi.ac.uk/QuickGO/GTerm?id=GO:0032650) \| 3 \| 0.2 \| 3.2E-2 \| 9.7 \| 3.0E-1 \| \| GOTERM_BP_FAT \| [positive regulation of interleukin-1 alpha production](http://www.ebi.ac.uk/QuickGO/GTerm?id=GO:0032730) \| 3 \| 0.2 \| 3.2E-2 \| 9.7 \| 3.0E-1 \| \| GOTERM_BP_FAT \| [regulation of endothelial cell differentiation](http://www.ebi.ac.uk/QuickGO/GTerm?id=GO:0045601) \| 3 \| 0.2 \| 3.2E-2 \| 9.7 \| 3.0E-1 \| \| GOTERM_BP_FAT \| [regulation of retroviral genome replication](http://www.ebi.ac.uk/QuickGO/GTerm?id=GO:0045091) \| 3 \| 0.2 \| 3.2E-2 \| 9.7 \| 3.0E-1 \| \| GOTERM_BP_FAT \| [response to lipoteichoic acid](http://www.ebi.ac.uk/QuickGO/GTerm?id=GO:0070391) \| 3 \| 0.2 \| 3.2E-2 \| 9.7 \| 3.0E-1 \| \| GOTERM_BP_FAT \| [positive regulation of B cell apoptosis](http://www.ebi.ac.uk/QuickGO/GTerm?id=GO:0002904) \| 3 \| 0.2 \| 3.2E-2 \| 9.7 \| 3.0E-1 \| \| GOTERM_BP_FAT \| [regulation of microtubule-based process](http://www.ebi.ac.uk/QuickGO/GTerm?id=GO:0032886) \| 9 \| 0.7 \| 3.2E-2 \| 2.4 \| 3.0E-1 \| \| GOTERM_BP_FAT \| [erythrocyte homeostasis](http://www.ebi.ac.uk/QuickGO/GTerm?id=GO:0034101) \| 9 \| 0.7 \| 3.2E-2 \| 2.4 \| 3.0E-1 \| \| GOTERM_BP_FAT \| [negative regulation of protein amino acid phosphorylation](http://www.ebi.ac.uk/QuickGO/GTerm?id=GO:0001933) \| 7 \| 0.5 \| 3.3E-2 \| 2.8 \| 3.0E-1 \| \| GOTERM_BP_FAT \| [detection of chemical stimulus](http://www.ebi.ac.uk/QuickGO/GTerm?id=GO:0009593) \| 7 \| 0.5 \| 3.3E-2 \| 2.8 \| 3.0E-1 \| \| GOTERM_BP_FAT \| [response to radiation](http://www.ebi.ac.uk/QuickGO/GTerm?id=GO:0009314) \| 24 \| 1.8 \| 3.4E-2 \| 1.6 \| 3.1E-1 \| \| GOTERM_BP_FAT \| [regulation of peptidyl-tyrosine phosphorylation](http://www.ebi.ac.uk/QuickGO/GTerm?id=GO:0050730) \| 11 \| 0.8 \| 3.4E-2 \| 2.1 \| 3.1E-1 \| \| GOTERM_BP_FAT \| [cytokinesis](http://www.ebi.ac.uk/QuickGO/GTerm?id=GO:0000910) \| 8 \| 0.6 \| 3.5E-2 \| 2.5 \| 3.2E-1 \| \| GOTERM_BP_FAT \| [regulation of myeloid leukocyte differentiation](http://www.ebi.ac.uk/QuickGO/GTerm?id=GO:0002761) \| 8 \| 0.6 \| 3.5E-2 \| 2.5 \| 3.2E-1 \| \| GOTERM_BP_FAT \| [negative regulation of specific transcription from RNA polymerase II promoter](http://www.ebi.ac.uk/QuickGO/GTerm?id=GO:0010553) \| 8 \| 0.6 \| 3.5E-2 \| 2.5 \| 3.2E-1 \| \| GOTERM_BP_FAT \| [regulation of coagulation](http://www.ebi.ac.uk/QuickGO/GTerm?id=GO:0050818) \| 8 \| 0.6 \| 3.5E-2 \| 2.5 \| 3.2E-1 \| \| GOTERM_BP_FAT \| [negative regulation of catalytic activity](http://www.ebi.ac.uk/QuickGO/GTerm?id=GO:0043086) \| 31 \| 2.4 \| 3.5E-2 \| 1.5 \| 3.2E-1 \| \| GOTERM_BP_FAT \| [positive regulation of binding](http://www.ebi.ac.uk/QuickGO/GTerm?id=GO:0051099) \| 12 \| 0.9 \| 3.6E-2 \| 2.0 \| 3.2E-1 \| \| GOTERM_BP_FAT \| [lymphocyte apoptosis](http://www.ebi.ac.uk/QuickGO/GTerm?id=GO:0070227) \| 4 \| 0.3 \| 3.6E-2 \| 5.2 \| 3.2E-1 \| \| GOTERM_BP_FAT \| [negative regulation of tissue remodeling](http://www.ebi.ac.uk/QuickGO/GTerm?id=GO:0034104) \| 4 \| 0.3 \| 3.6E-2 \| 5.2 \| 3.2E-1 \| \| GOTERM_BP_FAT \| [phagocytosis. engulfment](http://www.ebi.ac.uk/QuickGO/GTerm?id=GO:0006911) \| 4 \| 0.3 \| 3.6E-2 \| 5.2 \| 3.2E-1 \| \| GOTERM_BP_FAT \| [chaperone mediated protein folding requiring cofactor](http://www.ebi.ac.uk/QuickGO/GTerm?id=GO:0051085) \| 4 \| 0.3 \| 3.6E-2 \| 5.2 \| 3.2E-1 \| \| GOTERM_BP_FAT \| [phosphoinositide-mediated signaling](http://www.ebi.ac.uk/QuickGO/GTerm?id=GO:0048015) \| 13 \| 1.0 \| 3.6E-2 \| 1.9 \| 3.2E-1 \| \| GOTERM_BP_FAT \| [nucleotide-excision repair. DNA gap filling](http://www.ebi.ac.uk/QuickGO/GTerm?id=GO:0006297) \| 5 \| 0.4 \| 3.7E-2 \| 3.8 \| 3.3E-1 \| \| GOTERM_BP_FAT \| [negative regulation of myeloid leukocyte differentiation](http://www.ebi.ac.uk/QuickGO/GTerm?id=GO:0002762) \| 5 \| 0.4 \| 3.7E-2 \| 3.8 \| 3.3E-1 \| \| GOTERM_BP_FAT \| [positive regulation of interleukin-1 production](http://www.ebi.ac.uk/QuickGO/GTerm?id=GO:0032732) \| 5 \| 0.4 \| 3.7E-2 \| 3.8 \| 3.3E-1 \| \| GOTERM_BP_FAT \| [regeneration](http://www.ebi.ac.uk/QuickGO/GTerm?id=GO:0031099) \| 11 \| 0.8 \| 3.7E-2 \| 2.1 \| 3.3E-1 \| \| GOTERM_BP_FAT \| [regulation of calcium ion transport](http://www.ebi.ac.uk/QuickGO/GTerm?id=GO:0051924) \| 11 \| 0.8 \| 3.7E-2 \| 2.1 \| 3.3E-1 \| \| GOTERM_BP_FAT \| [positive regulation of myeloid cell differentiation](http://www.ebi.ac.uk/QuickGO/GTerm?id=GO:0045639) \| 7 \| 0.5 \| 3.8E-2 \| 2.8 \| 3.3E-1 \| \| GOTERM_BP_FAT \| [regulation of caspase activity](http://www.ebi.ac.uk/QuickGO/GTerm?id=GO:0043281) \| 12 \| 0.9 \| 3.9E-2 \| 2.0 \| 3.4E-1 \| \| GOTERM_BP_FAT \| [positive regulation of transcription factor activity](http://www.ebi.ac.uk/QuickGO/GTerm?id=GO:0051091) \| 10 \| 0.8 \| 3.9E-2 \| 2.2 \| 3.4E-1 \| \| GOTERM_BP_FAT \| [chromosome condensation](http://www.ebi.ac.uk/QuickGO/GTerm?id=GO:0030261) \| 6 \| 0.5 \| 3.9E-2 \| 3.1 \| 3.4E-1 \| \| GOTERM_BP_FAT \| [regulation of leukocyte mediated cytotoxicity](http://www.ebi.ac.uk/QuickGO/GTerm?id=GO:0001910) \| 6 \| 0.5 \| 3.9E-2 \| 3.1 \| 3.4E-1 \| \| GOTERM_BP_FAT \| [regulation of foam cell differentiation](http://www.ebi.ac.uk/QuickGO/GTerm?id=GO:0010743) \| 6 \| 0.5 \| 3.9E-2 \| 3.1 \| 3.4E-1 \| \| GOTERM_BP_FAT \| [cell-cell signaling](http://www.ebi.ac.uk/QuickGO/GTerm?id=GO:0007267) \| 59 \| 4.5 \| 4.0E-2 \| 1.3 \| 3.5E-1 \| \| GOTERM_BP_FAT \| [elevation of cytosolic calcium ion concentration](http://www.ebi.ac.uk/QuickGO/GTerm?id=GO:0007204) \| 15 \| 1.1 \| 4.2E-2 \| 1.8 \| 3.6E-1 \| \| GOTERM_BP_FAT \| [regulation of protein complex assembly](http://www.ebi.ac.uk/QuickGO/GTerm?id=GO:0043254) \| 13 \| 1.0 \| 4.2E-2 \| 1.9 \| 3.6E-1 \| \| GOTERM_BP_FAT \| [negative regulation of response to stimulus](http://www.ebi.ac.uk/QuickGO/GTerm?id=GO:0048585) \| 14 \| 1.1 \| 4.2E-2 \| 1.8 \| 3.6E-1 \| \| GOTERM_BP_FAT \| [response to endoplasmic reticulum stress](http://www.ebi.ac.uk/QuickGO/GTerm?id=GO:0034976) \| 7 \| 0.5 \| 4.3E-2 \| 2.7 \| 3.6E-1 \| \| GOTERM_BP_FAT \| [regulation of cell morphogenesis](http://www.ebi.ac.uk/QuickGO/GTerm?id=GO:0022604) \| 17 \| 1.3 \| 4.4E-2 \| 1.7 \| 3.6E-1 \| \| GOTERM_BP_FAT \| [erythrocyte differentiation](http://www.ebi.ac.uk/QuickGO/GTerm?id=GO:0030218) \| 8 \| 0.6 \| 4.4E-2 \| 2.4 \| 3.7E-1 \| \| GOTERM_BP_FAT \| [response to organic cyclic substance](http://www.ebi.ac.uk/QuickGO/GTerm?id=GO:0014070) \| 16 \| 1.2 \| 4.4E-2 \| 1.7 \| 3.7E-1 \| \| GOTERM_BP_FAT \| [regulation of cytokine production during immune response](http://www.ebi.ac.uk/QuickGO/GTerm?id=GO:0002718) \| 5 \| 0.4 \| 4.5E-2 \| 3.6 \| 3.7E-1 \| \| GOTERM_BP_FAT \| [negative regulation of cellular component organization](http://www.ebi.ac.uk/QuickGO/GTerm?id=GO:0051129) \| 18 \| 1.4 \| 4.5E-2 \| 1.6 \| 3.7E-1 \| \| GOTERM_BP_FAT \| [regulation of cellular component biogenesis](http://www.ebi.ac.uk/QuickGO/GTerm?id=GO:0044087) \| 18 \| 1.4 \| 4.5E-2 \| 1.6 \| 3.7E-1 \| \| GOTERM_BP_FAT \| [response to antibiotic](http://www.ebi.ac.uk/QuickGO/GTerm?id=GO:0046677) \| 6 \| 0.5 \| 4.5E-2 \| 3.0 \| 3.7E-1 \| \| GOTERM_BP_FAT \| [protein-DNA complex assembly](http://www.ebi.ac.uk/QuickGO/GTerm?id=GO:0065004) \| 13 \| 1.0 \| 4.6E-2 \| 1.9 \| 3.7E-1 \| \| GOTERM_BP_FAT \| [viral reproductive process](http://www.ebi.ac.uk/QuickGO/GTerm?id=GO:0022415) \| 10 \| 0.8 \| 4.6E-2 \| 2.1 \| 3.8E-1 \| \| GOTERM_BP_FAT \| [double-strand break repair](http://www.ebi.ac.uk/QuickGO/GTerm?id=GO:0006302) \| 10 \| 0.8 \| 4.6E-2 \| 2.1 \| 3.8E-1 \| \| GOTERM_BP_FAT \| [positive regulation of interferon-gamma biosynthetic process](http://www.ebi.ac.uk/QuickGO/GTerm?id=GO:0045078) \| 4 \| 0.3 \| 4.7E-2 \| 4.7 \| 3.8E-1 \| \| GOTERM_BP_FAT \| [prostaglandin biosynthetic process](http://www.ebi.ac.uk/QuickGO/GTerm?id=GO:0001516) \| 4 \| 0.3 \| 4.7E-2 \| 4.7 \| 3.8E-1 \| \| GOTERM_BP_FAT \| [negative regulation of cell-substrate adhesion](http://www.ebi.ac.uk/QuickGO/GTerm?id=GO:0010812) \| 4 \| 0.3 \| 4.7E-2 \| 4.7 \| 3.8E-1 \| \| GOTERM_BP_FAT \| [prostanoid biosynthetic process](http://www.ebi.ac.uk/QuickGO/GTerm?id=GO:0046457) \| 4 \| 0.3 \| 4.7E-2 \| 4.7 \| 3.8E-1 \| \| GOTERM_BP_FAT \| [negative regulation of T cell differentiation](http://www.ebi.ac.uk/QuickGO/GTerm?id=GO:0045581) \| 4 \| 0.3 \| 4.7E-2 \| 4.7 \| 3.8E-1 \| \| GOTERM_BP_FAT \| [protein maturation](http://www.ebi.ac.uk/QuickGO/GTerm?id=GO:0051604) \| 16 \| 1.2 \| 4.7E-2 \| 1.7 \| 3.8E-1 \| \| GOTERM_BP_FAT \| [defense response to bacterium](http://www.ebi.ac.uk/QuickGO/GTerm?id=GO:0042742) \| 15 \| 1.1 \| 4.8E-2 \| 1.7 \| 3.8E-1 \| \| GOTERM_BP_FAT \| [cellular component disassembly](http://www.ebi.ac.uk/QuickGO/GTerm?id=GO:0022411) \| 9 \| 0.7 \| 4.8E-2 \| 2.2 \| 3.8E-1 \| \| GOTERM_BP_FAT \| [positive regulation of protein complex assembly](http://www.ebi.ac.uk/QuickGO/GTerm?id=GO:0031334) \| 7 \| 0.5 \| 4.8E-2 \| 2.6 \| 3.9E-1 \| \| GOTERM_BP_FAT \| [ER-nuclear signaling pathway](http://www.ebi.ac.uk/QuickGO/GTerm?id=GO:0006984) \| 7 \| 0.5 \| 4.8E-2 \| 2.6 \| 3.9E-1 \| \| GOTERM_BP_FAT \| [negative regulation of organelle organization](http://www.ebi.ac.uk/QuickGO/GTerm?id=GO:0010639) \| 12 \| 0.9 \| 4.9E-2 \| 1.9 \| 3.9E-1 \| \| GOTERM_BP_FAT \| [activation of MAPK activity](http://www.ebi.ac.uk/QuickGO/GTerm?id=GO:0000187) \| 12 \| 0.9 \| 4.9E-2 \| 1.9 \| 3.9E-1 \| |
| --- | --- | --- | --- | --- | --- | --- | --- | --- | --- | --- | --- | --- | --- | --- | --- | --- | --- | --- | --- | --- | --- | --- | --- | --- | --- | --- | --- | --- | --- | --- | --- | --- | --- | --- | --- | --- | --- | --- | --- | --- | --- | --- | --- | --- | --- | --- | --- | --- | --- | --- | --- | --- | --- | --- | --- | --- | --- | --- | --- | --- | --- | --- | --- | --- | --- | --- | --- | --- | --- | --- | --- | --- | --- | --- | --- | --- | --- | --- | --- | --- | --- | --- | --- | --- | --- | --- | --- | --- | --- | --- | --- | --- | --- | --- | --- | --- | --- | --- | --- | --- | --- | --- | --- | --- | --- | --- | --- | --- | --- | --- | --- | --- | --- | --- | --- | --- | --- | --- | --- | --- | --- | --- | --- | --- | --- | --- | --- | --- | --- | --- | --- | --- | --- | --- | --- | --- | --- | --- | --- | --- | --- | --- | --- | --- | --- | --- | --- | --- | --- | --- | --- | --- | --- | --- | --- | --- | --- | --- | --- | --- | --- | --- | --- | --- | --- | --- | --- | --- | --- | --- | --- | --- | --- | --- | --- | --- | --- | --- | --- | --- | --- | --- | --- | --- | --- | --- | --- | --- | --- | --- | --- | --- | --- | --- | --- | --- | --- | --- | --- | --- | --- | --- | --- | --- | --- | --- | --- | --- | --- | --- | --- | --- | --- | --- | --- | --- | --- | --- | --- | --- | --- | --- | --- | --- | --- | --- | --- | --- | --- | --- | --- | --- | --- | --- | --- | --- | --- | --- | --- | --- | --- | --- | --- | --- | --- | --- | --- | --- | --- | --- | --- | --- | --- | --- | --- | --- | --- | --- | --- | --- | --- | --- | --- | --- | --- | --- | --- | --- | --- | --- | --- | --- | --- | --- | --- | --- | --- | --- | --- | --- | --- | --- | --- | --- | --- | --- | --- | --- | --- | --- | --- | --- | --- | --- | --- | --- | --- | --- | --- | --- | --- | --- | --- | --- | --- | --- | --- | --- | --- | --- | --- | --- | --- | --- | --- | --- | --- | --- | --- | --- | --- | --- | --- | --- | --- | --- | --- | --- | --- | --- | --- | --- | --- | --- | --- | --- | --- | --- | --- | --- | --- | --- | --- | --- | --- | --- | --- | --- | --- | --- | --- | --- | --- | --- | --- | --- | --- | --- | --- | --- | --- | --- | --- | --- | --- | --- | --- | --- | --- | --- | --- | --- | --- | --- | --- | --- | --- | --- | --- | --- | --- | --- | --- | --- | --- | --- | --- | --- | --- | --- | --- | --- | --- | --- | --- | --- | --- | --- | --- | --- | --- | --- | --- | --- | --- | --- | --- | --- | --- | --- | --- | --- | --- | --- | --- | --- | --- | --- | --- | --- | --- | --- | --- | --- | --- | --- | --- | --- | --- | --- | --- | --- | --- | --- | --- | --- | --- | --- | --- | --- | --- | --- | --- | --- | --- | --- | --- | --- | --- | --- | --- | --- | --- | --- | --- | --- | --- | --- | --- | --- | --- | --- | --- | --- | --- | --- | --- | --- | --- | --- | --- | --- | --- | --- | --- | --- | --- | --- | --- | --- | --- | --- | --- | --- | --- | --- | --- | --- | --- | --- | --- | --- | --- | --- | --- | --- | --- | --- | --- | --- | --- | --- | --- | --- | --- | --- | --- | --- | --- | --- | --- | --- | --- | --- | --- | --- | --- | --- | --- | --- | --- | --- | --- | --- | --- | --- | --- | --- | --- | --- | --- | --- | --- | --- | --- | --- | --- | --- | --- | --- | --- | --- | --- | --- | --- | --- | --- | --- | --- | --- | --- | --- | --- | --- | --- | --- | --- | --- | --- | --- | --- | --- | --- | --- | --- | --- | --- | --- | --- | --- | --- | --- | --- | --- | --- | --- | --- | --- | --- | --- | --- | --- | --- | --- | --- | --- | --- | --- | --- | --- | --- | --- | --- | --- | --- | --- | --- | --- | --- | --- | --- | --- | --- | --- | --- | --- | --- | --- | --- | --- | --- | --- | --- | --- | --- | --- | --- | --- | --- | --- | --- | --- | --- | --- | --- | --- | --- | --- | --- | --- | --- | --- | --- | --- | --- | --- | --- | --- | --- | --- | --- | --- | --- | --- | --- | --- | --- | --- | --- | --- | --- | --- | --- | --- | --- | --- | --- | --- | --- | --- | --- | --- | --- | --- | --- | --- | --- | --- | --- | --- | --- | --- | --- | --- | --- | --- | --- | --- | --- | --- | --- | --- | --- | --- | --- | --- | --- | --- | --- | --- | --- | --- | --- | --- | --- | --- | --- | --- | --- | --- | --- | --- | --- | --- | --- | --- | --- | --- | --- | --- | --- | --- | --- | --- | --- | --- | --- | --- | --- | --- | --- | --- | --- | --- | --- | --- | --- | --- | --- | --- | --- | --- | --- | --- | --- | --- | --- | --- | --- | --- | --- | --- | --- | --- | --- | --- | --- | --- | --- | --- | --- | --- | --- | --- | --- | --- | --- | --- | --- | --- | --- | --- | --- | --- | --- | --- | --- | --- | --- | --- | --- | --- | --- | --- | --- | --- | --- | --- | --- | --- | --- | --- | --- | --- | --- | --- | --- | --- | --- | --- | --- | --- | --- | --- | --- | --- | --- | --- | --- | --- | --- | --- | --- | --- | --- | --- | --- | --- | --- | --- | --- | --- | --- | --- | --- | --- | --- | --- | --- | --- | --- | --- | --- | --- | --- | --- | --- | --- | --- | --- | --- | --- | --- | --- | --- | --- | --- | --- | --- | --- | --- | --- | --- | --- | --- | --- | --- | --- | --- | --- | --- | --- | --- | --- | --- | --- | --- | --- | --- | --- | --- | --- | --- | --- | --- | --- | --- | --- | --- | --- | --- | --- | --- | --- | --- | --- | --- | --- | --- | --- | --- | --- | --- | --- | --- | --- | --- | --- | --- | --- | --- | --- | --- | --- | --- | --- | --- | --- | --- | --- | --- | --- | --- | --- | --- | --- | --- | --- | --- | --- | --- | --- | --- | --- | --- | --- | --- | --- | --- | --- | --- | --- | --- | --- | --- | --- | --- | --- | --- | --- | --- | --- | --- | --- | --- | --- | --- | --- | --- | --- | --- | --- | --- | --- | --- | --- | --- | --- | --- | --- | --- | --- | --- | --- | --- | --- | --- | --- | --- | --- | --- | --- | --- | --- | --- | --- | --- | --- | --- | --- | --- | --- | --- | --- | --- | --- | --- | --- | --- | --- | --- | --- | --- | --- | --- | --- | --- | --- | --- | --- | --- | --- | --- | --- | --- | --- | --- | --- | --- | --- | --- | --- | --- | --- | --- | --- | --- | --- | --- | --- | --- | --- | --- | --- | --- | --- | --- | --- | --- | --- | --- | --- | --- | --- | --- | --- | --- | --- | --- | --- | --- | --- | --- | --- | --- | --- | --- | --- | --- | --- | --- | --- | --- | --- | --- | --- | --- | --- | --- | --- | --- | --- | --- | --- | --- | --- | --- | --- | --- | --- | --- | --- | --- | --- | --- | --- | --- | --- | --- | --- | --- | --- | --- | --- | --- | --- | --- | --- | --- | --- | --- | --- | --- | --- | --- | --- | --- | --- | --- | --- | --- | --- | --- | --- | --- | --- | --- | --- | --- | --- | --- | --- | --- | --- | --- | --- | --- | --- | --- | --- | --- | --- | --- | --- | --- | --- | --- | --- | --- | --- | --- | --- | --- | --- | --- | --- | --- | --- | --- | --- | --- | --- | --- | --- | --- | --- | --- | --- | --- | --- | --- | --- | --- | --- | --- | --- | --- | --- | --- | --- | --- | --- | --- | --- | --- | --- | --- | --- | --- | --- | --- | --- | --- | --- | --- | --- | --- | --- | --- | --- | --- | --- | --- | --- | --- | --- | --- | --- | --- | --- | --- | --- | --- | --- | --- | --- | --- | --- | --- | --- | --- | --- | --- | --- | --- | --- | --- | --- | --- | --- | --- | --- | --- | --- | --- | --- | --- | --- | --- | --- | --- | --- | --- | --- | --- | --- | --- | --- | --- | --- | --- | --- | --- | --- | --- | --- | --- | --- | --- | --- | --- | --- | --- | --- | --- | --- | --- | --- | --- | --- | --- | --- | --- | --- | --- | --- | --- | --- | --- | --- | --- | --- | --- | --- | --- | --- | --- | --- | --- | --- | --- | --- | --- | --- | --- | --- | --- | --- | --- | --- | --- | --- | --- | --- | --- | --- | --- | --- | --- | --- | --- | --- | --- | --- | --- | --- | --- | --- | --- | --- | --- | --- | --- | --- | --- | --- | --- | --- | --- | --- | --- | --- | --- | --- | --- | --- | --- | --- | --- | --- | --- | --- | --- | --- | --- | --- | --- | --- | --- | --- | --- | --- | --- | --- | --- | --- | --- | --- | --- | --- | --- | --- | --- | --- | --- | --- | --- | --- | --- | --- | --- | --- | --- | --- | --- | --- | --- | --- | --- | --- | --- | --- | --- | --- | --- | --- | --- | --- | --- | --- | --- | --- | --- | --- | --- | --- | --- | --- | --- | --- | --- | --- | --- | --- | --- | --- | --- | --- | --- | --- | --- | --- | --- | --- | --- | --- | --- | --- | --- | --- | --- | --- | --- | --- | --- | --- | --- | --- | --- | --- | --- | --- | --- | --- | --- | --- | --- | --- | --- | --- | --- | --- | --- | --- | --- | --- | --- | --- | --- | --- | --- | --- | --- | --- | --- | --- | --- | --- | --- | --- | --- | --- | --- | --- | --- | --- | --- | --- | --- | --- | --- | --- | --- | --- | --- | --- | --- | --- | --- | --- | --- | --- | --- | --- | --- | --- | --- | --- | --- | --- | --- | --- | --- | --- | --- | --- | --- | --- | --- | --- | --- | --- | --- | --- | --- | --- | --- | --- | --- | --- | --- | --- | --- | --- | --- | --- | --- | --- | --- | --- | --- | --- | --- | --- | --- | --- | --- | --- | --- | --- | --- | --- | --- | --- | --- | --- | --- | --- | --- | --- | --- | --- | --- | --- | --- | --- | --- | --- | --- | --- | --- | --- | --- | --- | --- | --- | --- | --- | --- | --- | --- | --- | --- | --- | --- | --- | --- | --- | --- | --- | --- | --- | --- | --- | --- | --- | --- | --- | --- | --- | --- | --- | --- | --- | --- | --- | --- | --- | --- | --- | --- | --- | --- | --- | --- | --- | --- | --- | --- | --- | --- | --- | --- | --- | --- | --- | --- | --- | --- | --- | --- | --- | --- | --- | --- | --- | --- | --- | --- | --- | --- | --- | --- | --- | --- | --- | --- | --- | --- | --- | --- | --- | --- | --- | --- | --- | --- | --- | --- | --- | --- | --- | --- | --- | --- | --- | --- | --- | --- | --- | --- | --- | --- | --- | --- | --- | --- | --- | --- | --- | --- | --- | --- | --- | --- | --- | --- | --- | --- | --- | --- | --- | --- | --- | --- | --- | --- | --- | --- | --- | --- | --- | --- | --- | --- | --- | --- | --- | --- | --- | --- | --- | --- | --- | --- | --- | --- | --- | --- | --- | --- | --- | --- | --- | --- | --- | --- | --- | --- | --- | --- | --- | --- | --- | --- | --- | --- | --- | --- | --- | --- | --- | --- | --- | --- | --- | --- | --- | --- | --- | --- | --- | --- | --- | --- | --- | --- | --- | --- | --- | --- | --- | --- | --- | --- | --- | --- | --- | --- | --- | --- | --- | --- | --- | --- | --- | --- | --- | --- | --- | --- | --- | --- | --- | --- | --- | --- | --- | --- | --- | --- | --- | --- | --- | --- | --- | --- | --- | --- | --- | --- | --- | --- | --- | --- | --- | --- | --- | --- | --- | --- | --- | --- | --- | --- | --- | --- | --- | --- | --- | --- | --- | --- | --- | --- | --- | --- | --- | --- | --- | --- | --- | --- | --- | --- | --- | --- | --- | --- | --- | --- | --- | --- | --- | --- | --- | --- | --- | --- | --- | --- | --- | --- | --- | --- | --- | --- | --- | --- | --- | --- | --- | --- | --- | --- | --- | --- | --- | --- | --- | --- | --- | --- | --- | --- | --- | --- | --- | --- | --- | --- | --- | --- | --- | --- | --- | --- | --- | --- | --- | --- | --- | --- | --- | --- | --- | --- | --- | --- | --- | --- | --- | --- | --- | --- | --- | --- | --- | --- | --- | --- | --- | --- | --- | --- | --- | --- | --- | --- | --- | --- | --- | --- | --- | --- | --- | --- | --- | --- | --- | --- | --- | --- | --- | --- | --- | --- | --- | --- | --- | --- | --- | --- | --- | --- | --- | --- | --- | --- | --- | --- | --- | --- | --- | --- | --- | --- | --- | --- | --- | --- | --- | --- | --- | --- | --- | --- | --- | --- | --- | --- | --- | --- | --- | --- | --- | --- | --- | --- | --- | --- | --- | --- | --- | --- | --- | --- | --- | --- | --- | --- | --- | --- | --- | --- | --- | --- | --- | --- | --- | --- | --- | --- | --- | --- | --- | --- | --- | --- | --- | --- | --- | --- | --- | --- | --- | --- | --- | --- | --- | --- | --- | --- | --- | --- | --- | --- | --- | --- | --- | --- | --- | --- | --- | --- | --- | --- | --- | --- | --- | --- | --- | --- | --- | --- | --- | --- | --- | --- | --- | --- | --- | --- | --- | --- | --- | --- | --- | --- | --- | --- | --- | --- | --- | --- | --- | --- | --- | --- | --- | --- | --- | --- | --- | --- | --- | --- | --- | --- | --- | --- | --- | --- | --- | --- | --- | --- | --- | --- | --- | --- | --- | --- | --- | --- | --- | --- | --- | --- | --- | --- | --- | --- | --- | --- | --- | --- | --- | --- | --- | --- | --- | --- | --- | --- | --- | --- | --- | --- | --- | --- | --- | --- | --- | --- | --- | --- | --- | --- | --- | --- | --- | --- | --- | --- | --- | --- | --- | --- | --- | --- | --- | --- | --- | --- | --- | --- | --- | --- | --- | --- | --- | --- | --- | --- | --- | --- | --- | --- | --- | --- | --- | --- | --- | --- | --- | --- | --- | --- | --- | --- | --- | --- | --- | --- | --- | --- | --- | --- | --- | --- | --- | --- | --- | --- | --- | --- | --- | --- | --- | --- | --- | --- | --- | --- | --- | --- | --- | --- | --- | --- | --- | --- | --- | --- | --- | --- | --- | --- | --- | --- | --- | --- | --- | --- | --- | --- | --- | --- | --- | --- | --- | --- | --- | --- | --- | --- | --- | --- | --- | --- | --- | --- | --- | --- | --- | --- | --- | --- | --- | --- | --- | --- | --- | --- | --- | --- | --- | --- | --- | --- | --- | --- | --- | --- | --- | --- | --- | --- | --- | --- | --- | --- | --- | --- | --- | --- | --- | --- | --- | --- | --- | --- | --- | --- | --- | --- | --- | --- | --- | --- | --- | --- | --- | --- | --- | --- | --- | --- | --- | --- | --- | --- | --- | --- | --- | --- | --- | --- | --- | --- | --- | --- | --- | --- | --- | --- | --- | --- | --- | --- | --- | --- | --- | --- | --- | --- | --- | --- | --- | --- | --- | --- | --- | --- | --- | --- | --- | --- | --- | --- | --- | --- | --- | --- | --- | --- | --- | --- | --- | --- | --- | --- | --- | --- | --- | --- | --- | --- | --- | --- | --- | --- | --- | --- | --- | --- | --- | --- | --- | --- | --- | --- | --- | --- | --- | --- | --- | --- | --- | --- | --- | --- | --- | --- | --- | --- | --- | --- | --- | --- | --- | --- | --- | --- | --- | --- | --- | --- | --- | --- | --- | --- | --- | --- | --- | --- | --- | --- | --- | --- | --- | --- | --- | --- | --- | --- | --- | --- | --- | --- | --- | --- | --- | --- | --- | --- | --- | --- | --- | --- | --- | --- | --- | --- | --- | --- | --- | --- | --- | --- | --- | --- | --- | --- | --- | --- | --- | --- | --- | --- | --- | --- | --- | --- | --- | --- | --- | --- | --- | --- | --- | --- | --- | --- | --- | --- | --- | --- | --- | --- | --- | --- | --- | --- | --- | --- | --- | --- | --- | --- | --- | --- | --- | --- | --- | --- | --- | --- | --- | --- | --- | --- | --- | --- | --- | --- | --- | --- | --- | --- | --- | --- | --- | --- | --- | --- | --- | --- | --- | --- | --- | --- | --- | --- | --- | --- | --- | --- | --- | --- | --- | --- | --- | --- | --- | --- | --- | --- | --- | --- | --- | --- | --- | --- | --- | --- | --- | --- | --- | --- | --- | --- | --- | --- | --- | --- | --- | --- | --- | --- | --- | --- | --- | --- | --- | --- | --- | --- | --- | --- | --- | --- | --- | --- | --- | --- | --- | --- | --- | --- | --- | --- | --- | --- | --- | --- | --- | --- | --- | --- | --- | --- | --- | --- | --- | --- | --- | --- | --- | --- | --- | --- | --- | --- | --- | --- | --- | --- | --- | --- | --- | --- | --- | --- | --- | --- | --- | --- | --- | --- | --- | --- | --- | --- | --- | --- | --- | --- | --- | --- | --- | --- | --- | --- | --- | --- | --- | --- | --- | --- | --- | --- | --- | --- | --- | --- | --- | --- | --- | --- | --- | --- | --- | --- | --- | --- | --- | --- | --- | --- | --- | --- | --- | --- | --- | --- | --- | --- | --- | --- | --- | --- | --- | --- | --- | --- | --- | --- | --- | --- | --- | --- | --- | --- | --- | --- | --- | --- | --- | --- | --- | --- | --- | --- | --- | --- | --- | --- | --- | --- | --- | --- | --- | --- | --- | --- | --- | --- | --- | --- | --- | --- | --- | --- | --- | --- | --- | --- | --- | --- | --- | --- | --- | --- | --- | --- | --- | --- | --- | --- | --- | --- | --- | --- | --- | --- | --- | --- | --- | --- | --- | --- | --- | --- | --- | --- | --- | --- | --- | --- | --- | --- | --- | --- | --- | --- | --- | --- | --- | --- | --- | --- | --- | --- | --- | --- | --- | --- | --- | --- | --- | --- | --- | --- | --- | --- | --- | --- | --- | --- | --- | --- | --- | --- | --- | --- | --- | --- | --- | --- | --- | --- | --- | --- | --- | --- | --- | --- | --- | --- | --- | --- | --- | --- | --- | --- | --- | --- | --- | --- | --- | --- | --- | --- | --- | --- | --- | --- | --- | --- | --- | --- | --- | --- | --- | --- | --- | --- | --- | --- | --- | --- | --- | --- | --- | --- | --- | --- | --- | --- | --- | --- | --- | --- | --- | --- | --- | --- | --- | --- | --- | --- | --- | --- | --- | --- | --- | --- | --- | --- | --- | --- | --- | --- | --- | --- | --- | --- | --- | --- | --- | --- | --- | --- | --- | --- | --- | --- | --- | --- | --- | --- | --- | --- | --- | --- | --- | --- | --- | --- | --- | --- | --- | --- | --- | --- | --- | --- | --- | --- | --- | --- | --- | --- | --- | --- | --- | --- | --- | --- | --- | --- | --- | --- | --- | --- | --- | --- | --- | --- | --- | --- | --- | --- | --- | --- | --- | --- | --- | --- | --- | --- | --- | --- | --- | --- | --- | --- | --- | --- | --- | --- | --- | --- | --- | --- | --- | --- | --- | --- | --- | --- | --- | --- | --- | --- | --- | --- | --- | --- | --- | --- | --- | --- | --- | --- | --- | --- | --- | --- | --- | --- | --- | --- | --- | --- | --- | --- | --- | --- | --- | --- | --- | --- | --- | --- | --- | --- | --- | --- | --- | --- | --- | --- | --- | --- | --- | --- | --- | --- | --- | --- | --- | --- | --- | --- | --- | --- | --- | --- | --- | --- | --- | --- | --- | --- | --- | --- | --- | --- | --- | --- | --- | --- | --- | --- | --- | --- | --- | --- | --- | --- | --- | --- | --- | --- | --- | --- | --- | --- | --- | --- | --- | --- | --- | --- | --- | --- | --- | --- | --- | --- | --- | --- | --- | --- | --- | --- | --- | --- | --- | --- | --- | --- | --- | --- | --- | --- | --- | --- | --- | --- | --- | --- | --- | --- | --- | --- | --- | --- | --- | --- | --- | --- | --- | --- | --- | --- | --- | --- | --- | --- | --- | --- | --- | --- | --- | --- | --- | --- | --- | --- | --- | --- | --- | --- | --- | --- | --- | --- | --- | --- | --- | --- | --- | --- | --- | --- | --- | --- | --- | --- | --- | --- | --- | --- | --- | --- | --- | --- | --- | --- | --- | --- | --- | --- | --- | --- | --- | --- | --- | --- | --- | --- | --- | --- | --- | --- | --- | --- | --- | --- | --- | --- | --- | --- | --- | --- | --- | --- | --- | --- | --- | --- | --- | --- | --- | --- | --- | --- | --- | --- | --- | --- | --- | --- | --- | --- | --- | --- | --- | --- | --- | --- | --- | --- | --- | --- | --- | --- | --- | --- | --- | --- | --- | --- | --- | --- | --- | --- | --- | --- | --- | --- | --- | --- | --- | --- | --- | --- | --- | --- | --- | --- | --- | --- | --- | --- | --- | --- | --- | --- | --- | --- | --- | --- | --- | --- | --- | --- | --- | --- | --- | --- | --- | --- | --- | --- | --- | --- | --- | --- | --- | --- | --- | --- | --- | --- | --- | --- | --- | --- | --- | --- | --- | --- | --- | --- | --- | --- | --- | --- | --- | --- | --- | --- | --- | --- | --- | --- | --- | --- | --- | --- | --- | --- | --- | --- | --- | --- | --- | --- | --- | --- | --- | --- | --- | --- | --- | --- | --- | --- | --- | --- | --- | --- | --- | --- | --- | --- | --- | --- | --- | --- | --- | --- | --- | --- | --- | --- | --- | --- | --- | --- | --- | --- | --- | --- | --- | --- | --- | --- | --- | --- | --- | --- | --- | --- | --- | --- | --- | --- | --- | --- | --- | --- | --- | --- | --- | --- | --- | --- | --- | --- | --- | --- | --- | --- | --- | --- | --- | --- | --- | --- | --- | --- | --- | --- | --- | --- | --- | --- | --- | --- | --- | --- | --- | --- | --- | --- | --- | --- | --- | --- | --- | --- | --- | --- | --- | --- | --- | --- | --- | --- | --- | --- | --- | --- | --- | --- | --- | --- | --- | --- | --- | --- | --- | --- | --- | --- | --- | --- | --- | --- | --- | --- | --- | --- | --- | --- | --- | --- | --- | --- | --- | --- | --- | --- | --- | --- | --- | --- | --- | --- | --- | --- | --- | --- | --- | --- | --- | --- | --- | --- | --- | --- | --- | --- | --- | --- | --- | --- | --- | --- | --- | --- | --- | --- | --- | --- | --- | --- | --- | --- | --- | --- | --- | --- | --- | --- | --- | --- | --- | --- | --- | --- | --- | --- | --- | --- | --- | --- | --- | --- | --- | --- | --- | --- | --- | --- | --- | --- | --- | --- | --- | --- | --- | --- | --- | --- | --- | --- | --- | --- | --- | --- | --- | --- | --- | --- | --- | --- | --- | --- | --- | --- | --- | --- | --- | --- | --- | --- | --- | --- | --- | --- | --- | --- | --- | --- | --- | --- | --- | --- | --- | --- | --- | --- | --- | --- | --- | --- | --- | --- | --- | --- | --- | --- | --- | --- | --- | --- | --- | --- | --- | --- | --- | --- | --- | --- | --- | --- | --- | --- | --- | --- | --- | --- | --- | --- | --- | --- | --- | --- | --- | --- | --- | --- | --- | --- | --- | --- | --- | --- | --- | --- | --- | --- | --- | --- | --- | --- | --- | --- | --- | --- | --- | --- | --- | --- | --- | --- | --- | --- | --- | --- | --- | --- | --- | --- | --- | --- | --- | --- | --- | --- | --- | --- | --- | --- | --- | --- | --- | --- | --- | --- | --- | --- | --- | --- | --- | --- | --- | --- | --- | --- | --- | --- | --- | --- | --- | --- | --- | --- | --- | --- | --- | --- | --- | --- | --- | --- | --- | --- | --- | --- | --- | --- | --- | --- | --- | --- | --- | --- | --- | --- | --- | --- | --- | --- | --- | --- | --- | --- | --- | --- | --- |
|  |
