# Supplementary material for: Genome-Wide Analysis of Differentially Expressed Genes and Splicing Isoforms in Clear Cell Renal Cell Carcinoma
Source: PLoS One. 2013 Oct 23;8(10):e78452. doi: 10.1371/journal.pone.0078452 (PMC3806822; doi:10.1371/journal.pone.0078452)
Supplement: Table S7 — KEGG pathway enrichment analysis of genes up-regulated in ccRCC performed by DAVID. Annotations were considered significantly over-represented when the p-value of the Fisher's exact test as used by DAVID (EASE Score) was < 0.05 and gene counts belonging to an annotation term was equal or greater than 2. (DOCX) [file pone.0078452.s007.docx]

| [Category](http://david.abcc.ncifcrf.gov/chartReport.jsp?visited=yes&d-16544-s=1&cbBenjamini=true&rowids=&count=2&d-16544-o=2&cbFC=true&d-16544-p=1&annot=47&ease=0.1&numRecords=1000&heading=) | [Term](http://david.abcc.ncifcrf.gov/chartReport.jsp?visited=yes&d-16544-s=2&cbBenjamini=true&rowids=&count=2&d-16544-o=2&cbFC=true&d-16544-p=1&annot=47&ease=0.1&numRecords=1000&heading=) | [Count](http://david.abcc.ncifcrf.gov/chartReport.jsp?visited=yes&d-16544-s=5&cbBenjamini=true&rowids=&count=2&d-16544-o=1&cbFC=true&d-16544-p=1&annot=47&ease=0.1&numRecords=1000&heading=) | [%](http://david.abcc.ncifcrf.gov/chartReport.jsp?visited=yes&d-16544-s=6&cbBenjamini=true&rowids=&count=2&d-16544-o=1&cbFC=true&d-16544-p=1&annot=47&ease=0.1&numRecords=1000&heading=) | [P-Value](http://david.abcc.ncifcrf.gov/chartReport.jsp?visited=yes&d-16544-s=7&cbBenjamini=true&rowids=&count=2&d-16544-o=1&cbFC=true&d-16544-p=1&annot=47&ease=0.1&numRecords=1000&heading=) | [Fold Enrichment](http://david.abcc.ncifcrf.gov/chartReport.jsp?visited=yes&d-16544-s=8&cbBenjamini=true&rowids=&count=2&d-16544-o=1&cbFC=true&d-16544-p=1&annot=47&ease=0.1&numRecords=1000&heading=) | [Benjamini](http://david.abcc.ncifcrf.gov/chartReport.jsp?visited=yes&d-16544-s=9&cbBenjamini=true&rowids=&count=2&d-16544-o=1&cbFC=true&d-16544-p=1&annot=47&ease=0.1&numRecords=1000&heading=) |
| --- | --- | --- | --- | --- | --- | --- |
| KEGG_PATHWAY | [Cell adhesion molecules (CAMs)](http://david.abcc.ncifcrf.gov/kegg.jsp?path=hsa04514$Cell%20adhesion%20molecules%20(CAMs)&termId=470038855&source=kegg) | 35 | 2.7 | 5.2E-8 | 2.7 | 8.2E-6 |
| KEGG_PATHWAY | [Viral myocarditis](http://david.abcc.ncifcrf.gov/kegg.jsp?path=hsa05416$Viral%20myocarditis&termId=470038923&source=kegg) | 24 | 1.8 | 8.9E-8 | 3.5 | 7.1E-6 |
| KEGG_PATHWAY | [Systemic lupus erythematosus](http://david.abcc.ncifcrf.gov/kegg.jsp?path=hsa05322$Systemic%20lupus%20erythematosus&termId=470038916&source=kegg) | 28 | 2.1 | 3.7E-7 | 2.9 | 2.0E-5 |
| KEGG_PATHWAY | [Natural killer cell mediated cytotoxicity](http://david.abcc.ncifcrf.gov/kegg.jsp?path=hsa04650$Natural%20killer%20cell%20mediated%20cytotoxicity&termId=470038868&source=kegg) | 33 | 2.5 | 7.0E-7 | 2.6 | 2.8E-5 |
| KEGG_PATHWAY | [p53 signaling pathway](http://david.abcc.ncifcrf.gov/kegg.jsp?path=hsa04115$p53%20signaling%20pathway&termId=470038836&source=kegg) | 22 | 1.7 | 8.1E-7 | 3.3 | 2.6E-5 |
| KEGG_PATHWAY | [Intestinal immune network for IgA production](http://david.abcc.ncifcrf.gov/kegg.jsp?path=hsa04672$Intestinal%20immune%20network%20for%20IgA%20production&termId=470038874&source=kegg) | 18 | 1.4 | 1.5E-6 | 3.8 | 4.0E-5 |
| KEGG_PATHWAY | [Allograft rejection](http://david.abcc.ncifcrf.gov/kegg.jsp?path=hsa05330$Allograft%20rejection&termId=470038917&source=kegg) | 15 | 1.1 | 2.7E-6 | 4.3 | 6.1E-5 |
| KEGG_PATHWAY | [Graft-versus-host disease](http://david.abcc.ncifcrf.gov/kegg.jsp?path=hsa05332$Graft-versus-host%20disease&termId=470038918&source=kegg) | 15 | 1.1 | 8.1E-6 | 4.0 | 1.6E-4 |
| KEGG_PATHWAY | [Primary immunodeficiency](http://david.abcc.ncifcrf.gov/kegg.jsp?path=hsa05340$Primary%20immunodeficiency&termId=470038919&source=kegg) | 13 | 1.0 | 6.1E-5 | 3.8 | 1.1E-3 |
| KEGG_PATHWAY | [Toll-like receptor signaling pathway](http://david.abcc.ncifcrf.gov/kegg.jsp?path=hsa04620$Toll-like%20receptor%20signaling%20pathway&termId=470038862&source=kegg) | 24 | 1.8 | 6.7E-5 | 2.5 | 1.1E-3 |
| KEGG_PATHWAY | [T cell receptor signaling pathway](http://david.abcc.ncifcrf.gov/kegg.jsp?path=hsa04660$T%20cell%20receptor%20signaling%20pathway&termId=470038869&source=kegg) | 25 | 1.9 | 7.1E-5 | 2.4 | 1.0E-3 |
| KEGG_PATHWAY | [DNA replication](http://david.abcc.ncifcrf.gov/kegg.jsp?path=hsa03030$DNA%20replication&termId=470038817&source=kegg) | 13 | 1.0 | 8.4E-5 | 3.7 | 1.1E-3 |
| KEGG_PATHWAY | [Type I diabetes mellitus](http://david.abcc.ncifcrf.gov/kegg.jsp?path=hsa04940$Type%20I%20diabetes%20mellitus&termId=470038888&source=kegg) | 14 | 1.1 | 1.0E-4 | 3.4 | 1.2E-3 |
| KEGG_PATHWAY | [Chemokine signaling pathway](http://david.abcc.ncifcrf.gov/kegg.jsp?path=hsa04062$Chemokine%20signaling%20pathway&termId=470038831&source=kegg) | 35 | 2.7 | 1.9E-4 | 1.9 | 2.1E-3 |
| KEGG_PATHWAY | [Autoimmune thyroid disease](http://david.abcc.ncifcrf.gov/kegg.jsp?path=hsa05320$Autoimmune%20thyroid%20disease&termId=470038915&source=kegg) | 15 | 1.1 | 2.3E-4 | 3.0 | 2.5E-3 |
| KEGG_PATHWAY | [Cell cycle](http://david.abcc.ncifcrf.gov/kegg.jsp?path=hsa04110$Cell%20cycle&termId=470038834&source=kegg) | 25 | 1.9 | 7.4E-4 | 2.1 | 7.3E-3 |
| KEGG_PATHWAY | [Antigen processing and presentation](http://david.abcc.ncifcrf.gov/kegg.jsp?path=hsa04612$Antigen%20processing%20and%20presentation&termId=470038860&source=kegg) | 19 | 1.5 | 7.7E-4 | 2.4 | 7.2E-3 |
| KEGG_PATHWAY | [Pathways in cancer](http://david.abcc.ncifcrf.gov/kegg.jsp?path=hsa05200$Pathways%20in%20cancer&termId=470038899&source=kegg) | 50 | 3.8 | 1.1E-3 | 1.6 | 9.6E-3 |
| KEGG_PATHWAY | [Hematopoietic cell lineage](http://david.abcc.ncifcrf.gov/kegg.jsp?path=hsa04640$Hematopoietic%20cell%20lineage&termId=470038867&source=kegg) | 19 | 1.5 | 1.2E-3 | 2.3 | 9.9E-3 |
| KEGG_PATHWAY | [Cytokine-cytokine receptor interaction](http://david.abcc.ncifcrf.gov/kegg.jsp?path=hsa04060$Cytokine-cytokine%20receptor%20interaction&termId=470038830&source=kegg) | 41 | 3.1 | 2.1E-3 | 1.6 | 1.6E-2 |
| KEGG_PATHWAY | [Focal adhesion](http://david.abcc.ncifcrf.gov/kegg.jsp?path=hsa04510$Focal%20adhesion&termId=470038853&source=kegg) | 33 | 2.5 | 2.9E-3 | 1.7 | 2.2E-2 |
| KEGG_PATHWAY | [Pancreatic cancer](http://david.abcc.ncifcrf.gov/kegg.jsp?path=hsa05212$Pancreatic%20cancer&termId=470038902&source=kegg) | 16 | 1.2 | 3.1E-3 | 2.3 | 2.2E-2 |
| KEGG_PATHWAY | [Fc gamma R-mediated phagocytosis](http://david.abcc.ncifcrf.gov/kegg.jsp?path=hsa04666$Fc%20gamma%20R-mediated%20phagocytosis&termId=470038872&source=kegg) | 19 | 1.5 | 3.8E-3 | 2.1 | 2.6E-2 |
| KEGG_PATHWAY | [Leukocyte transendothelial migration](http://david.abcc.ncifcrf.gov/kegg.jsp?path=hsa04670$Leukocyte%20transendothelial%20migration&termId=470038873&source=kegg) | 22 | 1.7 | 4.1E-3 | 1.9 | 2.7E-2 |
| KEGG_PATHWAY | [B cell receptor signaling pathway](http://david.abcc.ncifcrf.gov/kegg.jsp?path=hsa04662$B%20cell%20receptor%20signaling%20pathway&termId=470038870&source=kegg) | 16 | 1.2 | 4.7E-3 | 2.2 | 3.0E-2 |
| KEGG_PATHWAY | [Asthma](http://david.abcc.ncifcrf.gov/kegg.jsp?path=hsa05310$Asthma&termId=470038914&source=kegg) | 9 | 0.7 | 5.0E-3 | 3.2 | 3.0E-2 |
| KEGG_PATHWAY | [Bladder cancer](http://david.abcc.ncifcrf.gov/kegg.jsp?path=hsa05219$Bladder%20cancer&termId=470038909&source=kegg) | 11 | 0.8 | 5.6E-3 | 2.7 | 3.3E-2 |
| KEGG_PATHWAY | [ECM-receptor interaction](http://david.abcc.ncifcrf.gov/kegg.jsp?path=hsa04512$ECM-receptor%20interaction&termId=470038854&source=kegg) | 17 | 1.3 | 5.9E-3 | 2.1 | 3.3E-2 |
| KEGG_PATHWAY | [Chronic myeloid leukemia](http://david.abcc.ncifcrf.gov/kegg.jsp?path=hsa05220$Chronic%20myeloid%20leukemia&termId=470038910&source=kegg) | 15 | 1.1 | 1.2E-2 | 2.1 | 6.2E-2 |
| KEGG_PATHWAY | [Jak-STAT signaling pathway](http://david.abcc.ncifcrf.gov/kegg.jsp?path=hsa04630$Jak-STAT%20signaling%20pathway&termId=470038866&source=kegg) | 25 | 1.9 | 1.3E-2 | 1.7 | 6.8E-2 |
| KEGG_PATHWAY | [Small cell lung cancer](http://david.abcc.ncifcrf.gov/kegg.jsp?path=hsa05222$Small%20cell%20lung%20cancer&termId=470038912&source=kegg) | 16 | 1.2 | 1.4E-2 | 2.0 | 6.8E-2 |
| KEGG_PATHWAY | [Pathogenic Escherichia coli infection](http://david.abcc.ncifcrf.gov/kegg.jsp?path=hsa05130$Pathogenic%20Escherichia%20coli%20infection&termId=470038898&source=kegg) | 11 | 0.8 | 4.5E-2 | 2.0 | 2.0E-1 |
| KEGG_PATHWAY | [Acute myeloid leukemia](http://david.abcc.ncifcrf.gov/kegg.jsp?path=hsa05221$Acute%20myeloid%20leukemia&termId=470038911&source=kegg) | 11 | 0.8 | 4.9E-2 | 2.0 | 2.2E-1 |
