# Supplementary material for: Genome-Wide Analysis of Differentially Expressed Genes and Splicing Isoforms in Clear Cell Renal Cell Carcinoma
Source: PLoS One. 2013 Oct 23;8(10):e78452. doi: 10.1371/journal.pone.0078452 (PMC3806822; doi:10.1371/journal.pone.0078452)
Supplement: Table S8 — Gene ontology enrichment analysis of genes down-regulated in ccRCC performed by DAVID. Annotations were considered significantly over-represented when the p-value of the Fisher's exact test as used by DAVID (EASE Score) was < 0.05 and gene counts belonging to an annotation term was equal or greater than 2. (DOCX) [file pone.0078452.s008.docx]

| [Category](http://david.abcc.ncifcrf.gov/chartReport.jsp?visited=yes&d-16544-s=1&cbBenjamini=true&rowids=&count=2&d-16544-o=2&cbFC=true&d-16544-p=1&annot=25&ease=0.1&numRecords=1000&heading=) | [Term](http://david.abcc.ncifcrf.gov/chartReport.jsp?visited=yes&d-16544-s=2&cbBenjamini=true&rowids=&count=2&d-16544-o=2&cbFC=true&d-16544-p=1&annot=25&ease=0.1&numRecords=1000&heading=) | [Count](http://david.abcc.ncifcrf.gov/chartReport.jsp?visited=yes&d-16544-s=5&cbBenjamini=true&rowids=&count=2&d-16544-o=1&cbFC=true&d-16544-p=1&annot=25&ease=0.1&numRecords=1000&heading=) | [%](http://david.abcc.ncifcrf.gov/chartReport.jsp?visited=yes&d-16544-s=6&cbBenjamini=true&rowids=&count=2&d-16544-o=1&cbFC=true&d-16544-p=1&annot=25&ease=0.1&numRecords=1000&heading=) | [P-Value](http://david.abcc.ncifcrf.gov/chartReport.jsp?visited=yes&d-16544-s=7&cbBenjamini=true&rowids=&count=2&d-16544-o=1&cbFC=true&d-16544-p=1&annot=25&ease=0.1&numRecords=1000&heading=) | [Fold Enrichment](http://david.abcc.ncifcrf.gov/chartReport.jsp?visited=yes&d-16544-s=8&cbBenjamini=true&rowids=&count=2&d-16544-o=1&cbFC=true&d-16544-p=1&annot=25&ease=0.1&numRecords=1000&heading=) | [Benjamini](http://david.abcc.ncifcrf.gov/chartReport.jsp?visited=yes&d-16544-s=9&cbBenjamini=true&rowids=&count=2&d-16544-o=1&cbFC=true&d-16544-p=1&annot=25&ease=0.1&numRecords=1000&heading=) |
| --- | --- | --- | --- | --- | --- | --- |
| GOTERM_BP_FAT | [oxidation reduction](http://www.ebi.ac.uk/QuickGO/GTerm?id=GO:0055114) | 116 | 10.1 | 2.5E-25 | 2.8 | 7.5E-22 |
| GOTERM_BP_FAT | [cofactor metabolic process](http://www.ebi.ac.uk/QuickGO/GTerm?id=GO:0051186) | 45 | 3.9 | 9.6E-14 | 3.6 | 1.4E-10 |
| GOTERM_BP_FAT | [coenzyme metabolic process](http://www.ebi.ac.uk/QuickGO/GTerm?id=GO:0006732) | 39 | 3.4 | 2.0E-13 | 4.0 | 2.0E-10 |
| GOTERM_BP_FAT | [ion transport](http://www.ebi.ac.uk/QuickGO/GTerm?id=GO:0006811) | 104 | 9.1 | 2.2E-13 | 2.1 | 1.6E-10 |
| GOTERM_BP_FAT | [transmembrane transport](http://www.ebi.ac.uk/QuickGO/GTerm?id=GO:0055085) | 77 | 6.7 | 4.9E-10 | 2.1 | 2.9E-7 |
| GOTERM_BP_FAT | [cation transport](http://www.ebi.ac.uk/QuickGO/GTerm?id=GO:0006812) | 73 | 6.4 | 4.3E-9 | 2.1 | 2.1E-6 |
| GOTERM_BP_FAT | [carboxylic acid catabolic process](http://www.ebi.ac.uk/QuickGO/GTerm?id=GO:0046395) | 27 | 2.4 | 4.9E-9 | 3.8 | 2.1E-6 |
| GOTERM_BP_FAT | [organic acid catabolic process](http://www.ebi.ac.uk/QuickGO/GTerm?id=GO:0016054) | 27 | 2.4 | 4.9E-9 | 3.8 | 2.1E-6 |
| GOTERM_BP_FAT | [excretion](http://www.ebi.ac.uk/QuickGO/GTerm?id=GO:0007588) | 19 | 1.7 | 1.0E-8 | 5.1 | 3.8E-6 |
| GOTERM_BP_FAT | [generation of precursor metabolites and energy](http://www.ebi.ac.uk/QuickGO/GTerm?id=GO:0006091) | 48 | 4.2 | 3.2E-8 | 2.4 | 1.1E-5 |
| GOTERM_BP_FAT | [vitamin metabolic process](http://www.ebi.ac.uk/QuickGO/GTerm?id=GO:0006766) | 20 | 1.7 | 1.5E-7 | 4.2 | 4.6E-5 |
| GOTERM_BP_FAT | [organic acid transport](http://www.ebi.ac.uk/QuickGO/GTerm?id=GO:0015849) | 29 | 2.5 | 1.8E-7 | 3.1 | 4.8E-5 |
| GOTERM_BP_FAT | [amine catabolic process](http://www.ebi.ac.uk/QuickGO/GTerm?id=GO:0009310) | 20 | 1.7 | 3.0E-7 | 4.0 | 7.5E-5 |
| GOTERM_BP_FAT | [carboxylic acid transport](http://www.ebi.ac.uk/QuickGO/GTerm?id=GO:0046942) | 28 | 2.4 | 5.4E-7 | 3.0 | 1.3E-4 |
| GOTERM_BP_FAT | [metal ion transport](http://www.ebi.ac.uk/QuickGO/GTerm?id=GO:0030001) | 59 | 5.1 | 6.3E-7 | 2.0 | 1.4E-4 |
| GOTERM_BP_FAT | [cellular amino acid catabolic process](http://www.ebi.ac.uk/QuickGO/GTerm?id=GO:0009063) | 18 | 1.6 | 8.4E-7 | 4.1 | 1.7E-4 |
| GOTERM_BP_FAT | [response to toxin](http://www.ebi.ac.uk/QuickGO/GTerm?id=GO:0009636) | 17 | 1.5 | 8.8E-7 | 4.4 | 1.6E-4 |
| GOTERM_BP_FAT | [anion transport](http://www.ebi.ac.uk/QuickGO/GTerm?id=GO:0006820) | 27 | 2.4 | 1.1E-6 | 3.0 | 1.9E-4 |
| GOTERM_BP_FAT | [pyruvate metabolic process](http://www.ebi.ac.uk/QuickGO/GTerm?id=GO:0006090) | 14 | 1.2 | 1.2E-6 | 5.2 | 2.0E-4 |
| GOTERM_BP_FAT | [glucose metabolic process](http://www.ebi.ac.uk/QuickGO/GTerm?id=GO:0006006) | 28 | 2.4 | 1.2E-6 | 2.9 | 1.9E-4 |
| GOTERM_BP_FAT | [hexose metabolic process](http://www.ebi.ac.uk/QuickGO/GTerm?id=GO:0019318) | 32 | 2.8 | 1.5E-6 | 2.6 | 2.3E-4 |
| GOTERM_BP_FAT | [cofactor catabolic process](http://www.ebi.ac.uk/QuickGO/GTerm?id=GO:0051187) | 12 | 1.0 | 1.7E-6 | 6.1 | 2.5E-4 |
| GOTERM_BP_FAT | [coenzyme catabolic process](http://www.ebi.ac.uk/QuickGO/GTerm?id=GO:0009109) | 11 | 1.0 | 2.2E-6 | 6.6 | 3.0E-4 |
| GOTERM_BP_FAT | [carbohydrate catabolic process](http://www.ebi.ac.uk/QuickGO/GTerm?id=GO:0016052) | 22 | 1.9 | 4.4E-6 | 3.2 | 5.8E-4 |
| GOTERM_BP_FAT | [monosaccharide metabolic process](http://www.ebi.ac.uk/QuickGO/GTerm?id=GO:0005996) | 34 | 3.0 | 4.5E-6 | 2.4 | 5.7E-4 |
| GOTERM_BP_FAT | [cellular aldehyde metabolic process](http://www.ebi.ac.uk/QuickGO/GTerm?id=GO:0006081) | 11 | 1.0 | 4.8E-6 | 6.2 | 5.8E-4 |
| GOTERM_BP_FAT | [cellular carbohydrate catabolic process](http://www.ebi.ac.uk/QuickGO/GTerm?id=GO:0044275) | 19 | 1.7 | 5.3E-6 | 3.5 | 6.1E-4 |
| GOTERM_BP_FAT | [monovalent inorganic cation transport](http://www.ebi.ac.uk/QuickGO/GTerm?id=GO:0015672) | 43 | 3.8 | 5.4E-6 | 2.1 | 6.1E-4 |
| GOTERM_BP_FAT | [fatty acid metabolic process](http://www.ebi.ac.uk/QuickGO/GTerm?id=GO:0006631) | 31 | 2.7 | 8.3E-6 | 2.5 | 8.9E-4 |
| GOTERM_BP_FAT | [nitrogen compound biosynthetic process](http://www.ebi.ac.uk/QuickGO/GTerm?id=GO:0044271) | 43 | 3.8 | 9.4E-6 | 2.1 | 9.8E-4 |
| GOTERM_BP_FAT | [inorganic anion transport](http://www.ebi.ac.uk/QuickGO/GTerm?id=GO:0015698) | 19 | 1.7 | 2.0E-5 | 3.2 | 2.0E-3 |
| GOTERM_BP_FAT | [secondary metabolic process](http://www.ebi.ac.uk/QuickGO/GTerm?id=GO:0019748) | 17 | 1.5 | 3.2E-5 | 3.4 | 3.1E-3 |
| GOTERM_BP_FAT | [cofactor biosynthetic process](http://www.ebi.ac.uk/QuickGO/GTerm?id=GO:0051188) | 19 | 1.7 | 3.6E-5 | 3.1 | 3.4E-3 |
| GOTERM_BP_FAT | [alcohol catabolic process](http://www.ebi.ac.uk/QuickGO/GTerm?id=GO:0046164) | 17 | 1.5 | 4.4E-5 | 3.3 | 4.0E-3 |
| GOTERM_BP_FAT | [amine biosynthetic process](http://www.ebi.ac.uk/QuickGO/GTerm?id=GO:0009309) | 17 | 1.5 | 4.4E-5 | 3.3 | 4.0E-3 |
| GOTERM_BP_FAT | [aerobic respiration](http://www.ebi.ac.uk/QuickGO/GTerm?id=GO:0009060) | 11 | 1.0 | 4.5E-5 | 4.9 | 4.0E-3 |
| GOTERM_BP_FAT | [acetyl-CoA catabolic process](http://www.ebi.ac.uk/QuickGO/GTerm?id=GO:0046356) | 9 | 0.8 | 5.5E-5 | 6.1 | 4.7E-3 |
| GOTERM_BP_FAT | [tricarboxylic acid cycle](http://www.ebi.ac.uk/QuickGO/GTerm?id=GO:0006099) | 9 | 0.8 | 5.5E-5 | 6.1 | 4.7E-3 |
| GOTERM_BP_FAT | [cellular amino acid biosynthetic process](http://www.ebi.ac.uk/QuickGO/GTerm?id=GO:0008652) | 13 | 1.1 | 6.6E-5 | 4.0 | 5.5E-3 |
| GOTERM_BP_FAT | [hexose biosynthetic process](http://www.ebi.ac.uk/QuickGO/GTerm?id=GO:0019319) | 10 | 0.9 | 9.4E-5 | 5.1 | 7.6E-3 |
| GOTERM_BP_FAT | [acetyl-CoA metabolic process](http://www.ebi.ac.uk/QuickGO/GTerm?id=GO:0006084) | 10 | 0.9 | 9.4E-5 | 5.1 | 7.6E-3 |
| GOTERM_BP_FAT | [coenzyme biosynthetic process](http://www.ebi.ac.uk/QuickGO/GTerm?id=GO:0009108) | 15 | 1.3 | 9.5E-5 | 3.4 | 7.5E-3 |
| GOTERM_BP_FAT | [gluconeogenesis](http://www.ebi.ac.uk/QuickGO/GTerm?id=GO:0006094) | 9 | 0.8 | 1.1E-4 | 5.6 | 8.3E-3 |
| GOTERM_BP_FAT | [energy derivation by oxidation of organic compounds](http://www.ebi.ac.uk/QuickGO/GTerm?id=GO:0015980) | 23 | 2.0 | 1.1E-4 | 2.5 | 8.5E-3 |
| GOTERM_BP_FAT | [protein tetramerization](http://www.ebi.ac.uk/QuickGO/GTerm?id=GO:0051262) | 11 | 1.0 | 1.2E-4 | 4.4 | 9.0E-3 |
| GOTERM_BP_FAT | [serine family amino acid metabolic process](http://www.ebi.ac.uk/QuickGO/GTerm?id=GO:0009069) | 9 | 0.8 | 1.5E-4 | 5.4 | 1.1E-2 |
| GOTERM_BP_FAT | [water-soluble vitamin metabolic process](http://www.ebi.ac.uk/QuickGO/GTerm?id=GO:0006767) | 11 | 1.0 | 1.6E-4 | 4.3 | 1.1E-2 |
| GOTERM_BP_FAT | [chemical homeostasis](http://www.ebi.ac.uk/QuickGO/GTerm?id=GO:0048878) | 55 | 4.8 | 1.8E-4 | 1.7 | 1.2E-2 |
| GOTERM_BP_FAT | [sodium ion transport](http://www.ebi.ac.uk/QuickGO/GTerm?id=GO:0006814) | 21 | 1.8 | 2.1E-4 | 2.5 | 1.4E-2 |
| GOTERM_BP_FAT | [regulation of hormone levels](http://www.ebi.ac.uk/QuickGO/GTerm?id=GO:0010817) | 23 | 2.0 | 2.3E-4 | 2.4 | 1.5E-2 |
| GOTERM_BP_FAT | [nitrogen compound catabolic process](http://www.ebi.ac.uk/QuickGO/GTerm?id=GO:0044270) | 14 | 1.2 | 2.7E-4 | 3.3 | 1.7E-2 |
| GOTERM_BP_FAT | [branched chain family amino acid metabolic process](http://www.ebi.ac.uk/QuickGO/GTerm?id=GO:0009081) | 7 | 0.6 | 3.0E-4 | 6.9 | 1.9E-2 |
| GOTERM_BP_FAT | [serine family amino acid biosynthetic process](http://www.ebi.ac.uk/QuickGO/GTerm?id=GO:0009070) | 6 | 0.5 | 3.5E-4 | 8.5 | 2.1E-2 |
| GOTERM_BP_FAT | [branched chain family amino acid catabolic process](http://www.ebi.ac.uk/QuickGO/GTerm?id=GO:0009083) | 6 | 0.5 | 3.5E-4 | 8.5 | 2.1E-2 |
| GOTERM_BP_FAT | [alcohol biosynthetic process](http://www.ebi.ac.uk/QuickGO/GTerm?id=GO:0046165) | 11 | 1.0 | 3.6E-4 | 3.9 | 2.1E-2 |
| GOTERM_BP_FAT | [isoprenoid metabolic process](http://www.ebi.ac.uk/QuickGO/GTerm?id=GO:0006720) | 11 | 1.0 | 3.6E-4 | 3.9 | 2.1E-2 |
| GOTERM_BP_FAT | [secretion](http://www.ebi.ac.uk/QuickGO/GTerm?id=GO:0046903) | 36 | 3.1 | 3.9E-4 | 1.9 | 2.3E-2 |
| GOTERM_BP_FAT | [chloride transport](http://www.ebi.ac.uk/QuickGO/GTerm?id=GO:0006821) | 13 | 1.1 | 4.0E-4 | 3.3 | 2.3E-2 |
| GOTERM_BP_FAT | [monosaccharide biosynthetic process](http://www.ebi.ac.uk/QuickGO/GTerm?id=GO:0046364) | 10 | 0.9 | 4.1E-4 | 4.2 | 2.3E-2 |
| GOTERM_BP_FAT | [retinoid metabolic process](http://www.ebi.ac.uk/QuickGO/GTerm?id=GO:0001523) | 8 | 0.7 | 4.2E-4 | 5.4 | 2.3E-2 |
| GOTERM_BP_FAT | [diterpenoid metabolic process](http://www.ebi.ac.uk/QuickGO/GTerm?id=GO:0016101) | 8 | 0.7 | 4.2E-4 | 5.4 | 2.3E-2 |
| GOTERM_BP_FAT | [pteridine and derivative metabolic process](http://www.ebi.ac.uk/QuickGO/GTerm?id=GO:0042558) | 8 | 0.7 | 4.2E-4 | 5.4 | 2.3E-2 |
| GOTERM_BP_FAT | [monovalent inorganic cation homeostasis](http://www.ebi.ac.uk/QuickGO/GTerm?id=GO:0055067) | 11 | 1.0 | 4.4E-4 | 3.8 | 2.4E-2 |
| GOTERM_BP_FAT | [ion homeostasis](http://www.ebi.ac.uk/QuickGO/GTerm?id=GO:0050801) | 45 | 3.9 | 4.5E-4 | 1.7 | 2.4E-2 |
| GOTERM_BP_FAT | [alditol metabolic process](http://www.ebi.ac.uk/QuickGO/GTerm?id=GO:0019400) | 8 | 0.7 | 7.3E-4 | 5.0 | 3.8E-2 |
| GOTERM_BP_FAT | [terpenoid metabolic process](http://www.ebi.ac.uk/QuickGO/GTerm?id=GO:0006721) | 8 | 0.7 | 7.3E-4 | 5.0 | 3.8E-2 |
| GOTERM_BP_FAT | [fat-soluble vitamin metabolic process](http://www.ebi.ac.uk/QuickGO/GTerm?id=GO:0006775) | 9 | 0.8 | 8.8E-4 | 4.3 | 4.5E-2 |
| GOTERM_BP_FAT | [cellular lipid catabolic process](http://www.ebi.ac.uk/QuickGO/GTerm?id=GO:0044242) | 14 | 1.2 | 9.5E-4 | 2.9 | 4.7E-2 |
| GOTERM_BP_FAT | [polyol catabolic process](http://www.ebi.ac.uk/QuickGO/GTerm?id=GO:0046174) | 4 | 0.3 | 9.9E-4 | 15.7 | 4.8E-2 |
| GOTERM_BP_FAT | [hormone metabolic process](http://www.ebi.ac.uk/QuickGO/GTerm?id=GO:0042445) | 17 | 1.5 | 1.1E-3 | 2.5 | 5.2E-2 |
| GOTERM_BP_FAT | [regulation of pH](http://www.ebi.ac.uk/QuickGO/GTerm?id=GO:0006885) | 9 | 0.8 | 1.1E-3 | 4.1 | 5.1E-2 |
| GOTERM_BP_FAT | [cellular respiration](http://www.ebi.ac.uk/QuickGO/GTerm?id=GO:0045333) | 16 | 1.4 | 1.2E-3 | 2.6 | 5.5E-2 |
| GOTERM_BP_FAT | [organic anion transport](http://www.ebi.ac.uk/QuickGO/GTerm?id=GO:0015711) | 10 | 0.9 | 1.6E-3 | 3.6 | 7.1E-2 |
| GOTERM_BP_FAT | [positive regulation of ATPase activity](http://www.ebi.ac.uk/QuickGO/GTerm?id=GO:0032781) | 5 | 0.4 | 1.6E-3 | 8.7 | 7.1E-2 |
| GOTERM_BP_FAT | [glycine metabolic process](http://www.ebi.ac.uk/QuickGO/GTerm?id=GO:0006544) | 6 | 0.5 | 1.8E-3 | 6.3 | 8.0E-2 |
| GOTERM_BP_FAT | [sulfate transport](http://www.ebi.ac.uk/QuickGO/GTerm?id=GO:0008272) | 6 | 0.5 | 1.8E-3 | 6.3 | 8.0E-2 |
| GOTERM_BP_FAT | [dicarboxylic acid transport](http://www.ebi.ac.uk/QuickGO/GTerm?id=GO:0006835) | 6 | 0.5 | 1.8E-3 | 6.3 | 8.0E-2 |
| GOTERM_BP_FAT | [purine nucleoside triphosphate metabolic process](http://www.ebi.ac.uk/QuickGO/GTerm?id=GO:0009144) | 18 | 1.6 | 1.9E-3 | 2.3 | 8.1E-2 |
| GOTERM_BP_FAT | [hydrogen transport](http://www.ebi.ac.uk/QuickGO/GTerm?id=GO:0006818) | 12 | 1.0 | 1.9E-3 | 3.0 | 8.2E-2 |
| GOTERM_BP_FAT | [folic acid and derivative metabolic process](http://www.ebi.ac.uk/QuickGO/GTerm?id=GO:0006760) | 6 | 0.5 | 2.5E-3 | 5.9 | 1.0E-1 |
| GOTERM_BP_FAT | [cellular amide metabolic process](http://www.ebi.ac.uk/QuickGO/GTerm?id=GO:0043603) | 11 | 1.0 | 2.6E-3 | 3.1 | 1.1E-1 |
| GOTERM_BP_FAT | [vitamin A metabolic process](http://www.ebi.ac.uk/QuickGO/GTerm?id=GO:0006776) | 7 | 0.6 | 2.6E-3 | 4.8 | 1.0E-1 |
| GOTERM_BP_FAT | [glycerol metabolic process](http://www.ebi.ac.uk/QuickGO/GTerm?id=GO:0006071) | 7 | 0.6 | 2.6E-3 | 4.8 | 1.0E-1 |
| GOTERM_BP_FAT | [ATP metabolic process](http://www.ebi.ac.uk/QuickGO/GTerm?id=GO:0046034) | 16 | 1.4 | 2.6E-3 | 2.4 | 1.0E-1 |
| GOTERM_BP_FAT | [cation homeostasis](http://www.ebi.ac.uk/QuickGO/GTerm?id=GO:0055080) | 32 | 2.8 | 2.7E-3 | 1.8 | 1.0E-1 |
| GOTERM_BP_FAT | [purine ribonucleoside triphosphate metabolic process](http://www.ebi.ac.uk/QuickGO/GTerm?id=GO:0009205) | 17 | 1.5 | 3.1E-3 | 2.3 | 1.2E-1 |
| GOTERM_BP_FAT | [homeostatic process](http://www.ebi.ac.uk/QuickGO/GTerm?id=GO:0042592) | 68 | 5.9 | 3.1E-3 | 1.4 | 1.2E-1 |
| GOTERM_BP_FAT | [polyol metabolic process](http://www.ebi.ac.uk/QuickGO/GTerm?id=GO:0019751) | 9 | 0.8 | 3.3E-3 | 3.5 | 1.2E-1 |
| GOTERM_BP_FAT | [amine transport](http://www.ebi.ac.uk/QuickGO/GTerm?id=GO:0015837) | 17 | 1.5 | 3.3E-3 | 2.3 | 1.2E-1 |
| GOTERM_BP_FAT | [ribonucleoside triphosphate metabolic process](http://www.ebi.ac.uk/QuickGO/GTerm?id=GO:0009199) | 17 | 1.5 | 3.3E-3 | 2.3 | 1.2E-1 |
| GOTERM_BP_FAT | [lipid catabolic process](http://www.ebi.ac.uk/QuickGO/GTerm?id=GO:0016042) | 22 | 1.9 | 3.4E-3 | 2.0 | 1.2E-1 |
| GOTERM_BP_FAT | [response to nutrient levels](http://www.ebi.ac.uk/QuickGO/GTerm?id=GO:0031667) | 24 | 2.1 | 3.6E-3 | 1.9 | 1.3E-1 |
| GOTERM_BP_FAT | [response to hormone stimulus](http://www.ebi.ac.uk/QuickGO/GTerm?id=GO:0009725) | 38 | 3.3 | 3.8E-3 | 1.6 | 1.3E-1 |
| GOTERM_BP_FAT | [cellular hormone metabolic process](http://www.ebi.ac.uk/QuickGO/GTerm?id=GO:0034754) | 11 | 1.0 | 3.8E-3 | 2.9 | 1.3E-1 |
| GOTERM_BP_FAT | [nucleoside triphosphate metabolic process](http://www.ebi.ac.uk/QuickGO/GTerm?id=GO:0009141) | 18 | 1.6 | 4.0E-3 | 2.2 | 1.4E-1 |
| GOTERM_BP_FAT | [NAD metabolic process](http://www.ebi.ac.uk/QuickGO/GTerm?id=GO:0019674) | 7 | 0.6 | 4.1E-3 | 4.4 | 1.4E-1 |
| GOTERM_BP_FAT | [glyoxylate metabolic process](http://www.ebi.ac.uk/QuickGO/GTerm?id=GO:0046487) | 4 | 0.3 | 4.5E-3 | 10.4 | 1.5E-1 |
| GOTERM_BP_FAT | [amino acid transport](http://www.ebi.ac.uk/QuickGO/GTerm?id=GO:0006865) | 14 | 1.2 | 4.5E-3 | 2.4 | 1.5E-1 |
| GOTERM_BP_FAT | [cellular amino acid derivative metabolic process](http://www.ebi.ac.uk/QuickGO/GTerm?id=GO:0006575) | 21 | 1.8 | 4.5E-3 | 2.0 | 1.5E-1 |
| GOTERM_BP_FAT | [proton transport](http://www.ebi.ac.uk/QuickGO/GTerm?id=GO:0015992) | 11 | 1.0 | 4.9E-3 | 2.8 | 1.6E-1 |
| GOTERM_BP_FAT | [dicarboxylic acid metabolic process](http://www.ebi.ac.uk/QuickGO/GTerm?id=GO:0043648) | 8 | 0.7 | 4.9E-3 | 3.7 | 1.6E-1 |
| GOTERM_BP_FAT | [oxidoreduction coenzyme metabolic process](http://www.ebi.ac.uk/QuickGO/GTerm?id=GO:0006733) | 10 | 0.9 | 5.2E-3 | 3.0 | 1.6E-1 |
| GOTERM_BP_FAT | [glutamine family amino acid metabolic process](http://www.ebi.ac.uk/QuickGO/GTerm?id=GO:0009064) | 10 | 0.9 | 5.2E-3 | 3.0 | 1.6E-1 |
| GOTERM_BP_FAT | [response to endogenous stimulus](http://www.ebi.ac.uk/QuickGO/GTerm?id=GO:0009719) | 40 | 3.5 | 6.6E-3 | 1.5 | 2.0E-1 |
| GOTERM_BP_FAT | [fatty acid catabolic process](http://www.ebi.ac.uk/QuickGO/GTerm?id=GO:0009062) | 8 | 0.7 | 6.9E-3 | 3.5 | 2.0E-1 |
| GOTERM_BP_FAT | [response to extracellular stimulus](http://www.ebi.ac.uk/QuickGO/GTerm?id=GO:0009991) | 25 | 2.2 | 7.1E-3 | 1.8 | 2.1E-1 |
| GOTERM_BP_FAT | [aromatic compound catabolic process](http://www.ebi.ac.uk/QuickGO/GTerm?id=GO:0019439) | 6 | 0.5 | 7.2E-3 | 4.7 | 2.1E-1 |
| GOTERM_BP_FAT | [vitamin biosynthetic process](http://www.ebi.ac.uk/QuickGO/GTerm?id=GO:0009110) | 7 | 0.6 | 7.4E-3 | 3.9 | 2.1E-1 |
| GOTERM_BP_FAT | [phosphorus metabolic process](http://www.ebi.ac.uk/QuickGO/GTerm?id=GO:0006793) | 82 | 7.2 | 7.5E-3 | 1.3 | 2.1E-1 |
| GOTERM_BP_FAT | [phosphate metabolic process](http://www.ebi.ac.uk/QuickGO/GTerm?id=GO:0006796) | 82 | 7.2 | 7.5E-3 | 1.3 | 2.1E-1 |
| GOTERM_BP_FAT | [organophosphate metabolic process](http://www.ebi.ac.uk/QuickGO/GTerm?id=GO:0019637) | 23 | 2.0 | 8.8E-3 | 1.8 | 2.4E-1 |
| GOTERM_BP_FAT | [purine ribonucleoside metabolic process](http://www.ebi.ac.uk/QuickGO/GTerm?id=GO:0046128) | 7 | 0.6 | 8.9E-3 | 3.8 | 2.4E-1 |
| GOTERM_BP_FAT | [purine nucleoside metabolic process](http://www.ebi.ac.uk/QuickGO/GTerm?id=GO:0042278) | 7 | 0.6 | 8.9E-3 | 3.8 | 2.4E-1 |
| GOTERM_BP_FAT | [regulation of heart contraction](http://www.ebi.ac.uk/QuickGO/GTerm?id=GO:0008016) | 12 | 1.0 | 9.4E-3 | 2.4 | 2.5E-1 |
| GOTERM_BP_FAT | [embryonic skeletal system development](http://www.ebi.ac.uk/QuickGO/GTerm?id=GO:0048706) | 12 | 1.0 | 9.4E-3 | 2.4 | 2.5E-1 |
| GOTERM_BP_FAT | [purine nucleoside triphosphate biosynthetic process](http://www.ebi.ac.uk/QuickGO/GTerm?id=GO:0009145) | 14 | 1.2 | 1.0E-2 | 2.2 | 2.6E-1 |
| GOTERM_BP_FAT | [nucleobase. nucleoside and nucleotide catabolic process](http://www.ebi.ac.uk/QuickGO/GTerm?id=GO:0034656) | 10 | 0.9 | 1.1E-2 | 2.7 | 2.8E-1 |
| GOTERM_BP_FAT | [nucleobase. nucleoside. nucleotide and nucleic acid catabolic process](http://www.ebi.ac.uk/QuickGO/GTerm?id=GO:0034655) | 10 | 0.9 | 1.1E-2 | 2.7 | 2.8E-1 |
| GOTERM_BP_FAT | [ATP biosynthetic process](http://www.ebi.ac.uk/QuickGO/GTerm?id=GO:0006754) | 13 | 1.1 | 1.1E-2 | 2.3 | 2.7E-1 |
| GOTERM_BP_FAT | [fatty acid oxidation](http://www.ebi.ac.uk/QuickGO/GTerm?id=GO:0019395) | 8 | 0.7 | 1.1E-2 | 3.2 | 2.7E-1 |
| GOTERM_BP_FAT | [lipid oxidation](http://www.ebi.ac.uk/QuickGO/GTerm?id=GO:0034440) | 8 | 0.7 | 1.1E-2 | 3.2 | 2.7E-1 |
| GOTERM_BP_FAT | [response to steroid hormone stimulus](http://www.ebi.ac.uk/QuickGO/GTerm?id=GO:0048545) | 22 | 1.9 | 1.1E-2 | 1.8 | 2.7E-1 |
| GOTERM_BP_FAT | [neurotransmitter metabolic process](http://www.ebi.ac.uk/QuickGO/GTerm?id=GO:0042133) | 6 | 0.5 | 1.1E-2 | 4.3 | 2.8E-1 |
| GOTERM_BP_FAT | [zinc ion homeostasis](http://www.ebi.ac.uk/QuickGO/GTerm?id=GO:0055069) | 4 | 0.3 | 1.1E-2 | 7.8 | 2.8E-1 |
| GOTERM_BP_FAT | [NADH metabolic process](http://www.ebi.ac.uk/QuickGO/GTerm?id=GO:0006734) | 4 | 0.3 | 1.1E-2 | 7.8 | 2.8E-1 |
| GOTERM_BP_FAT | [response to inorganic substance](http://www.ebi.ac.uk/QuickGO/GTerm?id=GO:0010035) | 23 | 2.0 | 1.2E-2 | 1.8 | 2.8E-1 |
| GOTERM_BP_FAT | [vitamin B6 metabolic process](http://www.ebi.ac.uk/QuickGO/GTerm?id=GO:0042816) | 3 | 0.3 | 1.2E-2 | 15.7 | 2.8E-1 |
| GOTERM_BP_FAT | [alditol catabolic process](http://www.ebi.ac.uk/QuickGO/GTerm?id=GO:0019405) | 3 | 0.3 | 1.2E-2 | 15.7 | 2.8E-1 |
| GOTERM_BP_FAT | [pyridoxine metabolic process](http://www.ebi.ac.uk/QuickGO/GTerm?id=GO:0008614) | 3 | 0.3 | 1.2E-2 | 15.7 | 2.8E-1 |
| GOTERM_BP_FAT | [pyridoxine biosynthetic process](http://www.ebi.ac.uk/QuickGO/GTerm?id=GO:0008615) | 3 | 0.3 | 1.2E-2 | 15.7 | 2.8E-1 |
| GOTERM_BP_FAT | [vitamin B6 biosynthetic process](http://www.ebi.ac.uk/QuickGO/GTerm?id=GO:0042819) | 3 | 0.3 | 1.2E-2 | 15.7 | 2.8E-1 |
| GOTERM_BP_FAT | [hexose catabolic process](http://www.ebi.ac.uk/QuickGO/GTerm?id=GO:0019320) | 11 | 1.0 | 1.2E-2 | 2.5 | 2.8E-1 |
| GOTERM_BP_FAT | [nicotinamide metabolic process](http://www.ebi.ac.uk/QuickGO/GTerm?id=GO:0006769) | 8 | 0.7 | 1.2E-2 | 3.1 | 2.9E-1 |
| GOTERM_BP_FAT | [ATP synthesis coupled proton transport](http://www.ebi.ac.uk/QuickGO/GTerm?id=GO:0015986) | 8 | 0.7 | 1.2E-2 | 3.1 | 2.9E-1 |
| GOTERM_BP_FAT | [energy coupled proton transport. down electrochemical gradient](http://www.ebi.ac.uk/QuickGO/GTerm?id=GO:0015985) | 8 | 0.7 | 1.2E-2 | 3.1 | 2.9E-1 |
| GOTERM_BP_FAT | [nicotinamide nucleotide metabolic process](http://www.ebi.ac.uk/QuickGO/GTerm?id=GO:0046496) | 8 | 0.7 | 1.2E-2 | 3.1 | 2.9E-1 |
| GOTERM_BP_FAT | [ribonucleotide metabolic process](http://www.ebi.ac.uk/QuickGO/GTerm?id=GO:0009259) | 18 | 1.6 | 1.2E-2 | 1.9 | 2.9E-1 |
| GOTERM_BP_FAT | [nucleoside triphosphate biosynthetic process](http://www.ebi.ac.uk/QuickGO/GTerm?id=GO:0009142) | 14 | 1.2 | 1.3E-2 | 2.1 | 2.9E-1 |
| GOTERM_BP_FAT | [phosphorylation](http://www.ebi.ac.uk/QuickGO/GTerm?id=GO:0016310) | 68 | 5.9 | 1.3E-2 | 1.3 | 2.9E-1 |
| GOTERM_BP_FAT | [monocarboxylic acid transport](http://www.ebi.ac.uk/QuickGO/GTerm?id=GO:0015718) | 9 | 0.8 | 1.3E-2 | 2.8 | 3.0E-1 |
| GOTERM_BP_FAT | [L-amino acid transport](http://www.ebi.ac.uk/QuickGO/GTerm?id=GO:0015807) | 6 | 0.5 | 1.3E-2 | 4.1 | 3.0E-1 |
| GOTERM_BP_FAT | [negative regulation of blood coagulation](http://www.ebi.ac.uk/QuickGO/GTerm?id=GO:0030195) | 6 | 0.5 | 1.3E-2 | 4.1 | 3.0E-1 |
| GOTERM_BP_FAT | [alkaloid metabolic process](http://www.ebi.ac.uk/QuickGO/GTerm?id=GO:0009820) | 8 | 0.7 | 1.4E-2 | 3.1 | 3.1E-1 |
| GOTERM_BP_FAT | [monosaccharide catabolic process](http://www.ebi.ac.uk/QuickGO/GTerm?id=GO:0046365) | 11 | 1.0 | 1.4E-2 | 2.4 | 3.1E-1 |
| GOTERM_BP_FAT | [sulfur metabolic process](http://www.ebi.ac.uk/QuickGO/GTerm?id=GO:0006790) | 15 | 1.3 | 1.5E-2 | 2.0 | 3.1E-1 |
| GOTERM_BP_FAT | [purine ribonucleotide metabolic process](http://www.ebi.ac.uk/QuickGO/GTerm?id=GO:0009150) | 17 | 1.5 | 1.5E-2 | 1.9 | 3.1E-1 |
| GOTERM_BP_FAT | [lipid biosynthetic process](http://www.ebi.ac.uk/QuickGO/GTerm?id=GO:0008610) | 32 | 2.8 | 1.5E-2 | 1.6 | 3.2E-1 |
| GOTERM_BP_FAT | [glycerolipid metabolic process](http://www.ebi.ac.uk/QuickGO/GTerm?id=GO:0046486) | 19 | 1.7 | 1.5E-2 | 1.8 | 3.2E-1 |
| GOTERM_BP_FAT | [protein oligomerization](http://www.ebi.ac.uk/QuickGO/GTerm?id=GO:0051259) | 20 | 1.7 | 1.5E-2 | 1.8 | 3.2E-1 |
| GOTERM_BP_FAT | [pyridine nucleotide metabolic process](http://www.ebi.ac.uk/QuickGO/GTerm?id=GO:0019362) | 8 | 0.7 | 1.6E-2 | 3.0 | 3.3E-1 |
| GOTERM_BP_FAT | [aspartate family amino acid metabolic process](http://www.ebi.ac.uk/QuickGO/GTerm?id=GO:0009066) | 6 | 0.5 | 1.6E-2 | 3.9 | 3.3E-1 |
| GOTERM_BP_FAT | [regulation of ATPase activity](http://www.ebi.ac.uk/QuickGO/GTerm?id=GO:0043462) | 5 | 0.4 | 1.6E-2 | 4.9 | 3.3E-1 |
| GOTERM_BP_FAT | [glutamate metabolic process](http://www.ebi.ac.uk/QuickGO/GTerm?id=GO:0006536) | 5 | 0.4 | 1.6E-2 | 4.9 | 3.3E-1 |
| GOTERM_BP_FAT | [water transport](http://www.ebi.ac.uk/QuickGO/GTerm?id=GO:0006833) | 5 | 0.4 | 1.6E-2 | 4.9 | 3.3E-1 |
| GOTERM_BP_FAT | [acyl-CoA metabolic process](http://www.ebi.ac.uk/QuickGO/GTerm?id=GO:0006637) | 5 | 0.4 | 1.6E-2 | 4.9 | 3.3E-1 |
| GOTERM_BP_FAT | [retinol metabolic process](http://www.ebi.ac.uk/QuickGO/GTerm?id=GO:0042572) | 4 | 0.3 | 1.6E-2 | 7.0 | 3.3E-1 |
| GOTERM_BP_FAT | [regulation of fibrinolysis](http://www.ebi.ac.uk/QuickGO/GTerm?id=GO:0051917) | 4 | 0.3 | 1.6E-2 | 7.0 | 3.3E-1 |
| GOTERM_BP_FAT | [response to nutrient](http://www.ebi.ac.uk/QuickGO/GTerm?id=GO:0007584) | 17 | 1.5 | 1.7E-2 | 1.9 | 3.3E-1 |
| GOTERM_BP_FAT | [response to metal ion](http://www.ebi.ac.uk/QuickGO/GTerm?id=GO:0010038) | 16 | 1.4 | 1.7E-2 | 1.9 | 3.4E-1 |
| GOTERM_BP_FAT | [triglyceride metabolic process](http://www.ebi.ac.uk/QuickGO/GTerm?id=GO:0006641) | 8 | 0.7 | 1.8E-2 | 2.9 | 3.5E-1 |
| GOTERM_BP_FAT | [cellular chemical homeostasis](http://www.ebi.ac.uk/QuickGO/GTerm?id=GO:0055082) | 36 | 3.1 | 1.8E-2 | 1.5 | 3.5E-1 |
| GOTERM_BP_FAT | [steroid biosynthetic process](http://www.ebi.ac.uk/QuickGO/GTerm?id=GO:0006694) | 12 | 1.0 | 1.9E-2 | 2.2 | 3.6E-1 |
| GOTERM_BP_FAT | [response to peptide hormone stimulus](http://www.ebi.ac.uk/QuickGO/GTerm?id=GO:0043434) | 18 | 1.6 | 1.9E-2 | 1.8 | 3.6E-1 |
| GOTERM_BP_FAT | [fluid transport](http://www.ebi.ac.uk/QuickGO/GTerm?id=GO:0042044) | 5 | 0.4 | 2.0E-2 | 4.6 | 3.7E-1 |
| GOTERM_BP_FAT | [response to drug](http://www.ebi.ac.uk/QuickGO/GTerm?id=GO:0042493) | 23 | 2.0 | 2.0E-2 | 1.7 | 3.7E-1 |
| GOTERM_BP_FAT | [cellular amino acid derivative biosynthetic process](http://www.ebi.ac.uk/QuickGO/GTerm?id=GO:0042398) | 9 | 0.8 | 2.0E-2 | 2.6 | 3.7E-1 |
| GOTERM_BP_FAT | [oxidative phosphorylation](http://www.ebi.ac.uk/QuickGO/GTerm?id=GO:0006119) | 13 | 1.1 | 2.2E-2 | 2.1 | 3.9E-1 |
| GOTERM_BP_FAT | [purine ribonucleoside triphosphate biosynthetic process](http://www.ebi.ac.uk/QuickGO/GTerm?id=GO:0009206) | 13 | 1.1 | 2.2E-2 | 2.1 | 3.9E-1 |
| GOTERM_BP_FAT | [leucine metabolic process](http://www.ebi.ac.uk/QuickGO/GTerm?id=GO:0006551) | 3 | 0.3 | 2.2E-2 | 11.7 | 3.9E-1 |
| GOTERM_BP_FAT | [protein homotetramerization](http://www.ebi.ac.uk/QuickGO/GTerm?id=GO:0051289) | 6 | 0.5 | 2.2E-2 | 3.6 | 3.9E-1 |
| GOTERM_BP_FAT | [negative regulation of coagulation](http://www.ebi.ac.uk/QuickGO/GTerm?id=GO:0050819) | 6 | 0.5 | 2.2E-2 | 3.6 | 3.9E-1 |
| GOTERM_BP_FAT | [cellular homeostasis](http://www.ebi.ac.uk/QuickGO/GTerm?id=GO:0019725) | 42 | 3.7 | 2.3E-2 | 1.4 | 3.9E-1 |
| GOTERM_BP_FAT | [female pregnancy](http://www.ebi.ac.uk/QuickGO/GTerm?id=GO:0007565) | 14 | 1.2 | 2.3E-2 | 2.0 | 3.9E-1 |
| GOTERM_BP_FAT | [urogenital system development](http://www.ebi.ac.uk/QuickGO/GTerm?id=GO:0001655) | 14 | 1.2 | 2.3E-2 | 2.0 | 3.9E-1 |
| GOTERM_BP_FAT | [cellular ion homeostasis](http://www.ebi.ac.uk/QuickGO/GTerm?id=GO:0006873) | 35 | 3.1 | 2.3E-2 | 1.5 | 4.0E-1 |
| GOTERM_BP_FAT | [ribonucleoside triphosphate biosynthetic process](http://www.ebi.ac.uk/QuickGO/GTerm?id=GO:0009201) | 13 | 1.1 | 2.3E-2 | 2.1 | 4.0E-1 |
| GOTERM_BP_FAT | [regulation of system process](http://www.ebi.ac.uk/QuickGO/GTerm?id=GO:0044057) | 30 | 2.6 | 2.4E-2 | 1.5 | 4.0E-1 |
| GOTERM_BP_FAT | [heterocycle catabolic process](http://www.ebi.ac.uk/QuickGO/GTerm?id=GO:0046700) | 11 | 1.0 | 2.4E-2 | 2.2 | 4.0E-1 |
| GOTERM_BP_FAT | [aromatic compound biosynthetic process](http://www.ebi.ac.uk/QuickGO/GTerm?id=GO:0019438) | 5 | 0.4 | 2.5E-2 | 4.3 | 4.0E-1 |
| GOTERM_BP_FAT | [regulation of blood coagulation](http://www.ebi.ac.uk/QuickGO/GTerm?id=GO:0030193) | 7 | 0.6 | 2.5E-2 | 3.0 | 4.1E-1 |
| GOTERM_BP_FAT | [ribonucleoside metabolic process](http://www.ebi.ac.uk/QuickGO/GTerm?id=GO:0009119) | 8 | 0.7 | 2.5E-2 | 2.7 | 4.1E-1 |
| GOTERM_BP_FAT | [regulation of neurotransmitter levels](http://www.ebi.ac.uk/QuickGO/GTerm?id=GO:0001505) | 10 | 0.9 | 2.6E-2 | 2.3 | 4.1E-1 |
| GOTERM_BP_FAT | [response to glucocorticoid stimulus](http://www.ebi.ac.uk/QuickGO/GTerm?id=GO:0051384) | 11 | 1.0 | 2.6E-2 | 2.2 | 4.1E-1 |
| GOTERM_BP_FAT | [glycolysis](http://www.ebi.ac.uk/QuickGO/GTerm?id=GO:0006096) | 8 | 0.7 | 2.8E-2 | 2.7 | 4.4E-1 |
| GOTERM_BP_FAT | [purine nucleotide metabolic process](http://www.ebi.ac.uk/QuickGO/GTerm?id=GO:0006163) | 20 | 1.7 | 2.8E-2 | 1.7 | 4.4E-1 |
| GOTERM_BP_FAT | [serine family amino acid catabolic process](http://www.ebi.ac.uk/QuickGO/GTerm?id=GO:0009071) | 4 | 0.3 | 2.9E-2 | 5.7 | 4.4E-1 |
| GOTERM_BP_FAT | [glutamine metabolic process](http://www.ebi.ac.uk/QuickGO/GTerm?id=GO:0006541) | 5 | 0.4 | 3.0E-2 | 4.1 | 4.5E-1 |
| GOTERM_BP_FAT | [glucose catabolic process](http://www.ebi.ac.uk/QuickGO/GTerm?id=GO:0006007) | 9 | 0.8 | 3.0E-2 | 2.4 | 4.5E-1 |
| GOTERM_BP_FAT | [fatty acid transport](http://www.ebi.ac.uk/QuickGO/GTerm?id=GO:0015908) | 6 | 0.5 | 3.0E-2 | 3.4 | 4.5E-1 |
| GOTERM_BP_FAT | [fatty acid beta-oxidation](http://www.ebi.ac.uk/QuickGO/GTerm?id=GO:0006635) | 6 | 0.5 | 3.0E-2 | 3.4 | 4.5E-1 |
| GOTERM_BP_FAT | [cellular carbohydrate biosynthetic process](http://www.ebi.ac.uk/QuickGO/GTerm?id=GO:0034637) | 10 | 0.9 | 3.1E-2 | 2.3 | 4.5E-1 |
| GOTERM_BP_FAT | [lipid modification](http://www.ebi.ac.uk/QuickGO/GTerm?id=GO:0030258) | 10 | 0.9 | 3.1E-2 | 2.3 | 4.5E-1 |
| GOTERM_BP_FAT | [steroid metabolic process](http://www.ebi.ac.uk/QuickGO/GTerm?id=GO:0008202) | 21 | 1.8 | 3.4E-2 | 1.6 | 4.8E-1 |
| GOTERM_BP_FAT | [skeletal system development](http://www.ebi.ac.uk/QuickGO/GTerm?id=GO:0001501) | 30 | 2.6 | 3.4E-2 | 1.5 | 4.9E-1 |
| GOTERM_BP_FAT | [ion transmembrane transport](http://www.ebi.ac.uk/QuickGO/GTerm?id=GO:0034220) | 8 | 0.7 | 3.5E-2 | 2.6 | 4.9E-1 |
| GOTERM_BP_FAT | [regulation of fatty acid metabolic process](http://www.ebi.ac.uk/QuickGO/GTerm?id=GO:0019217) | 8 | 0.7 | 3.5E-2 | 2.6 | 4.9E-1 |
| GOTERM_BP_FAT | [acylglycerol metabolic process](http://www.ebi.ac.uk/QuickGO/GTerm?id=GO:0006639) | 8 | 0.7 | 3.5E-2 | 2.6 | 4.9E-1 |
| GOTERM_BP_FAT | [isoprenoid biosynthetic process](http://www.ebi.ac.uk/QuickGO/GTerm?id=GO:0008299) | 5 | 0.4 | 3.5E-2 | 3.9 | 4.9E-1 |
| GOTERM_BP_FAT | [ethanol metabolic process](http://www.ebi.ac.uk/QuickGO/GTerm?id=GO:0006067) | 3 | 0.3 | 3.6E-2 | 9.4 | 5.0E-1 |
| GOTERM_BP_FAT | [ethanol oxidation](http://www.ebi.ac.uk/QuickGO/GTerm?id=GO:0006069) | 3 | 0.3 | 3.6E-2 | 9.4 | 5.0E-1 |
| GOTERM_BP_FAT | [monohydric alcohol metabolic process](http://www.ebi.ac.uk/QuickGO/GTerm?id=GO:0034308) | 3 | 0.3 | 3.6E-2 | 9.4 | 5.0E-1 |
| GOTERM_BP_FAT | [calcium ion transport](http://www.ebi.ac.uk/QuickGO/GTerm?id=GO:0006816) | 16 | 1.4 | 3.7E-2 | 1.8 | 5.1E-1 |
| GOTERM_BP_FAT | [neutral lipid metabolic process](http://www.ebi.ac.uk/QuickGO/GTerm?id=GO:0006638) | 8 | 0.7 | 3.8E-2 | 2.5 | 5.1E-1 |
| GOTERM_BP_FAT | [carboxylic acid biosynthetic process](http://www.ebi.ac.uk/QuickGO/GTerm?id=GO:0046394) | 17 | 1.5 | 3.9E-2 | 1.7 | 5.2E-1 |
| GOTERM_BP_FAT | [organic acid biosynthetic process](http://www.ebi.ac.uk/QuickGO/GTerm?id=GO:0016053) | 17 | 1.5 | 3.9E-2 | 1.7 | 5.2E-1 |
| GOTERM_BP_FAT | [carbohydrate transport](http://www.ebi.ac.uk/QuickGO/GTerm?id=GO:0008643) | 9 | 0.8 | 3.9E-2 | 2.3 | 5.2E-1 |
| GOTERM_BP_FAT | [protein homooligomerization](http://www.ebi.ac.uk/QuickGO/GTerm?id=GO:0051260) | 12 | 1.0 | 3.9E-2 | 2.0 | 5.2E-1 |
| GOTERM_BP_FAT | [peptidyl-threonine modification](http://www.ebi.ac.uk/QuickGO/GTerm?id=GO:0018210) | 5 | 0.4 | 4.1E-2 | 3.7 | 5.3E-1 |
| GOTERM_BP_FAT | [neutral amino acid transport](http://www.ebi.ac.uk/QuickGO/GTerm?id=GO:0015804) | 5 | 0.4 | 4.1E-2 | 3.7 | 5.3E-1 |
| GOTERM_BP_FAT | [kidney development](http://www.ebi.ac.uk/QuickGO/GTerm?id=GO:0001822) | 12 | 1.0 | 4.2E-2 | 2.0 | 5.4E-1 |
| GOTERM_BP_FAT | [post-embryonic development](http://www.ebi.ac.uk/QuickGO/GTerm?id=GO:0009791) | 10 | 0.9 | 4.2E-2 | 2.1 | 5.3E-1 |
| GOTERM_BP_FAT | [glycerol ether metabolic process](http://www.ebi.ac.uk/QuickGO/GTerm?id=GO:0006662) | 8 | 0.7 | 4.2E-2 | 2.5 | 5.3E-1 |
| GOTERM_BP_FAT | [response to corticosteroid stimulus](http://www.ebi.ac.uk/QuickGO/GTerm?id=GO:0031960) | 11 | 1.0 | 4.4E-2 | 2.0 | 5.5E-1 |
| GOTERM_BP_FAT | [regulation of coagulation](http://www.ebi.ac.uk/QuickGO/GTerm?id=GO:0050818) | 7 | 0.6 | 4.4E-2 | 2.7 | 5.5E-1 |
| GOTERM_BP_FAT | [sensory perception of sound](http://www.ebi.ac.uk/QuickGO/GTerm?id=GO:0007605) | 12 | 1.0 | 4.5E-2 | 1.9 | 5.5E-1 |
| GOTERM_BP_FAT | [heme metabolic process](http://www.ebi.ac.uk/QuickGO/GTerm?id=GO:0042168) | 5 | 0.4 | 4.8E-2 | 3.6 | 5.7E-1 |
| GOTERM_BP_FAT | [response to vitamin A](http://www.ebi.ac.uk/QuickGO/GTerm?id=GO:0033189) | 7 | 0.6 | 4.9E-2 | 2.6 | 5.8E-1 |
| GOTERM_BP_FAT | [gland development](http://www.ebi.ac.uk/QuickGO/GTerm?id=GO:0048732) | 15 | 1.3 | 4.9E-2 | 1.7 | 5.8E-1 |
