# Supplementary material for: Genome-Wide Analysis of Differentially Expressed Genes and Splicing Isoforms in Clear Cell Renal Cell Carcinoma
Source: PLoS One. 2013 Oct 23;8(10):e78452. doi: 10.1371/journal.pone.0078452 (PMC3806822; doi:10.1371/journal.pone.0078452)
Supplement: Table S9 — KEGG pathway enrichment analysis of genes down-regulated in ccRCC performed by DAVID. Annotations were considered significantly over-represented when the p-value of the Fisher's exact test as used by DAVID (EASE Score) was < 0.05 and gene counts belonging to an annotation term was equal or greater than 2. (DOCX) [file pone.0078452.s009.docx]

| [Category](http://david.abcc.ncifcrf.gov/chartReport.jsp?visited=yes&d-16544-s=1&cbBenjamini=true&rowids=&count=2&d-16544-o=2&cbFC=true&d-16544-p=1&annot=47&ease=0.1&numRecords=1000&heading=) | [Term](http://david.abcc.ncifcrf.gov/chartReport.jsp?visited=yes&d-16544-s=2&cbBenjamini=true&rowids=&count=2&d-16544-o=2&cbFC=true&d-16544-p=1&annot=47&ease=0.1&numRecords=1000&heading=) | [Count](http://david.abcc.ncifcrf.gov/chartReport.jsp?visited=yes&d-16544-s=5&cbBenjamini=true&rowids=&count=2&d-16544-o=1&cbFC=true&d-16544-p=1&annot=47&ease=0.1&numRecords=1000&heading=) | [%](http://david.abcc.ncifcrf.gov/chartReport.jsp?visited=yes&d-16544-s=6&cbBenjamini=true&rowids=&count=2&d-16544-o=1&cbFC=true&d-16544-p=1&annot=47&ease=0.1&numRecords=1000&heading=) | [P-Value](http://david.abcc.ncifcrf.gov/chartReport.jsp?visited=yes&d-16544-s=7&cbBenjamini=true&rowids=&count=2&d-16544-o=1&cbFC=true&d-16544-p=1&annot=47&ease=0.1&numRecords=1000&heading=) | [Fold Enrichment](http://david.abcc.ncifcrf.gov/chartReport.jsp?visited=yes&d-16544-s=8&cbBenjamini=true&rowids=&count=2&d-16544-o=1&cbFC=true&d-16544-p=1&annot=47&ease=0.1&numRecords=1000&heading=) | [Benjamini](http://david.abcc.ncifcrf.gov/chartReport.jsp?visited=yes&d-16544-s=9&cbBenjamini=true&rowids=&count=2&d-16544-o=1&cbFC=true&d-16544-p=1&annot=47&ease=0.1&numRecords=1000&heading=) |
| --- | --- | --- | --- | --- | --- | --- |
| KEGG_PATHWAY | [Valine, leucine and isoleucine degradation](http://david.abcc.ncifcrf.gov/kegg.jsp?path=hsa00280$Valine,%20leucine%20and%20isoleucine%20degradation&termId=470038748&source=kegg) | 25 | 2,2 | 1,9E-16 | 7,6 | 3,9E-14 |
| KEGG_PATHWAY | [Propanoate metabolism](http://david.abcc.ncifcrf.gov/kegg.jsp?path=hsa00640$Propanoate%20metabolism&termId=470038789&source=kegg) | 18 | 1,6 | 1,0E-11 | 7,5 | 8,7E-10 |
| KEGG_PATHWAY | [Pyruvate metabolism](http://david.abcc.ncifcrf.gov/kegg.jsp?path=hsa00620$Pyruvate%20metabolism&termId=470038787&source=kegg) | 17 | 1,5 | 8,3E-9 | 5,7 | 4,8E-7 |
| KEGG_PATHWAY | [Fatty acid metabolism](http://david.abcc.ncifcrf.gov/kegg.jsp?path=hsa00071$Fatty%20acid%20metabolism&termId=470038734&source=kegg) | 16 | 1,4 | 6,9E-8 | 5,3 | 3,0E-6 |
| KEGG_PATHWAY | [Citrate cycle (TCA cycle)](http://david.abcc.ncifcrf.gov/kegg.jsp?path=hsa00020$Citrate%20cycle%20(TCA%20cycle)&termId=470038726&source=kegg) | 14 | 1,2 | 1,1E-7 | 6,0 | 3,8E-6 |
| KEGG_PATHWAY | [Glycine, serine and threonine metabolism](http://david.abcc.ncifcrf.gov/kegg.jsp?path=hsa00260$Glycine,%20serine%20and%20threonine%20metabolism&termId=470038746&source=kegg) | 14 | 1,2 | 1,1E-7 | 6,0 | 3,8E-6 |
| KEGG_PATHWAY | [Glycolysis / Gluconeogenesis](http://david.abcc.ncifcrf.gov/kegg.jsp?path=hsa00010$Glycolysis%20/%20Gluconeogenesis&termId=470038725&source=kegg) | 19 | 1,7 | 1,8E-7 | 4,2 | 5,1E-6 |
| KEGG_PATHWAY | [Butanoate metabolism](http://david.abcc.ncifcrf.gov/kegg.jsp?path=hsa00650$Butanoate%20metabolism&termId=470038790&source=kegg) | 14 | 1,2 | 4,0E-7 | 5,5 | 1,0E-5 |
| KEGG_PATHWAY | [Arginine and proline metabolism](http://david.abcc.ncifcrf.gov/kegg.jsp?path=hsa00330$Arginine%20and%20proline%20metabolism&termId=470038752&source=kegg) | 16 | 1,4 | 4,3E-6 | 4,0 | 9,4E-5 |
| KEGG_PATHWAY | [Tryptophan metabolism](http://david.abcc.ncifcrf.gov/kegg.jsp?path=hsa00380$Tryptophan%20metabolism&termId=470038756&source=kegg) | 13 | 1,1 | 2,1E-5 | 4,3 | 4,0E-4 |
| KEGG_PATHWAY | [Lysine degradation](http://david.abcc.ncifcrf.gov/kegg.jsp?path=hsa00310$Lysine%20degradation&termId=470038751&source=kegg) | 13 | 1,1 | 6,0E-5 | 3,9 | 1,0E-3 |
| KEGG_PATHWAY | [beta-Alanine metabolism](http://david.abcc.ncifcrf.gov/kegg.jsp?path=hsa00410$beta-Alanine%20metabolism&termId=470038758&source=kegg) | 9 | 0,8 | 1,1E-4 | 5,5 | 1,8E-3 |
| KEGG_PATHWAY | [Histidine metabolism](http://david.abcc.ncifcrf.gov/kegg.jsp?path=hsa00340$Histidine%20metabolism&termId=470038753&source=kegg) | 10 | 0,9 | 1,7E-4 | 4,6 | 2,5E-3 |
| KEGG_PATHWAY | [Limonene and pinene degradation](http://david.abcc.ncifcrf.gov/kegg.jsp?path=hsa00903$Limonene%20and%20pinene%20degradation&termId=470038804&source=kegg) | 7 | 0,6 | 3,0E-4 | 6,7 | 4,0E-3 |
| KEGG_PATHWAY | [PPAR signaling pathway](http://david.abcc.ncifcrf.gov/kegg.jsp?path=hsa03320$PPAR%20signaling%20pathway&termId=470038821&source=kegg) | 15 | 1,3 | 4,6E-4 | 2,9 | 5,7E-3 |
| KEGG_PATHWAY | [Glycerolipid metabolism](http://david.abcc.ncifcrf.gov/kegg.jsp?path=hsa00561$Glycerolipid%20metabolism&termId=470038775&source=kegg) | 11 | 1,0 | 1,4E-3 | 3,3 | 1,7E-2 |
| KEGG_PATHWAY | [Retinol metabolism](http://david.abcc.ncifcrf.gov/kegg.jsp?path=hsa00830$Retinol%20metabolism&termId=470038801&source=kegg) | 12 | 1,0 | 1,8E-3 | 3,0 | 1,9E-2 |
| KEGG_PATHWAY | [Drug metabolism](http://david.abcc.ncifcrf.gov/kegg.jsp?path=hsa00982$Drug%20metabolism&termId=470038809&source=kegg) | 12 | 1,0 | 5,5E-3 | 2,6 | 5,5E-2 |
| KEGG_PATHWAY | [Ascorbate and aldarate metabolism](http://david.abcc.ncifcrf.gov/kegg.jsp?path=hsa00053$Ascorbate%20and%20aldarate%20metabolism&termId=470038731&source=kegg) | 6 | 0,5 | 6,6E-3 | 4,7 | 6,2E-2 |
| KEGG_PATHWAY | [Aldosterone-regulated sodium reabsorption](http://david.abcc.ncifcrf.gov/kegg.jsp?path=hsa04960$Aldosterone-regulated%20sodium%20reabsorption&termId=470038890&source=kegg) | 9 | 0,8 | 9,7E-3 | 2,9 | 8,5E-2 |
| KEGG_PATHWAY | [Metabolism of xenobiotics by cytochrome P450](http://david.abcc.ncifcrf.gov/kegg.jsp?path=hsa00980$Metabolism%20of%20xenobiotics%20by%20cytochrome%20P450&termId=470038808&source=kegg) | 11 | 1,0 | 1,2E-2 | 2,4 | 1,0E-1 |
| KEGG_PATHWAY | [Lysosome](http://david.abcc.ncifcrf.gov/kegg.jsp?path=hsa04142$Lysosome&termId=470038840&source=kegg) | 16 | 1,4 | 2,7E-2 | 1,8 | 2,0E-1 |
| KEGG_PATHWAY | [Cysteine and methionine metabolism](http://david.abcc.ncifcrf.gov/kegg.jsp?path=hsa00270$Cysteine%20and%20methionine%20metabolism&termId=470038747&source=kegg) | 7 | 0,6 | 3,8E-2 | 2,7 | 2,6E-1 |
| KEGG_PATHWAY | [Selenoamino acid metabolism](http://david.abcc.ncifcrf.gov/kegg.jsp?path=hsa00450$Selenoamino%20acid%20metabolism&termId=470038760&source=kegg) | 6 | 0,5 | 4,0E-2 | 3,1 | 2,7E-1 |
| KEGG_PATHWAY | [Tyrosine metabolism](http://david.abcc.ncifcrf.gov/kegg.jsp?path=hsa00350$Tyrosine%20metabolism&termId=470038754&source=kegg) | 8 | 0,7 | 4,3E-2 | 2,4 | 2,7E-1 |
