# Supplementary figures and images for: Genome-Wide Analysis of Differentially Expressed Genes and Splicing Isoforms in Clear Cell Renal Cell Carcinoma
Source: PLoS One. 2013 Oct 23;8(10):e78452. doi: 10.1371/journal.pone.0078452 (PMC3806822; doi:10.1371/journal.pone.0078452)

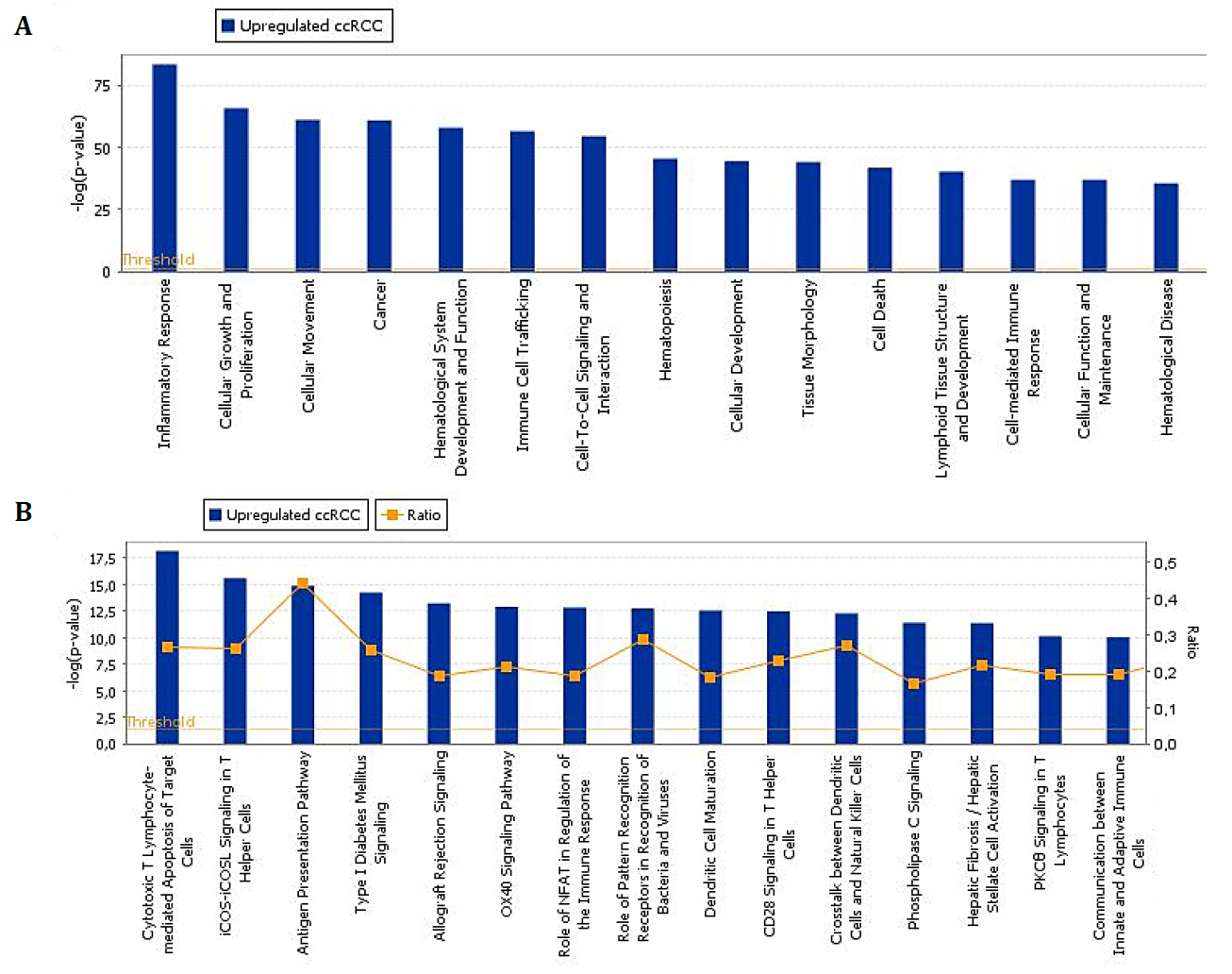

Supplement: Figure S1 — IPA analysis of up-regulated genes in ccRCC. Histograms show the top functions (A) and canonical pathways (B) enriched in the up-regulated data set, ranked for their significance. In the canonical pathway pane (B) the line graph shows the ratio of the number of molecules from the dataset that are in the pathway relative to the total number of molecules in the pathway (y-axis on the right). For both enrichment analyses significance threshold was set at -log(0.05). (TIF) [file pone.0078452.s011.tif]

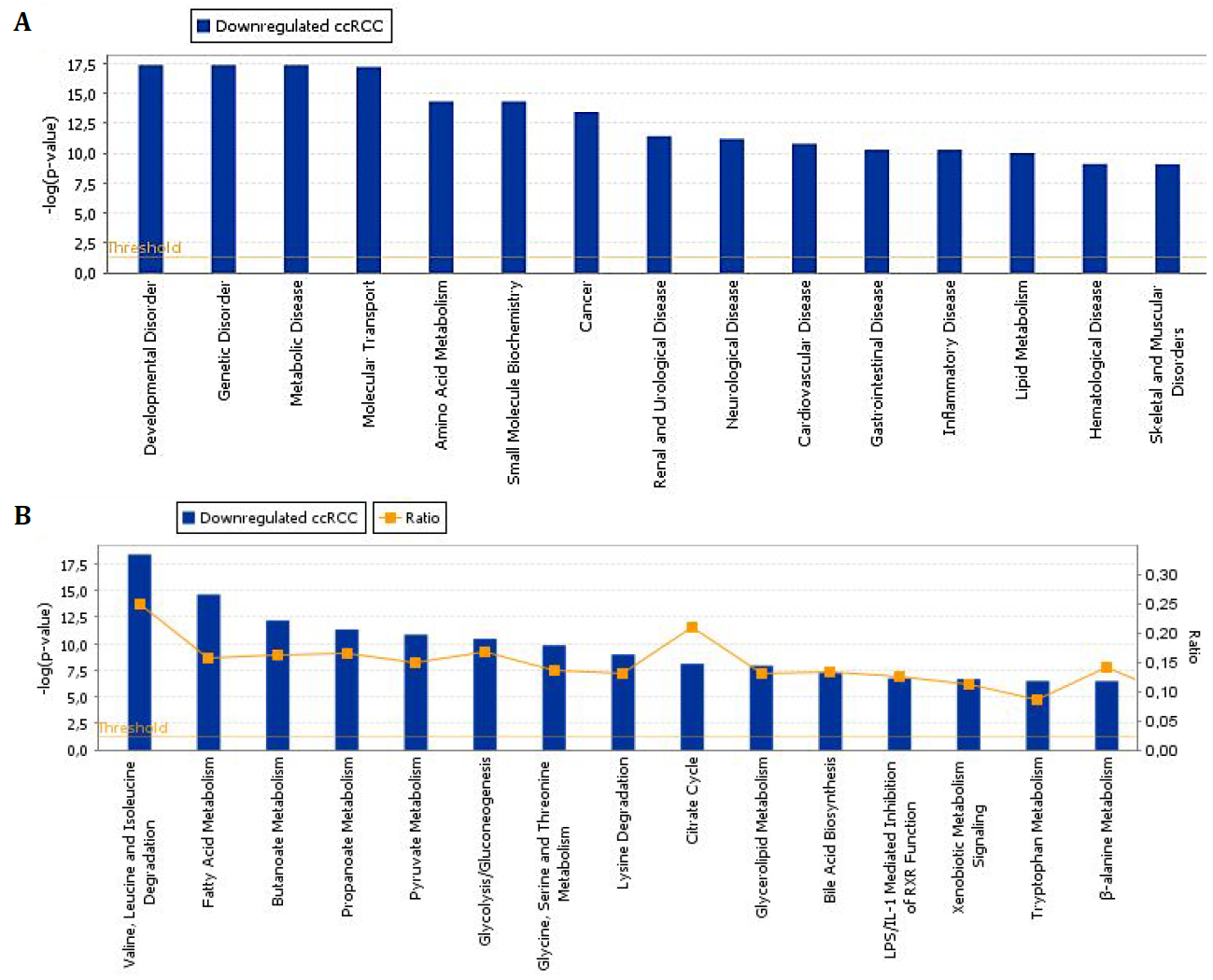

Supplement: Figure S2 — IPA analysis of down-regulated genes in ccRCC. Histograms show the top functions (A) and canonical pathways (B) enriched in the down-regulated data set, ranked for their significance. In the canonical pathway pane (B) the line graph shows the ratio of the number of molecules from the dataset that are in the pathway relative to the total number of molecules in the pathway (y-axis on the right). For both enrichment analyses significance threshold was set at -log(0.05). (TIF) [file pone.0078452.s012.tif]

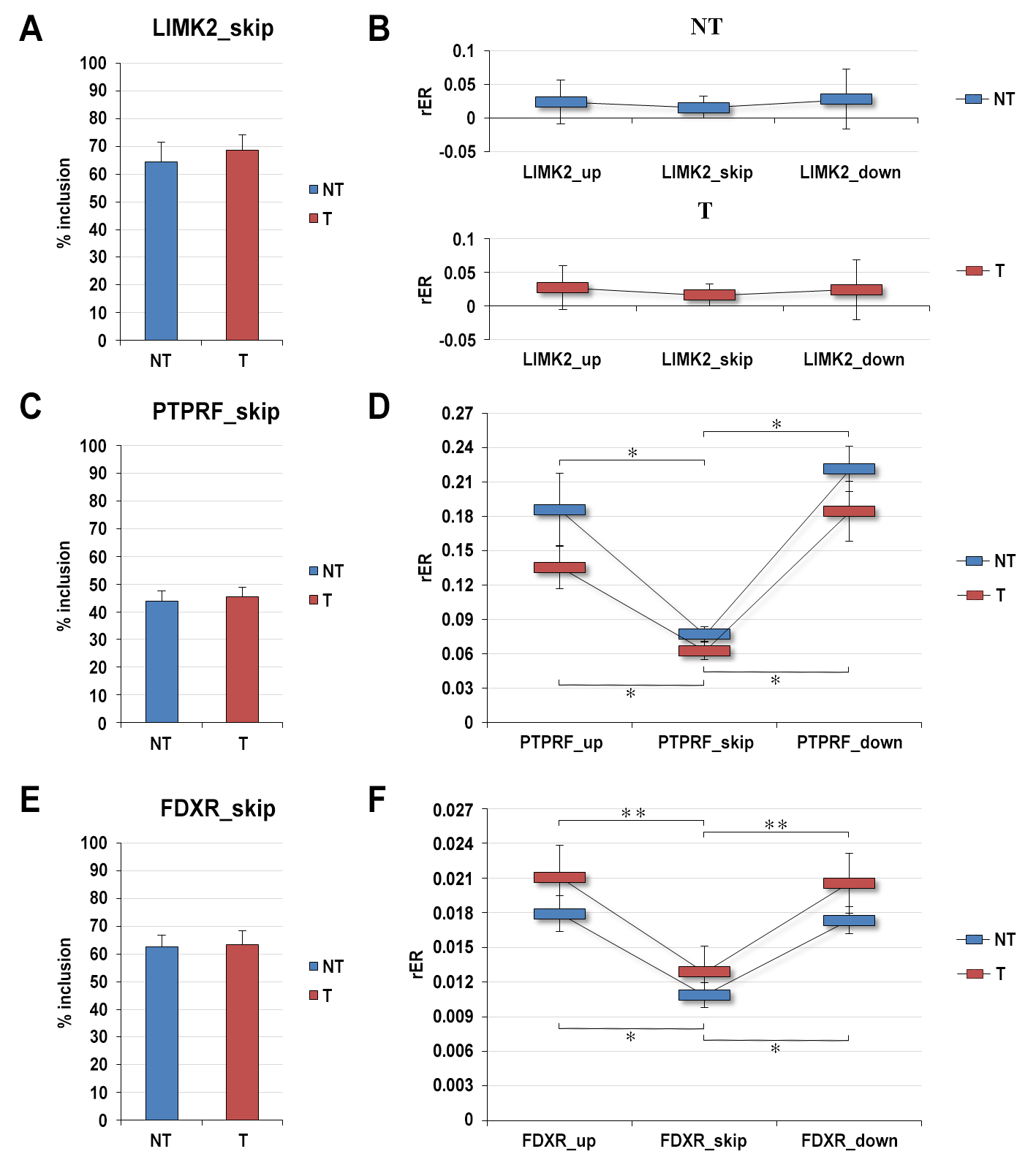

Supplement: Figure S3 — qRT-PCR analysis of differentially alternative splicing exons identified in LIMK2, PTPRF, and FDXR genes. Left panels (A, C and E) show the percentage of inclusion of the skipped exon in tumoral (T) and non-tumoral (NT) tissues, calculated as the average of the expression ratio of skipped exon relative to both upstream exon and the downstream one. Right panels (B, D and F) show the relative expression ratio (mean ± SE) of each exon of the triplet involved in the splicing event both in NT and T samples. Expression levels were calculated relative to the mean expression levels of ACTB and RPL13 genes. * = p-value < 0.01; ** = p-value < 0.05. (TIF) [file pone.0078452.s013.tif]
